# Supplementary material for: Self-directed learning in health professions: A mixed-methods systematic review of the literature
Source: PLoS One. 2025 May 2;20(5):e0320530. doi: 10.1371/journal.pone.0320530 (PMC12047769; doi:10.1371/journal.pone.0320530)
Supplement: S1 Appendix — (DOCX) [file pone.0320530.s001.docx]

Appendix S1 – Protocol

Self-directed learning in health professions: protocol for a systematic review

Administrative Information

1.Title: Self-directed learning in health professions: a systematic PRISMA review

2 Registration

The protocol for this review has not yet been registered.

3 Authors

Contact information of corresponding author

Linda Krista, B. Sc.

Master student in psychology

Email: linda.krista@students.unibe.ch / linda-krista@hotmail.com

Phone number: +41 76 434 36 74

Address: Haselmatte 2c, 6210 Sursee, Switzerland

Organizational affiliation of the review

This study is a collaboration between

• Institute of Psychology, University of Bern, Bern, Switzerland

• Institute for Medical Education, University of Bern, Bern, Switzerland

Collaborators and their organizational affiliations

Prof. Dr. phil. Sissel Guttormsen, Institute for Medical Education, University of Bern;

Prof. Dr. phil. Achim Elfering, Institute of Psychology, University of Bern

Review team members and their organizational affiliations

B. Sc. Linda Krista, Institute of Psychology, University of Bern;

Dr. med Artemisa Gogollari, Institute for Medical Education, University of Bern

4 Support

MSc, MA LIS Heidrun Janka, Information Specialist Medicine, Medical Library, University Library

Bern

5 Amendments

Any amendment of this protocol will be described and marked by date in this section.

• 17.07.2020: Research questions were refined based on discussions with the team

• 25.08.2020: Inclusion and exclusion criteria have been refined after piloting the developed

flowchart to a sample of 100 papers

• 28.09.2020: Change of decision not to split the articles for screening but that both reviewers

screen all articles because of the low inter-rater agreement

• 27.11.2020: With the growing understanding of matter, research question 1a “From a work

psychological perspective, what factors hamper and promote SDL in organizations in

general?”, research question 2a “What are empirical derived evidence-based outcomes

related to SDL in organizations in general?” and research question 4 “In which ways are

such models or factors applicable for the work situation of health professionals in relation to

SDL?” have become superfluous. Our search strategy does not support the answer to these

questions.

• 18.01.2021: Research question 1c was added.

Introduction

6 Rationale

Conceptual issues of Self-Directed Learning (SDL)

Over the years, various definitions and models of SDL have been established by a number of

scholars. A consistent key feature of SDL is the active role of the individual in the learning process.

SDL has been considered from various perspectives, namely the personal, process and contextual perspectives. The person perspective includes characteristics of the learner such as motivation, resource use, cognitive strategies, creativity, critical reflection, enthusiasm, life experience, life satisfaction, previous education, resilience and self-concept. The process perspective focuses on learner autonomy (manifested in the process of planning, monitoring and evaluating learnings), facilitation, learning skills, learning styles, teaching styles and technological skills. The contextual perspective encompasses environmental and sociopolitical factors such as the learning environment, support (feedback, peer collaboration, communication), culture, power, finances, gender, learning climate, organizational policies, political milieu, race, and sexual orientation. SDL is also influenced by ongoing digitalization.

Finally, different tools have been developed to measure the readiness for SDL.

*SDL in the perspective of work and organizational psychology*

To maximize and optimize the benefits of SDL in a workplace, it is important to better understand the factors that enhance SDL, such as contextual factors. Clardy (2000) model seems to be one of the few that explains the occurrence of SDL and its influencing factors using the theory of SDLPs. Research suggests positive outcomes of SDL in the workplace can include improved employee job effectiveness, increased job performance, savings in training costs while focusing also on the individual learner needs, greater scheduling flexibility, problem solving meta-skills, updating of skills and knowledge in highly specialized fields.

*SDL in health professions*

Knowledge in healthcare is constantly evolving. For health professionals it is a challenge to keep up to date in their field of expertise. An important driver for SDL is the constantly developing technology. To manage the information load and continual advancements in healthcare and to provide the best patient care, health professionals must commit to lifelong learning after their formal education. Most learning takes place in the workplace but there is little understanding of how to promote lifelong learning in this environment. SDL is considered an important approach for health professionals to achieve lifelong learning. Basically, the literature surrounding SDL in the context of health professional continuing education, confirms and mirrors insights from other fields. According to the findings presented above in section 2.3 there is potential to foster SDL systematically, which

does not seem to be actively utilized as yet.

7 Objectives

To understand the “mechanisms” of SDL and the degree to which they are used in a systematic way, we performed a systematic review. The specific aim of our review was to better understand the potential of SDL for the health professions from a work and organizational psychology perspective by finding and applying conceptual frameworks for SDL. The following overall research questions guided this work:

1) Which models exist that describe the broader context of professional SDL?

2) What do we know about SDL impact of on employees, quality of work and services?

3) How are the models applicable for the work situation of health professionals in relation to

SDL?

The following specific research questions have been deduced from the theoretical background

considered above.

Research question 1: What can we learn from models of SDL in the context of continued medical education?

a) Which models of SDL are commonly applied in health organizations?

b) Which models of SDL are reported in the work and organizational psychology literature in

general?

c) What do the results from 1a and 1b have in common?

Research question 2: What factors a) promote and b) hamper SDL in health organizations?

Research question 3: Which empirical derived evidence-based outcomes are related to SDL in health organizations?

Methods

8 Eligibility criteria

*Population:*

This review considered studies that included health professionals in clinical settings. The health professionals considered included those (clerkship students, residents, doctors, nurses, or other practicing health professionals) that treat patients or provide direct patient care of any type. A clinical setting refers to the location in which the primary purpose is the delivery of patient care (e.g. hospital).

*Phenomenon of interest:*

This review considered studies that examined or addressed SDL in any form and focused on

informal, intentional self-directed learning. Meaning “any self-initiated or directed set of activities with the primary purpose of learning about job, vocational, or occupational subjects” (Clardy, 2000, p. 109). The terms and definitions of SDL vary throughout the literature. In order not to lose important papers, we accepted the terms and definitions of the authors and also included studies that presented a SDL-like background.

9 Information sources

A systematic search was conducted using the following electronic databases:

- MEDLINE via Ovid (1946 - Present)
- Embase via Ovid (1974 - Present)
- PsycINFO via Ovid (1806 - Present)
- ERIC via Ovid (1965 - Present)
- Cochrane Library (1996 - Present)

In addition, Google Scholar was used for an unsystematic search for relevant gaps that the structured search indicated.

10 Search strategy (see Appendix S2)

11 Study records

*Data management*

The references identified through the searches were imported into a literature database using the literature management program EndNote X9 (The EndNote Team, 2013). The bibliographic software was used to manage and screen the citations which emerged from the electronic database search. After removing duplicated references in EndNote, the number of records retrieved was 2583. Seven further articles identified through Google Scholar were also imported into the EndNote library.

Selection process

Electronic database search

The literature recovered from the electronic database searched was screened in two stages. First, the abstracts and titles of the papers were screened according to predefined inclusion and exclusion criteria. Second, the full text of the included papers was retrieved and rescreened with the same inclusion and exclusion criteria. The more detailed screening process proceeded as described below.

Stage one

A flow chart (see Figure 2) with inclusion and exclusion criteria was developed for the first stage screening process and this was piloted by two reviewers on a sample of 100 articles.

Studies fulfilling the following eligibility criteria were included:

• SDL (in any form) was studied

• Health professionals as the target group

Exclusion criteria were:

• Neither the abstract nor the full text was available with reasonable effort

• Studies falling outside the SDL topic

• Formal education and learning

• Not a target group

• If the type of work was a conference abstract, report abstract, congress paper,

comment/reply, proceeding or reference material

Studies in languages other than English and German that could be translated adequately using Google translate were included if the inclusion criteria were met. Where articles were still present in duplicate, the articles were included and excluded once.

Based on the refined criteria after the pilot screening, four further rounds with 100 articles each were screened by both reviewers independently and categorized in EndNote as a) included or b) excluded.

Cohen's κ was run at every round to determine if there was agreement between two reviewer’s

judgement as to whether to include or exclude a study. According to Landis and Koch (1977)

benchmarks, the agreement between the two reviewers was moderate in the first round, κ =.59, and almost perfect, κ = .81 in the second. The third round the agreement was substantial, κ = .79. In the last round the agreement was moderate, κ = .55. After reconciliation in every round complete interrater agreement was reached. We infer that this process of inclusion or exclusion of an article required difficult judgements because of the vagueness of the SDL concept. Often, information in the abstract and title was insufficient to fully decide on inclusion or exclusion. In order to prevent the rejection of relevant papers, it was beneficial if both reviewers screened all articles. Therefore, the remaining articles were screened by both reviewers over four rounds of about 545 articles. Cohen's κ was again run for each. The inter-rater agreement was κ = .51 in the first round, κ = .56 in the second, κ = .53 in the third and κ = .5 in the final round. Discrepancies were resolved through discussion between the two reviewers after every round until complete agreement was reached. Where there was

uncertainty, the paper was included. Following the initial screening 180 papers progressed to the second assessment stage.

Stage two

In the second stage, the papers were read in full and selections were made based on the same

inclusion and exclusion criteria as previously. Articles were screened independently by both

reviewers in four rounds of about 45 articles. Again, each article was categorized in EndNote as a) included or b) excluded. Discrepancies were resolved through discussion between the two reviewers after every round until complete agreement was reached. Full texts that were not available with reasonable effort were excluded, e.g. when they could not be acquired due to high cost and/or long delivery times.

Unsystematic search in Google Scholar

The full text papers identified by internet searches via Google Scholar were looked through by the reviewers and included if they met eligibility criteria.

Inclusion criteria

• Inclusion criteria

• SDL model was presented

• Health professionals or another group of professionals were targeted

• Work and organizational psychology aspects were also addressed

12 Data extraction

Data collection process

A data extraction sheet based on the Cochrane Consumers and Communication Review Groups data extraction template was developed (Ryan, 2013). The template was pilot tested on ten randomly selected included studies and refined accordingly. In the final stage, the selected articles were sorted by study type before data was extracted by the author and entered into an Excel spreadsheet (see Appendix C). This permitted an overview of the content and an appreciation of any patterns between the included articles.

Data Items

The following information was extracted, if available, from the included studies:

• General Information: record number, reference title, author, year of publication, country of

publication, type of study and specific study classification

• Aim of study and research question(s)

• Method (e.g. design, operationalization, measuring instrument)

• Population studied: country of origin, professional background (e.g. clerkship, resident,

surgeon, nurse), type of setting (e.g. hospital, practice)

• Theoretical background of the study

• Description of SDL (e.g. term used, definition)

• Models of SDL

• Factors affecting SDL (promote and/or hamper)

• Evidence-based outcomes of SDL

• Other information: additional articles findings, conclusion of study authors, references to

other relevant studies, notes

13 Risk of quality of studies

All the journals publishing the included studies were cross-checked with the list of inappropriate journals (Stop Predatory Journals, n.d.). No studies appeared in such journals. Original peer-reviewed research articles were considered as good. The methods sections were checked in all articles for comprehensibility and good scientific practice. Where there was uncertainty, a senior collaborator was consulted, and the articles were read in full. Disagreements were resolved through consensus. In conclusion, all articles from the second screening were eligible with regard to the journal and quality.

14 Data synthesis

The entire process of article selection is shown in a PRISMA flow diagram in the results. A Table presents details of the included studies, including record number, general information, type of study and specific study classification, population, used term for SDL, model of SDL, factors of SDL and

evidence-based outcomes of SDL.

Outcomes and prioritization

Primary outcomes:

• Research questions 1-3

Secondary outcomes:

• Data driven
