# Supplementary material for: Self-directed learning in health professions: A mixed-methods systematic review of the literature
Source: PLoS One. 2025 May 2;20(5):e0320530. doi: 10.1371/journal.pone.0320530 (PMC12047769; doi:10.1371/journal.pone.0320530)
Supplement: S2 Appendix — (DOCX) [file pone.0320530.s002.docx]

S2 Appendix– Search Strategy

An information specialist from the University of Bern developed the specific search strategies based on our requirements as discussed in four meetings. An initial search was conducted by the information specialist at the University of Bern Medical Library, via Ovid MEDLINE. The search strategy was designed with controlled vocabulary and free text terms for concepts related to SDL in health professionals. After the first search, the MEDLINE search strategy was adapted to the syntax and subject headings of the other databases. The search for all above mentioned databases was run on June 25th, 2020. A search update was carried out on April 16^th^, 2024. The full search strategies for the final search are shown below.

The search strategy was based on the following considerations. From the methods section - **Theoretical background of SDL for building a research protocol**, it is clear that SDL plays an important role for health professionals, so this explains the AND operator between SDL and the health profession. It was also assumed that health profession organizations have a work and organizational psychological approach to SDL (at least in part), meaning for example that they are concerned about the organizational aspects of SDL.

This explains the AND operator between SDL in health professions and terms for work and organizational psychology. So, the assumption was that there is an overlap between SDL and work and organizational psychology in health professionals. This rationale was applied to the search in all the databases.

Ovid MEDLINE(R) ALL <1946 to April 15, 2024>

Date Run: 16/04/2024

ID Search Hits

--------------------------------------------------------------------------------

1 anatomists/ or exp anesthetists/ or audiologists/ or caregivers/ or exp dental staff/ or exp dentists/ or exp medical staff/ or exp nurses/ or exp nursing staff/ or exp personnel, hospital/ or physical therapists/ or exp physicians/ (473421)

2 ((healthcare or health) adj (personnel or professional? or provider?)).ab,kw,ti. (164327)

3 (medical adj (professional? or practitioner?)).ab,kw,ti. (18461)

4 (clinician? or clinical practice or clinical psychologist?).ab,kw,ti. (567362)

5 (physician* or general practitioner?).ab,kw,ti. (526312)

6 (nursing or nurse? or caregiv* or midwife* or midwives).ab,kw,ti. (639897)

7 (anatomist? or audiolog* or an?esthesiolog* or cardiolog* or dermatolog* or dentist* or endodontist* or endocrinolog* or gastroenterolog* or geriatri* or hospital* or nephrolog* or neurolog* or orthodontist* or oncolog* or ophthalmolog* or osteopath* or otolaryngolog* or patholog* or p?ediatric* or physiatrist* or pulmonolog* or radiolog* or rheumatolog* or surgeon* or surgery or urolog*).ab,kw,ti. (5295686)

8 or/1-7 (6592029)

9 Self-Directed Learning as Topic/ (106)

10 ((self-regulated or self-directed or autonomous or self-planned or self-initiated or informal or continuous or lifelong or workplace or independent) adj (learning or e-learning or learners)).ab,kw,ti. (6756)

11 ((self-direction or self-regulation) adj3 (learning or e-learning)).ab,kw,ti. (295)

12 or/9-11 (7017)

13 8 and 12 (3296)

14 exp Workplace/ or exp Health Facilities/ (942942)

15 (((workplace or work or working or hospital? or clinic? or ambulatory or health facilit* or care facilit* or care unit? or health centre? or health center? or organi?ation* or institution* or department? or management? or supervisor? or leadership?) adj3 (factor? or situation? or condition? or culture? or tolerance or style?)) or peer collaboration).ab,kw,ti. (115904)

16 14 or 15 (1038264)

17 Psychology, Industrial/ or Time Management/ or exp models, educational/ or exp models, organizational/ (34446)

18 (((business or economic? or organi?ational or occupational? or education*) adj (psycholog* or model?)) or work psychology or time management).ab,kw,ti. (15230)

19 17 or 18 (48100)

20 Professionalism/ or Clinical Competence/ or exp Evidence-Based Practice/ or education, dental, continuing/ or education, medical, continuing/ or education, nursing, continuing/ or education, professional, retraining/ or education, dental, graduate/ or education, medical, graduate/ or education, nursing, graduate/ or education, public health professional/ or exp Professional Practice/ or exp mental competency/ or exp attitude/ or human characteristics/ or exp motivation/ or exp personality/ (1535723)

21 (professionalism or professional practice? or competence* or medical education).ab,kw,ti. (145274)

22 ((learn* adj3 (motivation or autonom* or evaluation? or monitor*)) or resource use* or cognitive strateg* or critical reflection or life experience or resilience* or self-concept? or self-responsibility or self responsibility or self-reliance or self reliance or self-reliability or self reliability or learning skill? or teaching style? or learning style? or technolog* skill*).ab,kw,ti. (90076)

23 20 or 21 or 22 (1701914)

24 16 or 19 or 23 (2582177)

25 13 and 24 (2354)

Embase <1974 to 2024 April 15>

Date Run: 16/04/2024

ID Search Hits

--------------------------------------------------------------------------------

1 exp health practitioner/ (63307)

2 ((healthcare or health) adj (personnel or professional? or provider?)).ab,kw,ti. (220480)

3 (medical adj (professional? or practitioner?)).ab,kw,ti. (24562)

4 (clinician? or clinical practice or clinical psychologist?).ab,kw,ti. (816217)

5 (physician* or general practitioner?).ab,kw,ti. (745991)

6 (nursing or nurse? or caregiv* or midwife* or midwives).ab,kw,ti. (762267)

7 (anatomist? or audiolog* or an?esthesiolog* or cardiolog* or dermatolog* or dentist* or endodontist* or endocrinolog* or gastroenterolog* or geriatri* or hospital* or nephrolog* or neurolog* or orthodontist* or oncolog* or ophthalmolog* or osteopath* or otolaryngolog* or patholog* or p?ediatric* or physiatrist* or pulmonolog* or radiolog* or rheumatolog* or surgeon* or surgery or urolog*).ab,kw,ti. (7684598)

8 or/1-7 (9092857)

9 exp self-directed learning/ (1582)

10 ((self-regulated or self-directed or autonomous or self-planned or self-initiated or informal or continuous or lifelong or workplace or independent) adj (learning or elearning or learners)).ab,kw,ti. (8212)

11 ((self-direction or self-regulation) adj3 (learning or e-learning)).ab,kw,ti. (284)

12 or/9-11 (8893)

13 8 and 12 (4332)

14 exp workplace/ or exp health care facility/ (2073149)

15 (((workplace or work or working or hospital? or clinic? or ambulatory or health facilit* or care facilit* or care unit? or health centre? or health center? or organi?ation* or institution* or department? or management? or supervisor? or leadership?) adj3 (factor? or situation? or condition? or culture? or tolerance or style?)) or peer collaboration).ab,kw,ti. (149630)

16 14 or 15 (2186092)

17 exp educational model/ or exp "organization and management"/ (2608431)

18 (((business or economic? or organi?ational or occupational? or education*) adj (psycholog* or model?)) or work psychology or time management).ab,kw,ti. (19036)

19 17 or 18 (2619875)

20 exp professionalism/ or exp professional competence/ or exp clinical competence/ or exp evidence based practice/ or continuing education/ or continuing education provider/ or exp clinical education/ (2060645)

21 (professionalism or professional practice? or competence* or medical education).ab,kw,ti. (173948)

22 ((learn* adj3 (motivation or autonom* or evaluation? or monitor*)) or resource use* or cognitive strateg* or critical reflection or life experience or resilience* or selfconcept? or self concept? or self-responsibility or self responsibility or self-reliance or self reliance or self-reliability or self reliability or learning skill? or teaching style? or learning style? or technolog* skill*).ab,kw,ti. (107982)

23 20 or 21 or 22 (2290535)

24 16 or 19 or 23 (6083068)

25 13 and 24 (2990)

APA PsycInfo <1806 to April Week 1 2024>

Date Run: 16/04/2024

ID Search Hits

--------------------------------------------------------------------------------

1 exp health personnel/ (199945)

2 ((healthcare or health) adj (personnel or professional? or provider?)).ab,id,ti. (67987)

3 (medical adj (professional? or practitioner?)).ab,id,ti. (5284)

4 (clinician? or clinical practice or clinical psychologist?).ab,id,ti. (168274)

5 (physician* or general practitioner?).ab,id,ti. (81998)

6 (nursing or nurse? or caregiv* or midwife* or midwives).ab,id,ti. (188012)

7 (anatomist? or audiolog* or an?esthesiolog* or cardiolog* or dermatolog* or dentist* or endodontist* or endocrinolog* or gastroenterolog* or geriatri* or hospital* or nephrolog* or neurolog* or orthodontist* or oncolog* or ophthalmolog* or osteopath* or otolaryngolog* or patholog* or p?ediatric* or physiatrist* or pulmonolog* or radiolog* or rheumatolog* or surgeon* or surgery or urolog*).ab,id,ti. (465687)

8 or/1-7 (872286)

9 exp Individualized Instruction/ or self-regulated learning/ (6223)

10 ((self-regulated or self-directed or autonomous or self-planned or self-initiated or informal or continuous or lifelong or workplace or independent) adj (learning or elearning or learners)).ab,id,ti. (11261)

11 ((self-direction or self-regulation) adj3 (learning or e-learning)).ab,id,ti. (880)

12 or/9-11 (14833)

13 8 and 12 (1238)

14 exp organizational characteristics/ or organizational learning/ or exp working conditions/ or exp hospital environment/ (76842)

15 (((workplace or work or working or hospital? or clinic? or ambulatory or health facilit* or care facilit* or care unit? or health centre? or health center? or organi?ation* or institution* or department? or management? or supervisor? or leadership?) adj3 (factor? or situation? or condition? or culture? or tolerance or style?)) or peer collaboration).ab,id,ti. (68938)

16 14 or 15 (128597)

17 exp "industrial and organizational psychology"/ or exp time management/ or "quality of work life"/ (14739)

18 (((business or economic? or organi?ational or occupational? or education*) adj (psycholog* or model?)) or work psychology or time management).ab,id,ti. (37625)

19 17 or 18 (48195)

20 exp professional competence/ or exp evidence based practice/ or exp job performance/ or Continuing Education/ (62399)

21 (professionalism or professional practice? or competence* or continuing education).ab,id,ti. (86989)

22 ((learn* adj3 (motivation or autonom* or evaluation? or monitor*)) or resource use* or cognitive strateg* or critical reflection or life experience or resilience* or self concept? or self-responsibility or self responsibility or self-reliance or self reliance or self-reliability or self reliability or learning skill? or teaching style? or learning style? or technolog* skill*).ab,id,ti. (99668)

23 20 or 21 or 22 (234051)

24 16 or 19 or 23 (392093)

25 13 and 24 (475)

ERIC <1965 to February 2024>

Date Run: 16/04/2024

ID Search Hits

--------------------------------------------------------------------------------

1 exp health personnel/ (22734)

2 ((healthcare or health) adj (personnel or professional? or provider?)).ti,id,ab. (4911)

3 (medical adj (professional? or practitioner?)).ti,id,ab. (470)

4 (clinician? or clinical practice or clinical psychologist?).ti,id,ab. (7056)

5 (physician* or general practitioner?).ti,id,ab. (5907)

6 (nursing or nurse? or caregiv* or midwife* or midwives).ti,id,ab. (24045)

7 (anatomist? or audiolog* or an?esthesiolog* or cardiolog* or dermatolog* or dentist* or endodontist* or endocrinolog* or gastroenterolog* or geriatri* or hospital* or nephrolog* or neurolog* or orthodontist* or oncolog* or ophthalmolog* or osteopath* or otolaryngolog* or patholog* or p?ediatric* or physiatrist* or pulmonolog* or radiolog* or rheumatolog* or surgeon* or surgery or urolog*).ti,id,ab. (23757)

8 or/1-7 (68894)

9 independent study/ (8442)

10 ((self-regulated or self-directed or autonomous or self-planned or self-initiated or informal or continuous or lifelong or workplace or independent) adj (learning or elearning or learners)).ti,id,ab. (17084)

11 ((self-direction or self-regulation) adj3 (learning or e-learning)).ti,id,ab. (665)

12 or/9-11 (23129)

13 8 and 12 (692)

14 exp organization/ or exp organizational climate/ or exp organizational culture/ or exp organizational development/ or exp health facilities/ (134296)

15 (((workplace or work or working or hospital? or clinic? or ambulatory or health facilit* or care facilit* or care unit? or health centre? or health center? or organi?ation* or institution* or department? or management? or supervisor? or leadership?) adj3 (factor? or situation? or condition? or culture? or tolerance or style?)) or peer collaboration).ti,id,ab. (22539)

16 14 or 15 (151275)

17 work environment/ or exp industrial psychology/ or time management/ or exp job satisfaction/ or exp labor conditions/ or exp performance technology/ or exp professional autonomy/ or exp professional isolation/ or exp "quality of working life"/ or exp supervisor supervisee relationship/ or exp work ethic/ or exp workplace learning/ (35528)

18 (((business or economic? or organi?ational or occupational? or education*) adj (psycholog* or model?)) or work psychology or time management).ti,id,ab. (10933)

19 17 or 18 (44881)

20 exp competence/ or exp competency based education/ or exp expertise/ or exp performance/ or exp skills/ or professional continuing education/ or exp distance education/ or exp extension education/ or exp lifelong learning/ or exp postsecondary education/ or exp staff development/ or exp evidence based practice/ (627840)

21 (professionalism or professional practice? or competence* or continuing education).ti,id,ab. (51182)

22 ((learn* adj3 (motivation or autonom* or evaluation? or monitor*)) or resource use* or cognitive strateg* or critical reflection or life experience or resilience* or selfconcept? or self concept? or self-responsibility or self responsibility or self-reliance or self reliance or self-reliability or self reliability or learning skill? or teaching style? or learning style? or technolog* skill*).ti,id,ab. (47373)

23 or/20-22 (678994)

24 16 or 19 or 23 (799174)

25 13 and 24 (530)

Database: Cochrane Library

Date Run: 16/04/2024

ID Search Hits

--------------------------------------------------------------------------------

#1 MeSH descriptor: [Health Personnel] explode all trees (15455)

#2 (((healthcare or health) NEXT (personnel or professional? or provider?))):ti,ab,kw (17334)

#3 ((medical NEXT (professional? or practitioner?))):ti,ab,kw (937)

#4 ((clinician? or clinical practice or clinical psychologist?)):ti,ab,kw (104867)

#5 ((physician* or general practitioner?)):ti,ab,kw (63615)

#6 ((nursing or nurse? or caregiv* or midwife* or midwives)):ti,ab,kw (76107)

#7 ((anatomist? or audiolog* or an?esthesiolog* or cardiolog* or dermatolog* or dentist* or endodontist* or endocrinolog* or gastroenterolog* or geriatri* or hospital* or nephrolog* or neurolog* or orthodontist* or oncolog* or ophthalmolog* or osteopath* or otolaryngolog* or patholog* or p?ediatric* or physiatrist* or pulmonolog* or radiolog* or rheumatolog* or surgeon* or surgery or urolog*)):ti,ab,kw (665967)

#8 {OR #1-#7} (789045)

#9 MeSH descriptor: [Self-Directed Learning as Topic] explode all trees (1618)

#10 (((self-regulated or self-directed or autonomous or self-planned or self-initiated or informal or continuous or lifelong or workplace or independent) NEXT (learning or elearning or learners))):ti,ab,kw (500)

#11 (((self-direction or self-regulation) NEXT/2 (learning or e-learning))):ti,ab,kw (1284)

#12 {OR #9-#11} (3132)

#13 #8 AND #12 (1893)

#14 MeSH descriptor: [Workplace] explode all trees (1298)

#15 MeSH descriptor: [Health Facilities] explode all trees (22970)

#16 (((workplace or work or working or hospital? or clinic? or ambulatory or health facilit* or care facilit* or care unit? or health centre? or health center? or organi?ation* or institution* or department? or management? or supervisor? or leadership?) NEXT/3 (factor? or situation? or condition? or culture? or tolerance or style?)) or peer

collaboration):ti,ab,kw (37129)

#17 {OR #14-#16} (60747)

#18 MeSH descriptor: [Psychology, Industrial] this term only (12)

#19 MeSH descriptor: [Time Management] this term only (54)

#20 MeSH descriptor: [Models, Educational] explode all trees (373)

#21 MeSH descriptor: [Models, Organizational] this term only (241)

#22 ((((business or economic? or organi?ational or occupational? or education*) NEXT (psycholog* or model?)) or work psychology or time management)):ti,ab,kw (55899)

#23 {OR #18-#22} (56385)

#24 MeSH descriptor: [Professionalism] explode all trees (21)

#25 MeSH descriptor: [Clinical Competence] explode all trees (4951)

#26 MeSH descriptor: [Evidence-Based Practice] explode all trees (4009)

#27 MeSH descriptor: [Education, Continuing] explode all trees (1402)

#28 MeSH descriptor: [Education, Public Health Professional] explode all trees (3)

#29 MeSH descriptor: [Mental Competency] explode all trees (124)

#30 MeSH descriptor: [Attitude] explode all trees (50599)

#31 MeSH descriptor: [Human Characteristics] explode all trees (1)

#32 MeSH descriptor: [Motivation] explode all trees (12262)

#33 MeSH descriptor: [Personality] explode all trees (19945)

#34 ((professionalism or professional practice? or competence* or medical education)):ti,ab,kw (34006)

#35 (((learn* NEXT/2 (motivation or autonom* or evaluation? or monitor*)) or resource use* or cognitive strateg* or critical reflection or life experience or resilience* or selfconcept? or self-responsibility or self responsibility or self-reliance or self reliance or self-reliability or self reliability or learning skill? or teaching style? or learning style? or technolog* skill*)):ti,ab,kw (50693)

#36 {OR #24-#35} (143239)

#37 {OR #17, #23, #36} (235081)

#38 #13 AND #37 (1455)
