# Supplementary material for: Self-directed learning in health professions: A mixed-methods systematic review of the literature
Source: PLoS One. 2025 May 2;20(5):e0320530. doi: 10.1371/journal.pone.0320530 (PMC12047769; doi:10.1371/journal.pone.0320530)
Supplement: S3 Appendix — (DOCX) [file pone.0320530.s003.docx]

**S3 Appendix - SDL Concept definition**

We first performed a general literature review on the topic of SDL and related terms[^18^](#_ENREF_18). The idea of SDL was early known as self-education[^19^](#_ENREF_19)^,^ [^20^](#_ENREF_20). The term later evolved to informal self-initiated learning [^21-23^](#_ENREF_21), and is currently, in virtue of global digitalization, of particular relevance[^24^](#_ENREF_24)^,^ [^25^](#_ENREF_25). The following concepts are used interchangeable with SDL: *self-regulated learning*, *autonomous learning*, *self-teaching*, *self-planned learning*, *independent learning,* *self-initiated learning* and *informal learning*[^26-29^](#_ENREF_26). Existing models of SDL, described from work and organizational psychological perspective, aim at supporting the understanding of the complex relationship between a perceived personal need for knowledge gain and how the work environment may support or even hinder this [^15^](#_ENREF_15)^,^ [^30-32^](#_ENREF_30).

We adopted a comprehensive approach for this study by initially considering existing models that collectively provide diverse perspectives on SDL, spanning from individual personal attributes to dynamic processes involved in the learning journey. The inclusivity of these models is evident in their applicability across various educational contexts, encompassing not only traditional work environments but also specialized domains like medical education. This broad exploration allowed us to capture the multifaceted nature of SDL and understand its dynamics in diverse settings. While our primary focus is on healthcare, this broader perspective was a crucial foundation for identifying overarching principles and potential adaptations necessary for SDL within the specific context of healthcare education and practice. The deliberate inclusion of models from work and health psychology further enriched our understanding, strategically bridging external knowledge and enhancing the relevance of SDL within healthcare. The varied insights gained from these models contributed to a more nuanced understanding of SDL, laying the groundwork for subsequent analyses and considerations tailored to the unique demands of the healthcare field.

From the mentioned models, three dimensions emerge: *process*, *personal* and *contextual*[^20^](#_ENREF_20)^,^ [^22^](#_ENREF_22)^,^ [^32-34^](#_ENREF_32). The *process* perspective focuses on individual autonomy in learning and includes the teaching-learning transaction, facilitation, learning skills, learning styles, planning, organizing, and evaluating abilities, teaching styles, and technological skills. The *personal* perspective considers individual learner characteristics (i.e. learner capabilities in regulating the learning process) and includes creativity, critical reflection, enthusiasm, life experience, life satisfaction, motivation, previous education, resilience, and self-concept. The *contextual* perspective describes the environment in which SDL takes place and includes the environmental and socio-political climate, such as culture, power, learning environment, finances, gender, learning climate, organizational policies, political milieu, race, and sexual orientation[^35^](#_ENREF_35). The *personal*, *process* and *contextual* elements are recognized as equally important[^36^](#_ENREF_36).

Candy[^33^](#_ENREF_33) was one of the first to refer to the significance of the contextual perspective of SDL, namely the environment in which learning takes place. SDL is an important source of competitive advantage for organizations, as it encourages workers to take more responsibility for their own learning[^31^](#_ENREF_31)^,^ [^37^](#_ENREF_37). Such a learning and development-friendly environment can improve individual and organizational performance[^31^](#_ENREF_31)^,^ [^38^](#_ENREF_38)^,^ [^39^](#_ENREF_39), increased employee job effectiveness and significant training cost savings[^31^](#_ENREF_31)^,^ [^40^](#_ENREF_40)^,^ [^41^](#_ENREF_41). Relevant environmental and organizational factors that promote SDL include the presence of a participative management style, a supportive environment and the possibility to experiment unplanned, non-sequential learning activities[^37^](#_ENREF_37).

Known positive factors influencing SDL in the workplace include learning-committed leadership and management, an internal culture committed to learning, work tools, resources, and learning networks[^42^](#_ENREF_42). Of particular relevance is a working culture where feedback is common[^43^](#_ENREF_43) and positive attitudes from supervisors[^41^](#_ENREF_41). Negative organizational factors include leadership and management uncommitted to learning, a slow-changing internal culture of entitlement, poor work tools and resources, staff that disrupt learning networks, structural inhibitors, lack of time due to workload, rapid changes and absence of learning[^42^](#_ENREF_42).

Similar factors seem to influence SDL in the clinical setting[^30^](#_ENREF_30). Personal, contextual, and social attributes have been identified that can either promote or deter self-regulated learning in the clinical workplace. *Personal attributes* include skills in regulatory mechanisms, beliefs about learning, motivations, previous experiences and the perceived task level of a task all of which had effects as well as attributes relating to self-efficacy. *Contextual attributes* included curriculum, facilities, atmosphere, patient-related factors, available time, the people present and engagement in the team. *Social attributes* included familiarity and the nature of the relationship with those present in a particular setting, the experience in and motivation for teaching of others and whether there were possibilities for self-regulated learning.
