# Supplementary material for: Self-directed learning in health professions: A mixed-methods systematic review of the literature
Source: PLoS One. 2025 May 2;20(5):e0320530. doi: 10.1371/journal.pone.0320530 (PMC12047769; doi:10.1371/journal.pone.0320530)
Supplement: S4 Appendix — (DOCX) [file pone.0320530.s004.docx]

S4 Appendix: Extraction Form

|  | Author/Year/ Country | Type of study | Objectives | Outcomes | Participants (charac/total number) | Phenomenon of Interest (intervention) | Groups | Setting/  context | Data collection | Analysis | Results | Comments | Type of SDL Model |
| --- | --- | --- | --- | --- | --- | --- | --- | --- | --- | --- | --- | --- | --- |
| 1 | Acar, 2023[^65^](#_ENREF_65), Turkey | Cross-sectional  Observational | To evaluate the relationship between lifelong learning perceptions of pediatric nurses and self-confidence and anxiety in clinical decision-making processes. | Insights into how lifelong learning perceptions influence self-confidence and anxiety during clinical decision-making among pediatric nurses. | 218 pediatric nurses | assessing the perceptions of lifelong learning among pediatric nurses and its impact on their clinical decision-making processes. | pediatric nurses working in the pediatric clinics of a hospital in the capital of Turkey | Pediatric clinics of a hospital in the capital city of Turkey | 10 min face-to-face interviews  (Nursing Anxiety and Self-Confidence with Clinical Decision-Making Scale (NASC-CDM©) and  Lifelong Learning Scale | Descriptive and inferential statistics, regression analysis | Lifelong learning perceptions significantly predicted self-confidence in clinical decision-making but did not significantly predict anxiety levels in clinical decision-making | Small sample size, high attrition rate, not generalizable results | None |
| 2 | Allen, 2024[^66^](#_ENREF_66), USA | Qualitative  Narrative analysis | Explore physicians' lifelong learning journeys and their struggles with continuing professional development | Not explicitly listed | 12 pediatricians from a large children's hospital network including academic hospitals, community hospitals, and primary care practices | Examination of physicians' experiences across their professional careers, focusing on CPD | Pediatricians from various subspecialties | A range of settings across a hospital's local and regional network in the USA | one-on-one semi-structured narrative interviews | Holistic narrative analysis situated within a social constructivist orientation | Identified challenges in the transition from graduate medical education (GME) to CPD, emphasizing the need for more supportive learning environments. | The study highlights the need for more effective CPD systems that address real-world learning and support physicians throughout their careers. | None |
| 3 | Andersen, 2022[^51^](#_ENREF_51), Denmark | Qualitative phenomenological | How GPs learn POCUS and which barriers they encounter | ---- | 13 danish GPs | Ways how danish GPs learn POCUS | Danish GPs | Danish GPs | Semi-structured interviews Individual composition of US practice/education following a continuous process | Inductive thematic cross-case analysis (systematic text condensation) | Continuous learning process. Need for motivated GPs.Need for explicit recommendations | Relatively old data (4 y). Representation bias. | Individual level: Dreyfus Model – development from novice to expert  Organisational level: Lam framework |
| 4 | Antofie, 2023[^67^](#_ENREF_67), Romania | Cross-sectional observational study | To identify the opinions of Romanian pharmacists regarding job satisfaction, willingness to attend training courses, and institutional support. | Insights into pharmacists' job satisfaction levels, participation in training courses, and the effectiveness of institutional support. | 481 pharmacists from various areas of activity including hospitals, pharmaceutical chains, laboratories, or community pharmacies. | Exploring pharmacists' opinions on their professional life and support structures. | Pharmacists in different sectors (hospitals, chains, labs, community pharmacies) | Pharmacists in different sectors (hospitals, chains, labs, community pharmacies) | Questionnaires | Descriptive statistics and inferential analysis | Pharmacists expressed varying levels of job satisfaction, with specific dissatisfaction regarding salary levels but satisfaction with colleague relationships. Participation in professional development was considerable, with a focus on leadership and communication skills training. | The study stresses the need for continuous legislative and organizational support to enhance pharmacists' professional satisfaction and effective participation in lifelong learning. | None |
| 5 | Berg Jansson, 2022[^68^](#_ENREF_68), Sweden | Qualitative study | To analyze and discuss temporary agency nurses' experiences of working and learning from a workplace learning perspective in Sweden, focusing on the conditions for integrating work and learning in a work situation characterized by flexibilization and individualization. | Examination of the conditions for workplace learning among temporary agency nurses, including both opportunities and challenges associated with their unique work environment. | 21 temporary agency nurses working in Swedish healthcare, with experiences across various temporary work agencies and client organizations. | The work and learning experiences of temporary agency nurses as they move between different organizational contexts. | Temporary agency nurses within the Swedish healthcare system. | Various healthcare settings across Sweden where temporary agency nurses are employed. | in-depth interviews | Thematic Analysis | Identified enabling conditions such as diverse work experiences and recognized competencies, as well as constraining conditions such as limited participation in development work and varied support and feedback from client organizations | The study highlights the complexity of temporary agency work and the need for better conditions and support structures to enhance effective workplace learning among temporary agency nurses.  Type of SDL Model: | 3-P model" introduced by Tynjälä, which adapts Biggs’ discussion of presage, process, and product to a workplace context |
| 6 | Bing-Jonsson, 2023[^69^](#_ENREF_69), Norway | Observational Cross-Sectional | To report on a competence enhancement program implemented during the COVID-19 pandemic for nursing staff in a municipality in Norway to fill identified competence gaps. | Insights into the competence development of registered nurses (RNs) and assistant nurses (ANs) in institutional community health services after completing blended learning activities. | 96 nursing staff from a municipality in Norway, including RNs and ANs, participated in the initial and final assessments | Blended learning program consisting of e-learning courses, lectures, supervision, vocational training, and meetings with a superior. | Nursing staff (RNs and Ans) | Community healthcare settings within a Norwegian municipality. | Electronic questionnaire Nursing Older People—Competence Evaluation Tool (NOP-CET) for pre- and post-intervention competence measurement. | Descriptive and inferential statistics | Significant improvement in competencies, especially for assistant nurses, attributed to targeted learning activities addressing identified competence gaps.  Effectiveness of workplace-based blended learning programs for ongoing professional development and competence enhancement in nursing staff. | Small sample size, high attrition rate, not generalizable results | None |
| 7 | Bolton, 2022[^70^](#_ENREF_70), Paraguay | Cross-sectional Observational study | To determine the specific role that empathy and lifelong learning play in reducing occupational stress among healthcare professionals in adverse working conditions. | how empathy and lifelong learning impact the levels of somatization, exhaustion, and work alienation among healthcare professionals. | 80 healthcare professionals, including 40 physicians and 40 nurses, working in direct patient contact in ambulatory consultations at a public healthcare institution in Paraguay. | Measuring empathy and lifelong learning as predictors of occupational stress outcomes. | Physicians and nurses working in various departments such as family medicine, general surgery, pediatrics, gynecology and obstetrics, and nephrology. | Regional Hospital of Concepcion  , Concepcion City, Paraguay, in a setting characterized by high demand and adverse working conditions. | Paper-form questionnaire with the Jefferson Scale of Empathy (JSE), Jefferson Scale of Physician’s Lifelong Learning (JeffSPLL) and Scale of Collateral Effects (SCE) | Multiple regression analyses were used to determine the influence of empathy and lifelong learning on somatization, exhaustion, and work alienation. | Empathy and lifelong learning significantly influence the prevention of occupational stress, with their effects varying by medical discipline. The study found significant relationships between these variables and the measures of occupational stress (somatization, exhaustion, work alienation). | The study emphasizes the importance of fostering empathy and lifelong learning in healthcare settings, particularly in challenging environments, to mitigate the effects of occupational stress. | None |
| 8 | Brydges 2012[^28^](#_ENREF_28), Canada | Reflective analysis | Snapshot in the state of SRL – importance of self-regulation | Multi-component theoretical framework | Integrative overview | Current state of SDL | Integrative framework | Multi-component theoretical framework | Narrative review | Narrative review | Social context  Integrative perspective  Grain size  Best practices | Contributions and limitations of SRL  Integrative perspective | None |
| 9 | Chakkaravarthy, 2020[^52^](#_ENREF_52), Brunei | Observational Cross-sectional | Explore the determinants of readiness towards SDL among nurses of Brunei | Score in the Fisher’s Self-directed Learning Readiness Scale | 616 nurses | Readiness towards self-directed learning | Single cohort | Public acute care hospitals nationwide | Survey | Descriptive and inferential statistics | High level of readiness towards SDL  Higher scores in the self-control and desire for learning domains  More experience and level of educations predisposes to SDL | Large sample  Less representative for midwives  Factors influencing SDL: graduateness, workplace, marital status and age | None |
| 10 | Claponea, 2023[^71^](#_ENREF_71), Romania | Observational Cross-sectional | To evaluate the levels of organizational justice, social support, wellbeing, and lifelong learning associated with the level of burnout experienced by medical and non-medical staff from public and private medical units in Romania | understand how social support, organizational justice, and lifelong learning impact wellbeing and burnout among healthcare professionals | 497 healthcare professionals, including 367 medical personnel (nurses, physicians, and other medical specialists) and 130 non-medical staff (administrative personnel) | understand the levels of burnout and wellbeing among healthcare professionals and how these are influenced by social support, organizational justice, and lifelong learning | Two cohorts: medical personnel and non-medical (administrative) personnel | Public and private medical units across Romania | Assessment using Maslach Burnout Inventory, the WHO Wellbeing Index, the Multidimensional Scale of Perceived Social Support (MSPSS) and the Revised Jefferson Scale of Physician’s Lifelong Learning (JeffS- PLL) | Descriptive and inferential analysis | Found significant differences in burnout levels between medical and non-medical staff, with organizational justice playing a moderating role in the relationship between social support, lifelong learning, and wellbeing | importance of social support and organizational justice in mitigating burnout and enhancing wellbeing among healthcare professionals | None |
| 11 | Claret, 2020[^53^](#_ENREF_53), Spain | Qualitative  etnographic study  (Social constructivist position) | Analysis of everyday interactions in the workplace from the point of view of informal and peer-learning |  | Doctors from an OB-Gyn department of a level II hospital  (1 head, 3 veterans, 12 seniors, 4 residents, 1 midwive head, 10 midwives, 12 nurses and 1 secretary) | Exchange of knowledge in an organization | All members of the OB-Gyn department | Doctors from an OB-Gyn department of a level II hospital | Participant observation, ad hoc conversations and semi-structured interviews | Thematic analysis | First attempt to design workplace-learning strategies including the value of participation opportunities and influence of a dynamic concept of equality | Not generalisable  No quantitative information | -Garrick  -Mayer and Schmidt post-modernism – the social position of the person influences the experience of informal learning |
| 12 | Clouder, 2022[^72^](#_ENREF_72), UK | Qualitative | To explore and contextualize how practitioners in integrated care teams learn and train within their practice settings, particularly focusing on the influences of various ecological system levels on their learning processes. | Insights into the dynamics of practice-based education and training that underpin successful integrated care teams. | 27 participants from nine integrated care teams in the West Midlands region of the UK, consisting of managers, established team members, and relatively new team members | Examination of the nature and effectiveness of practice-based education and training across various integrated care teams using an ecological systems theory lens. | Healthcare practitioners from multiple disciplines working in integrated care settings. | Integrated care teams in various healthcare settings across the West Midlands, UK. | Semi-structured interviews | Thematic Analysis | The study identified themes: impact of shifting contexts within integrated teams on learning, influence of leadership on education and training, importance of informal learning, and role of interprofessional interactions in fostering a knowledge-sharing culture. | It points out the significant role of informal learning processes and the challenges related to sustainability and funding. | Bronfenbrenner's Ecological Systems Theory |
| 13 | Cuyvers, 2024[^73^](#_ENREF_73), the Netherlands | Qualitative | To enhance the conceptual understanding of self-regulated workplace learning (SRwpL) strategies and practices of nurses in clinical wards and offer insights for designing effective educational interventions supporting the development of nurses’ SRwpL. | Identification of self-regulatory strategies conditional for SRwpL in addition to strategies that initiate, progress, and evaluate the learning process in clinical settings. | 28 nurses from 6 different wards (gastro-intestinal medicine, emergency, vascular surgery, pediatrics, maternity, and geriatrics), head nurses from these wards, and learning counselors from a teaching hospital in northwest Netherlands. | Investigation of self-regulated learning strategies as engaged by nurses during their day-to-day practice in clinical wards. | Nurses, head nurses, and learning counselors involved in clinical education and practice. | A 400-bed teaching hospital in northwest Netherlands | observations, shadowing, and stimulated recall interviews | Qualitative Content analysis | The study revealed several self-regulatory strategies used by nurses which are crucial for effective SRwpL. However, these strategies are often sporadically engaged in, and there is a reported lack of variation and engagement in these strategies by head nurses and learning counselors. | The results highlight the need for more structured support and awareness among nurses and their educators to enhance the engagement in SRwpL strategies effectively. | Self-Regulation of Professional Learning (SRPL) model developed by Cuyvers et al. in 2021 |
| 14 | Fahlman, 2013[^54^](#_ENREF_54), Canada | Mixed-methods | Self-reported strategies for informal  Learning with mobile devices; How RN engage in informal learning using mobile devices | Frequency of strategies of informal learning using a mobile device, frequency of purposes of informal learning | 170 Registered Nurses | Characteristics of nurses using mobile devices for SDL | Single cohort  Registered nurses | Online survey of registered nurses; Semi-structured interviews | survey | Descriptive and inferential statistics, Thematic analysis | Positive perceptions with use of mobile devices  Minimal age-associated differences.  Use of devices for SDL, non-collaborative, professional development, patient teaching, maintaining competency | Exploratory nature of the study (need for confirmatory studies) | Marsick, Watkins, Callahan and Volpe informal and incidental learning model |
| 15 | Gathu, 2022[^74^](#_ENREF_74), Kenya | Narrative Review | To demonstrate the factors influencing the adoption of reflective learning for postgraduate students and highlight their importance to good clinical practice | facilitators and barriers to reflective learning: | ---- | Adoption of reflective learning in postgraduate medical education. | ----- | ----- | Literature review | Analysis of literature | Structural facilitators include curriculum alignment and flexibility in instruction. Assessment facilitators include multidimensional assessments. Relational facilitators include motivation and coaching. Barriers include rigid structures, ambiguous assessments, and insufficient motivation or training. | The study emphasizes the importance of flexibility and a supportive relational environment in promoting effective reflective learning. | none |
| 16 | Ghiyasvandian 2015 [^55^](#_ENREF_55), Iran | Qualitative  Case study | Explore nurses’ activities for SDL |  | 19 Registered Nurses | Nurses’ activities for SDL |  | Registered nurses in 4 hospitals from the same centre | Thematic analysis Content analysis | Semi-structured interviews | Two-step process of Kn. acquisition and skill development | Not generalizable | None |
| 17 | Hill, 2010[^56^](#_ENREF_56), USA | Mixed-Methods  (quantitative part: Cross-sectional observational study and Multi-case study) | self-directed learning activities that rural HCWrs undertake through practice-based clinical decision making for patient care how SDL is applied in practice through the clinical decision  making process | To analyse the types of information sources used by rural doctors | 150 physicians,  37 physician assistants  24 nurse practitioners | evaluate the use of information for clinical decision  making by healthcare professionals | Single cohort | Paper survey | Descriptive statistics Content analysis | Survey and Semi-structured interviews | Realization of surprise situations 🡪 movement to Stage 1  For specific care problems 🡪 move to stage 2 and 3 | The theory provides a framework for understanding the learning that takes place for rural practitioners | Slotnick’s four stage theory of self-directed learning |
| 18 | Kim, 2024[^75^](#_ENREF_75), South Korea | Quantitative  Feasibility study | To develop and evaluate a microlearning-based self-directed learning chatbot on medication administration for newly hired nurses | confidence in medication administration knowledge, intrinsic learning motivation, satisfaction with the chatbot's learning content, and usability ratings | 58 newly hired nurses on standby | A chatbot designed to support self-directed learning in medication administration for new nurses | Single group of newly hired nurses | clinical setting where the newly hired nurses were expected to start working | Two-phased methodology involving the development of the chatbot and its evaluation through surveys measuring confidence, motivation, satisfaction, and usability | Descriptive and inferential statistics | Participants showed increased confidence in medication administration knowledge across all topics except for "Understanding the concept of 5Right." They also reported high levels of intrinsic learning motivation, satisfaction with learning content, and found the chatbot easy to use. | A microlearning-based chatbot can effectively aid new nurses in improving their medication administration knowledge through self-directed learning | None |
| 19 | Kyndt, 2016[^57^](#_ENREF_57), Belgium | Cross-sectional observational | Determine which organisational learning conditions and individual  characteristics predict the learning outcomes nurses achieve through informal learning activities | perception of the available learning conditions, the learning outcomes they acquired by executing their  job and their self-efficacy, proactive personality and learning motivation | 203 Nurses | How selected learning conditions and personal characteristics predict  learning outcomes | Single cohort | Online survey | Questionnaire | Descriptive and inferential statistics, regression analysis | Regarding generic and job-specific learning outcomes, analyses identified the  same predictors for both levels of learning outcomes, being opportunities for  feedback and self-efficacy. More opportunities for feedback and higher self-efficacy  are associated with more job-specific and generic learning outcomes (more) |  | None |
| 20 | Lee, 2017[^58^](#_ENREF_58), South Korea (Dissertation) | Cross-sectional observational | Determine the factors that contribute to the lifelong workplace learning process of nurses in hospitals | Individual and organizational factors influencing informal learning | 221 nurses | What is the relationship of personal and organizational factors with nurses’ informal learning | Single cohort | Online survey | Questionnaire | Descriptive and inferential statistics, regression analysis | Informal learning level: 3.6  Individual level explains 84.6% of variance  Task variety, task significance, workplace friendship and learning motivation were significant | Shed light on which variables influence informal learning in nurses  Likely underpowered | Marsick & Watkins models for informal and incidental learning |
| 21 | Lim, 2021[^59^](#_ENREF_59), USA | Cross-sectional, Observational | Needs analysis to determine whether faculty feel equipped to foster SDL | % of “yes responses on the faculty needs assessment for fostering SDL (FNA-fSDL) scale and its dimensions | 339 Faculty members | Which learning approaches promote SDL | Single cohort | Online survey | Survey | Descriptive and inferential statistics | SDL essential for student success, it fosters lifelong skills  Teachers use metacognitive and affective approaches for SDL |  | Process- and learning oriented instruction |
| 22 | Lin, 2023[^76^](#_ENREF_76), Taiwan | Cross-Sectional  Observational | To explore the effects of job characteristics on physicians' lifelong learning using the job demands-control-support (JDCS) model. | relationships between job demands, job control, social support, and physicians' orientation toward lifelong learning. | 321 physicians from three medical centers in Taiwan | job characteristics as defined by the JDCS model (job demands, job control, social support) without a specific intervention. | Physicians were categorized based on job demand and control levels into four groups: active, passive, high strain, and low strain. | 3 medical centers across Taiwan. | questionnaire including the Chinese version of the Job Content Questionnaire (C-JCQ) and the revised Jefferson Scale of Physician Lifelong Learning (JeffSPLL) | Descriptive, inferential and regression statistics | Job demands and control, along with social support from supervisors and colleagues, were positively associated with lifelong learning. The interaction effects of these variables also significantly influenced lifelong learning orientation. | importance of considering job characteristics in educational strategies to enhance physicians' lifelong learning | Karasek’s Job Demands-Control-Support (JDCS) model |
| 23 | Liu, 2024[^77^](#_ENREF_77), China | Cross-Sectional  Observational | To compare and analyze the differences in clinical reasoning competence among nurses with varying years of experience and explore their relationship with self-directed learning competence. | differences in clinical reasoning and self-directed learning competences among nurses | 376 nurses from four independent hospitals in China | competences of clinical reasoning and self-directed learning without a specific intervention, focusing on natural variations among participants. | Nurses, grouped based on their years of work experience | multiple clinical settings within four independent hospitals in China. | online questionnaires | Descriptive, inferential and regression analyis | Higher clinical reasoning scores were found among nurses with more than ten years of experience, while variations in self-directed learning scores were noted based on experience years. The study also highlighted a positive correlation between the two competences. | Need for nursing managers to consider the development characteristics of these competences and tailor training strategies | Malcolm Knowles' SDL Model  Barry Zimmerman’s SDL Theory |
| 24 | Malekian, 2015[^60^](#_ENREF_60), Iran | Observational  Cross-sectional | Explore the determinants of readiness towards SDL among nurses of Iran | Score in the Fisher’s Self-directed Learning Readiness Scale | 297 nurses | Readiness towards self-directed learning | Single cohort | Public acute care hospitals in a region | Survey | Descriptive and inferential statistics | High level of readiness towards SDL  No correlation with age, gender, academics and marital status | Single cohort, use of self-reported scale, lack of qualitative data | none |
| 25 | Mamiya, 2023[^78^](#_ENREF_78), Japan | Observational  Cross-sectional | To explore Japanese pharmacists' perceptions of self-development skills and their attitudes towards continuing professional development. | To recognize the importance of self-development and CPD | 529 pharmacists from Japan. | The study focused on the pharmacists' perceptions of CPD and self-developmen | Pharmacists working in community and hospital settings | community and hospital pharmacies across Japan. | Survey | principal component analysis and general descriptive statistics | Most pharmacists acknowledged the necessity of ongoing self-development, with many indicating a willingness to engage in further education and training. The study identified specific areas where pharmacists felt they needed more training and development. | importance of integrating systematic CPD into the education of pharmacists | None |
| 26 | Papanagnou,  2022[^79^](#_ENREF_79)2022[^79^](#_ENREF_79)  USA | Qualitative  Scholarly Perspective/Conceptual Analysis | To introduce informal and incidental learning (IIL) as a framework for understanding how healthcare teams navigate complexity in clinical environments, especially under the stresses and uncertainties exacerbated by the COVID-19 pandemic | process of informal and incidental learning as it occurs naturally among healthcare teams dealing with complex and chaotic situations, particularly during the early stages of the COVID-19 pandemic | N/A | N/A | N/A | Clinical learning environments within healthcare settings during the COVID-19 pandemic | Conceptual analysis supported by literature review and theoretical interpretation | Use of the Marsick and Watkins model to interpret existing concepts and the authors' observations of learning behaviors during the pandemic | The paper argues that understanding and facilitating informal and incidental learning is crucial for managing the inherent uncertainty and complexity of clinical practice, particularly in times of crisis such as the COVID-19 pandemic | The authors call for a greater recognition of informal and incidental learning processes in clinical education and suggest integrating these concepts into training and development frameworks to better prepare healthcare professionals for future challenges | Marsick and Watkins model of Informal and Incidental Learning |
| 27 | Sockalingam, 2022[^80^](#_ENREF_80), Canada | Pre-post study | To explore the impact of participation in the ECHO Ontario Mental Health (ECHO-ONMH) program on interprofessional healthcare providers’ orientation towards lifelong learning | The study found an increase in the orientation to lifelong learning among participants after the ECHO program, with greater effects observed in more frequent participants | 67 Healthcare professionals enrolled in the ECHO-ONMH program | Participation in the ECHO-ONMH program, which utilizes a virtual tele-education model to enhance capacity in mental health care. | Participants were classified as ‘high’ or ‘low’ users based on median session attendance. | The ECHO-ONMH program, a virtual continuing professional development and capacity-building program offered through the Centre for Addiction and Mental Health and the University of Toronto | pre- and post-program surveys, employing the Jefferson Scale of Lifelong Learning. | Descriptive and inferential statistics | Statistically significant increases in lifelong learning orientation were observed, particularly among high-frequency participants. | The results highlight the potential of virtual CPD programs like Project ECHO to enhance lifelong learning among health professionals | self-determination theory (SDT) |
| 28 | Strachan, 2015[^61^](#_ENREF_61), UK (Editorial) | Editorial | Discussion about the importance of self-regulated learning | N/A | N/A | N/A | N/A | N/A | N/A | N/A | Self-regulated learning applied to clinical practice |  | White and Grupen (cyclical nature of SDL) |
| 29 | Sturesson-Sabel, 2021[^62^](#_ENREF_62), Sweden | Narrative review (Overview) | provide an overview of  the state of knowledge and research on doctors' continuing education  and learning | N/A | N/A | overview of  the state of knowledge and research on doctors' continuing education  and learning | N/A | N/A | Narrative review | N/A | methods for  to systematically evaluate the effects of physician training  after achieving specialist competence are needed |  | None |
| 30 | Taylor, 2015[^63^](#_ENREF_63), USA | Observational  Cross-sectional | Explore the relationship between orientation toward LLL, pursuit of knowledge and perceived professional competence | Score of the JeffsPLL scale  Score of the abb Personal Competencies Scale (PCS) | 136 members of the Illinois Psychological Association | N/A | Single cohort | members of a Psychological Association | Online Survey | Descriptive and inferential statistics | Correlation between JeffsPLL and PCS | Caucasian psychologists  Self-assessment measures  LLL may be a relevant component of a psychologist’s professional identity | None |
| 31 | Yang, 2023[^81^](#_ENREF_81), China | Cross-sectional  Observational | To examine the status quo of self-regulated learning among Chinese master of nursing specialists, explore the relationships between self-regulated learning, mindful agency, and psychological resilience, and investigate how mindful agency and psychological resilience influence self-regulated learning. | explore the relationships between self-regulated learning, mindful agency, and psychological resilience | 216 Master of Nursing Specialists from higher nursing institutions in Shandong, Liaoning, and other regions. | iIteraction between self-regulated learning, mindful agency, and psychological resilience without a specific intervention. | Master of Nursing Specialists | Various regions of China, involving those engaged in clinical practice | Online surveys | Descriptive and inferential statistics,  regression analysis | The study highlighted the crucial role of mindful agency and psychological resilience in supporting self-regulated learning among nursing specialists in clinical settings. | Need for clinical education programs to consider these psychological factors to enhance the self-regulated learning capabilities of nursing specialists. | None |
| 32 | Yao, 2023[^82^](#_ENREF_82), China | Qualitative | To investigate SDL strategies used by hospital pharmacists in depth, providing them with a reference for the development of their SDL skills. | The study summarized 12 learning strategies related to SDL, which were grouped into four themes: use of information resources, application of cognitive strategies, development of learning plans, and use of learning platforms. | 17 hospital pharmacists from 3 tertiary hospitals in Henan province, China. | The study focused on the self-directed learning strategies employed by hospital pharmacists without a specific intervention | hospital pharmacists, including dispensing pharmacists, clinical pharmacists, and pharmacy department managers. | 3 large tertiary hospitals in Henan province, China. | one-on-one interviews and focus group discussions | Thematic analysis | Hospital pharmacists utilize a range of SDL strategies, involving both traditional learning methods and contemporary digital resources, to enhance their professional knowledge and skills. | The findings emphasize the importance of both classical and modern learning strategies in the ongoing professional development of hospital pharmacists | None |
| 33 | Yun, 2019[^64^](#_ENREF_64), South Korea | Cross-sectional,  Observational | Examine the influence of informal learning and learning transfer in nurse’s clinical performance | Ham & Cho informal learning scale values  Lee’s learning transfer scale  Lee’s clinical performance scale | 200 Nurses working in tertiary hospitals | Nurses’ informal learning, learning transfer and clinical performance | Single cohort | Nurses working in tertiary hospitals | Survey | Descriptive and inferential statistics,  regression analysis | Clinical career and voluntary participation were related with clinical performance, informal learning and learning transfer | Small sample  (concerns on generalisability)  Self-reporting | None |
| 34 | Wang, 2024[^83^](#_ENREF_83), China | Qualitative | To examine the intrinsic and external motivation factors influencing self-directed learning among hospital pharmacists | Identification of key intrinsic and extrinsic motivational factors that influence hospital pharmacists' engagement in self-directed learning. | Sixteen hospital pharmacists | understanding the motivational factors (both intrinsic and extrinsic) that drive hospital pharmacists to engage in self-directed learning. | N/A | nine public hospitals located in urban areas of Henan Province | semi-structured interviews | Thematic analysis | Identified intrinsic motivations include curiosity, interest, achievement, self-efficacy, and personal growth. Extrinsic motivations include interpersonal motivations, work-related motivations, and career-related motivations. | complexity and hierarchical nature of learning motivation among hospital pharmacists and underscores the need for tailored strategies to enhance self-directed learning capabilities in this group. | None |
