# Supplementary material for: Self-directed learning in health professions: A mixed-methods systematic review of the literature
Source: PLoS One. 2025 May 2;20(5):e0320530. doi: 10.1371/journal.pone.0320530 (PMC12047769; doi:10.1371/journal.pone.0320530)
Supplement: S5 Appendix — (DOCX) [file pone.0320530.s005.docx]

**S5 Appendix:** List of Study Findings with Illustrations (qualitative results)

| Study: Allen 2024 | |
| --- | --- |
| Finding | Learning through hunting & gathering (C) |
| Illustration | ‘I think there's two kinds of learning that you need to do in keeping up, at least clinically right. I think there's foraging - so you're out there just walking around in the educational sphere and the CME, and the new lit- erature sphere and making sure that you're kind of aware of new developments that might only tangen- tially impact you ... But then there's really kind of hunting and gathering in a really directed way. So, you have a clinical question–knowing how to make use of search functions and make use of the library. And then finding things and then applying those through reading and stuff like that, or through going to conferences and stuff’. (Participant 7, Male, 28 years post training) |
| Finding | Train-on-a-track to treading water (C) |
| Illustration | ‘I think that since fellowship I have transitioned from being, I guess I'll use a metaphor, being on a train track and following the path and having the resources there and having everything kind of structured and built to going a lot more free flow and self-directed learning ... going from being on a train track to treading water in a pool. Yeah, there was no system in place’. (Participant 2, Female, 1 year post-training) |
| Study: Andersen 2022 | |
| Finding | Learning as a continuous process (U) |
| Illustration | Many GPs described a structured process of developing skills where they started scanning just one or a few anatomical areas to become familiar with these examinations before moving on to include more examinations in their portfolio. Other GPs, however, said that they started scanning exploratively within several anatomical areas before deciding on a portfolio suited for their everyday practice. Despite having an established practice with POCUS, all GPs, except one, planned continuously to expand their portfolio or update their competences by participating in future ultrasound courses. As one of the GPs described: We attended an ultrasound course before we bought the scanner. Then we participated in another course at the same time as we bought the scanner, and then again afterwards. I am not at all done with ultrasound courses (GP12). |
| Finding | Self-study and self-practice using Digital Aids (U) |
| Illustration | I have arranged one afternoon each week where I stay after work to do ultrasound examinations. There I have 20 minutes appointments. I tell the patients from the beginning that I have to practice and that I will treat them just as well as before. Typically, the session takes place using books and YouTube. (GP8) |
| Finding | Obstacles during the learning process: Financial (U) |
| Illustration | The extra time spent on an examination was described as money lost for the GP due to the lack of a fee for performing the examination. Some GPs explained that they had made organisational changes to make room for practising ultrasound examinations, for example by re-booking patients: … the main reason for re-booking the ultrasound examination a few days later is for me to be able to prepare for the scan. (GP17) |
| Finding | Learning with formal courses (C) |
| Illustration | Almost all GPs said that they had updated and expanded these previously obtained competences through at least one formalized ultrasound course. Such courses were described as a fundamental part of achieving ultrasound competences, and GPs with no prior experience from a hospital position had all gained their basic ultrasound competences by participating in a formalized ultrasound course. |
| Finding | Obstacles during learning process: workload and time constraints (U) |
| Illustration | The GPs described that their great workload and tight timeframe limited the time to practice their ultrasound skills during their normal working hours and they sometimes had to abandon an ultrasound examination: We are already racing against time. The consultation timeframe limits how much time you have to practice. (GP4). |
| Finding | Different learning strategies (C) |
| Illustration | All moved though the same gradual learning process when striving to achieve scanning proficiency, but their planning and approach to learning differed considerably. Some GPs described a deliberate approach and a clear strategy while others described themselves as ‘happy amateurs’ who had plunged into using POCUS with great enthusiasm, making random choices during the learning process. |
| Finding | Diverse learning Backgrounds (C) |
| Illustration | The GPs described different starting points. Especially younger GPs had used ultrasound in their residence training in hospitals, whereas others started as complete novices. |
| Study: Berg Jansson 2022 | |
| Finding | Learning enabled: valued competence (U) |
| Illustration | This theme illustrate how nursing as common ground provided opportunities for regular nurses and temporary agency nurses to share knowledge and support each other in daily work. Furthermore, how daily work as an temporary agency nurse were linked to feeling more appreciated and valued, and that one’s competence was more recognized and visible, than when working as a regular nurse. |
| Finding | Changing workplace as learning context (U) |
| Illustration | Only changing workplace was really competence development for me! I felt that I had stagnated a lot; it was the same people and maybe also a certain kind of operations. I almost never got the opportunity to participate in certain types of operations. (I10) |
| Finding | Difficulty in finding accessible learning (U) |
| Illustration | One temporary agency nurse said she had asked for courses, but that the temporary work agency had not been able to respond to that. ‘They have simply said that they do not have the courses. That they may arrange it in the future, or that if you can get a course at the client organization, we will pay for it’ (I5). Some temporary agency nurses, who had been offered shorter courses or lectures by their temporary work agencies, also described obstacles to participation in terms of distance and few options: There may be an email that ‘Now it’s a lecture’, and it is always in a city far away from here. Like, a single date and time. So, it’s a bit difficult for us who live here to take part. (I5) |
| Study: Bridges 2012 | |
| Finding | Contextual Influence on Self-Regulation: (C) |
| Illustration | The research by Kennedy et al. highlighted the significant role that cultural and contextual factors play in shaping how medical trainees engage in self-regulation. It specifically looks at the reluctance of trainees to seek help in clinical settings, showing how cultural norms and expectations, along with the supervisor-trainee relationship, critically influence learning behaviors. |
| Finding | Supporting Self-Regulation (N) |
| Illustration | There is an emerging focus on not just establishing environments that support self-regulation but also actively assisting learners in developing self-regulatory skills. This involves creating informed self-assessment opportunities, facilitating reflection, and guiding learners through directed self-guided activities. This approach helps manage the transition towards independent practice and learning. |
| Finding | Individual Factors Influencing Self-Regulation (C) |
| Illustration | Prior knowledge, beliefs, and emotions play critical roles in how learners manage and adapt their learning strategies. For instance, experiences from previous educational settings influence how trainees perceive and engage with new learning environments and expectations. |
| Finding | Learning Environments and Self-Regulated Learning (C) |
| Illustration | Various studies explore how educational settings can either promote or demand self-regulation. The concept of self-directed learning (SDL) is prominent, with environments designed to foster opportunities for self-regulation, like Problem-Based Learning (PBL) and lifelong learning modules. These settings not only support knowledge acquisition but also facilitate smoother transitions in learning phases, such as moving from PBL to clinical clerkships. |
| Finding | Self-Regulation in Practice (C) |
| Illustration | Research also focuses on ‘self-regulation in action,’ exploring how trainees set and pursue goals within novel learning environments, such as simulations. This research extends to clinical settings, examining how trainees seek feedback and use it to adjust their practices. |
| Study: Claret 2020 | |
| Finding | Role transition (U) |
| Illustration | Talos ((3rd year resident doctor)): […] but there is a point that you have to change your role, that you no longer do everything that nurses do, what midwives do […] And you start being more senior than a midwife, more doctor than a nurse […] and then you assume the role where you have to start saying what everyone else has to do! (18/01/2017) Collective interview with resident doctors. |
| Finding | Contextual Characterisation of Informality (U) |
| Illustration | When they are explaining who has been on duty, they always refer to the person by their surname, never by their first name. However, when they are explaining details referring to a specific situation, then they always address each other by their first name (05/10/2016). |
| Finding | Importance of equality in organisational learning (C) |
| Illustration | Artemis ((senior doctor)) comments that […] maybe they should be careful about a possible urethral injury […] Persephone ((senior doctor on a part-time contract)) tries to make a comment aloud three times, but all three times he is silenced by Poseidon ((senior veteran doctor)) and Artemis. Poseidon is especially managerial (perhaps because Athena ((head of department)) is not there), but Artemis does not pay much attention to his instructions. (02/12/2016) Fieldnotes. |
| Study: Clouder 2022 | |
| Finding | humility and an open- ness to one another as sources for learning (U) |
| Illustration | Not one person can know everything. We’ve got specialities and although we might have different training and experiences in other aspects, you can’t know everything. So I don’t know a lot about drug and alcohol [use] so I might turn to [colleague] or [another collea- gue] regarding that (3A). |
| Finding | Nature of in-service training (C) |
| Illustration | Team training was identified as important in achieving change. Where whole days or longer spells of formal training were required the logistics of maintaining a service were overcome by training half of the team while the other half kept the service going and then swapping to maintain cover. On occasions, or for specialist training, two staff members might be released for training on the basis that they would be more likely to effect change based on their joint learning. Whereas some regular training sessions were profession spe- cific, many were generic, suited to the whole team, and provided from within the team |
| Finding | Co-location triggered a natural tendency for information sharing (U) |
| Illustration | Because of us being in the same office, it’s easy to catch up with people on a more informal basis. So you’ll see somebody and say “I’ve been meaning to tell you this.”(4A) I’ll be on the phone and one of my health colleagues has overheard and said “sorry, do you mind if I chip in?” and it’s like “no, that’s great, it’s helpful.” (7A) we’re all in the same office . . . . it makes a massive difference because communication is so much better . . . and we all can discuss things a lot more easily and you just get to know people so you work a lot better alongside each other. (5C) |
| Finding | The shifting context of working in integrated teams (C) |
| Illustration | The first theme, providing a backdrop to learning in practice, emerged from recognition of the highly diverse nature of integrated care teams, how they were established, their intended scope and worries about sustainability. |
| Finding | Influence of leadership (U) |
| Illustration | We have clinical and managerial supervision on a one to one basis every 4 to 6 weeks. We’ve said these people are available [for clinical supervision],and they’ve [staff] said who they’re most comfortable with. We don’t discuss what happens in clinical supervision, we just need to know it’s on a regular basis and that if there’s any problems then either the supervisor or the member of staff discusses it. I suppose if we have got concerns then we’d discuss it more openly, but we just need to make sure it’s going on and we encourage them to access it as well. (3A) |
| Finding | Limited Funding (U) |
| Illustration | We’ve always had limited funding . . . . We’ve never really known that we’ve been safe. We’ve had contracts that have been time limited, 18 months when we first started, another 12 months, then we went up for tender so we didn’t know who was going to take over, if anybody. Now we’ve been taken over and it’s about getting that new service. I suppose that’s been most challenging, not having any money, not having any funding when members of staff have left, no funding to advertise those posts and get anybody else in (3A). |
| Finding | Working in the community was identified as very different to working in a hospital (U) |
| Illustration | The ward was okay but I felt that I couldn’t offer the support that I could there. I didn’t have time, the ward was busy. The OT did their part of the work but we never managed to spend time with the OT or physio because we were doing something else, whereas here you can spend time with the district nurses or the ambulance men if there’s a problem (4B). |
| Study: Cuyvers 2024 | |
| Finding | Affective and cognitive perceptions (U) |
| Illustration | Nurse 5: “… I am 60 and I really wanted to come to the hospital to work. I have spent most of my working career in elderly care. So, I come from care, and now I am very much into cure. Doing an intake for me is exciting and intriguing” Nurse 6: “… I find it inconvenient… because she is asking for an answer to her question… and normally… with infection rates, we answer rather generically, and this is very specific….” Head nurse 2: “well, for example, something new is introduced on the ward, and then everybody finds this a bit scary and is wondering how to deal with that… that is, we have a patient who will get parenteral nutrition, so intravenously. This is new on our ward and then the unrest on the ward can be felt. This patient and how to deal with this is often mentioned and questioned by the nurses. |
| Finding | Metacognitive awareness (U) |
| Illustration | Nurse 10: “I ask nurse Z because she is far more experienced than me”. |
| Finding | Awareness of learning needs (U) |
| Illustration | Nurse 3: “I work on a variable basis and because of that I sometimes lack rhythm. Consequently, I always have to pay very good attention to ensure all the paperwork is in order, and check whether people still need prescriptions when they are being dismissed. This is very important. I want to acquire this rhythm in the dismission procedure, I find that important”. |
| Finding | Self-Regulatory mechanisms (U) |
| Illustration | For example, one nurse challenges himself to improve in injecting drip. This challenge started as a joke, but certainly initiated subsequent learning: “… I restarted to practicing injecting drips, for fun, as a joke…and I aimed for poking the needle right, eight times in a row… yeah, it’s also just fun to challenge yourself…” |
| Finding | recognizing situations, tasks, and cases as affordances (U) |
| Illustration | Nurse 1: “… well, mainly about applying hypnosis… because we are just starting this as an approach, and we are trying to apply it… you need the right moment and the child that is open for it… so I was able to apply it one time on our ward, and for me each time is always a bit learning, because evidently it is different with each child”. Nurse 2: “… obviously this patient shows neurological failure, and I don’t think they are expecting it to come from the brain, but I think they are suspecting that it is an infectious disease, which of course is extremely instructive… Borrelia or Lyme’s disease, I don’t know much about that…”. |
| Finding | Questioning oneself, and one’s competencies (U) |
| Illustration | Head nurse 1: “…we had this situation recently where a patient had a heparin pump. This patient was going for surgery and the pump had to be stopped on a specific point of time before the operation. Then suddenly, I received a notification that something was wrong with this patient, and this is what happened: before going to surgery, this patient needed an extra examination, and the nurse simply detached the pump, removed the catheter, and transported the patient …just like that, far too long before the pump should have been stopped. So, I called this nurse with me, and I said, well I received this notification, this and this happened, why did you remove the heparin pump like you did? Do you know what this could have had as a consequence? Then the nurse said, yes, I know what the consequences can be. And I don’t know why I did this… I didn’t even think about it then, because it was busy on the ward, and I had a student nurse with me… And I know the protocol… I don’t know”. |
| Finding | Learning activities (U) |
| Illustration | Nurse 2: So I saw on the computer that radiculitus was mentioned, and then I failed to remember what that was, so I looked it up” Nurse 8: “well, the male patient of bed n◦ three was very ill last night. The attending physician came to see him many times and now he seems to be reviving a little bit… I find this… not interesting but instructional… you have to keep an extra eye on a patient like this, do extra checkups, and then give feedback to the physician about the patients’ status. I draw a lot from that” Nurse 9: “Yes, well, I know I should dive into this, but then it doesn’t happen. That’s it…I would need someone to sit next to me instruct me on how and what… but it is always like, here you go, that’s it… We help each other, but with this we actually gather a bit of what is needed here and a bit there… and that’s it. |
| Finding | Self-evaluation judgments (U) |
| Illustration | Nurse 13: “It may sound silly, but daily, when I am working… I always have, I mean, it comes spontaneously that I reflect… One can reflect on a shift or action… with me it comes spontaneously… when I end my shift… you see I come to work by bike, and then when I bike home, or when I change my clothes to go home, it doesn’t have to take long… then I think about how my day went, and what I possibly could do better in certain situations… So I do this automatically.” Nurse 14: “like with this ABCDE-method… physicians often refer to this, and that is still very hard for me… what exactly is A, what exactly is B, and so on… and so I always learn on this method…” Nurse 10: “… well today I learned to recognize when an anaphylactic reaction is taking place… I had never seen this so closeby, and react on it… so this is what this day brought to me…”. Head nurse 3: “… He is someone who waves it away… it happened but no harm is done, so …but as a head nurse, this is not the quality I want to provide on my ward… If this were me, and I would have made such a mistake, I would not sleep so to say, I would feel guilty towards this patient for having done something wrong… I find it so difficult when it is just waved away…”. |
| Finding | Prior experiences (U) |
| Illustration | “… nurse X is briefing nurse Y, who has not performed this procedure for many years. Nurse X says to nurse Y: ‘but I have never succeeded before’… in the meantime the daughter of the patient comes telling both nurses that her father is throwing up a brown flowing substance… nurse Y says that if nurse X instructs her, she will then try to place the pump…”. |
| Finding | Metacognitive monitoring (U) |
| Illustration | Nurse 11: “I notice in the moment that I am thinking a lot about how to perform this procedure, and that there is no routine yet.” Nurse 12: “I will notice when I get better…”. |
| Finding | Reflection (U) |
| Illustration | Nurse 9: “I will be honest with you, I am older, so for me that is just a thing. I find it difficult because there is so much to it when a patient is discharged from the hospital. You have to fax this, email that, ask the physician for that, a medical letter… all those things…”. Nurse 15: “I am almost 62…I was thinking lately, I used to work in pediatric daycare. I was very much into pediatric oncology, children being at home in the terminal phase… it is still so much in my heart… I find it… if I would have to do everything over, I would have… life goes as it goes, but I might have worked much more in pediatric oncology”. |
| Study: Fahlman 2013 | |
| Finding | Intentional Use of Mobile Devices (C) |
| Illustration | Informal learning using mobile device as a planned and intentional response to new and non-routine situations in their workplaces. two rural RNs downloaded Web resources at home and then accessed these resources offline in their workplaces. Most interviewees used their personal mobile devices and incurred data plan costs to engage in workplace informal learning. Some interviewees expressed trepidations related to employer’s perceptions of inappropriate mobile device use for personal communication. |
| Finding | Collaborative vs. Individual Learning Modes (N) |
| Illustration | While both collaborative and individual learning modes are used, there is a noted preference for individual strategies. This preference might stem from the nature of mobile device use, which is often more suited to personal, self-directed learning activities. |
| Finding | Individual and Collaborative Informal Strategies and Processes using Mobile Devices (C) |
| Illustration | only two interviewees acknowledged using collaborative modes (interacting with other people via e-mail and asking questions in a professional listserv or online community). In the online survey, interacting with other people via e-mail was frequently self reported. But when probed, the interviewees stated that they emailed via their mobile devices for communication purposes only (i.e., they did not use this process for informal learning). |
| Finding | Purposes of Informal Learning for using mobile devices (C) |
| Illustration | For accessing resources for evidence-based support and professional development, the actual frequency was more than expected while for maintaining competency the actual frequency was less than expected. |
| Finding | Positive perceptions of learning with mobile devices (U) |
| Illustration | Positive perceptions of engaging in informal learning strategies/process using mobile devices . The interviewees alluded to increased self-confidence and efficiencies in clinical practices attributed to the proactive use of mobile devices for accessing timely up-to-date information for informal learning. "These days with the younger generation, if you pull out your iPad or Palm and you come up with the information, you are seen as competent. You are seen as having the advanced knowledge. If you say “well just a minute, I have to go find my book” and you are flipping through the book then you are seen as old fashioned and that you aren't as current as you should be. I don't know that if it is necessarily a competence thing, but a lot of young people these days aren't going to sit there and want to watch you look through a book". |
| Finding | Healthcare Context (C) |
| Illustration | The context of the healthcare workplace may have influenced the selection of informal strategies/ processes implemented using the mobile devices |
| Finding | Reflective Practice (C) |
| Illustration | Through reflective practice, RNs problem solved using reflection-in-action during their experience, and self-evaluated after the encounter using reflection-on-action, to develop more effective ways of action for future practice |
| Study: Gathu, 2022 | |
| Finding | Flexibility in instruction (C) |
| Illustration | Creating conditions that foster reflection rather than imposing a predetermined reflective format on students has been argued to be the ideal structure to facilitate reflection. |
| Finding | Empowerment (N) |
| Illustration | A move to empower students to reflect, rather than imposing reflection on them, may be the fundamental change that would be needed to improve their acceptance by graduate students |
| Finding | Safe environment (N) |
| Illustration | Creating conditions that foster reflection rather than imposing a predetermined reflective format on students has been argued to be the ideal structure to facilitate reflection |
| Finding | Ambiguity and Misalignment (N) |
| Illustration | If students and faculty are unclear about the purpose and methods of reflection, it can hinder its effectiveness. |
| Finding | Inadequate Training and Confidence in Educators (N) |
| Illustration | Without sufficient training and confidence, teachers may not be able to facilitate or assess reflection adequately, leading to poor student engagement. |
| Finding | Coaching (N) |
| Illustration | Coaching helps learners identify their own learning needs and know what questions to ask to enhance the reflective process |
| Finding | Curriculum Alignment (N) |
| Illustration | Understanding the context of the reflective exercise and its alignment within the curriculum helps facilitate reflection for graduate students |
| Finding | Faculty Development (C) |
| Illustration | teachers of higher medical education need to receive some training on how to facilitate and measure reflection in postgraduate students |
| Finding | Time Constraints (N) |
| Illustration | Reflective practices require time, which is often limited |
| Finding | Accomodating diversity (C) |
| Illustration | allowing students to navi- gate their way around an approach to reflective practice may be crucial in accommodating their diverse learning styles. This theme has been mentioned by other authors |
| Finding | Creative writing (N) |
| Illustration | Appreciating differences in the methodology of reflective writing is of utmost importance |
| Finding | Incorporating multiple approaches for Assessment (N) |
| Illustration | evaluating the reflective process would help exclude subjectivity from this process and become beneficial in the formative assessment, facilitators must be aware that it may not be a ‘one-size-fits-all’ assessment process. |
| Finding | Role modeling (C) |
| Illustration | modeling of reflective practice by faculty and tutors is significant in facilitating students’ reflective capacity |
| Finding | Motivation of students (C) |
| Illustration | Motivation in the academic setting has been unequivocally found to facilitate reflection by several authors |
| Finding | Motivation (N) |
| Illustration | without intrinsic motivation, the activity can become a formality rather than a meaningful educational experience. |
| Finding | Use of Portfolios of Learning (C) |
| Illustration | adopting instructional approaches with some structure is superior compared to following some abstract principles of reflection |
| Finding | Sociocultural Context in Reflection (N) |
| Illustration | Innovating different ways to incorporate reflection, such as group reflection, may promote an interactional social dimension that could enhance its output. |
| Finding | Formative assessment (C) |
| Illustration | providing ongoing feedback on student reflective writing, rather than summative, can promote the improvement of the depth and quality of reflective practice over time. |
| Study: Ghyiasvandian 2015 | |
| Finding | Having a Subjective Care Plan (U) |
| Illustration | A work experience of several years in intensive care units has made my care practice purposeful. Primarily, my practice mainly focused on symptom management. However, currently, when I’m caring for high-risk patients, I try to search and find different ways for managing their problems. For instance, I had a patient with intracranial hemorrhage. Based on a subjective pattern, I understood that this patient has or will develop disorders such as respiratory distress, speech dysfunction, incontinence, confusion, immobility, and impaired nutrition. Accordingly, I focused my care on these disorders (P. 11). |
| Finding | Early Risk Identification (C) |
| Illustration | We had a patient in our unit who had experienced an injury to his seventh cervical vertebra (C7). At shift turnover, I said [to my colleagues] that you need to be ready because the edema will soon progress from C7 toward C1 and the medulla, suppressing patient’s respiratory center and causing apnea. I put a ventilator standby at patient’s bedside and explained the care plan for the colleagues. Predictions were accurate and when I returned to the unit the next morning, the patient had been attached to the ventilator (P. 15). |
| Finding | Role-Modeling (U) |
| Illustration | I had a knowledgeable and expert colleague who was eager to solve patients’ problems. I had a good feeling about his knowledge and practice. In my opinion, he was a real nurse— knowledgeable, skillful, and interested in managing patients’ problems. Well, I modeled him and gradually became interested in working in the same way as he did (P. 16). |
| Finding | Practicing as a Prerequisite to Expertise (U) |
| Illustration | One of my good experiences was learning cardiac ar hythmias. This was a complex skill which I learned through attending classes, practicing, and observing. However, this did not suffice. The important point was that I had to learn to immediately manage arrhythmias after diagnosing them. I learned how to manage arrhythmias again by practicing in real situations. It is like learning how to drive. Despite having a driver’s license, you will not become an experienced driver unless you drive in a city (P. 13). |
| Finding | Hypothesis Making (U) |
| Illustration | I always try to make some hypotheses based on the signs and the symptoms experienced by a patient. For instance, when a patient has tachypnea, I ask myself whether this patient is suffering from oxygen saturation disturbances, or she/he needs a chest X-ray assessment, or this sign is due to other problems such as pain (P.18). |
| Finding | Careful Observation (U) |
| Illustration | Despite having a wealth of academic information, many skills that I had learned in skill lab were not useful to me until observing experienced colleagues and physicians implementing nursing procedures such as dressing. Observation was very helpful to me. I reviewed the steps of the procedures while observing others doing] them (P. 12). |
| Finding | Learning Support (U) |
| Illustration | When I started practicing nursing in a dialysis unit, I felt frustrated despite having considerable experience of working in an emergency department. My colleagues supported me and boosted my morale. The unit head-nurse assigned me to an experienced nurse. That nurse, trained and supervised me and gave me constructive feedbacks. I remember that she said to me, ‘Patients receiving dialysis have distended veins and hence, when performing venipuncture, you should put the tip of the needle on the ventral rather than the lateral surface of the vein. Otherwise, the vein will be ruptured’ (P. 4). |
| Finding | Reflection on Performance (U) |
| Illustration | I have understood that each experience can be reflected on and such reflection can broaden that experience. Consequently, I usually question my experience to be obliged to think about it more carefully. For instance, I admitted a patient who had just undergone an appendectomy. He had a constant pain. I called the surgeon and administered analgesic for several times. However, the pain was persistent. Finally, the patient developed hypotension and was transferred again to the operating room. The diagnosis was pyloric rupture. I questioned myself to understand what type of care I had to provide to this patient (P. 12). |
| Finding | Desire for Questioning (C) |
| Illustration | Whenever I was allocated to a new ward, I understood that the staffs of that ward do their tasks based on a same routine. Perhaps, they had a convincing reason for such routine practice which was, ‘Routines are more convenient’. However, I refrained following routines and asked myself, ‘Can’t we do this task on any other ways?’ |
| Finding | Learning by doing (U) |
| Illustration | Another way for promoting my learning was learning by doing. An instance was the insertion of an NGT. I had read in books that we should first insert the tube into the fossa and once it reaches the oropharynx, we should bend patient’s head and ask him/her to swallow it. However, there was nothing in books about the nasopharynx and managing difficulties in inserting NGT into nasopharynx. I learned about that through using the trial and error method and practicing this skill (P. 10). |
| Finding | Learning From an Expert Colleague (U) |
| Illustration | My clinical learning was discussion-based and resulted from interacting with others. As I wanted to be a good nurse, I searched for skillful and knowledgeable nurses because they can create a halo of changes around themselves—like a fruitful tree whose branches, leaves, and fruits reach others. I’ve never missed such opportunities and have kept in touch with them (P. 9). |
| Finding | Anxiety Management (U) |
| Illustration | Experience is a wonderful thing. I have some levels of fear and anxiety while doing a new clinical task. However, alongside practicing and doing the task for several times, I learn to work more actively (P. 19). |
| Finding | Active Listening (C) |
| Illustration | I have learned to listen to patients because by using this strategy, I can both establish a therapeutic communication with them and get familiar with the subjective symptoms of their diseases (P. 1). |
| Finding | Maintaining a Caring Attitude (U) |
| Illustration | I’m very active at work. Excessive fatigue or external supervision does not matter. I know that I should provide care to patients. I search to find patients’ problems and overcome them accordingly. A practice like this gives me such good feelings that I become interested in following it more eagerly (P. 8). |
| Study: Hill 2010 | |
| Finding | Learning new information changing practice (U) |
| Illustration | Drl: Yes... In that the standard of care changes and I'm not always aware of it. (Interview, 2/11/10). Dr2: Sometimes. Sometimes it's just a matter of confirming what I felt. Sometimes I've gone, "Ooo I'm glad I looked that up." Sometimes I'll look things up because a patient will tell me something and I'll say, "No. That doesn't sound right." And then I'll basically look it up and be pleasantly surprised that they were right and I was wrong and I needed to look that up. (Interview, 12/2/09). |
| Finding | Strategies to answer clinical questions (U) |
| Illustration | Dr4: You know, if it's something that I know that I have a book on or I know that I have seen something and I just want to double check that information I would go to the book first and then otherwise I would do an internet or an UpToDate® search. And then the third strategy is, of course, the telephone is the best medical invention ever and you can always call and consult. That's a very, you know, yesterday I had a patient with a subarachnoid hemorrhage that was very small and no mass affect and I just got the head of neurosurgery at Harborview on the phone and linked him to the patient's CT and discussed it with him. (Interview, 2/3/10). |
| Finding | Learning into practice (reflection-in-action/reflection-on-action) (U) |
| Illustration | PA5: No. I like to... I mean, while my patient is going for x-rays and I'm drawing labs I like to go research the topic to see if there's anything out there, again, newer than what I know already. To make sure when I give them discharge instructions I give them the most current, best instructions I can... In the emergency room yeah, because if I have to get a chest x-ray or an EKG I have 10 or 15 minutes to go sit in a room by myself or go get on a computer and just look and see what's out there new. (Interview, 2/25/10). |
| Finding | Learning Style (U) |
| Illustration | Dr8:1 personally like all three. (But) you know, what's the question I need to have answered? The reading type things is, number one is that I'm an old timer, and so I feel very comfortable reading and looking at books. Generally you find that the people who are 60 and older are not necessarily adept at electronic stuff. It's uncomfortable. The under 60s and up to about age 40 they're comfortable with it, but it's not second nature. Under 40 they grew up with and especially the people under 20 they've grown up with personal computers and so people seek out information according to what they're comfortable with. Most of us older folks like printed stuff. (Interview, 1/9/10). |
| Finding | Use of virtual information for CME purposes (U) |
| Illustration | Dr6: As far as electronic information, we use our CME money.. .to buy subscriptions to various information; electronic information. People use different things. I know three or four of us use UpToDate®. A couple of the others use MDConsult®, I believe...We're basically allocated funds per year for CME. So, it's just up to the provider of what they want to do with those funds. (Interview, 11/30/09). |
| Finding | Access to virtual sources for SDL (U) |
| Illustration | We have computer stations and various people have individual access to programs that they have subscribed to, but as the hospital per se it does not have an official library. All of us have our own libraries and so most of the current books, I mean, a lot of the books relate to public health stuff that was back in the 80s and stuff and I've personally been throwing a bunch of them away. The most current library is mostly what I have supplied.. .Folks here are not real motivated into getting into lots of.. .they're just not into the reference books. The older doctors have a tendency to look at reference books. The younger ones have a tendency to look at online access stuff. (Interview, 1/9/10). |
| Finding | Online resources are the most used information resource (U) |
| Illustration | Dr7:1 probably use it every day (UpToDate®). I used to use a PDA a lot. I still have one, but I don't use it as much. Mainly because I'm using the computer more. I have a bunch of reference books in the doctor's lounge in the hospital and I have some of my own in the office that I do use pretty regularly. (Interview, 2/16/10). |
| Study: Papanagnou 2022 | |
| Finding | Impact on Clinical Practice (C) |
| Illustration | The uncertainty surrounding clinical practice forced healthcare teams to constantly adapt and innovate. There was no pre-defined playbook, and teams had to learn in real-time, often making high-stakes decisions without a clear right answer. |
| Finding | Chaos to Complex Transition (C) |
| Illustration | Initially, clinical teams found themselves in chaotic situations without clear directives or precedents. The Cynefin framework helped them transition to a complex phase where they could begin to understand the links between actions and outcomes, albeit not straightforwardly. |
| Finding | Learning Models (C) |
| Illustration | It also stresses the importance of learning models that accommodate the non-linear, complex nature of real-world clinical practice, encouraging a shift from traditional learning paradigms to those that foster innovation, critical thinking, and adaptability |
| Finding | Uncertainty (C) |
| Illustration | The article suggests that health professions education should prepare practitioners to handle uncertainty more effectively. It calls for integrating sensemaking frameworks like Cynefin into educational curricula to help future healthcare providers navigate complex and ambiguous situations more adeptly. |
| Finding | Networks and Collaboration (C) |
| Illustration | Effective management of the pandemic required reliance on networks of knowledge and collaborative efforts within and across teams. The fluidity in team dynamics facilitated learning and adaptation to the shifting clinical landscape. |
| Finding | Informal and Incidental Learning (C) |
| Illustration | The pandemic underscored the importance of IIL, where learning happens in the flow of work without formal structures. Teams had to be creative and responsive, integrating new knowledge and adapting existing practices in response to rapidly changing conditions. |
| Finding | Volatility, Uncertainty, Complexity, and Ambiguity (C) |
| Illustration | These elements defined the healthcare environment during the pandemic, highlighting challenges like PPE shortages and the need for rapid adaptation and learning by healthcare teams. |
| Finding | Sensemaking as Reconfiguration (C) |
| Illustration | Instead of just acquiring new knowledge, teams reconfigured existing knowledge to make sense of and respond to unfolding events |
| Study: Yao 2023 | |
| Finding | Continuing education (U) |
| Illustration | When I came to Shanghai, I studied for two months, eight weeks. In fact, I think I progressed greatly during those eight weeks… I think those eight weeks were eight weeks of my life where I made a lot of progress. (P2) |
| Finding | Social platforms (U) |
| Illustration | I feel like I have learned a lot through those official accounts… For example, an official account about critical care medicine…. You can keep updated with a lot of cutting-edge knowledge. (P3) |
| Finding | Professional medical databases (U) |
| Illustration | UpToDate… the mobile version of Micromedex, the memo and the collection function of WeChat. (P4) |
| Finding | Professional books and literature (U) |
| Illustration | So, for me, er, I still trust professional literature more and feel they are more authoritative and reliable. (P4) |
| Finding | Online courses (U) |
| Illustration | There are some good courses, and they are all online. I can take part in any course that I want to… When I know what my fellows are doing, I probably know the gap between us and whether I am doing a good job. (P2) |
| Finding | Setting goals (U) |
| Illustration | There should definitely be a goal… Then, there may be various adjustments during this process. (G2) |
| Finding | Implementing plans (U) |
| Illustration | If we encounter some unexpected events, such as the schedule being disrupted, I might make up for it afterward, and sometimes I will work overtime to get this done. (G2) |
| Finding | Summarising and reflection (U) |
| Illustration | Sharing with others by summarizing is a mutually beneficial relationship. In the process of sharing with others and reflecting, it is a process where you urge yourself to study. (P4) |
| Finding | Integration of theory and practice (U) |
| Illustration | For pharmacists, if we have a question, we will go to study. After that, if you don’t put it into practice and you don’t summarize it by yourself, you may think that you have learned 100%. However, in the end, only you have learned only 10%. (G1) |
| Finding | Memorisation and imagery (U) |
| Illustration | The fact is that you can’t memorize all these paragraphs at one time, but you can memorize paragraph by paragraph and understand paragraph by paragraph. We like to draw lines and mark the key points. I think these can be summarized as what I just called cognitive strategies. (P2) |
| Finding | Professional forums (U) |
| Illustration | Like Dingxiangyuan, those are the forums that doctors often use. We also use Medlive as well as their application or website. (P2) |
| Finding | Making plans (U) |
| Illustration | For example, I plan to finish it in two weeks… Then, you need to write down all the things you plan to do each day and divide them into different parts. (P2) |
| Study: Wang 2024 | |
| Finding | Occasional stimulus (U) |
| Illustration | All of a sudden, there was something that hit me so hard, and I was impressed that I felt that it should be one of the beliefs I have learned so far. From that moment on, I told myself I can’t go on like this and start learning. (P2) |
| Finding | Task relevance (U) |
| Illustration | Let’s say there’s a new drug. I think this is helpful for patients, and it also makes a lot of improvement of myself. (P11) |
| Finding | Promotion opportunities (U) |
| Illustration | I hope to be promoted to a higher position before 40, so I need to set a learning plan.(P9) |
| Finding | Peer influence (U) |
| Illustration | She has been learning, keep learning. She is now growing particularly fast. Now she is accepted by all as a model. You know, I got a strong feeling of challenge and pressure from her. And I began to ask myself: why couldn’t I become her? why couldn’t I exceed her? (P12) |
| Finding | Career pressure (U) |
| Illustration | My job here is usually about checking prescriptions and dispensing medications. I realize that this job is very easy to alternative, so I have been in and constantly looking for different ways to improve myself. (P2) |
| Finding | Personal growth (U) |
| Illustration | I just believe in myself and my ability to get better grades. Then, er…I'm quite conscious of my studies and I learn well (P1) |
| Finding | Professional development (U) |
| Illustration | I am also really not very clear about the future. However, I think our job has a bright future in foreign countries, or you have the chance to carry out work at least. So, just do it. (P11) |
| Finding | Patients' influence (U) |
| Illustration | Even if when I work at the pharmacy dispenses place, I think patients will ask some questions about drugs. We have to be professional at least, so I often turn over some pharmacology books. (P9) |
| Finding | Learning atmosphere (U) |
| Illustration | Everyone around you is constantly learning and improving. You can also facilitate learning by working in that environment. (P8) |
| Finding | Industry trends (U) |
| Illustration | The government has released a lot of documents and policies on providing professional pharmacy services to patients, which makes me feel that pharmacists are very important, so I’m going to study (P13) |
| Finding | Self-efficacy (U) |
| Illustration | I feel self-improvement or self-investment is very important, so I insist on buying (learning materials)”(P2) |
| Finding | Interest (U) |
| Illustration | I just feel myself slowly, err(modal particle), will be more like my career. I think it is the power of interest and then I am more willing to carry out SDL. (P4) |
| Finding | Achievement (U) |
| Illustration | The most important is a sense of recognition in clinical. The sense of value, will continue to promote my learning. (P8) |
| Finding | Avoidance of negative consequences (U) |
| Illustration | Our teacher said that if you do not write you cannot graduate. Then, okay, I wrote it, and finished it within ten days. This was also a driving force. (P7) |
| Finding | Adaptation to change (U) |
| Illustration | When I moved to a new department, which is a completely new area, then I was actually utterly confused, and began to learn. (P5) |
| Finding | Social support (U) |
| Illustration | My family, I care about them, and they have high expectations of me. I will work hard, keep learning and keep improving. (P16) |
