# Supplementary figures and images for: Self-directed learning in health professions: A mixed-methods systematic review of the literature
Source: PLoS One. 2025 May 2;20(5):e0320530. doi: 10.1371/journal.pone.0320530 (PMC12047769; doi:10.1371/journal.pone.0320530)

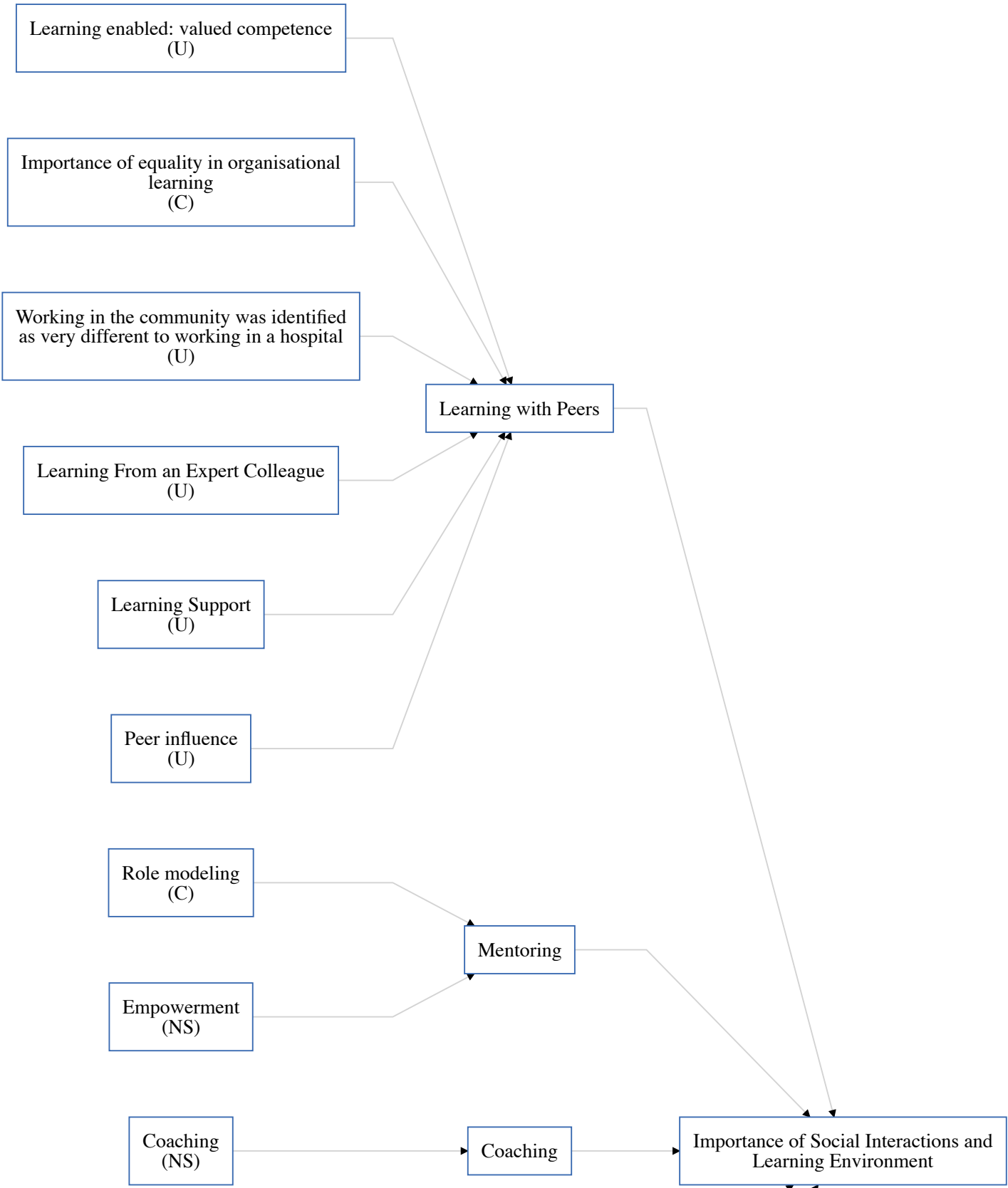



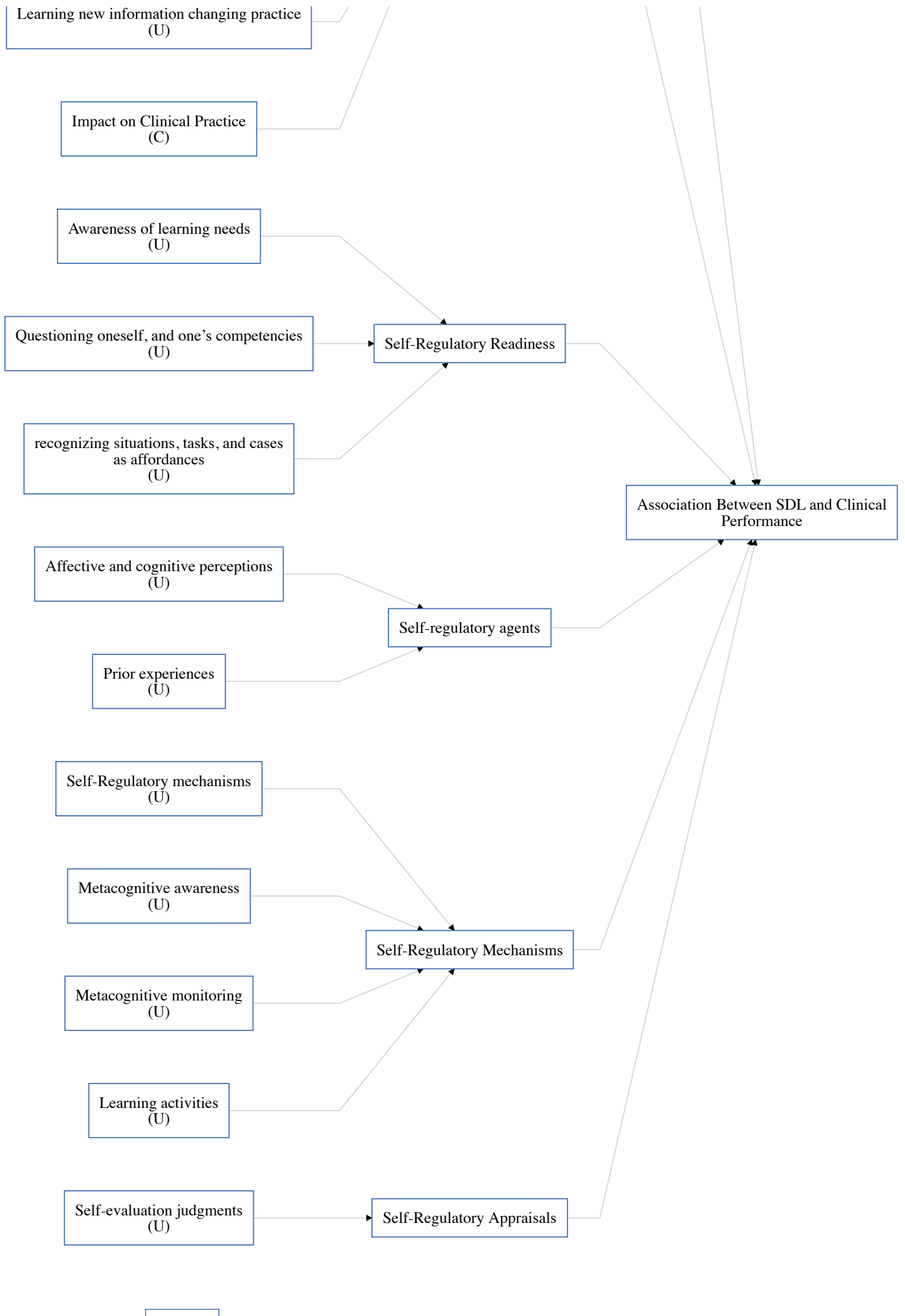

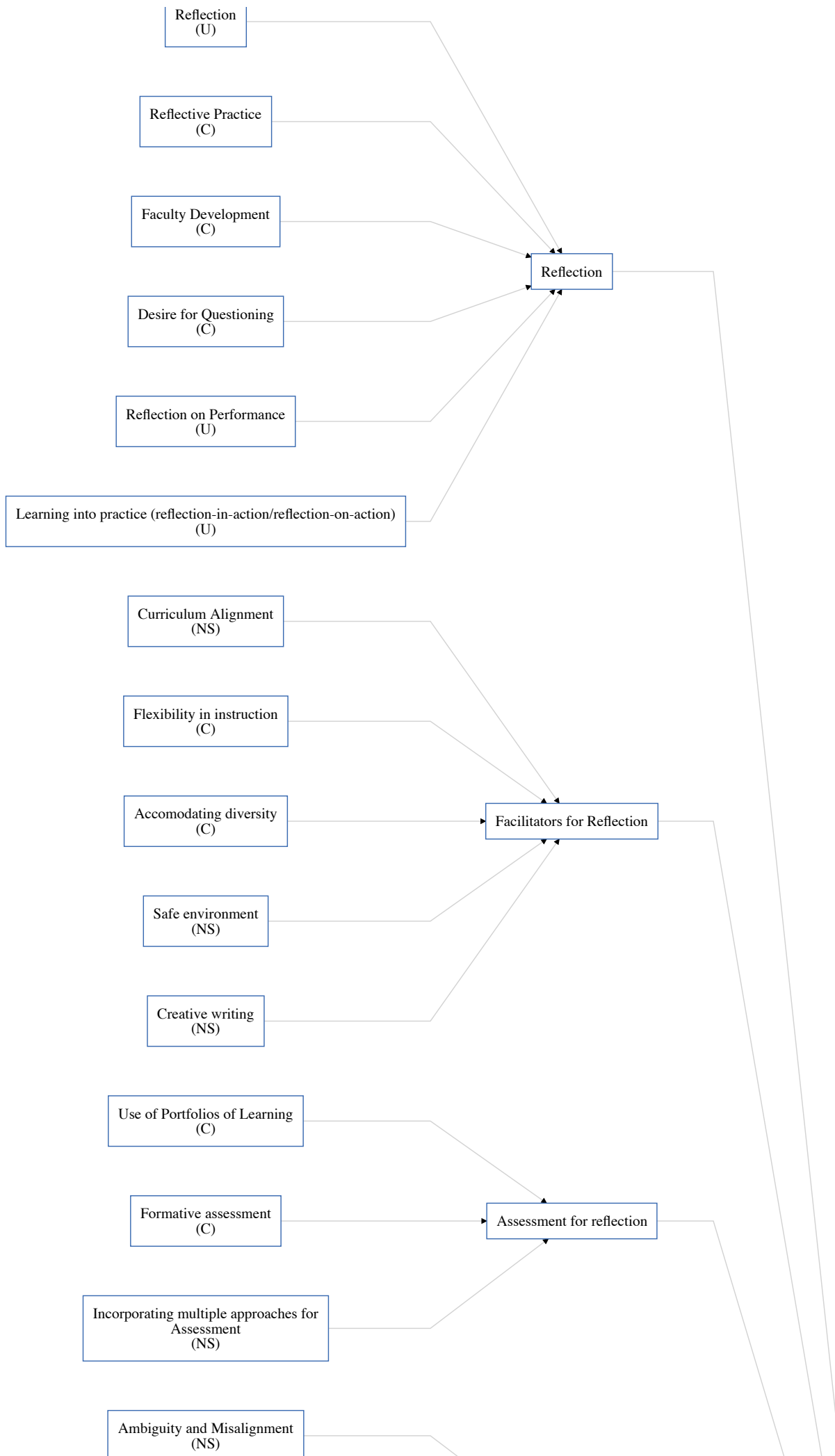

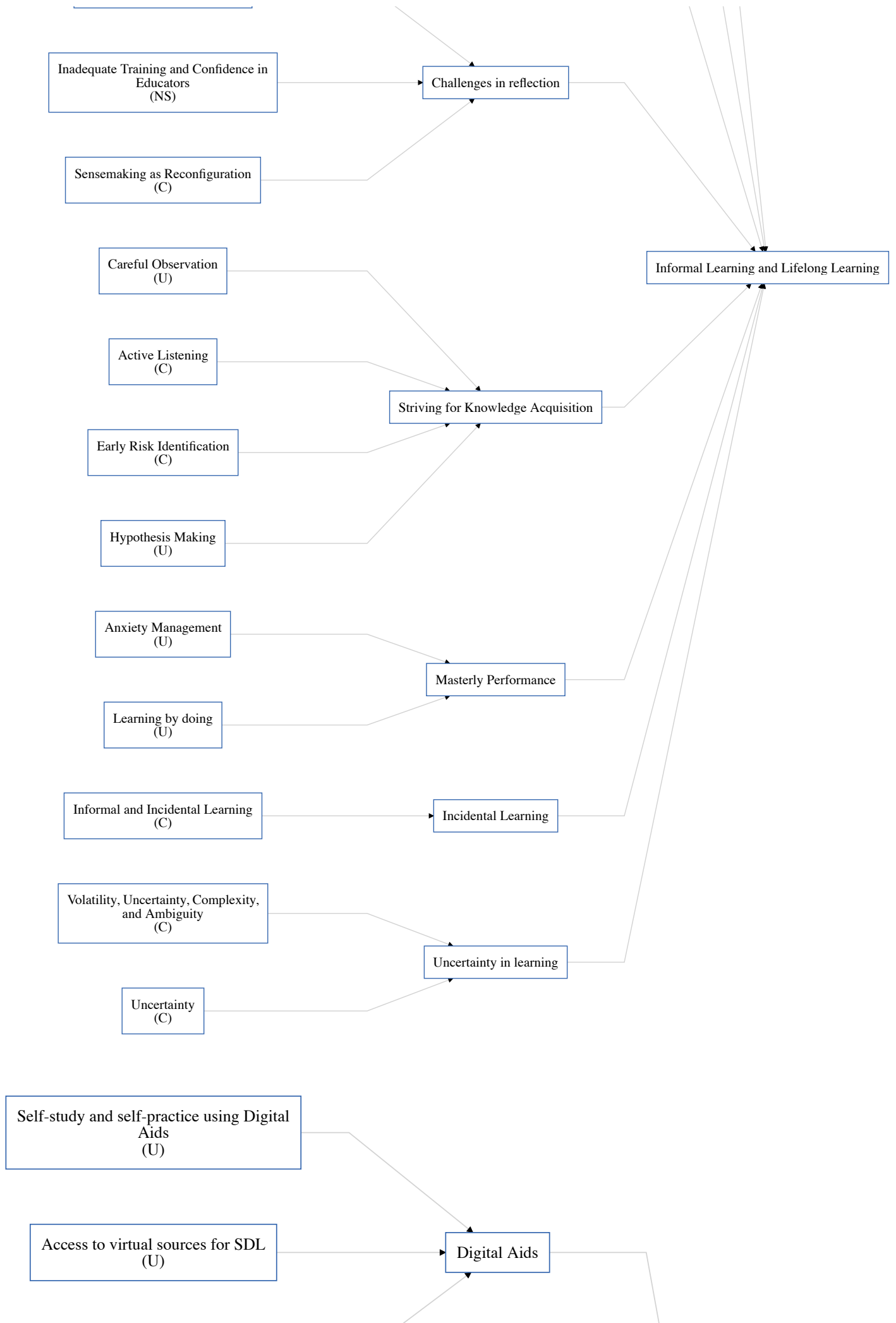

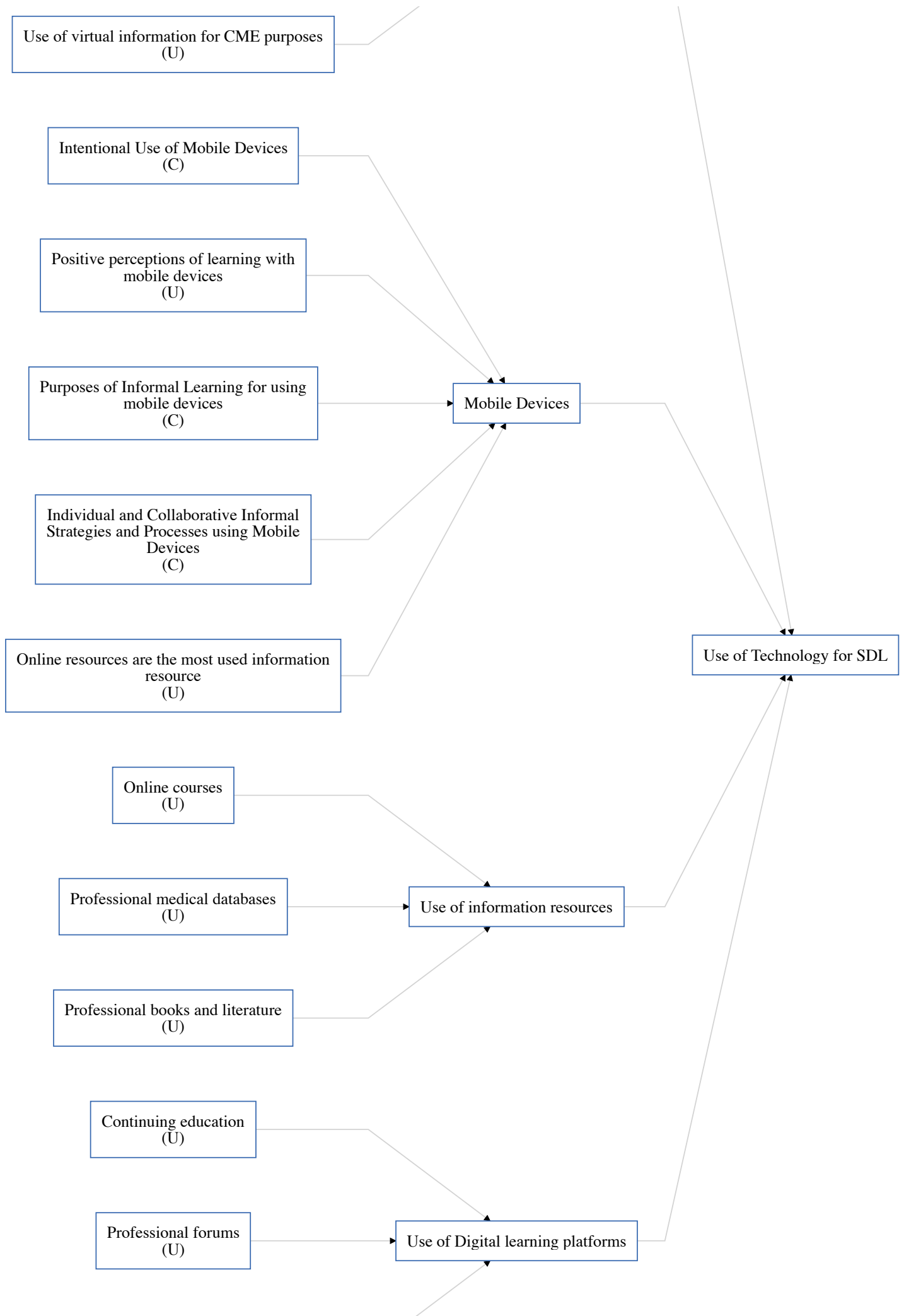

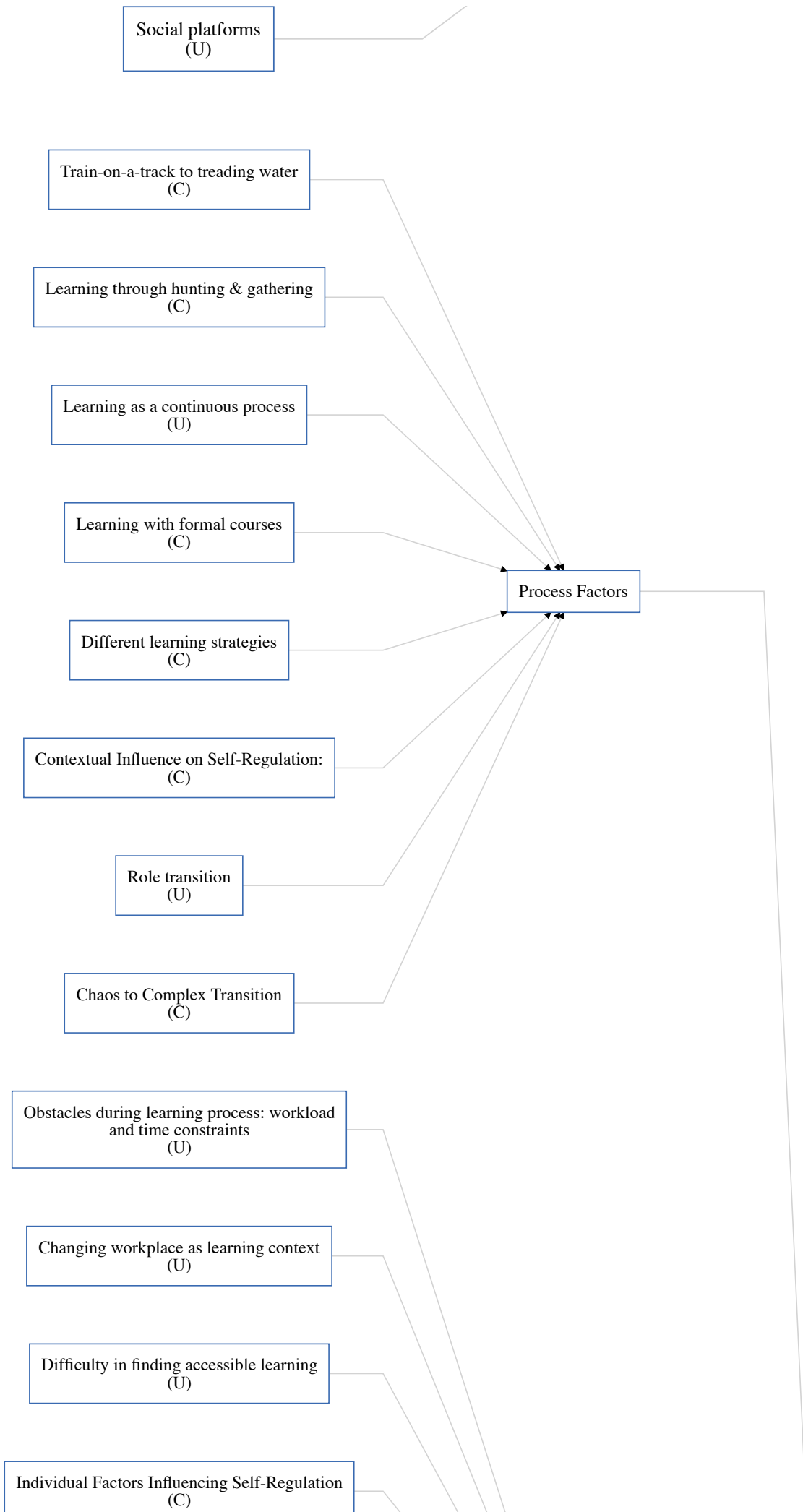



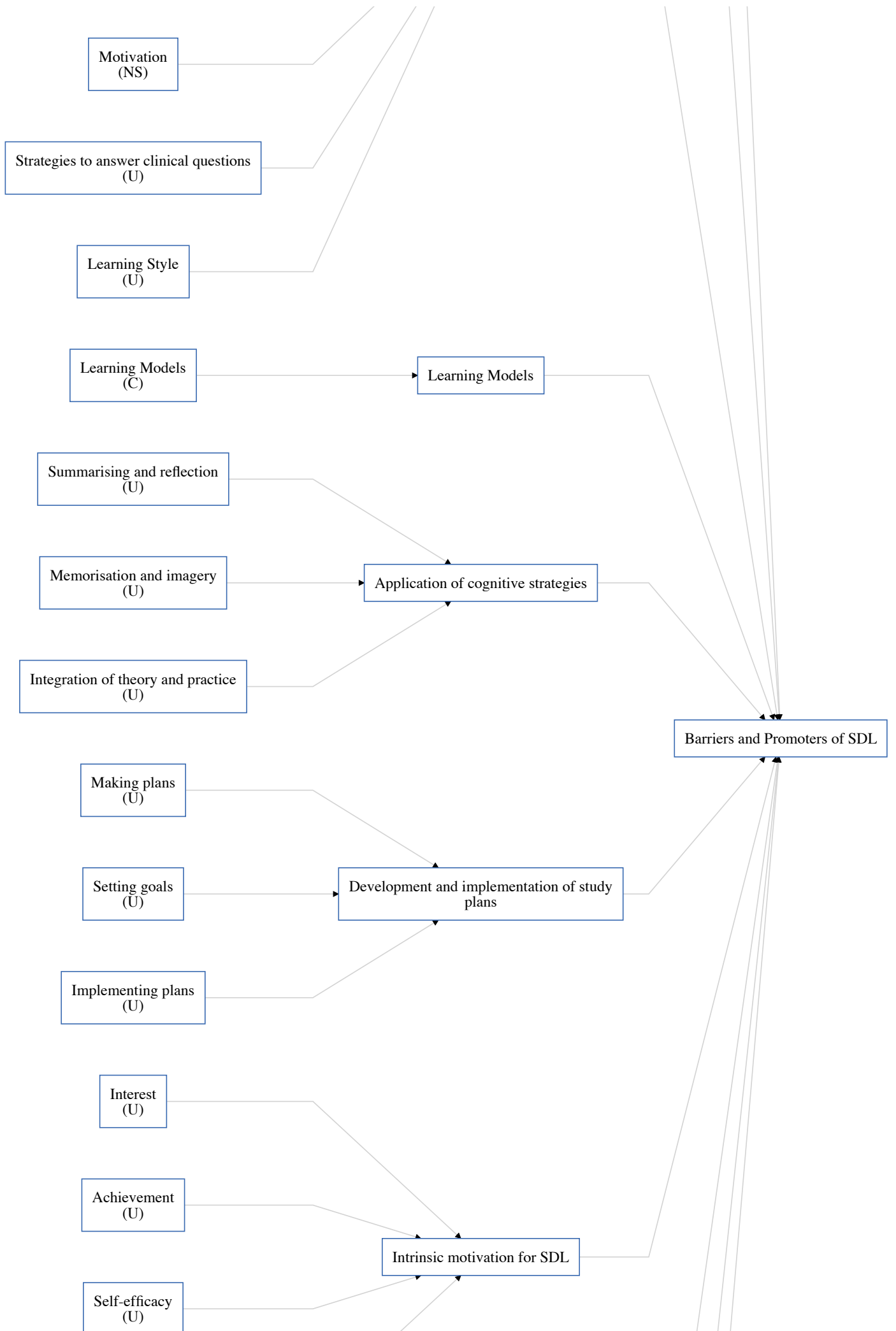

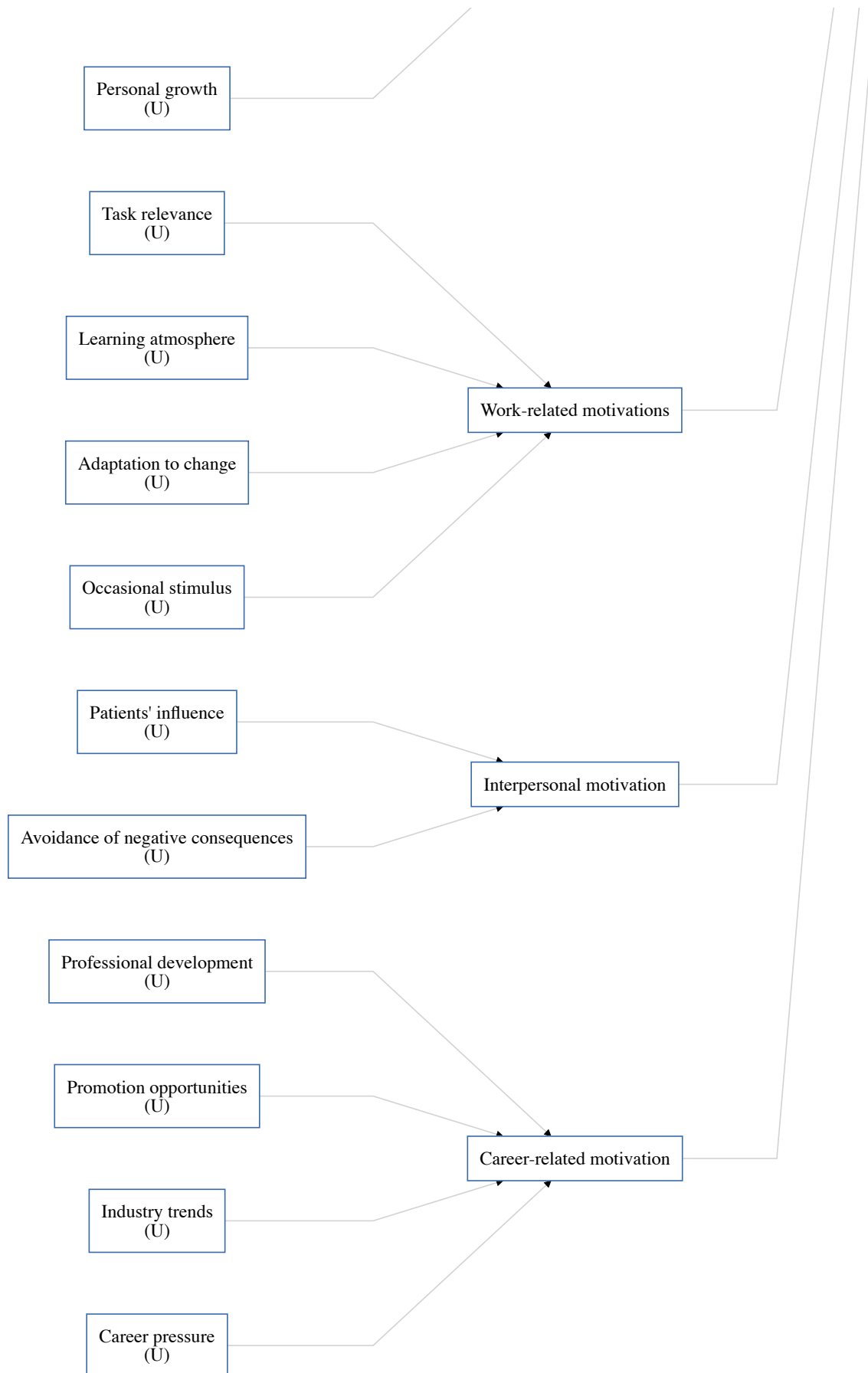

Supplement: S6 Appendix — (PDF) [file pone.0320530.s006.pdf]
