# Supplementary material for: Self-directed learning in health professions: A mixed-methods systematic review of the literature
Source: PLoS One. 2025 May 2;20(5):e0320530. doi: 10.1371/journal.pone.0320530 (PMC12047769; doi:10.1371/journal.pone.0320530)
Supplement: S1 File — (DOCX) [file pone.0320530.s008.docx]

| **Nr.** | **Reason for Exclusion** | **Citation** | |
| --- | --- | --- | --- |
| 1 | Rejected by TIAB Screening | Continuing Education in the Professions, Number 1. Current Information Sources. Syracuse Univ., NY. ERIC Clearinghouse on Adult Education.; 1967. | |
| 2 | Rejected by TIAB Screening | Proceedings Book of the National Conference on Continuing Education for Nurses (1st, Williamsburg, Virginia, November 10-14, 1969). Virginia Commonwealth Univ., Richmond.; 1969. | |
| 3 | Rejected by TIAB Screening | Computer-based Instruction of Basic Nursing Utilizing Inquiry Approach: Final Report. Reports - Research. Illinois Univ., Urbana. Computer-Based Education Research Lab.; 1970. | |
| 4 | Rejected by TIAB Screening | Viewpoints of the Discussion Group: Motivation and Relevance. Journal of Medical Education. 1970;45(11):109-17. | |
| 5 | Rejected by TIAB Screening | Promising Practices in Small High Schools. A Report of 15 Northwest Projects. Northwest Regional Educational Lab., Portland, OR.; 1970. | |
| 6 | Rejected by TIAB Screening | A Refresher Course for Registered Nurses: A Guide for Instructors and Students. Guides - General. Health Resources Administration (DHEW/PHS), Bethesda, MD. Div. of Nursing.; 1974. | |
| 7 | Rejected by TIAB Screening | Higher Education in Ohio Master Plan: 1976. Reports - Descriptive. Ohio Board of Regents, Columbus.; 1976. | |
| 8 | Rejected by TIAB Screening | Development for Independent and Group Learning in a Core Curriculum. Final Project Report. Reports - Descriptive. Saint Anselm's Coll., Manchester, NH. School of Nursing.; 1977. | |
| 9 | Rejected by TIAB Screening | Library Research and Computer Search Module, Revised Edition. National College of Education: Field Experience Programs. Guides - Classroom - Learner Reference Materials - Bibliographies. National Coll. of Education, Evanston, IL.; 1982. | |
| 10 | Rejected by TIAB Screening | Proceedings of the Annual Adult Education Research Conference (24th, Montreal, Quebec, April 8-10, 1983). Collected Works - Proceedings Reports - Research. Montreal Univ. (Quebec)., Concordia Univ., Montreal (Quebec). 1983. | |
| 11 | Rejected by TIAB Screening | Emerging Perspectives on the General Professional Education of the Physician: Problems, Priorities, and Prospects. Reported to the Panel by the Medical Schools, Colleges, and Academic Societies. Opinion Papers Reports - Research. Association of American Medical Colleges, Washington, DC.; 1983. | |
| 12 | Rejected by TIAB Screening | Availability and Utilization of Self-Learning Materials in Continuing Education. Report on a Study. Reports - General. World Health Organization, Copenhagen (Denmark). Regional Office for Europe.; 1983. | |
| 13 | Rejected by TIAB Screening | Conclusions and Recommendations of Panel on Medical-Student Education. Chronicle of Higher Education. 1984;29(5):15-20. | |
| 14 | Rejected by TIAB Screening | Physicians for the Twenty-First Century. The GPEP Report: Report of the Panel on the General Professional Education of the Physician and College Preparation for Medicine. Reports - Descriptive. Association of American Medical Colleges, Washington, DC.; 1984. | |
| 15 | Rejected by TIAB Screening | Canadian Association for the Study of Adult Education. Proceedings of the Annual Conference (4th, Montreal, Quebec, Canada, May 28-30, 1985). Collected Works - Proceedings. Canadian Association for the Study of Adult Education, Guelph (Ontario). 1985. | |
| 16 | Rejected by TIAB Screening | Adult Education and the Elderly. Case Studies from Latin America, Africa, Asia and Europe. Background Materials. The Literacy Debate. General Discussion. Literacy and Women. Literacy and Health-Programmes. Adult Education and Development. Number 24. Adult Education and Development n24 Mar. 1985. | |
| 17 | Rejected by TIAB Screening | Commentary on the Report of the Panel on the General Professional Education of the Physician and College Preparation for Medicine. Journal of Medical Education. 1986;61(4):345-52. | |
| 18 | Rejected by TIAB Screening | Midwest Research-to-Practice Conference in Adult, Continuing and Community Education (8th, St. Louis, Missouri, October 12-13, 1989). Collected Works - Proceedings Reports - Research Reports - Descriptive. Missouri Univ., St. Louis. Univ. Extension - East Central Region.; 1989. | |
| 19 | Rejected by TIAB Screening | Helping Learners at a Distance. Annual Conference on Teaching at a Distance (5th, Madison, Wisconsin, August 8-10, 1989). Collected Works - Proceedings Reports - Research Reports - Descriptive. Wisconsin Univ., Madison. School of Education.; 1989. | |
| 20 | Rejected by TIAB Screening | Reflecting on Practice in Adult Higher Education. National Conference on Alternative and External Degree Programs for Adults (11th, Mobile, Alabama, October 10-12, 1991). Collected Works - Proceedings Reports - Descriptive. Alliance, an Association for Alternative Degree Programs., American Council on Education, Washington, DC.; 1991. | |
| 21 | Rejected by TIAB Screening | L.P.N. Update Program. A Program of Self-Study for Nurses. Guides - Classroom - Teacher. Western Wisconsin Technical Coll., La Crosse.; 1991. | |
| 22 | Rejected by TIAB Screening | Professionals' Ways of Knowing and the Implications for CPE. Proceedings, Commission for Continuing Professional Education of the AAACE Pre-Conference (Montreal, Quebec, Canada, October 13-15, 1991). Collected Works - Proceedings. American Association for Adult and Continuing Education, Washington, DC. Commission of Professors of Adult Education.; 1991. | |
| 23 | Rejected by TIAB Screening | Integrated Occupational Program: Information Manual for Administrators, Counsellors, and Teachers. Curriculum Standards. Reports - Descriptive Guides - Non-Classroom. Alberta Dept. of Education, Edmonton. Curriculum Standards Branch.; 1994. | |
| 24 | Rejected by TIAB Screening | Nurse Aide. Occupational Competency Analysis Profile. Guides - Classroom - Teacher. Ohio State Univ., Columbus. Vocational Instructional Materials Lab.; 1995. | |
| 25 | Rejected by TIAB Screening | Students in Transition: Critical Mileposts in the Collegiate Journey. Inaugural National Conference Proceedings (1st, Dallas, Texas, November 9-11, 1995). Collected Works - Proceedings. Texas Higher Education Coordinating Board, Austin., South Carolina Univ., Columbia. National Resource Center for the Freshman Year Experience and Students in Transition.; 1995. | |
| 26 | Rejected by TIAB Screening | Project WORKSMART. A Final Report. Reports - Descriptive Reports - Evaluative. NashvilleREAD, Nashville, TN.; 1998. | |
| 27 | Rejected by TIAB Screening | Bringing down the Barriers. First WEA Submission to "The Learning Age" Consultation. Opinion Papers. Workers Educational Association, London (England). 1998. | |
| 28 | Rejected by TIAB Screening | Distance Learning '99. Proceedings of the Annual Conference on Distance Teaching and Learning (15th, Madison, Wisconsin, August 4-6, 1999). Collected Works - Proceedings. Wisconsin Univ. System, Madison.; 1999. | |
| 29 | Rejected by TIAB Screening | HRD and Employee Outcomes. Symposium 42. [Concurrent Symposium Session at AHRD Annual Conference, 2000.]. Collected Works - General Speeches/Meeting Papers. 2000. | |
| 30 | Rejected by TIAB Screening | Sketches of Innovators in Education: A Collection of Articles on Teaching with Technology. Fourth Edition. Collected Works - General Reports - Descriptive. Indiana State Univ., Terre Haute.; 2002. | |
| 31 | Rejected by TIAB Screening | National Data on Participation in VET in Schools Programs & School-Based New Apprenticeships for the 2004 School Year. Ministerial Council on Education, Employment, Training and Youth Affairs. 2005. | |
| 32 | Rejected by TIAB Screening | Conflicts of interest in medical education. Conflict of interest in medical research, education, and practice. Washington, DC: National Academies Press; US; 2009. p. 122-65. | |
| 33 | Rejected by TIAB Screening | Group 11a: School-Based Psychologists. IMPACT: The District of Columbia Public Schools Effectiveness Assessment System for School-Based Personnel, 2012-2013. District of Columbia Public Schools. 2012. | |
| 34 | Rejected by TIAB Screening | How People Learn II: Learners, Contexts, and Cultures. National Academies Press. 2018. | |
| 35 | Rejected by TIAB Screening | Abbott PA, Brooker R, Hu W, Hampton S, Reath J. "I Just Had No Idea What It Was Like to Be in Prison and What Might Be Helpful": Educator and Learner Views on Clinical Placements in Correctional Health. Teaching and learning in medicine. 2020:1-12. | |
| 36 | Rejected by TIAB Screening | Abbott S, Renfrew MJ, McFadden A. 'Informal' learning to support breastfeeding: local problems and opportunities. Maternal & Child Nutrition. 2006;2(4):232-8. | |
| 37 | Rejected by TIAB Screening | Abdel Aziz F. Education and the professionals. Some aspects of innovation in medical education adopted by Ahfad University for Women. Ahfad Journal. 1994;11(2):33-40. | |
| 38 | Rejected by TIAB Screening | Abdel Meguid EM, Khalil MK. Measuring medical students' motivation to learning anatomy by cadaveric dissection. Anatomical sciences education. 2017;10(4):363-71. | |
| 39 | Rejected by TIAB Screening | Abdellatif H, Dechow PC, Jones DL. Principles of evidence-based dental practice (EBDP). Texas Dental Journal. 2011;128(2):155-64. | |
| 40 | Rejected by TIAB Screening | Abdulla D. Attitudes of college students enrolled in 2-year health care programs towards online learning. Computers & Education. 2012;59(4):1215-23. | |
| 41 | Rejected by TIAB Screening | Abernethy A, Abrahams E, Barker A, Buetow K, Burkholder R, Dalton WS, et al. Turning the tide against cancer through sustained medical innovation: The pathway to progress. Clinical Cancer Research. 2014;20(5):1081-6. | |
| 42 | Not Target Group | Abraham RR, Fisher M, Kamath A, Izzati TA, Nabila S, Atikah NN. Exploring First-Year Undergraduate Medical Students' Self-Directed Learning Readiness to Physiology. Advances in Physiology Education. 2011;35(4):393-5. | |
| 43 | Rejected by TIAB Screening | Abraham S, McBride A. Contraception: A new practical learning package. Medical Education. 2000;34(8):679-81. | |
| 44 | Rejected by TIAB Screening | Abrahamson S. Harvard Medical School Tries a Problem-Based Curriculum; Its Effect on the Education of Physicians Could Be Profound. Chronicle of Higher Education. 1987;34(8):B1-2. | |
| 45 | Rejected by TIAB Screening | Abu-Moghli FA, Khalaf I, Halabi J, Wardam L. Jordanian baccalaureate nursing students' perception of their learning styles. International Nursing Review. 2005;52(1):39-45. | |
| 46 | Rejected by TIAB Screening | Abuelo A, Castillo C, May SA. Usefulness of Crossword Puzzles in Helping First-Year BVSc Students Learn Veterinary Terminology. Journal of veterinary medical education. 2016;43(3):255-62. | |
| 47 | Rejected by TIAB Screening | Ackman ML, Romanick M. Developing preceptors through virtual communities and networks: Experiences from a pilot project. Canadian Journal of Hospital Pharmacy. 2011;64(6):405-11. | |
| 48 | Rejected by TIAB Screening | Adam KA. Hyposkillia: A sign of sagging medical profession-A pediatric perspective. Sudanese Journal of Paediatrics. 2014;14(1):11-20. | |
| 49 | Rejected by TIAB Screening | Adams J, Altshuler L, Fox J, Kurland S, Hanley K, Gillespie C, et al. Your patient's sugar is too high!: Resident physician interprofessional phone communication skills. Journal of General Internal Medicine. 2014;29:S261. | |
| 50 | Rejected by TIAB Screening | Adams J, Kulasekaran V, Guiton G, Oatis K, Gong J. The denver health longitudinal integrated clerkship (DH-LIC): An innovation fostering patient-centered advocates for social justice and health equity. Journal of General Internal Medicine. 2019;34 (2 Supplement):S852. | |
| 51 | Rejected by TIAB Screening | Adams S. A Self Study Tool for Independent Learning in Nursing. Journal of Continuing Education in Nursing. 1971;2(3):27-31. | |
| 52 | Rejected by TIAB Screening | Adetayo OA, Ford RS, Nair L, Eliann Reinhardt M. The Oxymoron of Financial Illiteracy in a Highly Educated Population: Are We Appropriately Equipping Trainees? Plastic and Reconstructive Surgery - Global Open. 2019;7(7):e2329. | |
| 53 | Rejected by TIAB Screening | Adeyanju O, Pestak K, Foshee C. E-learning in pediatrics: Virtual patient scenarios. Academic Pediatrics. 2017;17 (5):e16. | |
| 54 | Rejected by TIAB Screening | Adler G, Von Dem Knesebeck J, Hanle MM. Quality of undergraduate, graduate and continuing medical education. [German]. Zeitschrift fur Evidenz, Fortbildung und Qualitat im Gesundheitswesen. 2008;102(4):235-43. | |
| 55 | Rejected by TIAB Screening | Adonizio C, Weber VD, Townsend J, Perlis SM, Clifton M. Implementing a longitudinal integrated clerkship (LIC) for the third year at the common wealth medial college. Journal of General Internal Medicine. 2012;27:S558. | |
| 56 | Not Target Group | Afifi M. Cross sectional study on lifelong learning's determinants among medical students in RAK Medical & Health Sciences University, UAE. JPMA - Journal of the Pakistan Medical Association. 2018;68(3):394-9. | |
| 57 | Rejected by TIAB Screening | Afrashtehfar KI, Assery MK. From dental science to clinical practice: Knowledge translation and evidence-based dentistry principles. Saudi Dental Journal. 2017;29(3):83-92. | |
| 58 | Rejected by TIAB Screening | Afzal MF, Ali AA, Hanif A. Performance of pediatrics' residents as clinical teachers: A student-based assessment. Pakistan Journal of Medical Sciences. 2019;35(6):1499-504. | |
| 59 | Rejected by TIAB Screening | Agre P, Dougherty J, Pirone J. Creating a CD-ROM program for cancer-related patient education. Oncology nursing forum. 2002;29(3):573-80. | |
| 60 | Rejected by TIAB Screening | Aherne M, Pereira J. A generative response to palliative service capacity in Canada. International Journal of Health Care Quality Assurance Incorporating Leadership in Health Services. 2005;18(1):iii-xxi. | |
| 61 | Rejected by TIAB Screening | Ahmed A, Abid MA, Bhatti NI. Balancing standardized testing with personalized training in surgery. Advances in Medical Education & Practice. 2017;8:25-9. | |
| 62 | Rejected by TIAB Screening | Ahmed HB. A qualitative and quantitative case study of the perceptions of health care students and health care faculty toward mentoring and mentoring relationships at the New York Chiropractic College, Seneca falls, New York. Dissertation Abstracts International Section A: Humanities and Social Sciences. 2010;70(7-A):2360. | |
| 63 | Rejected by TIAB Screening | Ahmed K, Amer T, Challacombe B, Jaye P, Dasgupta P, Khan MS. How to develop a simulation programme in urology. BJU International. 2011;108(11):1698-702. | |
| 64 | Rejected by TIAB Screening | Ahmed K, Wang TT, Ashrafian H, Layer GT, Darzi A, Athanasiou T. The effectiveness of continuing medical education for specialist recertification. Canadian Urological Association Journal. 2013;7(7-8):266-72. | |
| 65 | Rejected by TIAB Screening | Ahn D. What is the social competency for doctors?. [Korean]. Journal of the Korean Medical Association. 2014;57(2):96-103. | |
| 66 | Not Target Group | Aho JM, Ruparel RK, Graham E, Zendejas-Mummert B, Heller SF, Farley DR, et al. Mentor-guided self-directed learning affects resident practice. Journal of Surgical Education. 2015;72(4):674-9. | |
| 67 | Rejected by TIAB Screening | Aij KH, Simons FE, Widdershoven GAM, Visse M. Experiences of leaders in the implementation of Lean in a teaching hospital - Barriers and facilitators in clinical practices: A qualitative study. BMJ Open. 2013;3(10). | |
| 68 | Rejected by TIAB Screening | Airey N, Marriott J, Rodd J. Learning styles of psychiatrists and other specialists. Psychiatric Bulletin. 2001;25(8):306-9. | |
| 69 | Rejected by TIAB Screening | Ajjawi R, Hilder J, Noble C, Teodorczuk A, Billett S. Using video-reflexive ethnography to understand complexity and change practice. Medical education. 2020;14. | |
| 70 | Rejected by TIAB Screening | Akelina Y. Microsurgery training program at the Department of Orthopaedic Surgery, Columbia University, NY: Its application to the world of small lab animal science. Journal of Investigative Surgery. 2012;25 (1):51. | |
| 71 | Rejected by TIAB Screening | Akle V, Pena-Silva RA, Valencia DM, Rincon-Perez CW. Validation of clay modeling as a learning tool for the periventricular structures of the human brain. Anatomical Sciences Education. 2018;11(2):137-45. | |
| 72 | Rejected by TIAB Screening | Al-Ahmadi TA. Measuring Patient Safety Culture in Riyadh's Hospitals: A Comparison between Public and Private Hospitals. Journal of the Egyptian Public Health Association. 2009;84(5-6):479-500. | |
| 73 | Rejected by TIAB Screening | Al-Halabi B, Hazan J, Safran T, Gilardino MS. The Role of Resident-Run Clinics for Aesthetic Surgery Training in the Context of Competency-based Plastic Surgery Education. Plastic and Reconstructive Surgery - Global Open. 2020;8(4):e2766. | |
| 74 | Rejected by TIAB Screening | Al-Jasmi F, Moldovan L, Clarke J. Computer-assisted teaching of mucopolysaccharidosis by patient management problems. Molecular Genetics and Metabolism. 2009;96 (2):S12. | |
| 75 | Not Target Group | Al-Kadri HM, Al-Moamary MS, Al-Takroni H, Roberts C, van der Vleuten CP. Self-assessment and students' study strategies in a community of clinical practice: a qualitative study. Medical Education Online. 2012;17:11204. | |
| 76 | Rejected by TIAB Screening | Al-Rawi W, Jacobs R, Hassan B, Sanderink G, Scarfe W. Evaluation of web-based instruction for anatomical interpretation in maxillofacial cone beam computed tomography. Dento maxillo facial radiology [Internet]. 2007; 36(8):[459‐64 pp.]. Available from: https://www.cochranelibrary.com/central/doi/10.1002/central/CN-00628485/full. | |
| 77 | Rejected by TIAB Screening | Alahmadi HA. Assessment of patient safety culture in Saudi Arabian hospitals. Quality & Safety in Health Care. 2010;19(5):e17. | |
| 78 | Rejected by TIAB Screening | Alaszewski A, Motherby E, Gates B, Ayer S, Manthorpe J. Diversity and Change: The Changing Roles and Education of Learning Disability Nurses. Researching Professional Education Research Reports Series. Reports - Research. English National Board for Nursing, Midwifery and Health Visiting, London.; 2001. | |
| 79 | Not Target Group | Alba G, Kelmenson D, Noble V, Murray A, Currier P. Faculty staff-guided versus self-guided ultrasound training for internal medicine residents. Medical education [Internet]. 2013; 47(11):[1099‐108 pp.]. Available from: https://www.cochranelibrary.com/central/doi/10.1002/central/CN-01121019/full. | |
| 80 | Not Target Group | Alegria DA, Boscardin C, Poncelet A, Mayfield C, Wamsley M. Using tablets to support self-regulated learning in a longitudinal integrated clerkship. Medical Education Online. 2014;19:23638. | |
| 81 | Outside SDL | Alenezi AN, Yaiesh SM. The ubiquitous invasion of social media in lifelong learning in medical education: Review article. Kuwait Medical Journal. 2018;50(3):271-7. | |
| 82 | Rejected by TIAB Screening | Alessio H. Student Perceptions about and Performance in Problem-Based Learning. Journal of Scholarship of Teaching and Learning. 2004;4(1):23-34. | |
| 83 | Rejected by TIAB Screening | Alfaqawi M, Bottcher B, Abuowda Y, Alaloul E, Elnajjar I, Elhout S, et al. Treating patients in a safe environment: a cross-sectional study of patient safety attitudes among doctors in the Gaza Strip, Palestine. BMC health services research. 2020;20(1):388. | |
| 84 | Rejected by TIAB Screening | Alhaqwi AI, Taha WS. Promoting excellence in teaching and learning in clinical education. Journal of Taibah University Medical Sciences. 2015;10(1):97-101. | |
| 85 | Not Target Group | Alharbi HA. Readiness for self-directed learning: How bridging and traditional nursing students differs? Nurse Education Today. 2018;61:231-4. | |
| 86 | Rejected by TIAB Screening | Allan J, O'Meara P, Pope R, Higgs J, Kent J. The role of context in establishing university clinics. Health and Social Care in the Community. 2011;19(2):217-24. | |
| 87 | Formal Teaching | Allen JE, Vennalaganti P, Gupta N, Hornung B, Choudhary A, Titi M, et al. Randomized Controlled Trial of Self-directed Versus In-Classroom Education of Narrow Band Imaging in Diagnosing Colorectal Polyps Using the NICE Criteria. Journal of Clinical Gastroenterology. 2018;52(5):413-7. | |
| 88 | Rejected by TIAB Screening | Allen S, Waterfield J, Rivers P. An investigation of pharmacy student perception of competence-based learning using the individual skills evaluation and development program, iSED. Pharmacy Education. 2016;16(1):72-80. | |
| 89 | Rejected by TIAB Screening | Allen T, Donde N, Hofstadter-Thalmann E, Keijser S, Moy V, Murama JJ, et al. Framework for industry engagement and quality principles for industry-provided medical education in Europe. Journal of European Cme. 2017;6(1):1348876. | |
| 90 | Rejected by TIAB Screening | Almy TP, Cohen RD, Ham TH, Hornig EO, Price J. Health-related experimental learning for college undergraduates. Journal of Medical Education. 1983;58(5):404-10. | |
| 91 | Rejected by TIAB Screening | Alnabelsi T, Al-Hussaini A, Owens D. Comparison of traditional face-to-face teaching with synchronous e-learning in otolaryngology emergencies teaching to medical undergraduates: a randomised controlled trial. European archives of oto-rhino-laryngology [Internet]. 2015; 272(3):[759‐63 pp.]. Available from: https://www.cochranelibrary.com/central/doi/10.1002/central/CN-01043475/full. | |
| 92 | Rejected by TIAB Screening | Alpert C, Hummel E, Hummel S, Gauvreau K, Bohannon K, Cooper S, et al. Mind the gap: Palliative care knowledge among cardiovascular clinicians. Journal of the American College of Cardiology. 2016;67(13):1553. | |
| 93 | Rejected by TIAB Screening | Alpi KM, Burnett HA, Bryant SJ, Anderson KM. Connecting knowledge resources to the veterinary electronic health record: Opportunities for learning at point of care. Journal of Veterinary Medical Education. 2011;38(2):110-22. | |
| 94 | Rejected by TIAB Screening | Altman M. Let's get certified: best practices for nurse leaders to create a culture of certification. AACN Advanced Critical Care. 2011;22(1):68-75. | |
| 95 | Rejected by TIAB Screening | Altmiller G. Clinical Educator as Coach: Supporting Staff to Meet Quality and Safety Competencies. Journal of Continuing Education in Nursing. 2019;50(5):201-4. | |
| 96 | Rejected by TIAB Screening | Alvarez DM. Socio-economic and psycho-affective factors and their influence on academic performance of residents in obstetrics and Gynecology. [Spanish]. Ginecologia y Obstetricia de Mexico. 2015;83(3):139-47. | |
| 97 | Rejected by TIAB Screening | Alvarez-Maestro M, Gomez Rivas J, Aguilera Bazan A, Martinez-Pineiro L, Juarez Soto A, Cozar Olmo JM, et al. [Training program in oncologic urology. Future prospectives.]. Archivos Espanoles de Urologia. 2018;71(1):108-13. | |
| 98 | Rejected by TIAB Screening | Alves S, Campos M. Obstetric epidural catheter placement: Learning curve. European Journal of Anaesthesiology. 2014;31:185-6. | |
| 99 | Rejected by TIAB Screening | Amadio J, Rambihar S, Sozio S, Brydges R, Nesbitt G. Enhancing Resident Competency Assessments in Transthoracic Echocardiography. Journal of the American College of Cardiology. 2020;75 (11 Supplement 1):3486. | |
| 100 | Rejected by TIAB Screening | American Academy of Pediatrics Committee on Pediatric W, Shipman SA, Pan RJ. Financing graduate medical education to meet the needs of children and the future pediatrician workforce. Pediatrics. 2008;121(4):855-61. | |
| 101 | Rejected by TIAB Screening | Amin Z. Theory and practice in continuing medical education. Annals of the Academy of Medicine, Singapore. 2000;29(4):498-502. | |
| 102 | Rejected by TIAB Screening | Aminoff MJ, Faulkner LR. The American Board of Psychiatry and Neurology: Looking back and moving ahead. The American Board of Psychiatry and Neurology: Looking back and moving ahead. Arlington, VA: American Psychiatric Publishing, Inc; 2012. p. xix, 343. | |
| 103 | Rejected by TIAB Screening | Amphlett AM, Heaton S, Freeman-Fielding N, Bourdeaux C. Quick-Link Learning: Introduction of rapid-access educational resources on the intensive care unit. Intensive Care Medicine Experimental Conference: 32nd European Society of Intensive Care Medicine Annual Congress, ESICM. 2019;7(Supplement 3). | |
| 104 | Rejected by TIAB Screening | Andersen SA. Virtual reality simulation training of mastoidectomy - studies on novice performance. Danish Medical Journal. 2016;63(8). | |
| 105 | Outside SDL | Andersen SAW, Frendo M, Guldager M, Sorensen MS. Understanding the effects of structured self-assessment in directed, self-regulated simulation-based training of mastoidectomy: A mixed methods study. Journal of Otology. 2019. | |
| 106 | Rejected by TIAB Screening | Anderson C. Training efforts to reduce reports of workplace violence in a community health care facility. Journal of professional nursing [Internet]. 2006; 22(5):[289‐95 pp.]. Available from: https://www.cochranelibrary.com/central/doi/10.1002/central/CN-00572349/full. | |
| 107 | Rejected by TIAB Screening | Anderson J, Murphy A, Boyle K, Yaeger K, Halamek L. Simulating extracorporeal membrane oxygenation emergencies to improve human performance. Part II: assessment of technical and behavioral skills. Simulation in healthcare [Internet]. 2006; 1(4):[228‐32 pp.]. Available from: https://www.cochranelibrary.com/central/doi/10.1002/central/CN-00665940/full. | |
| 108 | Outside SDL | Anderson MC. New opportunities to improve physicians' lifelong learning. Academic Medicine. 1996;71(2):115-6. | |
| 109 | Rejected by TIAB Screening | Anderson R, McCafferty JR. Competencies: a tool for continuous learning and change. Aspen's Advisor for Nurse Executives. 1996;11(8):1, 3-6. | |
| 110 | Rejected by TIAB Screening | Andersson N, Klang B, Petersson G. Differences in clinical reasoning among nurses working in highly specialised paediatric care. Journal of Clinical Nursing. 2012;21(5-6):870-9. | |
| 111 | Outside SDL | Anonymous. Lifelong learning: options in continuing education. Journal of the American Dental Association. 1988;116(7):825-32. | |
| 112 | Outside SDL | Anonymous. Evidence-based care: 5. Lifelong learning: how can we learn to be more effective? Evidence-Based Care Resource Group. CMAJ Canadian Medical Association Journal. 1994;150(12):1971-3. | |
| 113 | Outside SDL | Anonymous. Communication and lifelong learning. Nursing Times. 2000;96(22):39-42. | |
| 114 | Rejected by TIAB Screening | Anonymous. Ensuring culturally effective pediatric care: Implications for education and health policy. Pediatrics. 2004;114(6):1677-85. | |
| 115 | Rejected by TIAB Screening | Anonymous. Demonstrating Your Clinical Competence in Women's Health Campbell Pam et al Demonstrating Your Clinical Competence in Women's Health 216pp Radcliffe Publishing 9781857756050 1857756053. Nursing Standard. 2005;19(32):36. | |
| 116 | Rejected by TIAB Screening | Anonymous. The Good Appraisal Toolkit for Primary Care Chambers Ruth et al The Good Appraisal Toolkit for Primary Care 189pp Radcliffe Publishing 9781857756029 1857756029. Nursing Standard. 2005;19(25):28. | |
| 117 | Formal Teaching | Anonymous. Lifelong learning. RCM Midwives. 2010:36-7. | |
| 118 | Rejected by TIAB Screening | Anonymous. Abstracts of the Canadian Conference on Medical Education, CCME 2010. Medical Education, Supplement Conference: Canadian Conference on Medical Education, CCME. 2010;44(SUPPL. 2). | |
| 119 | Rejected by TIAB Screening | Anonymous. 2012 Paramedics Australasia Conference. Australasian Journal of Paramedicine Conference. 2012;10(3). | |
| 120 | Rejected by TIAB Screening | Anonymous. Monash Pharmacy Education Symposium 2013 - Teaching for Learning. Pharmacy Education. 2013;13 (1):93. | |
| 121 | Rejected by TIAB Screening | Anonymous. Singapore Health and Biomedical Congress, SHBC 2013. Annals of the Academy of Medicine Singapore Conference: Singapore Health and Biomedical Congress, SHBC. 2013;42(Supplement 9). | |
| 122 | Not Target Group | Anonymous. Lifelong learning and the healthcare executive. Healthcare Executive. 2014;29(2):102-3. | |
| 123 | Rejected by TIAB Screening | Anonymous. Abstracts from the 40th Annual Meeting of the Society of General Internal Medicine, SGIM 2017. Journal of General Internal Medicine Conference: 40th Annual Meeting of the Society of General Internal Medicine, SGIM. 2017;32(2 Supplement 1). | |
| 124 | Rejected by TIAB Screening | Ansted CJ, Johnson MD, Binford SH, Kennedy RS. Continuing professional development in psychiatry and neurology: The 3 <sup>rd</sup> annual chair summit. Health Outcomes Research in Medicine. 2011;2(3):e133-e40. | |
| 125 | Formal Teaching | Antonoff MB. Expanding surgical skills and knowledge after training: Lifelong learning puts patients first. Journal of Thoracic & Cardiovascular Surgery. 2017;154(1):380-1. | |
| 126 | Rejected by TIAB Screening | Apgar C. Making it count: key factors to consider when assessing continuing professional educational offers. Journal of Trauma Nursing. 1999;6(1):6-14. | |
| 127 | Rejected by TIAB Screening | Appel AL, Malcolm PA. Specialist education and practice in nursing: an Australian perspective. Nurse education today. 1998;18(2):144-52. | |
| 128 | Rejected by TIAB Screening | Applegate K. Image Gently: A campaign to promote radiation protection for children worldwide. South African Journal of Radiology. 2015;19(2). | |
| 129 | Rejected by TIAB Screening | Archer-Dyer HA, Cruz AJ, Blanco I, Grayson MS. Community based service learning: A shift from student clubs to an educational curriculum. Journal of General Internal Medicine. 2016;31(2):S811. | |
| 130 | Rejected by TIAB Screening | Arfanis K, Campbell G, Smith AF. Distractions, interruptions and their impact on patient safety. Anaesthetists' perceptions and existing coping strategies. European Journal of Anaesthesiology. 2012;29:217-8. | |
| 131 | Not Target Group | Arias A, Peters OA, Broyles IL. New curricular design in biostatistics to prepare residents for an evidence-based practice and lifelong learning education: a pilot approach. International Endodontic Journal. 2017;50(10):999-1010. | |
| 132 | Rejected by TIAB Screening | Arif S, Zehireva S, Sridhar SB, Rabbani SA. Implementation of a continuing education series to promote interprofessional collaboration and evidence based practice in the United Arab Emirates. JACCP Journal of the American College of Clinical Pharmacy. 2020;3 (1):326. | |
| 133 | Rejected by TIAB Screening | Arif SA, Dilich A, Ramel C, Strong S. Impact of an interprofessional international experience abroad on the attitudes of health care professional students. Currents in Pharmacy Teaching and Learning. 2014;6(5):639-45. | |
| 134 | Rejected by TIAB Screening | Arita E. Why pharmacists need education in the humanities: A medical professional educator's viewpoint. [Japanese]. Yakugaku Zasshi. 2020;140(3):411-4. | |
| 135 | Rejected by TIAB Screening | Armbrust W, Bos J, Cappon J, van RM, Sauer P, Wulffraat N, et al. Design and acceptance of Rheumates@Work, a combined internet-based and in person instruction model, an interactive, educational, and cognitive behavioral program for children with juvenile idiopathic arthritis. Pediatric rheumatology online journal [Internet]. 2015; 13:[31 p.]. Available from: https://www.cochranelibrary.com/central/doi/10.1002/central/CN-01258953/full. | |
| 136 | Rejected by TIAB Screening | Armstrong AR. Relationships between individual learning style differences and cognitive, affective, and behavioral attitudes toward interactive videodisc training programs among multi-skilled health care professionals. Dissertation Abstracts International Section A: Humanities and Social Sciences. 1995;56(3-A):0801. | |
| 137 | Rejected by TIAB Screening | Armstrong L, Shepherd A, Harris F. An evaluation of approaches used to teach quality improvement to pre-registration healthcare professionals: An integrative review. International journal of nursing studies. 2017;73:70-84. | |
| 138 | Outside SDL | Armstrong ML, Gessner BA. Is reading part of the lifelong learning process for RNs? Journal of Nursing Staff Development. 1991;7(1):21-6. | |
| 139 | Outside SDL | Armstrong ML, Gessner BA. Lifelong learning: putting reading first. Nursing Management. 1992;23(6):61. | |
| 140 | Rejected by TIAB Screening | Armstrong ML, Johnston BA, Bridges RA, Gessner BA. The impact of graduate education on reading for lifelong learning. Journal of Continuing Education in Nursing. 2003;34(1):19-25. | |
| 141 | Rejected by TIAB Screening | Armstrong P, Elliott T, Ronald J, Paterson B. Comparison of traditional and interactive teaching methods in a UK emergency department. European journal of emergency medicine [Internet]. 2009; 16(6):[327‐9 pp.]. Available from: https://www.cochranelibrary.com/central/doi/10.1002/central/CN-00767663/full. | |
| 142 | Rejected by TIAB Screening | Aronson L, Rivera J, Macking L. An innovative program to geriatricize non-geriatrics health professionals. Journal of the American Geriatrics Society. 2014;62:S2. | |
| 143 | Rejected by TIAB Screening | Aronson L, Schwalbe W. The art and craft of writing for self-care and narrative advocacy: A workshop in reflective and public writing. Journal of Pain and Symptom Management. 2015;49 (2):322. | |
| 144 | Rejected by TIAB Screening | Arora S, Kalishman S, Thornton K, Dion D, Murata G, Deming P, et al. Expanding access to hepatitis C virus treatment--Extension for Community Healthcare Outcomes (ECHO) project: disruptive innovation in specialty care. Hepatology. 2010;52(3):1124-33. | |
| 145 | Rejected by TIAB Screening | Arrington NM. The Effects of Participating in a Service-Learning Experience on the Development of Self-Efficacy for Self-Regulated Learning of Third Graders in an Urban Elementary School in Southeastern United States. ProQuest LLC PhD Dissertation, Clemson University. 2010. | |
| 146 | Formal Teaching | Artino Jr AR, Dong T, Dezee KJ, Gilliland WR, Waechter DM, Cruess D, et al. Achievement goal structures and self-regulated learning: Relationships and changes in medical school. Academic Medicine. 2012;87(10):1375-81. | |
| 147 | Rejected by TIAB Screening | Aryasomayajula S, Raithatha A, Haywood M, Jobanputra R, Roplekar R, Acharya V. Hands-on teaching, shadowing, and supported learning through acute clinics to help improve the confidence of and meet training needs for junior doctors working in ear, nose, and throat surgery. Advances in Medical Education & Practice. 2018;9:827-35. | |
| 148 | Formal Teaching | Arzouman J. Pomp and Circumstance and Lifelong Learning. MEDSURG Nursing. 2015;24(3):141-2. | |
| 149 | Rejected by TIAB Screening | Asare AA. A few good faculty. The New York state dental journal. 2008;74(3):23-7. | |
| 150 | Rejected by TIAB Screening | Ashwin C, Hutchinson JC, Kang X, Langan D, Jones R, Norman W, et al. Learning effect on perinatal post-mortem magnetic resonance imaging reporting: single reporter diagnostic accuracy of 200 cases. Prenatal Diagnosis. 2017;37(6):566-74. | |
| 151 | Rejected by TIAB Screening | Assael L. Education of the oral and maxillofacial surgeon in the 21st century. International Journal of Oral and Maxillofacial Surgery. 2011;40 (10):1012-3. | |
| 152 | Rejected by TIAB Screening | Assiter A. Cognitive Skills in Workplace Learning? Reports - Descriptive Speeches/Meeting Papers. 1994. | |
| 153 | Rejected by TIAB Screening | Atack L. Becoming a web-based learner: Registered nurses' experiences. Journal of Advanced Nursing. 2003;44(3):289-97. | |
| 154 | Rejected by TIAB Screening | Attwood ML, Cheney-Stern MR. Developing Leadership Skills in Allied Health and Nursing Students. Journal of Studies in Technical Careers. 1983;5(1):79-86. | |
| 155 | Formal Teaching | August-Brady MM. The effect of a metacognitive intervention on approach to and self-regulation of learning in baccalaureate nursing students. Journal of Nursing Education. 2005;44(7):297-304. | |
| 156 | Rejected by TIAB Screening | Averns H, Maraschiello M, van ME, Day A. Evaluation of a web-based teaching module on examination of the hand. Journal of rheumatology [Internet]. 2009; 36(3):[623‐7 pp.]. Available from: https://www.cochranelibrary.com/central/doi/10.1002/central/CN-00684773/full. | |
| 157 | Rejected by TIAB Screening | Axton C. Time to train: Development of an eModule for swallow screen training. International Journal of Stroke. 2015;10:42. | |
| 158 | Rejected by TIAB Screening | Ayello EA, Zulkowski K, Capezuti E, Jicman WH, Sibbald RG. Educating Nurses in the United States about Pressure Injuries. Advances in Skin & Wound Care. 2017;30(2):83-94. | |
| 159 | Rejected by TIAB Screening | Ayoub J, Vanderboom C, Knight M, Walsh K, Briggs R, Grekin K. A study of the effectiveness of an interactive computer classroom. Computers in nursing [Internet]. 1998; 16(6):[333‐8 pp.]. Available from: https://www.cochranelibrary.com/central/doi/10.1002/central/CN-00157681/full. | |
| 160 | Rejected by TIAB Screening | Azarpazhooh A, Mayhall JT, Leake JL. Introducing dental students to evidence-based decisions in dental care. Journal of Dental Education. 2008;72(1):87-109. | |
| 161 | Rejected by TIAB Screening | Azer SA. Do recommended textbooks contain adequate information about bile salt transporters for medical students? American Journal of Physiology - Advances in Physiology Education. 2004;28:36-43. | |
| 162 | Rejected by TIAB Screening | Azer SA. Evaluation of gastroenterology and hepatology articles on Wikipedia: Are they suitable as learning resources for medical students? European Journal of Gastroenterology and Hepatology. 2014;26(2):155-63. | |
| 163 | Rejected by TIAB Screening | Babajide AJ, J. K. Lester, P. Training Child Psychiatry Fellows in Pediatric Integrated Behavioral Health: An Examination of Key Rotation Components and Perceived Value by Trainees. Journal of the American Academy of Child and Adolescent Psychiatry. 2018;57 (10 Supplement):S153. | |
| 164 | Rejected by TIAB Screening | Babenko O, Daniels LM, White J, Oswald A, Ross S. Achievement Goals of Medical Students and Physicians. Educational Research and Reviews. 2018;13(2):74-80. | |
| 165 | Outside SDL | Babenko O, Koppula S, Daniels L, Nadon L, Daniels V. Lifelong learning along the education and career continuum: meta-analysis of studies in health professions. Journal of Advances in Medical Education & Professionalism. 2017;5(4):157-63. | |
| 166 | Outside SDL | Babenko ON, L. Koppula, S. Lifelong learning in health professions Meta-analysis along the education and career continuum. Canadian Family Physician. 2018;64 (2 Supplement 1):S12. | |
| 167 | Rejected by TIAB Screening | Babione JM. Evidence-based practice in psychology: An ethical framework for graduate education, clinical training, and maintaining professional competence. Ethics & Behavior. 2010;20(6):443-53. | |
| 168 | Rejected by TIAB Screening | Bach CA-S, P. Strouth, A. Blackburn, S. Dial "M" for mentor: Mentorship in the life of the oncology social worker. Journal of Psychosocial Oncology. 2016;34 (1-2):119-20. | |
| 169 | Rejected by TIAB Screening | Badia M, Vicario E, Garcia-Solanes L, Servia L, Justes M, Trujillano J. [Pediatric critical care in an adult ICU. Utility of the PIM index]. Medicina Intensiva. 2013;37(2):83-90. | |
| 170 | Rejected by TIAB Screening | Bahadori M, Soltanzadeh P, Salimi M, Raadabadi M, Moghri J, Ravangard R. Application of WHO model for evaluating Patient Safety Friendly Hospital Initiatives (PSFHI) in an Eye hospital in Tehran, Iran. Electronic Physician [Electronic Resource]. 2013;5(2):631-6. | |
| 171 | Rejected by TIAB Screening | Bai X, Zhang X, Wang X, Lu L, Liu Q, Zhou Q. Follow-up assessment of problem-based learning in dental alveolar surgery education: a pilot trial. International Dental Journal. 2017;67(3):180-5. | |
| 172 | Rejected by TIAB Screening | Baier PC, May U, Scheller J, Rose-John S, Schiffelholz T. Impaired hippocampus-dependent and -independent learning in IL-6 deficient mice. Behavioural Brain Research. 2009;200(1):192-6. | |
| 173 | Rejected by TIAB Screening | Bailey JV, Murray E, Rait G, Mercer CH, Morris RW, Peacock R, et al. Interactive computer‐based interventions for sexual health promotion. Cochrane Database of Systematic Reviews. 2010(9). | |
| 174 | Rejected by TIAB Screening | Bailey MET, D. Student nurses' experiences of using a learning contract as a method of assessment. Nurse Education Today. 2009;29(7):758-62. | |
| 175 | Rejected by TIAB Screening | Bajalan MA, Z. Roberts, S. Educational philosophies within medical graduates undertaking a dental degree when compared with other non-medical graduates. International Journal of Oral and Maxillofacial Surgery. 2017;46 (Supplement 1):365. | |
| 176 | Rejected by TIAB Screening | Baker MC. A descriptive study of the view from the top: Perspectives of experts in continuing medical education. Dissertation Abstracts International Section A: Humanities and Social Sciences. 2011;71(11-A):3883. | |
| 177 | Rejected by TIAB Screening | Baldwin DC, Jr., Daugherty SR. How Residents Say They Learn: A National, Multi-Specialty Survey of First- and Second-Year Residents. Journal of Graduate Medical Education. 2016;8(4):631-9. | |
| 178 | Rejected by TIAB Screening | Bandara IC, G. General practitioners: Uncelebrated adult learners - A qualitative study. Education for Primary Care. 2002;13(3):370-8. | |
| 179 | Rejected by TIAB Screening | Barbato A, et al. Comparison of Graduates of Regular Curriculum and Unified Basic-Science--Clinical Curriculum. Journal of Medical Education. 1988;63(7):505-14. | |
| 180 | Rejected by TIAB Screening | Barbato A, Frazier R, Leischner R, Gunzburger L, Loesch T, Yang LM. Comparison of graduates of regular curriculum and unified basic-science-clinical curriculum. Journal of Medical Education. 1988;63(7):505-14. | |
| 181 | Rejected by TIAB Screening | Barker C, King N, Snowden M, Ousey K. Study time within pre-registration nurse education: A critical review of the literature. Nurse Education Today. 2016;41:17-23. | |
| 182 | Rejected by TIAB Screening | Barker G, et al. Rights & Responsibilities. Personnel Management Module. Guides - Classroom - Learner. Hotel and Catering Training Co., London (England). 1991. | |
| 183 | Rejected by TIAB Screening | Baron RM, D. Martin, J. Ward, B. Whiteman, I. Higher professional education for GPs in the North West of England - Feedback from the first three years. Education for Primary Care. 2001;12(4):421-9. | |
| 184 | Rejected by TIAB Screening | Barrett JL, Denegar CR, Mazerolle SM. Challenges Facing New Educators: Expanding Teaching Strategies for Clinical Reasoning and Evidence-Based Medicine. Athletic Training Education Journal. 2018;13(4):359-66. | |
| 185 | Rejected by TIAB Screening | Barrett JT, S. C. McColl, G. Novice students navigating the clinical environment in an early medical clerkship. Medical education. 2017;51(10):1014-24. | |
| 186 | Rejected by TIAB Screening | Barrett TJ. Self-directed learning in family medicine. Handbook of research on adult and community health education: Tools, trends, and methodologies. Hershey, PA: Medical Information Science Reference/IGI Global; US; 2014. p. 141-63. | |
| 187 | Rejected by TIAB Screening | Barrow EJ, Lyte G, Butterworth T. An evaluation of problem-based learning in a nursing theory and practice module. Nurse Education in Practice. 2002;2(1):55-62. | |
| 188 | Rejected by TIAB Screening | Barrows HS. The Scope of Clinical Education. Journal of Medical Education. 1986;61(9):23-33. | |
| 189 | Rejected by TIAB Screening | Barrows HS. Practice-Based Learning: Problem-Based Learning Applied to Medical Education. Guides - Classroom - Teacher. Southern Illinois Univ., Carbondale. School of Medicine.; 1994. | |
| 190 | Rejected by TIAB Screening | Barry C. Case based learning exercises versus informal teaching session to enhance educational experience on rheumatology rotation. Journal of Rheumatology. 2018;45 (7):1037. | |
| 191 | Rejected by TIAB Screening | Barthelemy F, Segard J, Fradin P, Hourdin N, Batard E, Pottier P, et al. ECG interpretation in Emergency Department residents: an update and e-learning as a resource to improve skills. European journal of emergency medicine [Internet]. 2017; 24(2):[149‐56 pp.]. Available from: https://www.cochranelibrary.com/central/doi/10.1002/central/CN-01341265/full. | |
| 192 | Rejected by TIAB Screening | Bartholomew L, Gold R, Parcel G, Czyzewski D, Sockrider M, Fernandez M, et al. Watch, Discover, Think, and Act: evaluation of computer-assisted instruction to improve asthma self-management in inner-city children. Patient education and counseling [Internet]. 2000; 39(2‐3):[269‐80 pp.]. Available from: https://www.cochranelibrary.com/central/doi/10.1002/central/CN-00361675/full. | |
| 193 | Outside SDL | Bashook P, Meyer TC, Richards RK, Long RJ, Parboosingh J. Symposium: Self-directed learning and physicians' practice changes: concepts, research and implications for CME. Research in medical education:proceedings of the annual Conference. 1986;25:329-38. | |
| 194 | Rejected by TIAB Screening | Battrell AM, Gadbury-Amyot CC, Overman PR. A qualitative study of limited access permit dental hygienists in Oregon. Journal of Dental Education. 2008;72(3):329-43. | |
| 195 | Rejected by TIAB Screening | Bauer J, Mulder RH. Modelling learning from errors in daily work. Learning in Health and Social Care. 2007;6(3):121-33. | |
| 196 | Rejected by TIAB Screening | Baxter SG, C. The application of student-centred learning approaches to clinical education. International Journal of Language and Communication Disorders. 2001;36(SUPPL.):396-400. | |
| 197 | Rejected by TIAB Screening | Bazan MT, Seale WS. A Combined Teaching Approach for a Preclinical Technique Course. Journal of Dental Education. 1983;47(5):343-45. | |
| 198 | Rejected by TIAB Screening | Beal JB, L. Sims, S. Stevenson, G. Tomaszewski, G. Advancing stroke expertise: Evaluating the Southwestern Ontario Stroke Network Acute Stroke Unit Orientation. International Journal of Stroke. 2015;10:61. | |
| 199 | Rejected by TIAB Screening | Beal T, Kemper K, Gardiner P, Woods C. Long-term impact of four different strategies for delivering an on-line curriculum about herbs and other dietary supplements. BMC medical education [Internet]. 2006; 6:[39 p.]. Available from: https://www.cochranelibrary.com/central/doi/10.1002/central/CN-00567159/full. | |
| 200 | Rejected by TIAB Screening | Beasley HLG, H. N. Wiegmann, D. A. Brys, N. A. Quamme, S. R. P. Greenberg, C. C. Strategies for building peer surgical coaching relationships. JAMA Surgery. 2017;152(4). | |
| 201 | Rejected by TIAB Screening | Beatriz MS, C. D. Mestre, M. Rodriguez, A. Sanchez-Rubiera, N. Applying lean initiatives to inpatient rounds to improve discharge delays. Academic Pediatrics. 2010;10 (4):e8. | |
| 202 | Formal Teaching | Beaty JH. Presidential address: Building the best... lifelong learning. Journal of the American Academy of Orthopaedic Surgeons. 2007;15(9):515-8. | |
| 203 | Rejected by TIAB Screening | Beaupre BA. An administrative marketing strategy: a different perspective on the nursing process. Journal of Nursing Administration. 1988;18(11):37-41. | |
| 204 | Rejected by TIAB Screening | Beausaert S, Segers M, Gijselaers W. The Personal Development Plan Practice Questionnaire: The Development and Validation of an Instrument to Assess the Employee's Perception of Personal Development Plan Practice. International Journal of Training and Development. 2011;15(4):249-70. | |
| 205 | Rejected by TIAB Screening | Beck S, Blake-Campbell B, McKay D. Partnership for the Advancement of Information Literacy in a Nursing Program. Community and Junior College Libraries. 2012;18(1):3-11. | |
| 206 | Rejected by TIAB Screening | Beck SD, C. Funk, H. Kosan, J. Issleib, M. Daubmann, A. Zollner, C. Kubitz, J. C. Basic life support training using shared mental models improves team performance of first responders on normal wards: A randomised controlled simulation trial. Resuscitation. 2019;144:33-9. | |
| 207 | Rejected by TIAB Screening | Beckingham AC. Models for Continuing Nursing Education in Gerontology. Educational Gerontology. 1995;21(1):35-53. | |
| 208 | Rejected by TIAB Screening | Bedforth NM. ESRA: How is competence defined when learning regional anaesthesia? Regional Anesthesia and Pain Medicine. 2013;38(5):E60-E2. | |
| 209 | Rejected by TIAB Screening | Beglinger JE. CNOs Gearing Up While Many are Winding Down: The Late Career DNP. Journal of Nursing Administration. 2016;46(3):109-10. | |
| 210 | Rejected by TIAB Screening | Behar-Horenstein LS, Zafar MA, Roberts KW. Impact of Faculty Development on Physical Therapy Professors' Beliefs. Journal of Faculty Development. 2012;26(2):37-46. | |
| 211 | Rejected by TIAB Screening | Beldham-Collins RM, D. A new graduate education program in radiation therapy. Radiography. 2009;15(1):26-33. | |
| 212 | Rejected by TIAB Screening | Belfi LMB, R. J. Giambrone, A. E. Davi, C. Min, R. J. "Flipping" the introductory clerkship in radiology: impact on medical student performance and perceptions. Academic Radiology. 2015;22(6):794-801. | |
| 213 | Outside SDL | Bell FR, P. Attitudes of nurses toward lifelong learning: one hospital examines the issues. Journal of Continuing Education in Nursing. 1979;10(1):15-20. | |
| 214 | Rejected by TIAB Screening | Bell HMM, T. A. McGartland, L. F. Perceptions of continuing professional development within the pharmacy profession. Journal of Social and Administrative Pharmacy. 2002;19(3):87-98. | |
| 215 | Rejected by TIAB Screening | Bellido I. A brief historical review of new technologies in the teaching of pharmacology. Basic and Clinical Pharmacology and Toxicology. 2011;109:7. | |
| 216 | Rejected by TIAB Screening | Bellman PH, C. Bertolucci, Y. Streeter, B. Facilitating physician access to medical reference information. Permanente Journal. 2005;9(4):27-32. | |
| 217 | Rejected by TIAB Screening | Bembenutty H. New Directions for Self-Regulation of Learning in Postsecondary Education. New Directions for Teaching and Learning n126 p117. 2011;124. | |
| 218 | Rejected by TIAB Screening | Benbassat J, Baumal R. Viewpoint: A proposal for teaching basic clinical skills for mastery: The case against vertical integration. Academic Medicine. 2007;82(1):83-91. | |
| 219 | Rejected by TIAB Screening | Benbassat JB, R. A proposal for teaching basic clinical skills for mastery: the case against vertical integration. Academic Medicine. 2007;82(1):83-91. | |
| 220 | Rejected by TIAB Screening | Bendiak GN. SPITTS: A novel teaching strategy for use in the cystic fibrosis clinic. Pediatric Pulmonology. 2013;48:448. | |
| 221 | Rejected by TIAB Screening | Benn R, Fieldhouse R. Training and Professional Development in Adult and Continuing Education. Occasional Paper Number 1. Collected Works - Proceedings Reports - Research. Exeter Univ. (England). Centre for Research in Continuing Education.; 1994. | |
| 222 | Rejected by TIAB Screening | Bennett DM, M. O'Flynn, S. Kelly, M. In the eye of the beholder: student perspectives on professional roles in practice. Medical Education. 2013;47(4):397-407. | |
| 223 | Rejected by TIAB Screening | Bennett EE. How an intranet provides opportunities for learning organizational culture: Implications for virtual HRD. Advances in Developing Human Resources. 2014;16(3):296-319. | |
| 224 | Rejected by TIAB Screening | Bennett JHB, E. R. Lowry, J. Derry, J. Defining the educational needs of recent dental graduates preparing for the Membership of the Faculty of Dental Surgery examination. British Dental Journal. 2005;Suppl:21-5. | |
| 225 | Rejected by TIAB Screening | Bennett S, Bennett JW. The process of evidence-based practice in occupational therapy: Informing clinical decisions. Australian Occupational Therapy Journal. 2000;47(4):171-80. | |
| 226 | Rejected by TIAB Screening | Berduzco-Torres NC-C, B. Medina, P. Chihuantito-Abal, L. A. Caballero, S. Gallegos, E. San-Martin, M. Delgado Bolton, R. C. Vivanco, L. Factors Related to the Differential Development of Inter-Professional Collaboration Abilities in Medicine and Nursing Students. Frontiers in Psychology. 2020;11:432. | |
| 227 | Rejected by TIAB Screening | Berg Jansson A, Engstrom A, Parding K. What about Learning?: A Study of Temporary Agency Staffing and Learning Conditions in Swedish Health Care. Journal of Workplace Learning. 2020;32(1):63-75. | |
| 228 | Rejected by TIAB Screening | Berg M. Learning disabilities in children with borderline personality disorder. Bulletin of the Menninger Clinic. 1992;56(3):379-92. | |
| 229 | Rejected by TIAB Screening | Berglund MF, R. Fransson, M. Hultcrantz, M. Eriksson, P. O. Englund, E. Westman, E. Myringoplasty Outcomes From the Swedish National Quality Registry. Laryngoscope. 2017;127(10):2389-95. | |
| 230 | Formal Teaching | Bergman EM, Sieben JM, Smailbegovic I, de Bruin AB, Scherpbier AJ, van der Vleuten CP. Constructive, collaborative, contextual, and self-directed learning in surface anatomy education. Anatomical sciences education. 2013;6(2):114-24. | |
| 231 | Rejected by TIAB Screening | Bergman J, Lorenz K, Acquah-Asare S, Scales C, Ryan G, Saigal C, et al. Urologist attitudes toward end-of-life care. Journal of Urology. 2013;189(4):e169. | |
| 232 | Rejected by TIAB Screening | Bergman J, Lorenz KA, Scales CD, Ryan G, Saigal C, Bennett CJ, et al. Avenues to integrate and improve care: Surgeon attitudes toward end-of-life care. Journal of Clinical Oncology Conference: ASCO's Quality Care Symposium. 2012;30(34 SUPPL. 1). | |
| 233 | Rejected by TIAB Screening | Bergman JL, K. A. Ballon-Landa, E. Kwan, L. Lerman, S. E. Saigal, C. S. Bennett, C. J. Litwin, M. S. A Scalable Web-Based Module for Improving Surgical and Medical Practitioner Knowledge and Attitudes about Palliative and End-of-Life Care. Journal of Palliative Medicine. 2015;18(5):415-20. | |
| 234 | Rejected by TIAB Screening | Bergold M, Strametz R, Weinbrenner S, Khan K, Zamora J, Moll P, et al. Evidence-based Medicine online for young doctors - a randomised controlled trial. Zeitschrift fur Evidenz, Fortbildung und Qualitat im Gesundheitswesen [Internet]. 2013; 107(1):[36‐43 pp.]. Available from: https://www.cochranelibrary.com/central/doi/10.1002/central/CN-00872356/full. | |
| 235 | Not Target Group | Berkhout JJ, Helmich E, Teunissen PW, van den Berg JW, van der Vleuten CP, Jaarsma A. Exploring the factors influencing clinical students' self-regulated learning. Medical Education. 2015;49(6):589-600. | |
| 236 | Not Target Group | Berkhout JJH, E. Teunissen, P. W. van der Vleuten, C. P. Jaarsma, A. D. How clinical medical students perceive others to influence their self-regulated learning. Medical Education. 2017;51(3):269-79. | |
| 237 | Rejected by TIAB Screening | Berlin LS. Proceedings of the Annual Midwest Research-to-Practice Conference in Adult and Continuing Education (4th, Ann Arbor, Michigan, October 10-11, 1985). Collected Works - Proceedings Reports - Research Opinion Papers. Michigan Univ., Ann Arbor. School of Education.; 1985. | |
| 238 | Rejected by TIAB Screening | Berlinger N, Zacharias RL. Resources for Teaching and Learning About Immigrant Health Care in Health Professions Education. AMA journal of ethics. 2019;21(1):E50-E7. | |
| 239 | Rejected by TIAB Screening | Bernard JS. The flipped classroom: Fertile ground for nursing education research. International Journal of Nursing Education Scholarship (IJNES). 2015;12(1):99-109. | |
| 240 | Rejected by TIAB Screening | Berndtsson ID, E. Pennbrant, S. Work-integrated learning as a pedagogical tool to integrate theory and practice in nursing education - An integrative literature review. Nurse Education in Practice. 2020;42:102685. | |
| 241 | Rejected by TIAB Screening | Bernstein E, Podoltsev NA, Lee A. Teaching hematology to fellows: A qualitative study. Blood Conference: 59th Annual Meeting of the American Society of Hematology, ASH. 2017;130(Supplement 1). | |
| 242 | Formal Teaching | Berube B. Royal College's CME initiative focuses on lifelong, practice-integrated learning. Cmaj. 1995;152(6):965-8. | |
| 243 | Rejected by TIAB Screening | Bezerra ELM, Vilar MJ, Azevedo GD. Elective rheumatology program with a primary health care focus. Arthritis and Rheumatism. 2009;10):1355. | |
| 244 | Rejected by TIAB Screening | Bhagat VH, M. Bin Abu Bakar, Y. I. Husain, R. Khairi, C. M. Emotional maturity of medical students impacting their adult learning skills in a newly established public medical school at the east coast of Malaysian Peninsula. Advances in Medical Education & Practice. 2016;7:575-84. | |
| 245 | Rejected by TIAB Screening | Bharamgoudar RS, A. Twelve tips for medical students to make the best use of ward-based learning. Medical Teacher. 2017;39(11):1119-22. | |
| 246 | Rejected by TIAB Screening | Bharathan R, Meritte S, Ahmed H. Laparoscopic management of tubal pregnancy: How effective is the training in the UK? Gynecological Surgery. 2010;7:S210. | |
| 247 | Rejected by TIAB Screening | Bharj KKE, M. Factors affecting quality of midwifery students learning in the workplace: Results of two ICM congress workshops. Midwifery. 2018;62:116-8. | |
| 248 | Rejected by TIAB Screening | Bhusnurmath S, Bhusnurmath B, Goyal S, Hafeez S, Abugroun A, Okpe J. Concept map as an adjunct tool to teach pathology. Indian Journal of Pathology and Microbiology. 2017;60(2):226-31. | |
| 249 | Rejected by TIAB Screening | Bientzle MC, U. Kimmerle, J. Development of domain-specific epistemological beliefs of physiotherapists: a longitudinal study. BMC Medical Education. 2019;19(1):401. | |
| 250 | Rejected by TIAB Screening | Bijol VB-D, C. J. Hoenig, M. P. Medical student web-based formative assessment tool for renal pathology. Medical Education Online. 2015;20:26765. | |
| 251 | Rejected by TIAB Screening | Bikker R, Meyer K, Domberg P, Brand K, Behrends M. Development and evaluation of point-of-care testing recertification with e-learning. Scandinavian Journal of Clinical and Laboratory Investigation. 2020;80(2):133-8. | |
| 252 | Rejected by TIAB Screening | Billett S, Choy S. Learning through Work: Emerging Perspectives and New Challenges. Journal of Workplace Learning. 2013;25(4):264-76. | |
| 253 | Rejected by TIAB Screening | Billett SR. Securing intersubjectivity through interprofessional workplace learning experiences. Journal of Interprofessional Care. 2014;28(3):206-11. | |
| 254 | Rejected by TIAB Screening | Billings DM. Designing nursing learning centers of the future. Computers in nursing. 1996;14(2):80-1, 7. | |
| 255 | Rejected by TIAB Screening | Billings DMW, J. W. Penton-Cooper, L. Distance learning in nursing. Seminars in Oncology Nursing. 2001;17(1):48-54. | |
| 256 | Rejected by TIAB Screening | Binda S, Erler D, Campbell M. Monaco Super User: An Agent of Change in Ensuring The Readiness of MRI Guided Radiation Therapy. Journal of Medical Imaging and Radiation Sciences. 2019;50 (2 Supplement):S17. | |
| 257 | Rejected by TIAB Screening | Bindon SL. Professional Development Strategies to Enhance Nurses' Knowledge and Maintain Safe Practice. AORN journal. 2017;106(2):99-110. | |
| 258 | Rejected by TIAB Screening | Binkley JL. Adventures in intersectionality: The journey of an early career psychologist. Radical psychology: Multicultural and social justice decolonization initiatives. Lanham, MD: Lexington Books/Rowman & Littlefield; US; 2018. p. 151-71. | |
| 259 | Rejected by TIAB Screening | Birch DW, Eady A, Robertson D, De Pauw S, Tandan V. Users' guide to the surgical literature: How to perform a literature search. Canadian Journal of Surgery. 2003;46(2):136-41. | |
| 260 | Rejected by TIAB Screening | Bishop TW, Gorniewicz J, Floyd M, Tudiver F, Odom A, Zoppi K. Innovative patient-centered skills training addressing challenging issues in cancer communications: Using patient's stories that teach. International Journal of Psychiatry in Medicine. 2016;51(4):357-66. | |
| 261 | Rejected by TIAB Screening | Bisset GS, 3rd, Bresolin LB. RSNA support for lifelong learning. Radiographics. 2009;29(2):623-4. | |
| 262 | Rejected by TIAB Screening | Bister-Set P, Hughes J, Bister D, Smith C, Curley A. Learning preference of postgraduate medical trainees in the teaching of practical neonatal US. Pediatric Radiology. 2013;43:S642. | |
| 263 | Rejected by TIAB Screening | Bitzer EM, Sporhase U. [Health Literacy and patient education in medical rehabilitation]. Bundesgesundheitsblatt, Gesundheitsforschung, Gesundheitsschutz. 2015;58(9):983-8. | |
| 264 | Rejected by TIAB Screening | Black-Schaffer RM, Kirsteins AE, Harvey RL. Stroke rehabilitation. 2. Co-morbidities and complications. Archives of Physical Medicine and Rehabilitation. 1999;80(5 SUPPL.):S8-S16. | |
| 265 | Outside SDL | Blaka G, Filstad C. How Does a Newcomer Construct Identity? A Socio-Cultural Approach to Workplace Learning. International Journal of Lifelong Education. 2007;26(1):59-73. | |
| 266 | Rejected by TIAB Screening | Blake H, Gartshore E. Workplace wellness using online learning tools in a healthcare setting. Nurse Education in Practice. 2016;20:70-5. | |
| 267 | Outside SDL | Blatchley ME, et al. Effects of Self-Study on Achievement in a Medical-Surgical Nursing Course. Nursing Outlook. 1978;26(7):444-7. | |
| 268 | Rejected by TIAB Screening | Blenkinsopp J. Regional Framework for Self Paced Learning Modules. South Fraser Health Region. Guides - Non-Classroom. La Grange Area Dept. of Special Education, IL.; 1997. | |
| 269 | Rejected by TIAB Screening | Bligh J. Independent learning among general practice trainees: An initial survey. Medical Education. 1992;26(6):497-502. | |
| 270 | Rejected by TIAB Screening | Block J, Lerwick P. Educational preferences among residents in the ICU. Critical Care Medicine Conference: 48th Critical Care Congress of the Society of Critical Care Medicine, SCCM. 2019;47(1 Supplement 1). | |
| 271 | Rejected by TIAB Screening | Blouin D. Impact of interpersonal relations on learning and development of professional identity: A study of residents' perceptions. Emergency Medicine Australasia. 2018;30(3):398-405. | |
| 272 | Rejected by TIAB Screening | Bluhm S. The development of a new educational logbook. Anaesthesia. 2019;74 (Supplement 2):68. | |
| 273 | Rejected by TIAB Screening | Blumberg PD, Steven. Good Student or Good Physician: What Are We Encouraging? Speeches/Meeting Papers Reports - Descriptive. 1989. | |
| 274 | Rejected by TIAB Screening | Boet S, Bould MD, Schaeffer R, Fischhof S, Stojeba N, Naik VN, et al. Learning fibreoptic intubation with a virtual computer program transfers to 'hands on' improvement. European Journal of Anaesthesiology. 2010;27(1):31-5. | |
| 275 | Rejected by TIAB Screening | Bok HG, Jaarsma DA, Spruijt A, Van Beukelen P, Van Der Vleuten CP, Teunissen PW. Feedback-giving behaviour in performance evaluations during clinical clerkships. Medical Teacher. 2016;38(1):88-95. | |
| 276 | Rejected by TIAB Screening | Bokma JP, Daily JA, Kovacs AH, Oechslin EN, Baumgartner H, Khairy P, et al. Learning strategies among adult CHD fellows. Cardiology in the Young. 2019;29(11):1356-60. | |
| 277 | Rejected by TIAB Screening | Bolding DJ. Factors influencing role behaviors by professional exemplars in hospitals. Dissertation Abstracts International Section A: Humanities and Social Sciences. 2014;75(2-A(E)):No Pagination Specified. | |
| 278 | Rejected by TIAB Screening | Bollington L, John DN. What barriers exist to increasing hospital preregistration pharmacist training places? International Journal of Pharmacy Practice. 2009;17 (S2):B21-B2. | |
| 279 | Rejected by TIAB Screening | Bondevik GTH, Lone Haugland, Mildrid Baerheim, Anders Raaheim, Arild. Interprofessional Workplace Learning in Primary Care: Students from Different Health Professions Work in Teams in Real-Life Settings. International Journal of Teaching and Learning in Higher Education. 2015;27(2):175-82. | |
| 280 | Rejected by TIAB Screening | Boninger M, Troen P, Green E, Borkan J, Lance-Jones C, Humphrey A, et al. Implementation of a longitudinal mentored scholarly project: an approach at two medical schools. Academic Medicine. 2010;85(3):429-37. | |
| 281 | Rejected by TIAB Screening | Bonnel WB, Starling CK, Wambach KA, Tarnow K. Blended roles: preparing the advanced practice nurse educator/clinician with a Web-based nurse educator certificate program. Journal of Professional Nursing. 2003;19(6):347-53. | |
| 282 | Rejected by TIAB Screening | Bonsaksen T. Factors Associated with Occupational Therapy Students&apos; Preferences for Courses and Teaching. Cogent Education. 2018;5(1). | |
| 283 | Outside SDL | Borduas FG, Robert Laprise, Rejean Lacoursiere, Yves. The Longitudinal Case Study: From Schon's Model to Self-Directed Learning. Journal of Continuing Education in the Health Professions. 2001;21(2):103-09. | |
| 284 | Rejected by TIAB Screening | Boreham NC. A theoretical framework for improving education in geriatric medicine. Age & Ageing. 1983;Suppl:46-50. | |
| 285 | Rejected by TIAB Screening | Borem P, de Cassia Sanchez R, Torres J, Delgado P, Petenate AJ, Peres D, et al. A Quality Improvement Initiative to Increase the Frequency of Vaginal Delivery in Brazilian Hospitals. Obstetrics and gynecology. 2020;135(2):415-25. | |
| 286 | Outside SDL | Borg K, Hagstrom H. [Physicians' lifelong learning]. Lakartidningen. 2007;104(38):2701-2. | |
| 287 | Rejected by TIAB Screening | Borgfeld JK. A registered nurse refresher course: serving the community. Journal of Continuing Education in Nursing. 2014;45(2):77-82. | |
| 288 | Rejected by TIAB Screening | Borsky AE, Flores EJ, Berliner E, Chang C, Umscheid CA, Chang SM. Next steps in improving healthcare value: AHRQ evidence-based practice center program-applying the knowledge to practice to data cycle to strengthen the value of patient care. Journal of Hospital Medicine. 2019;14(5):311-4. | |
| 289 | Rejected by TIAB Screening | Borum ML. Introducing gastroenterology fellows to research utilizing autonomous learning theory: Impact upon scholarly activity and clinical skills. Gastroenterology. 2017;152 (5 Supplement 1):S228. | |
| 290 | Rejected by TIAB Screening | Bosma J, Laszakovits D, Hattery RR. Self-assessment for maintenance of certification. Journal of the American College of Radiology. 2007;4(1):45-52. | |
| 291 | Rejected by TIAB Screening | Bosmans H, Bliznakova K, Padovani R, Christofides S, Van Peteghem N, Tsapaki V, et al. EUTEMPE-RX: A new ec supported course for medical physics experts in radiology. Physica Medica. 2014;1):e11. | |
| 292 | Rejected by TIAB Screening | Bostrom A-M, Sommerfeld DK, Stenhols AW, Kiessling A. Capability beliefs on, and use of evidence-based practice among four health professional and student groups in geriatric care: A cross sectional study. PLoS ONE Vol 13(2), 2018, ArtID e0192017. 2018;13(2). | |
| 293 | Rejected by TIAB Screening | Bostrom AM, Sommerfeld DK, Stenhols AW, Kiessling A. Capability beliefs on, and use of evidence-based practice among four health professional and student groups in geriatric care: A cross sectional study. PLoS ONE [Electronic Resource]. 2018;13(2):e0192017. | |
| 294 | Rejected by TIAB Screening | Boucouvalas MA, Mejai. Proceedings of the Commission for International Adult Education (CIAE) International Pre-Conference (64th, Oklahoma City, Oklahoma, November 15-17, 2015). Commission for International Adult Education. 2015. | |
| 295 | Rejected by TIAB Screening | Bound HL, Magdalene. Developing Competence at Work. Vocations and Learning. 2013;6(3):403-20. | |
| 296 | Rejected by TIAB Screening | Bourgeois F, Simons W, Olson K, Brownstein J, Mandl K. Evaluation of influenza prevention in the workplace using a personally controlled health record: randomized controlled trial. Journal of medical Internet research [Internet]. 2008; 10(1):[e5 p.]. Available from: https://www.cochranelibrary.com/central/doi/10.1002/central/CN-00630828/full. | |
| 297 | Formal Teaching | Bourgeois JA. Continuum: Lifelong Learning in Neurology - Introduction. CONTINUUM Lifelong Learning in Neurology. 2006;12(5):13-4. | |
| 298 | Rejected by TIAB Screening | Bourner TK, Tim Watson, David, editor. New Directions in Professional Higher Education2000. | |
| 299 | Rejected by TIAB Screening | Bowden SC. Key skills for the evidence-based practitioner. Neuropsychological assessment in the age of evidence-based practice: Diagnostic and treatment evaluations. New York, NY: Oxford University Press; US; 2017. p. 281-8. | |
| 300 | Rejected by TIAB Screening | Bowen JL. Adapting residency training. Training adaptable residents. Western Journal of Medicine. 1998;168(5):371-7. | |
| 301 | Rejected by TIAB Screening | Bowen L, Shaw A, Lyttle MD, Purdy S. The transition to clinical expert: enhanced decision making for children aged less than 5 years attending the paediatric ED with acute respiratory conditions. Emergency Medicine Journal. 2017;34(2):76-81. | |
| 302 | Rejected by TIAB Screening | Bowen SI, Scott KM, Anderson JL, Holland AJA, Soundappan SVS, Gunasekera H. Improving paediatric surgery teaching and learning in a medical program. Journal of Paediatrics and Child Health. 2019;55 (Supplement 2):15-6. | |
| 303 | Rejected by TIAB Screening | Bowling JE. Impact of Repetitive Text Messages on Retention of Knowledge. Journal of Continuing Education in Nursing. 2019;50(9):399-403. | |
| 304 | Not Target Group | Bowman M. The transition to self-regulated learning for first-year dental students: threshold concepts. European Journal of Dental Education. 2017;21(3):142-50. | |
| 305 | Rejected by TIAB Screening | Boyd P. Academic Induction for Professional Educators: Supporting the Workplace Learning of Newly Appointed Lecturers in Teacher and Nurse Education. International Journal for Academic Development. 2010;15(2):155-65. | |
| 306 | Rejected by TIAB Screening | Boyd PS, Caroline Ilhan Beyaztas, Dilek. Evaluating Academic Workplaces: The Hyper-Expansive Environment Experienced by University Lecturers in Professional Fields. International Journal for Academic Development. 2015;20(1):18-32. | |
| 307 | Rejected by TIAB Screening | Bradley S, Egan JP, Henning M. Paediatric team handover: a time to learn? New Zealand Medical Journal. 2018;131(1476):70-80. | |
| 308 | Rejected by TIAB Screening | Braithwaite J, Westbrook MT, Iedema R, Mallock NA, Nugus P, Forsyth R, et al. Are health systems changing in support of patient safety?: A multi-methods evaluation of education, attitudes and practice. International Journal of Health Care Quality Assurance. 2007;20(7):585-601. | |
| 309 | Rejected by TIAB Screening | Branda LA. Implementing Problem-Based Learning. Journal of Dental Education. 1990;54(9):548-49. | |
| 310 | Rejected by TIAB Screening | Brandeis D, Holtmann M, Strehl U, Wachtlin D, Aggensteiner P, Feedback C. A randomized controlled multicentre study on the efficacy of slow cortical potential neurofeedback in ADHD. ADHD Attention Deficit and Hyperactivity Disorders. 2015;7:S9-S10. | |
| 311 | Rejected by TIAB Screening | Brandt Jr EN. Continuing education/beyond higher education. Bulletin of the Medical Library Association. 1990;78(2):157-60. | |
| 312 | Not Target Group | Branitzki-Heinemann K, Brogden G, Von Kckritz-Blickwede M, Naim H. The Vetvip consortium of the EU-Lifelong Learning Programme presents: "Death by Broken Heart". FASEB Journal Conference: Experimental Biology. 2014;28(1 SUPPL. 1). | |
| 313 | Formal Teaching | Bransen D, Govaerts MJB, Sluijsmans DMA, Driessen EW. Beyond the self: The role of co-regulation in medical students' self-regulated learning. Medical Education. 2020;54(3):234-41. | |
| 314 | Rejected by TIAB Screening | Branzetti J, Gisondi MA, Hopson LR, Regan L. Aiming Beyond Competent: The Application of the Taxonomy of Significant Learning to Medical Education. Teaching & Learning in Medicine. 2019;31(4):466-78. | |
| 315 | Formal Teaching | Bravata DM, Huot SJ, Abernathy HS, Skeff KM, Bravata DM. The development and implementation of a curriculum to improve clinicians' self-directed learning skills: a pilot project. BMC Medical Education. 2003;3:7. | |
| 316 | Rejected by TIAB Screening | Braymer MRS, Jennie D. Nontraditional Options for Continuing Education in the Health Professions: A Bibliography, 1970-1980. Reference Materials - Bibliographies. Virginia Commonwealth Univ., Richmond.; 1980. | |
| 317 | Rejected by TIAB Screening | Brazel A. Evolution of practice - The shape of things to come. Journal of Medical Radiation Sciences. 2015;62 (Supplement 1):80. | |
| 318 | Formal Teaching | Breckwoldt J, Gruber H. Will we continuously improve?. Lifelong learning. [German]. Notfall und Rettungsmedizin. 2012;15(3):207-12. | |
| 319 | Rejected by TIAB Screening | Brekelmans GP, Rob F. van Wijk, Kees. Factors Influencing Continuing Professional Development: A Delphi Study among Nursing Experts. European Journal of Training and Development. 2013;37(3):313-25. | |
| 320 | Rejected by TIAB Screening | Brent DA. Update on mood disorders, psychopharmacology, and prevention. Journal of the American Academy of Child and Adolescent Psychiatry. 2016;55 (10 Supplement 1):S91. | |
| 321 | Not Target Group | Bresolin L, McLoud TC, Becker GJ, Kwakwa F. Education techniques for lifelong learning: international variations in initial certification and maintenance of certification in radiology: a multinational survey. Radiographics. 2008;28(1):13-20. | |
| 322 | Rejected by TIAB Screening | Breuer G. [Training -- competency-based education -- learning theory and practice]. Anasthesiologie, Intensivmedizin, Notfallmedizin, Schmerztherapie. 2013;48(11-12):708-13. | |
| 323 | Not Target Group | Brewer CC, Don G. Rhythms of Learning: Creative Tools for Developing Lifelong Skills. Guides - Classroom - Teacher Guides - Non-Classroom. 1991. | |
| 324 | Rejected by TIAB Screening | Bridgemohan C, Levy S, Veluz A, Knight J. Teaching paediatric residents about learning disorders: use of standardised case discussion versus multimedia computer tutorial. Medical education [Internet]. 2005; 39(8):[797‐806 pp.]. Available from: https://www.cochranelibrary.com/central/doi/10.1002/central/CN-00523529/full. | |
| 325 | Rejected by TIAB Screening | Bridges J, Fuller A. Creating learning environments for compassionate care: a programme to promote compassionate care by health and social care teams. International Journal of Older People Nursing. 2015;10(1):48-58. | |
| 326 | Rejected by TIAB Screening | Bridges S. An Emic Lens into Online Learning Environments in PPL in Undergraduate Dentistry. Pedagogies: An International Journal. 2015;10(1):22-37. | |
| 327 | Rejected by TIAB Screening | Brier N. Self-regulated learning: Practical interventions for struggling teens. Champaign, IL: Research Press; US; 2010. | |
| 328 | Rejected by TIAB Screening | Brigley S. Continuing education for medical professionals: A reflective model. Postgraduate Medical Journal. 1997;73(855):23-6. | |
| 329 | Rejected by TIAB Screening | Briscoe GS, Brown LG. Self-Regulated E-Learning Modules for Prenursing Success. Nursing Education Perspectives. 2019;40(3):186-8. | |
| 330 | Rejected by TIAB Screening | Britton CV. Ensuring culturally effective pediatric care: implications for education and health policy. Pediatrics. 2004;114(6):1677-85. | |
| 331 | Rejected by TIAB Screening | Britton CV, American Academy of Pediatrics Committee on Pediatric W. Ensuring culturally effective pediatric care: implications for education and health policy. Pediatrics. 2004;114(6):1677-85. | |
| 332 | Not Target Group | Brockett RGS, Susan L. Fogerson, Dewey L. Cox, Barry F. Canipe, James B. Chuprina, Larissa A. Donaghy, Robert C. Chadwell, Nancy E. Two Decades of Literature on Self-Directed Learning: A Content Analysis. Information Analyses Reports - Research Speeches/Meeting Papers. 2000. | |
| 333 | Rejected by TIAB Screening | Brocklehurst PR. Personal construct psychology: a theory to help understand professional development, a philosophy to support it. Primary Dental Care. 2010;17(4):179-87. | |
| 334 | Outside SDL | Bromley P, Ehrenreich D. The journey of lifelong learning. American Journal of Nursing. 2001;101(1):73-4. | |
| 335 | Rejected by TIAB Screening | Brooks WSW, Kristina T. C. Panizzi Jackson, James R. Hoesley, Craig J. Integration of Gross Anatomy in an Organ System-Based Medical Curriculum: Strategies and Challenges. Anatomical Sciences Education. 2015;8(3):266-74. | |
| 336 | Rejected by TIAB Screening | Brose L. Involving Native Nursing Students in Their Own Learning. Reports - Descriptive. 1988. | |
| 337 | Rejected by TIAB Screening | Brotman LM, Dawson-McClure S, Calzada EJ, Huang KY, Kamboukos D, Palamar JJ, et al. Cluster (school) RCT of parentcorps: Impact on kindergarten academic achievement. Pediatrics. 2013;131(5):e1521-e9. | |
| 338 | Rejected by TIAB Screening | Brown B, Holt-Macey S, Martin B, Skau K, Vogt EM. Developing the reflective practitioner: What, so what, now what. Currents in Pharmacy Teaching and Learning. 2015;7(5):705-15. | |
| 339 | Rejected by TIAB Screening | Brown BL. A New Focus for Allied Health Occupations. Trends and Issues Alerts. ERIC Publications Reference Materials - Bibliographies. ERIC Clearinghouse on Adult, Career, and Vocational Education, Columbus, OH.; 1997. Report No.: RR93002001. | |
| 340 | Rejected by TIAB Screening | Brown MT, Zhang G. Gaining competencies during early medical training: Medical students as teaching assistants in dissection-based anatomy course. FASEB Journal Conference: Experimental Biology. 2016;30(Meeting Abstracts). | |
| 341 | Rejected by TIAB Screening | Brown TCea. Experiences with a Competency-Based Educational Approach to Reproductive Biology. Speeches/Meeting Papers. Utah Univ., Salt Lake City.; 1974. | |
| 342 | Rejected by TIAB Screening | Brown WE, Jr. Dotterer, Robert W. Gainor, Dia Judd, Richard L. Larmon, Baxter Lewis, Kathryn M. Margolis, Gregg S. Mercer, Steve Mistovich, Joseph J. Newell, Lawrence D. Politis, Jonathan F. Stoy, Walt A. Stupar, James A. Walz, Bruce J. Wagoner, Robert. EMT-Paramedic and EMT-Intermediate Continuing Education. National Guidelines. Guides - Classroom - Teacher Legal/Legislative/Regulatory Materials. National Highway Traffic Safety Administration (DOT), Washington, DC., Health Resources and Services Administration (DHHS/PHS), Washington, DC. Maternal and Child Health Bureau.; 1999. | |
| 343 | Rejected by TIAB Screening | Brownrigg N, Braga L, Rickard M, Farrokhyar F, Easterbrook B, Dekirmendjian A, et al. The impact of a bladder training video versus standard urotherapy on quality of life of children with bladder and bowel dysfunction: a randomized controlled trial. Journal of pediatric urology [Internet]. 2017; 13(4):[374.e1‐.e8 pp.]. Available from: https://www.cochranelibrary.com/central/doi/10.1002/central/CN-01615670/full. | |
| 344 | Rejected by TIAB Screening | Brueggeman PM. Applying adult learning principles to supervision. Seminars in Hearing. 2006;27(2):86-91. | |
| 345 | Rejected by TIAB Screening | Brugnolli A, Benaglio C. [Teaching methods for clinical settings: a literature review]. Assistenza Infermieristica e Ricerca:Air. 2017;36(1):14-23. | |
| 346 | Not Target Group | Brunell J. Nursing motivations for professional lifelong education: Students/graduate nurses from an AD and RN to BSN program. Dissertation Abstracts International Section A: Humanities and Social Sciences. 2008;68(11-A):4575. | |
| 347 | Rejected by TIAB Screening | Bruno TO, Hicks CB, Naggie S, Wohl DA, Albrecht H, Thielman NM, et al. VISION: a regional performance improvement initiative for HIV health care providers. The Journal of continuing education in the health professions. 2014;34(3):171-8. | |
| 348 | Rejected by TIAB Screening | Brunton PA, Morrow LA, Hoad-Reddick G, McCord JF, Wilson NH. Students' perceptions of seminar and lecture-based teaching in restorative dentistry. European Journal of Dental Education. 2000;4(3):108-11. | |
| 349 | Rejected by TIAB Screening | Bryan J, Lindsay H. The Dunning-Kruger effect in medical education: Double trouble for the learner in difficulty. Canadian Journal of Emergency Medicine. 2017;19 (Supplement 1):S86. | |
| 350 | Accepted | Brydges R, Butler D. A reflective analysis of medical education research on self-regulation in learning and practice. Medical Education. 2012;46(1):71-9. | |
| 351 | Not Target Group | Brydges R, Carnahan H, Rose D, Dubrowski A. Comparing self-guided learning and educator-guided learning formats for simulation-based clinical training. Journal of Advanced Nursing. 2010;66(8):1832-44. | |
| 352 | Rejected by TIAB Screening | Brydges R, Carnahan H, Rose D, Rose L, Dubrowski A. Coordinating progressive levels of simulation fidelity to maximize educational benefit. Academic medicine [Internet]. 2010; 85(5):[806‐12 pp.]. Available from: https://www.cochranelibrary.com/central/doi/10.1002/central/CN-00751652/full. | |
| 353 | Rejected by TIAB Screening | Bryson EM, Dale R. Library Research Manual: The Health Sciences. Guides - Classroom - Learner. Chicago State Univ., IL.; 1981. | |
| 354 | Rejected by TIAB Screening | Bube S, Konge L, Hansen RB. Simulation-based training for flexible cystoscopy-A patient transfer randomized trial. Scandinavian Journal of Urology. 2017;51 (220):36. | |
| 355 | Not Target Group | Buchmann RF, Greenberg SB. RadioGraphics: residency training to lifelong learning. Radiographics. 2009;29(2):625-6. | |
| 356 | Rejected by TIAB Screening | Buckley JD, Addrizzo-Harris DJ, Clay AS, Curtis JR, Kotloff RM, Lorin SM, et al. Multisociety task force recommendations of competencies in pulmonary and critical care medicine. American Journal of Respiratory and Critical Care Medicine. 2009;180(4):290-5. | |
| 357 | Rejected by TIAB Screening | Buckley PF, Madaan V. Leadership and Professional Workforce Development. Psychiatric Clinics of North America. 2008;31(1):105-22. | |
| 358 | Rejected by TIAB Screening | Bugaj TJ, Nikendei C, Groener JB, Stiepak J, Huber J, Moltner A, et al. Ready to run the wards? - A descriptive follow-up study assessing future doctors' clinical skills. BMC medical education. 2018;18(1):257. | |
| 359 | Rejected by TIAB Screening | Bugaj TJ, Schmid C, Koechel A, Stiepak J, Groener JB, Herzog W, et al. Shedding light into the black box: A prospective longitudinal study identifying the CanMEDS roles of final year medical students' on-ward activities. Medical Teacher. 2017;39(8):883-90. | |
| 360 | Rejected by TIAB Screening | Bullard B, Lawless L, Williams M, Bergstrom D. Mental Health Counselor's Clinical Practice Companion: A Continuing Education Program1999. | |
| 361 | Rejected by TIAB Screening | Bullen M, Janes D. Making the Transition to E-Learning: Strategies and Issues. Information Science Publishing. 2007. | |
| 362 | Rejected by TIAB Screening | Bullock A. Does technology help doctors to access, use and share knowledge? Medical Education. 2014;48(1):28-33. | |
| 363 | Rejected by TIAB Screening | Bullock A, Firmstone V, Fielding A, Frame J, Thomas D, Belfield C. Participation of UK dentists in continuing professional development. British Dental Journal. 2003;194(1):47-51. | |
| 364 | Rejected by TIAB Screening | Bulmer P, James M, Ellis-Jones J, Smith D, Timoney A, Donovan J. A randomized trial comparing the effectiveness and preference of a touch-screen computer system with a leaflet for providing women with information on urinary symptoms suggestive of detrusor instability. BJU international [Internet]. 2001; 88(6):[532‐5 pp.]. Available from: https://www.cochranelibrary.com/central/doi/10.1002/central/CN-00369397/full. | |
| 365 | Outside SDL | Bunkers SS. Lifelong learning: a human becoming perspective. Nursing Science Quarterly. 2002;15(4):294-300. | |
| 366 | Rejected by TIAB Screening | Burford B, Morrow G, Morrison J, Baldauf B, Spencer J, Johnson N, et al. Newly qualified doctors' perceptions of informal learning from nurses: implications for interprofessional education and practice. Journal of Interprofessional Care. 2013;27(5):394-400. | |
| 367 | Rejected by TIAB Screening | Burg G, French LE. [The age of Gutenberg is over: a consideration of medical education--past, present and future]. Hautarzt. 2012;63 Suppl 1:38-44. | |
| 368 | Rejected by TIAB Screening | Burgess A, Matar E, Neuen B, Fox GJ. A longitudinal faculty development program: supporting a culture of teaching. BMC medical education. 2019;19(1):400. | |
| 369 | Rejected by TIAB Screening | Burhenn PS, Ferrell B, Johnson S, Hurria A. Improving Nurses' Knowledge About Older Adults With Cancer. Oncology Nursing Forum. 2016;43(4):497-504. | |
| 370 | Rejected by TIAB Screening | Burke AE, Benson B, Englander R, Carraccio C, Hicks PJ. Domain of competence: Practice-based learning and improvement. Academic Pediatrics. 2014;14(2 SUPPL.):s38-s54. | |
| 371 | Rejected by TIAB Screening | Burke C, Masters DE, O'Sullivan P, Sheu L. The role of third year clerkship students and the potential for change. Journal of General Internal Medicine. 2017;32 (2 Supplement 1):S350. | |
| 372 | Rejected by TIAB Screening | Burke GC, McGough SA, Kostroun PW. Renewal and change for clinical laboratory managers. Clinical Laboratory Management Review. 1993;7(1):43-8. | |
| 373 | Rejected by TIAB Screening | Burke LS. Tutor Training: An Independent Learning Approach. Adult Literacy Independent Learning Packet. Guides - Non-Classroom. Lehigh Univ., Bethlehem, PA. Tri-Valley Literacy Staff Development Center Region 7.; 1993. | |
| 374 | Rejected by TIAB Screening | Burm E, Choi AS, Gu JA, Kim HG, Lee E, Yee O. The effects of applying havruta learning method in nursing classes. Indian Journal of Public Health Research and Development. 2019;10(11):4475-80. | |
| 375 | Formal Teaching | Bustamante-Balen M, Satorres C, Puchades L, Navarro B, Garcia-Morales N, Alonso N, et al. Non-guided self-learning program for high-proficiency optical diagnosis of diminutive and small colorectal lesions: A single-endoscopist pilot study. World Journal of Gastroenterology. 2019;25(10):1278-88. | |
| 376 | Outside SDL | Butcher D, Bruce A. Nurses and Lifelong Learning: Creating "Makers and Shapers" or "Users and Choosers"? Nursing Forum. 2016;51(2):97-104. | |
| 377 | Rejected by TIAB Screening | Butter J, McGaghie W, Cohen E, Kaye M, Wayne D. Simulation-based mastery learning improves cardiac auscultation skills in medical students. Journal of general internal medicine [Internet]. 2010; 25(8):[780‐5 pp.]. Available from: https://www.cochranelibrary.com/central/doi/10.1002/central/CN-00780046/full. | |
| 378 | Rejected by TIAB Screening | Bye AM, Connolly AM, Netherton C, Looker P, Burgess A, Lonergan A. A triangulated approach to the assessment of teaching in childhood epilepsy. Medical Teacher. 2007;29(2-3):255-7. | |
| 379 | Outside SDL | Byrick RJ. Professional self-regulation: learning from the disciplinary process. Canadian Journal of Anaesthesia. 2013;60(10):960-5. | |
| 380 | Rejected by TIAB Screening | Byrne BJ, Frintner MP, Abraham HN, Starmer AJ. Attitudes and Experiences of Early and Midcareer Pediatricians With the Maintenance of Certification Process. Academic pediatrics. 2017;17(5):487-96. | |
| 381 | Rejected by TIAB Screening | Cabaniss DL, Havel LK, Berger S, Deo A, Arbuckle MR. The microprocess moment: A tool for evaluating skills in psychodynamic psychotherapy. Academic Psychiatry. 2017;41(1):51-4. | |
| 382 | Rejected by TIAB Screening | Cadorin L, Grassetti L, Paoletti E, Cara A, Truccolo I, Palese A. Evaluating self-directed learning abilities as a prerequisite of health literacy among older people: Findings from a validation and a cross-sectional study. International Journal of Older People Nursing. 2020;15(1):e12282. | |
| 383 | Not Target Group | Cadorin L, Rei A, Dante A, Bulfone T, Viera G, Palese A. Enhancing self-directed learning among Italian nursing students: A pre- and post-intervention study. Nurse Education Today. 2015;35(6):746-53. | |
| 384 | Formal Teaching | Cadorin L, Suter N, Saiani L, Williamson SN, Palese A. Self-Rating Scale of Self-Directed Learning (SRSSDL): Preliminary results from the Italian validation process. Journal of Research in Nursing. 2011;16(4):363-73. | |
| 385 | Rejected by TIAB Screening | Caldwell BJ, Carter EMA. The Return of the Mentor: Strategies for Workplace Learning. Education Policy Perspectives Series1993. | |
| 386 | Rejected by TIAB Screening | Calpin-Davies PJ. Management and Leadership: A Dual Role in Nursing Education. Nurse Education Today. 2003;23(1):3-10. | |
| 387 | Rejected by TIAB Screening | Cameron M, Ray R, Sabesan S. Remote supervision of medical training via videoconference in northern Australia: A qualitative study of the perspectives of supervisors and trainees. BMJ Open. 2015;5(3):1-10. | |
| 388 | Rejected by TIAB Screening | Campbell B, Mackay G. Continuing competence: an Ontario nursing regulatory program that supports nurses and employers. Nursing Administration Quarterly. 2001;25(2):22-30. | |
| 389 | Rejected by TIAB Screening | Campbell C, Parboosingh J, Gondocz T, Babitskaya G, Pham B. A study of the factors that influence physicians' commitments to change their practices using learning diaries. Academic Medicine. 1999;74(Suppl 10):S34-S6. | |
| 390 | Rejected by TIAB Screening | Campbell C, Silver I, Sherbino J, Ten Cate O, Holmboe ES. Competency-based continuing professional development. Medical Teacher. 2010;32(8):657-62. | |
| 391 | Rejected by TIAB Screening | Campbell M, Bernhardt J, Waldmiller M, Jackson B, Potenziani D, Weathers B, et al. Varying the message source in computer-tailored nutrition education. Patient education and counseling [Internet]. 1999; 36(2):[157‐69 pp.]. Available from: https://www.cochranelibrary.com/central/doi/10.1002/central/CN-00413566/full. | |
| 392 | Rejected by TIAB Screening | Campbell MD. Mandatory Continuing Professional Education: Help or Hindrance to Quality Education? New Directions for Continuing Education. 1982;22. | |
| 393 | Rejected by TIAB Screening | Campos LR, Ribeiro MR, Depes VB. [Autonomy of nursing undergraduate student in the (re)construction of knowledge mediated by problem-based learning]. Revista Brasileira de Enfermagem. 2014;67(5):818-24. | |
| 394 | Rejected by TIAB Screening | Cannella AC, Kissin EY, Kaeley GS, Higgs JB, Caverzagie KJ, Torralba KD. Preliminary entrustable professional activities in muscloskeletal ultrasound for rheumatology fellowship training. Arthritis and Rheumatism. 2013;65:S1205-S6. | |
| 395 | Formal Teaching | Canning R. Older Workers in the Hospitality Industry: Valuing Experience and Informal Learning. International Journal of Lifelong Education. 2011;30(5):667-79. | |
| 396 | Rejected by TIAB Screening | Cantillon P. Workplace learning: the influences of culture, power and organisation on becoming a doctor. Education for Primary Care. 2016;27(6):507-8. | |
| 397 | Rejected by TIAB Screening | Cantwell Sean R, Bonadurer GF, Pawlina W, Lachman N. A near peer-driven dissection selective: Primer to a medical school anatomy course. Clinical Anatomy. 2016;29 (1):81. | |
| 398 | Formal Teaching | Canty D, Barth J, Yang Y, Peters N, Palmer A, Royse A, et al. Comparison of learning outcomes for teaching focused cardiac ultrasound to physicians: A supervised human model course versus an eLearning guided self- directed simulator course. Journal of Critical Care. 2019;49:38-44. | |
| 399 | Rejected by TIAB Screening | Caporiccio J, Louis KR, Lewis-O'Connor A, Son KQ, Raymond N, Garcia-Rodriguez IA, et al. Continuing Education for Haitian Nurses: Evidence from Qualitative and Quantitative Inquiry. Annals of Global Health. 2019;85(1):01. | |
| 400 | Rejected by TIAB Screening | Carbonne B, Sabri-Kaci I. Assessment of an e-learning training program for cardiotocography analysis: a multicentre randomized study. European journal of obstetrics, gynecology, and reproductive biology [Internet]. 2016; 197:[111‐5 pp.]. Available from: https://www.cochranelibrary.com/central/doi/10.1002/central/CN-01214904/full. | |
| 401 | Formal Teaching | Carcich G, Rafti K. Experienced registered nurses' satisfaction with using self-learning modules versus traditional lecture/discussion to achieve competency goals during hospital orientation. Journal for nurses in staff development [Internet]. 2007; 23(5):[214‐20; quiz 21‐2 pp.]. Available from: https://www.cochranelibrary.com/central/doi/10.1002/central/CN-00619817/full. | |
| 402 | Rejected by TIAB Screening | Cardiff LM, Lum EPM, Mitchell C, Nissen LM, Patounas MP, McBride LJ. Teaching the principles of safe prescribing to a mixed profession postgraduate cohort: Program development. Journal of Multidisciplinary Healthcare. 2018;11:635-44. | |
| 403 | Rejected by TIAB Screening | Cardiff RD. Teaching problem solving in pathology. General Professional Education of Physicians (GPEP) anticipated. Archives of Pathology & Laboratory Medicine. 1986;110(9):780-3. | |
| 404 | Rejected by TIAB Screening | Carlsen T, Bratland SZ, Claudi T, Cooper J, Telje J, Waaler HM, et al. [Efficient learning with data from own practice--experiences from the SATS-project]. Tidsskrift for Den Norske Laegeforening. 1999;119(29):4306-9. | |
| 405 | Rejected by TIAB Screening | Carlton KH. Redefining continuing education delivery. Computers in Nursing. 1997;15(1):17-8, 22. | |
| 406 | Rejected by TIAB Screening | Carnall L. Developing student autonomy in education: The Independent Option. The British Journal of Occupational Therapy. 1998;61(12):551-5. | |
| 407 | Rejected by TIAB Screening | Carney P, Abraham L, Cook A, Feig S, Sickles E, Miglioretti D, et al. Impact of an educational intervention designed to reduce unnecessary recall during screening mammography. Academic radiology [Internet]. 2012; 19(9):[1114‐20 pp.]. Available from: https://www.cochranelibrary.com/central/doi/10.1002/central/CN-00854017/full. | |
| 408 | Rejected by TIAB Screening | Carney PA, Allison KH, Oster NV, Frederick PD, Morgan TR, Geller BM, et al. Identifying and processing the gap between perceived and actual agreement in breast pathology interpretation. Modern Pathology. 2016;29(7):717-26. | |
| 409 | Rejected by TIAB Screening | Carney PA, Frederick PD, Reisch LM, Titus L, Knezevich SR, Weinstock MA, et al. Complexities of perceived and actual performance in pathology interpretation: A comparison of cutaneous melanocytic skin and breast interpretations. Journal of cutaneous pathology. 2018;45(7):478-90. | |
| 410 | Rejected by TIAB Screening | Carr EC. "Effects of performance feedback on patient pain outcomes": Commentary. Clinical Nursing Research. 2000;9(4):398-401. | |
| 411 | Rejected by TIAB Screening | Carr P, Mahmood S, Lastrilla G, Kee K. Training the "Untrained" making improvements in stroke rehabilitation. Cerebrovascular Diseases. 2014;37 (Supplement 1):318. | |
| 412 | Rejected by TIAB Screening | Carroll V. Learning is fundamental. Journal of Neuroscience Nursing. 2012;44(6):297. | |
| 413 | Rejected by TIAB Screening | Caruana CJ, Christofides S, Hartmann GH. European Federation of Organisations for Medical Physics (EFOMP) Policy Statement 12.1: Recommendations on Medical Physics Education and Training in Europe 2014. Physica Medica. 2014;30(6):598-603. | |
| 414 | Rejected by TIAB Screening | Carvalho IP, Ribeiro-Silva R, Pais VG, Figueiredo-Braga M, Castro-Vale I, Teles A, et al. Teaching doctor-patient communication: A proposal in practice. [Portuguese]. Acta Medica Portuguesa. 2010;23(3):527-32. | |
| 415 | Rejected by TIAB Screening | Casey B, Webb M. Imaging Journeys of Recovery and Learning: A Participatory Arts-Based Inquiry. Qualitative health research. 2019;29(6):833-45. | |
| 416 | Rejected by TIAB Screening | Cassiani SHB, Wilson LL, Mikael SSE, Pena LM, Grajales RAZ, McCreary LL, et al. The situation of nursing education in Latin America and the Caribbean towards universal health. Revista Latino-Americana de Enfermagem. 2017;25:e2913. | |
| 417 | Rejected by TIAB Screening | Cassiani SHDB, Wilson LL, Mikael SdSE, Moran Pena L, Zarate Grajales RA, McCreary LL, et al. The situation of nursing education in Latin America and the Caribbean towards universal health. Revista Latino-Americana de Enfermagem Vol 25 2017, ArtID e2913. 2017;25. | |
| 418 | Rejected by TIAB Screening | Castanelli DJ, Weller JM, Molloy E, Bearman M. Shadow systems in assessment: how supervisors make progress decisions in practice. Advances in health sciences education : theory and practice. 2020;25(1):131-47. | |
| 419 | Rejected by TIAB Screening | Castillo Contreras O, Soriano Alvarez C. [Continuing medical education in gastroenterology and recertification in Peru]. Revista de Gastroenterologia del Peru. 2017;37(3):279-86. | |
| 420 | Rejected by TIAB Screening | Castillo JDD. Effect of knowledge of learning styles on test score achievement of certified registered nurse anesthetists. Dissertation Abstracts International Section A: Humanities and Social Sciences. 2017;78(5-A(E)):No Pagination Specified. | |
| 421 | Rejected by TIAB Screening | Catton C. How to write a clinical paper. Journal of Medical Imaging and Radiation Oncology. 2013;57:54. | |
| 422 | Rejected by TIAB Screening | Celii A, Cross A. Are residents prepared to enter the "real world"?: Surveying both attending and resident viewpoints. Critical Care Medicine. 2014;42(12):A1438-A9. | |
| 423 | Rejected by TIAB Screening | Cervai S, Polo F. Evaluating the Quality of the Learning Outcome in Healthcare Sector: The Expero4care Model. Journal of Workplace Learning. 2015;27(8):611-26. | |
| 424 | Rejected by TIAB Screening | Chabeli M. Perceptions of postbasic nursing students in the use of seminars as a teaching method. Curationis. 1999;22(4):69-74. | |
| 425 | Rejected by TIAB Screening | Chae SJ. Analysis of Medical School Alumni's Perception on the Importance and Educational Needs of Outcomes. Korean Journal of Medical Education. 2010;22(2):113-20. | |
| 426 | Rejected by TIAB Screening | Chaghari M, Saffari M, Ebadi A, Ameryoun A. Empowering Education: A New Model for In-service Training of Nursing Staff. Journal of Advances in Medical Education & Professionalism. 2017;5(1):26-32. | |
| 427 | Rejected by TIAB Screening | Chakravarti A, Raazi M, O'Brien J, Balaton B. Anesthesiology Resident Wellness Program at the University of Saskatchewan: concept and development. Canadian Journal of Anesthesia. 2017;64(2):185-98. | |
| 428 | Accepted | Chakkaravarthy K, Ibrahim N, Mahmud M, Hardaker G, Ramasamy Venkatasalu M. Determinants of readiness towards self-directed learning among nurses and midwives: Results from national survey. Nurse Educ Pract. 2020;47:102824. | |
| 429 | Rejected by TIAB Screening | Chalmers H, Swallow VM, Miller J. Accredited Work-based Learning: An Approach for Collaboration between Higher Education and Practice. Nurse Education Today. 2001;21(8):597-606. | |
| 430 | Rejected by TIAB Screening | Chalmers JM, Robinson J, Nankivell N. The practical oral care video--evaluation of a dental awareness month initiative. Australian Dental Journal. 2005;50(2):75-80. | |
| 431 | Rejected by TIAB Screening | Chamberlain D, Hegney D, Harvey C, Knight B, Garrahy A, Tsai LPS. The factors influencing the effective early career and rapid transition to a nursing specialty in differing contexts of practice: A modified Delphi consensus study. BMJ Open. 2019;9(8). | |
| 432 | Outside SDL | Chan AKL. Evidence-based medicine for general practitioners - A tool for lifelong self-directed learning? Hong Kong Practitioner. 2004;26(9):400-7. | |
| 433 | Rejected by TIAB Screening | Chan SW, Chan MF, Lee SY, Henderson A. Retracted: Nurses learning in the workplace: a comparison of workplace attributes in acute care settings in Australia and Singapore. International Nursing Review. 2014;61(1):82-9. | |
| 434 | Rejected by TIAB Screening | Chang AY, Ghose S, Littman-Quinn R, Anolik RB, Kyer A, Mazhani L, et al. Use of mobile learning by resident physicians in Botswana. Telemedicine Journal & E-Health. 2012;18(1):11-3. | |
| 435 | Not Target Group | Chang SY, Tung IP, Chin K, Fraser RS, Razack S. Clinical relevance under the microscope: Using pathology to stimulate medical student motivation and self-regulated learning in histology. Laboratory Investigation. 2011;91:128A-9A. | |
| 436 | Rejected by TIAB Screening | Chao L, Enokihara M, Silveira P, Gomes S, Böhm G. Telemedicine model for training non-medical persons in the early recognition of melanoma. Journal of telemedicine and telecare [Internet]. 2003; 9 Suppl 1:[S4‐7 pp.]. Available from: https://www.cochranelibrary.com/central/doi/10.1002/central/CN-00559116/full. | |
| 437 | Rejected by TIAB Screening | Chao YMY, Chiang HH. Narrative Pedagogy in Nursing Education: The Essence of Clinical Nursing Process Recording. [Chinese]. Hu li za zhi The journal of nursing. 2017;64(1):32-40. | |
| 438 | Rejected by TIAB Screening | Chao YY, Chiang HH. [Narrative Pedagogy in Nursing Education: The Essence of Clinical Nursing Process Recording]. Hu Li Tsa Chih - Journal of Nursing. 2017;64(1):32-40. | |
| 439 | Rejected by TIAB Screening | Chapman J. 'Bildung': An educational ideal to promote continuing professional development by enhancing critical reflective practice. Physiotherapy (United Kingdom). 2011;97:eS1448-eS9. | |
| 440 | Outside SDL | Chapman J, Aspin D. Schools as Centres of Lifelong Learning for All. Opinion Papers. 1997. | |
| 441 | Rejected by TIAB Screening | Chapman LRE. How workplace learning theory may contribute to reducing diagnostic error. Diagnosis. 2016;3 (2):eA10-eA1. | |
| 442 | Rejected by TIAB Screening | Chau D, Bowe E, Brown R, DiLorenzo A. Implementation of a comprehensive but simple method to manage and track resident knowledge and skill acquisition during pediatric anesthesia training: Impact on its first graduating class. Anesthesia and Analgesia. 2012;114(5):S236. | |
| 443 | Rejected by TIAB Screening | Chau JP, Lam LW, Lui MH, Ip WY, Chien WT, Lee IF, et al. A survey of registered nurses' perceptions of the code of professional conduct in Hong Kong. Journal of Clinical Nursing. 2010;19(23-24):3527-34. | |
| 444 | Rejected by TIAB Screening | Chaudhry S, Fornari A. Creating an internet based medical education journal club. Journal of General Internal Medicine. 2016;31(2):S813. | |
| 445 | Rejected by TIAB Screening | Chegwidden WR. A problem-based learning pathway for medical students: Improving the process through action research. Annals of the Academy of Medicine Singapore. 2006;35(9):642-6. | |
| 446 | Rejected by TIAB Screening | Chen H, Sheu L, O'Sullivan P, Cate O, Teherani A. Legitimate workplace roles and activities for early learners. Medical Education. 2014;48(2):136-45. | |
| 447 | Rejected by TIAB Screening | Chen J, Li YP, Li J. Method of teaching evidence-based medicine in medical postgraduates - Examination paper analysis. [Chinese]. Chinese Journal of Evidence-Based Medicine. 2005;5(7):511-4+61. | |
| 448 | Not Target Group | Chen JH, Bjorkman A, Zou JH, Engstrom M. Self-regulated learning ability, metacognitive ability, and general self-efficacy in a sample of nursing students: A cross-sectional and correlational study. Nurse Education in Practice. 2019;37:15-21. | |
| 449 | Rejected by TIAB Screening | Chen X. Application of the intelligent mobile terminal in the medical oral English teaching. Basic and Clinical Pharmacology and Toxicology. 2019;125 (SUPPL, 9):52. | |
| 450 | Rejected by TIAB Screening | Cheng C-Y, Liou S-R, Hsu T-H, Pan M-Y, Liu H-C, Chang C-H. Preparing nursing students to be competent for future professional practice: Applying the team-based learning-teaching strategy. Journal of Professional Nursing. 2014;30(4):347-56. | |
| 451 | Not Target Group | Cheng S-F, Kuo C-L, Lin K-C, Lee-Hsieh J. Development and preliminary testing of a self-rating instrument to measure self-directed learning ability of nursing students. International Journal of Nursing Studies. 2010;47(9):1152-8. | |
| 452 | Rejected by TIAB Screening | Cheng TL, Greenberg L, Loeser H, Keller D. Teaching Prevention in Pediatrics. Academic Medicine. 2000;75(7):S66-71. | |
| 453 | Rejected by TIAB Screening | Cheong YC, Abdullahi H, Lashen H, Fairlie FM. Can formal education and training improve the outcome of instrumental delivery? European Journal of Obstetrics, Gynecology, & Reproductive Biology. 2004;113(2):139-44. | |
| 454 | Rejected by TIAB Screening | Cheren ME, et al. Learning Management: Emerging Directions for Learning To Learn in the Workplace. Information Series No. 320. ERIC Publications. ERIC Clearinghouse on Adult, Career, and Vocational Education, Columbus, OH.; 1987. Report No.: 400-84-0011. | |
| 455 | Rejected by TIAB Screening | Chestnut DH. On the Road to Professionalism. Anesthesiology. 2017;126(5):780-6. | |
| 456 | Rejected by TIAB Screening | Chetlen AL, Dell CM, Solberg AO, Otero HJ, Burton KR, Heller MT, et al. Another Time, Another Space: The Evolution of the Virtual Journal Club. Academic Radiology. 2017;24(3):273-85. | |
| 457 | Rejected by TIAB Screening | Chetlen AL, Petscavage-Thomas J, Cherian RA, Ulano A, Nandwana SB, Curci NE, et al. Collaborative Learning in Radiology: From Peer Review to Peer Learning and Peer Coaching. Academic Radiology. 2019. | |
| 458 | Rejected by TIAB Screening | Chiang H-Y. Effects and users' perceptions of computer-based instruction. Dissertation Abstracts International: Section B: The Sciences and Engineering. 2007;68(4-B):2222. | |
| 459 | Rejected by TIAB Screening | Chiang HH. [Subjectivity of the Body in Nursing Care]. Hu Li Tsa Chih - Journal of Nursing. 2019;66(5):26-31. | |
| 460 | Outside SDL | Chichester M. Lifelong learning, part 2: pursuing your BSN and beyond. Nursing for Women's Health. 2011;15(2):171-5. | |
| 461 | Rejected by TIAB Screening | Chichester T, Hagglund K, Edhayan E. Teaching surgical residents to evaluate scholarly articles: a constructivist approach. American Journal of Surgery. 2013;205(3):259-62; discussion 63. | |
| 462 | Rejected by TIAB Screening | Chin MH, Humikowski CA. When is risk stratification by race or ethnicity justified by medical care? Academic Medicine. 2002;77(3):202-8. | |
| 463 | Rejected by TIAB Screening | Chinula L, Chiudzu G, Tang JH, Gopal S, Tomoka T, Kachingwe J, et al. A tailored approach to building specialized surgical oncology capacity: Early experiences and outcomes in Malawi. Gynecologic Oncology Reports. 2018;26:60-5. | |
| 464 | Rejected by TIAB Screening | Chirico M, Thompson JR, Steil C. Development and implementation of a collaborative interprofessional learning program. Currents in Pharmacy Teaching and Learning. 2014;6(4):550-7. | |
| 465 | Rejected by TIAB Screening | Chiu M, Polivka BJ, Stanley SAR. Evaluation of a Disaster-Surge Training for Public Health Nurses. Public Health Nursing. 2012;29(2):136-42. | |
| 466 | Formal Teaching | Chiu YL, Liang JC, Mao PC, Tsai CC. Improving Health Care Providers' Capacity for Self-Regulated Learning in Online Continuing Pharmacy Education: The Role of Internet Self-Efficacy. Journal of Continuing Education in the Health Professions. 2016;36(2):89-95. | |
| 467 | Rejected by TIAB Screening | Cho J, Kwon H, Kim H, Oh J, Yoon K. Effects on diabetes management of a health-care provider mediated, remote coaching system via a PDA-type glucometer and the Internet. Journal of telemedicine and telecare [Internet]. 2011; 17(7):[365‐70 pp.]. Available from: https://www.cochranelibrary.com/central/doi/10.1002/central/CN-00905075/full. | |
| 468 | Not Target Group | Cho KK, Marjadi B, Langendyk V, Hu W. Medical student changes in self-regulated learning during the transition to the clinical environment. BMC Medical Education. 2017;17(1):59. | |
| 469 | Rejected by TIAB Screening | Cho MK, Kim MY. Outcomes and influential factors applying flipped learning methods in a clinical adult nursing practicum. International Journal of Nursing Practice. 2019;25(2):e12724. | |
| 470 | Rejected by TIAB Screening | Choi SE, Kim EA. Effects of Learning Activities on Application of Learning Portfolio in Nursing Management Course. [Korean]. Journal of Korean Academy of Nursing. 2016;46(1):90-9. | |
| 471 | Not Target Group | Choi-Lundberg DL, Low TF, Patman P, Turner P, Sinha SN. Medical student preferences for self-directed study resources in gross anatomy. Anatomical sciences education. 2016;9(2):150-60. | |
| 472 | Rejected by TIAB Screening | Chou CH, Wu SF, Liang SY, Chung ML, Chen MY, Chen ML, et al. [Taiwan nursing student assessment of the value of a competence-based medical-surgical clinical performance examination model]. [Chinese]. Hu li za zhi The journal of nursing. 2010;57(3):43-50. | |
| 473 | Rejected by TIAB Screening | Chou CL, Teherani A, Masters DE, Vener M, Wamsley M, Poncelet A. Workplace learning through peer groups in medical school clerkships. Medical Education Online. 2014;19:25809. | |
| 474 | Rejected by TIAB Screening | Chow M, Chan L, Lo B, Chu WP, Chan T, Lai YM. Exploring the intention to use a clinical imaging portal for enhancing healthcare education. Nurse Education Today. 2013;33(6):655-62. | |
| 475 | Rejected by TIAB Screening | Chow SL, Herman-Kideckel S, Mahendira D, McDonald-Blumer H. Immunology for rheumatology residents: Working toward a Canadian national curriculum consensus. Journal of Clinical Rheumatology. 2015;21(1):10-4. | |
| 476 | Rejected by TIAB Screening | Chua RL, de Guzman AB. Do you see what I see? Understanding Filipino elderly's needs, benefits, and expectations from an adult continuing education program. Educational Gerontology. 2014;40(1):1-15. | |
| 477 | Rejected by TIAB Screening | Chung AS. Mindfulness in emergency medicine. Annals of Emergency Medicine. 2015;66(4):S162. | |
| 478 | Rejected by TIAB Screening | Chung AS, Felber R, Han E, Mathew T, Rebillot K, Likourezos A. A high-impact mindfulness in emergency medicine curriculum for medical students. Annals of Emergency Medicine. 2017;70 (4 Supplement 1):S166-S7. | |
| 479 | Rejected by TIAB Screening | Chung JC. Active learning of geriatric rehabilitation: deliberations of an undergraduate occupational therapy programme. Scandinavian Journal of Caring Sciences. 2001;15(3):250-6. | |
| 480 | Formal Teaching | Cibulka NJ. Educating nurses about research ethics and practices with a self-directed practice-based learning program. The Journal of Continuing Education in Nursing. 2011;42(11):516-21. | |
| 481 | Rejected by TIAB Screening | Ciccarelli MR, Gladstone EB, Armstrong Richardson EA. Implementation of a Transdisciplinary Team for the Transition Support of Medically and Socially Complex Youth. Journal of pediatric nursing. 2015;30(5):661-7. | |
| 482 | Rejected by TIAB Screening | Cismondi IA, Kohan R, Adams H, Bond M, Brown R, Cooper JD, et al. Guidelines for incorporating scientific knowledge and practice on rare diseases into higher education: neuronal ceroid lipofuscinoses as a model disorder. Biochimica et Biophysica Acta. 2015;1852(10 Pt B):2316-23. | |
| 483 | Rejected by TIAB Screening | Claesson M, Jonasson LL, Lindberg E, Josefsson K. What implies registered nurses' leadership close to older adults in municipal home health care? A systematic review. BMC Nursing. 2020;19:30. | |
| 484 | Rejected by TIAB Screening | Clardy PF, Schwartzstein RM. Considering cognition. Current challenges and future directions in pulmonary and critical care fellowship training. Annals of the American Thoracic Society. 2015;12(4):474-9. | |
| 485 | Not Target Group | Clark KM, Dickinson G. Self-Directed and Other-Directed Continuing Education: A Study of Nurses' Participation. Journal of Continuing Education in Nursing. 1976;7(4):16-24. | |
| 486 | Rejected by TIAB Screening | Clarke C, Martin M, Sadlo G, de-Visser R. The development of an authentic professional identity on role-emerging placements. The British Journal of Occupational Therapy. 2014;77(5):222-9. | |
| 487 | Formal Teaching | Clarke JL. Portfolios and practices for lifelong learning in ultrasound education. Ultrasound in Medicine and Biology. 2019;45 (Supplement 1):S73. | |
| 488 | Rejected by TIAB Screening | Clauson KA, Singh-Franco D, Sircar-Ramsewak F, Joseph S, Sandars J. Social media use and educational preferences among first-year pharmacy students. Teaching and Learning in Medicine. 2013;25(2):122-8. | |
| 489 | Rejected by TIAB Screening | Claussen C, Wells LM, Aspenlieder L, Boutilier S. Developing domestic violence primary prevention capacity through a community of practice project: Learnings from Alberta, Canada. Cogent Medicine. 2017;4(1). | |
| 490 | Rejected by TIAB Screening | Cleary M, Freeman A. Fostering a culture of support in mental health settings: alternatives to traditional models of clinical supervision. Issues in Mental Health Nursing. 2006;27(9):985-1000. | |
| 491 | Not Target Group | Cleary TJ. Self-Regulated Learning Interventions with At-Risk Youth: Enhancing Adaptability, Performance, and Well-Being. Applying Psychology in the Schools Series. American Psychological Association. 2015. | |
| 492 | Rejected by TIAB Screening | Clementz L, Dolansky M, Lawrence RH, Rusterholtz A, Singh S, Heilman A, et al. Dyad teams: Interprofessional collaboration and learning in the ambulatory setting. Journal of General Internal Medicine. 2015;30:S493-S4. | |
| 493 | Rejected by TIAB Screening | Clinard ES, Dudding CC. Integrating Simulations Into Communication Sciences and Disorders Clinical Curriculum: Impact of Student Perceptions. American Journal of Speech-Language Pathology. 2019;28(1):136-47. | |
| 494 | Rejected by TIAB Screening | Coffman JM, McConkey MJ, Colee J. Effectiveness of video-assisted, self-directed, and peer-guided learning in the acquisition of surgical skills by veterinary students. Veterinary Surgery. 2020;49(3):582-9. | |
| 495 | Rejected by TIAB Screening | Coggins CC. Midwest Research-to-Practice. Proceedings of the Conference in Adult and Continuing Education (7th, Madison, Wisconsin, October 21-22, 1988). Collected Works - Proceedings Reports - Research. Wisconsin Univ., Madison. Dept of Continuing and Vocational Education.; 1988. | |
| 496 | Rejected by TIAB Screening | Cohen A, Nottingham C, Packiam V, Jaskowiak N, Gundeti M. Attitudes and knowledge of urethral catheters: a targeted educational intervention. BJU International. 2016;118(4):654-9. | |
| 497 | Rejected by TIAB Screening | Cole JG. A study of practice-based learning in family physicians in an academic clinical practice. Dissertation Abstracts International Section A: Humanities and Social Sciences. 2012;72(9-A):3076. | |
| 498 | Outside SDL | Collard N, O'Leary K, Allinson Y, Kirsa S. Implementing MoodleTM to support lifelong learning for pharmacists. Pharmacy Education. 2013;13 (1):105-6. | |
| 499 | Rejected by TIAB Screening | Collier AP, Heilig LF, Schilling LM, Dellavalle RP. Clinical questions asked by medical students: A learning tool for dermatology rotations. Dermatology. 2007;214(2):108-11. | |
| 500 | Rejected by TIAB Screening | Collin K, Paloniemi S, Virtanen A, Etelapelto A. Constraints and Challenges on Learning and Construction of Identities at Work. Vocations and Learning. 2008;1(3):191-210. | |
| 501 | Rejected by TIAB Screening | Collin K, Sintonen T, Paloniemi S, Auvinen T. Work, power and learning in a risk filled occupation. Management Learning. 2011;42(3):301-18. | |
| 502 | Formal Teaching | Collins J. Education techniques for lifelong learning: Principles of adult learning. Radiographics. 2004;24(5):1483-9. | |
| 503 | Rejected by TIAB Screening | Collins J, Blankenbaker DG, Albanese MA, Stack SP, Heiserman KK, Primack SL, et al. Chest radiology case exchange program: a paradigm for resident teaching and independent resident learning. Academic Radiology. 1999;6(1):34-9. | |
| 504 | Rejected by TIAB Screening | Collins J, Riebe JD, Albanese MA, Dobos N, Heiserman K, Primack SL, et al. Medical students and radiology residents: Can they learn as effectively with the same educational materials? Academic Radiology. 1999;6(11 SUPPL. 7):691-5. | |
| 505 | Rejected by TIAB Screening | Collins JW, Wisz P. Training in robotic surgery, replicating the airline industry. How far have we come? World journal of urology. 2019;17. | |
| 506 | Rejected by TIAB Screening | Comes J, Darracq MA, Armenian P, Thornton SL. Emergency medicine residency toxicology education: A survey study. Academic Emergency Medicine. 2017;24 (Supplement 1):S250. | |
| 507 | Rejected by TIAB Screening | Commander CW, Pabon-Ramos WM, Isaacson AJ, Yu H, Burke CT, Dixon RG. Assessing medical students' knowledge of IR at two American medical schools. Journal of Vascular and Interventional Radiology. 2014;25(11):1801-7.e5. | |
| 508 | Rejected by TIAB Screening | Conceicao SCO, Johaningsmeir S, Colby H, Gordon J. Family Caregivers as Lay Trainers: Perceptions of Learning and the Relationship between Life Experience and Learning. Adult Learning. 2014;25(4):151-9. | |
| 509 | Not Target Group | Confessore GJ, Confessore SJ. Adopting Self-Directed Learning in Continuing Professional Education: Physicians and Architects. New Directions for Adult and Continuing Education n64 p31. 1994;38. | |
| 510 | Rejected by TIAB Screening | Connell SE, Yates P, Barrett L. Understanding the optimal learning environment in palliative care. Nurse Education Today. 2011;31(5):472-6. | |
| 511 | Rejected by TIAB Screening | Connelly DP, Sielaff BH, Scott EP. ESPRE - Expert system for platelet request evaluation. American Journal of Clinical Pathology. 1990;94(4):S19-S24. | |
| 512 | Rejected by TIAB Screening | Connolly M, Thomas JM, Orford JA, Schofield N, Whiteside S, Morris J, et al. The impact of the SAGE & THYME foundation level workshop on factors influencing communication skills in health care professionals. The Journal of continuing education in the health professions. 2014;34(1):37-46. | |
| 513 | Rejected by TIAB Screening | Connor DE. Bengt liliequist: Life and accomplishments of a true renaissance man. Journal of Neurosurgery. 2013;119 (2):A557. | |
| 514 | Formal Teaching | Considine J, Botti M, Thomas S. Effect of a self-directed learning package on emergency nurses' knowledge of assessment of oxygenation and use of supplemental oxygen. Nursing & Health Sciences. 2005;7(3):199-208. | |
| 515 | Rejected by TIAB Screening | Considine J, Botti M, Thomas S. The effects of specific educational preparation on emergency nurses' clinical decisions regarding supplemental oxygen administration. Nursing & Health Sciences. 2006;8(2):73-80. | |
| 516 | Rejected by TIAB Screening | Consoli S, Ben SM, Jean J, Ménard J, Plouin P, Chatelier G. Evaluation of a computer assisted training program for hypertensive patients. Archives des maladies du coeur ET des vaisseaux [Internet]. 1994; 87(8):[1093‐6 pp.]. Available from: https://www.cochranelibrary.com/central/doi/10.1002/central/CN-00114286/full. | |
| 517 | Rejected by TIAB Screening | Consoli S, Ben SM, Jean J, Menard J, Plouin P, Chatelier G. Interactive electronic teaching (ISIS): has the future started? Journal of human hypertension [Internet]. 1996; 10 Suppl 1:[S69‐72 pp.]. Available from: https://www.cochranelibrary.com/central/doi/10.1002/central/CN-00125534/full. | |
| 518 | Rejected by TIAB Screening | Conway PF, Munthe E. The practice turn: Research-informed clinical teacher education in two countries. From vocational to professional education: Educating for social welfare. New York, NY: Routledge/Taylor & Francis Group; US; 2015. p. 146-63. | |
| 519 | Rejected by TIAB Screening | Conway PH, Cassel CK. Engaging physicians and leveraging professionalism: A key to success for quality measurement and improvement. JAMA: Journal of the American Medical Association. 2012;308(10):979-80. | |
| 520 | Formal Teaching | Cook DA, Aljamal Y, Pankratz VS, Sedlack RE, Farley DR, Brydges R. Supporting self-regulation in simulation-based education: a randomized experiment of practice schedules and goals. Advances in Health Sciences Education. 2019;24(2):199-213. | |
| 521 | Rejected by TIAB Screening | Cook DA, Holmboe ES, Sorensen KJ, Berger RA, Wilkinson JM. Getting maintenance of certification to work: a grounded theory study of physicians' perceptions. JAMA Internal Medicine. 2015;175(1):35-42. | |
| 522 | Rejected by TIAB Screening | Cook JM, Newman E, Simiola V. Trauma training: Competencies, initiatives, and resources. Psychotherapy. 2019;56(3):409-21. | |
| 523 | Rejected by TIAB Screening | Cooke IE, Sackett DL. Evidence-based obstetrics and gynaecology. Bailliere's Clinical Obstetrics and Gynaecology. 1996;10(4):535-49. | |
| 524 | Outside SDL | Cooper K. The Value of Lifelong Learning. Oregon Nurse. 2015:2. | |
| 525 | Rejected by TIAB Screening | Cooper LD, Wieckowski AT. A structured approach to reflective practice training in a clinical practicum. Training and Education in Professional Psychology. 2017;11(4):252-9. | |
| 526 | Rejected by TIAB Screening | Corbett D. Letter - Response to 'Future-proofing primary health care: GP recruitment and retention in the new NHS'. British Journal of General Practice. 2011;19:19. | |
| 527 | Rejected by TIAB Screening | Cordovani L, Wong A, Monteiro S. Maintenance of certification for practicing physicians: a review of current challenges and considerations. Canadian Medical Education Journal [Electronic Resource]. 2020;11(1):e70-e80. | |
| 528 | Rejected by TIAB Screening | Cornell RL. A Study of Transformational Change at Three Schools of Nursing Implementing Healthcare Informatics. ProQuest LLC EdD Dissertation, University of South Florida. 2009. | |
| 529 | Rejected by TIAB Screening | Cowan P. Postgraduate training options in dentistry--the RCSI roadmap. Surgeon Journal of the Royal Colleges of Surgeons of Edinburgh & Ireland. 2007;5(2):90-3; quiz 3, 121. | |
| 530 | Rejected by TIAB Screening | Cox AM. Students' Experience of University Space: An Exploratory Study. International Journal of Teaching and Learning in Higher Education. 2011;23(2):197-207. | |
| 531 | Outside SDL | Cox J, Simpson MD. Exploring the Link between Self-Efficacy, Workplace Learning and Clinical Practice. Asia Pacific Journal of Cooperative Education. 2016;17(3):215-25. | |
| 532 | Outside SDL | Crellin A. Revalidation and lifelong learning. Clinical Oncology (Royal College of Radiologists). 2011;23(10):657-8. | |
| 533 | Rejected by TIAB Screening | Creta AM, Gross AH. Components of an Effective Professional Development Strategy: The Professional Practice Model, Peer Feedback, Mentorship, Sponsorship, and Succession Planning. Seminars in Oncology Nursing. 2020;36(3):151024. | |
| 534 | Outside SDL | Cribbin J, Kennedy P. Lifelong Learning in Action: Hong Kong Practitioners' Perspectives2002. | |
| 535 | Rejected by TIAB Screening | Criscitelli T. Informal mentoring in nursing. Mentoring in formal and informal contexts. Charlotte, NC: IAP Information Age Publishing; US; 2016. p. 263-77. | |
| 536 | Rejected by TIAB Screening | Crits-Christoph P, Ring-Kurtz S, McClure B, Temes C, Kulaga A, Gallop R, et al. A randomized controlled study of a web-based performance improvement system for substance abuse treatment providers. Journal of substance abuse treatment [Internet]. 2010; 38(3):[251‐62 pp.]. Available from: https://www.cochranelibrary.com/central/doi/10.1002/central/CN-00752897/full. | |
| 537 | Rejected by TIAB Screening | Crockett ET. A research education program model to prepare a highly qualified workforce in biomedical and health-related research and increase diversity. BMC Medical Education. 2014;14:202. | |
| 538 | Rejected by TIAB Screening | Croen LG, Lief PD, Frishman WH. Integrating basic science and clinical teaching for third-year medical students. Journal of Medical Education. 1986;61(6):444-53. | |
| 539 | Rejected by TIAB Screening | Cronin L, Hughes J, Grafton R, Leach P, Lewis H, Moss K, et al. The knowledge gained by a novice inflammatory bowel disease nurse in the first year of service in comparison to experienced IBD nurses knowledge. Journal of Crohn's and Colitis. 2015;9:S449. | |
| 540 | Not Target Group | Crook J. A validation study of a self-directed learning readiness scale. Journal of Nursing Education. 1985;24(7):274-9. | |
| 541 | Rejected by TIAB Screening | Cross V. The professional development diary. A case study of one cohort of physiotherapy students. Physiotherapy. 1997;83(7):375-83. | |
| 542 | Rejected by TIAB Screening | Crosser GH. Decoding Fad Diets. Nutrition in Health Promotion Series, Number 20. Guides - Classroom - Learner. Ohio State Univ., Columbus. Dept. of Family Medicine.; 1985. | |
| 543 | Rejected by TIAB Screening | Crossland A. Influence of efficacy beliefs on the learning experiences of children with cancer in the hospital setting. Dissertation Abstracts International Section A: Humanities and Social Sciences. 2001;62(4-A):1323. | |
| 544 | Rejected by TIAB Screening | Crossley JG. Development and validation of a trustworthy multisource feedback instrument to support nurse appraisals. The Journal of continuing education in the health professions. 2015;35(2):91-8. | |
| 545 | Rejected by TIAB Screening | Crutcher RA, Szafran O, Woloschuk W, Chaytors RG, Topps DA, Humphries PW, et al. Where Canadian family physicians learn procedural skills. Family Medicine. 2005;37(7):491-5. | |
| 546 | Not Target Group | Cudney SA. Mediated Self-Instruction of Basic Nursing Skills. Nurse Educator. 1976;1(2):14-5. | |
| 547 | Rejected by TIAB Screening | Cullen MW, Geske JB, Anavekar NS, McAdams JA, Beliveau ME, Ommen SR, et al. Reinvigorating Continuing Medical Education: Meeting the Challenges of the Digital Age. Mayo Clinic Proceedings. 2019;94(12):2501-9. | |
| 548 | Rejected by TIAB Screening | Culley DJ, Sun H, Harman AE, Warner DO. Perceived value of Board certification and the Maintenance of Certification in Anesthesiology Program (MOCA R). Journal of Clinical Anesthesia. 2013;25(1):12-9. | |
| 549 | Rejected by TIAB Screening | Cummings J. Enhancing airway management capacity for out-of-hospital providers in a limited resource setting. Annals of Emergency Medicine. 2011;58(4):S332-S3. | |
| 550 | Rejected by TIAB Screening | Cunningham R, Whiteside L, Chermack S, Zimmerman M, Shope J, Bingham C, et al. Dating violence: outcomes following a brief motivational interviewing intervention among at-risk adolescents in an urban emergency department. Academic emergency medicine [Internet]. 2013; 20(6):[562‐9 pp.]. Available from: https://www.cochranelibrary.com/central/doi/10.1002/central/CN-00963485/full. | |
| 551 | Rejected by TIAB Screening | Curioso WH, Montori VM, Curioso WI. [Evidence based medicine for the gastroenterologist]. Revista de Gastroenterologia del Peru. 2004;24(1):75-91. | |
| 552 | Not Target Group | Curran V, Gustafson DL, Simmons K, Lannon H, Wang C, Garmsiri M, et al. Adult Learners&apos; Perceptions of Self-Directed Learning and Digital Technology Usage in Continuing Professional Education: An Update for the Digital Age. Journal of Adult and Continuing Education. 2019;25(1):74-93. | |
| 553 | Rejected by TIAB Screening | Curran V, Kirby F, Allen M, Sargeant J. A Mixed Learning Technology Approach for Continuing Medical Education. Medical Education Online. 2003;8(1):4341. | |
| 554 | Rejected by TIAB Screening | Curran V, Matthews L, Fleet L, Simmons K, Gustafson DL, Wetsch L. A Review of Digital, Social, and Mobile Technologies in Health Professional Education. The Journal of continuing education in the health professions. 2017;37(3):195-206. | |
| 555 | Formal Teaching | Currow DC, Abernethy AP. Quality palliative care: practitioners' needs for dynamic lifelong learning. Journal of Pain & Symptom Management. 2005;29(4):332-4. | |
| 556 | Rejected by TIAB Screening | Curtis K, Caldwell E, Delprado A, Munroe B. Traumatic injury in Australia and New Zealand. Australasian Emergency Nursing Journal. 2012;15(1):45-54. | |
| 557 | Rejected by TIAB Screening | Cutrer WB, Atkinson HG, Friedman E, Deiorio N, Gruppen LD, Dekhtyar M, et al. Exploring the characteristics and context that allow Master Adaptive Learners to thrive. Medical Teacher. 2018;40(8):791-6. | |
| 558 | Rejected by TIAB Screening | Cuvelier L. Taking Risks to Improve Safety? Workplace Learning in Anesthesia. Journal of Workplace Learning. 2019;31(8):537-50. | |
| 559 | Rejected by TIAB Screening | Czabanowska K, Smith T, Konings KD, Sumskas L, Otok R, Bjegovic-Mikanovic V, et al. In search for a public health leadership competency framework to support leadership curriculum-a consensus study. European journal of public health. 2014;24(5):850-6. | |
| 560 | Rejected by TIAB Screening | Czekalla J. Critical appraisal of clinical studies and meta-analyses - An educational article on the most important validity criteria according to Evidence-based Medicine (EbM). [German]. Psychopharmakotherapie. 2006;13(6):224-30. | |
| 561 | Rejected by TIAB Screening | Czeskleba A, Holzhausen Y, Peters H. Patient safety during final-year clerkships: A qualitative study of possible error sources and of the potential of Entrustable Professional Activities. GMS Journal for Medical Education. 2019;36(2):Doc18. | |
| 562 | Rejected by TIAB Screening | Daelmans HEM, Hoogenboom RJI, Donker AJM, Scherpbier AJJA, Stehouwer CDA, Van Der Vleuten CPM. Effectiveness of clinical rotations as a learning environment for achieving competences. Medical Teacher. 2004;26(4):305-12. | |
| 563 | Rejected by TIAB Screening | Daeyoung W. Effects of programmed information on coping behavior and emotions of mothers of young children undergoing IV procedures. Taehan Kanho Hakhoe chi [Internet]. 2006; 36(8):[1301‐7 pp.]. Available from: https://www.cochranelibrary.com/central/doi/10.1002/central/CN-00577653/full. | |
| 564 | Rejected by TIAB Screening | Dale VH, Pierce SE, May SA. Motivating factors and perceived barriers to participating in continuing professional development: a national survey of veterinary surgeons. Veterinary Record. 2013;173(10):247. | |
| 565 | Rejected by TIAB Screening | Daly M, Gonzalez E, Siracuse-Lee D, Legutko P. Efficacy of surgical simulator training versus traditional wet-lab training on operating room performance of ophthalmology residents during the capsulorhexis in cataract surgery. Journal of cataract and refractive surgery [Internet]. 2013; 39(11):[1734‐41 pp.]. Available from: https://www.cochranelibrary.com/central/doi/10.1002/central/CN-01120720/full. | |
| 566 | Rejected by TIAB Screening | Dandavino M, Snell L, Wiseman J. Why medical students should learn how to teach. Medical Teacher. 2007;29(6):558-65. | |
| 567 | Rejected by TIAB Screening | Daniel M, Gay T, Mangrulkar R, Ross P, Weir S, Hogikyan E, et al. Training wheels needed: Lessons in professionalism from a liberal deferral policy. Perspectives on Medical Education. 2019;8(3):187-90. | |
| 568 | Formal Teaching | Daniels EB. The Impact of an Educational Intervention on Self-Directed Learning Readiness and Behaviors in Undergraduate Nursing Students. ProQuest LLC PhD Dissertation, Capella University. 2011. | |
| 569 | Rejected by TIAB Screening | Darbyshire P. In defence of pedagogy: a critique of the notion of andragogy. Nurse education today. 1993;13(5):328-35. | |
| 570 | Rejected by TIAB Screening | DaRosa DA, et al. Evaluating the Effectiveness of the Lecture versus Independent Study. Evaluation and Program Planning. 1991;14(3):141-46. | |
| 571 | Rejected by TIAB Screening | Dave Davis DA, McMahon GT. Translating evidence into practice: Lessons for CPD. Medical Teacher. 2018;40(9):892-5. | |
| 572 | Formal Teaching | David Y, Chang AA, Lee MS. Fostering self-directed online learning through resident-led group teaching. Journal of General Internal Medicine. 2018;33 (2 Supplement 1):708. | |
| 573 | Rejected by TIAB Screening | Davidson I, Thomas H. Lessons from the higher professional education scheme for general practice training. Education for Primary Care. 2008;19(6):605-14. | |
| 574 | Rejected by TIAB Screening | Davies D, Quick C. Medical gross anatomy: Interdisciplinary efforts to increase active learning through collection and histological assessment of biopsies. FASEB Journal Conference: Experimental Biology. 2015;29(1 Meeting Abstracts). | |
| 575 | Rejected by TIAB Screening | Davies K, Curtin M, Robson K. Impact of an international workplace learning placement on personal and professional development. Australian Occupational Therapy Journal. 2017;64(2):121-8. | |
| 576 | Rejected by TIAB Screening | Davis C, Burke L. The effectiveness of clinical supervision for a group of ward managers based in a district general hospital: An evaluative study. Journal of Nursing Management. 2012;20(6):782-93. | |
| 577 | Formal Teaching | Davis DA, Mazmanian PE, Fordis M, Harrison R, Thorpe KE, Perrier L. Accuracy of Physician Self-assessment Compared With Observed Measures of Competence: A Systematic Review. JAMA: Journal of the American Medical Association. 2006;296(9):1094-102. | |
| 578 | Not Target Group | Davis J. Education through Self-directed Learning. Australian Nursing & Midwifery Journal. 2015;23(1):26-7. | |
| 579 | Rejected by TIAB Screening | Davis K, White S, Stephenson M. The influence of workplace culture on nurses' learning experiences: a systematic review of qualitative evidence. JBI Database Of Systematic Reviews And Implementation Reports. 2016;14(6):274-346. | |
| 580 | Formal Teaching | Davis M, Leggatt L, Romano S, Van Aarsen K. Self-directed learning in advanced care paramedics: Perceived deficits and completed activities. Canadian Journal of Emergency Medicine. 2018;20 (Supplement 1):S66-S7. | |
| 581 | Rejected by TIAB Screening | Davis M, Leggatt L, Van Aarsen K, Romano S. A descriptive needs-based assessment of paramedic continuing education. Canadian Journal of Emergency Medicine. 2018;20 (Supplement 1):S66. | |
| 582 | Rejected by TIAB Screening | Davis MH, Ponnamperuma GG, Ker JS. Student perceptions of a portfolio assessment process. Medical Education. 2009;43(1):89-98. | |
| 583 | Rejected by TIAB Screening | Davison AG, Jongepier L. Training deficiencies and lack of confidence around knowledge in primary care nurses treating asthma and COPD patients. Thorax. 2012;67:A167. | |
| 584 | Rejected by TIAB Screening | Davoodi R, Mohammadzadeh Shabestari M, Takbiri A, Soltanifar A, Sabouri G, Rahmani S, et al. Patient Safety Culture Based on Medical Staff Attitudes in Khorasan Razavi Hospitals, Northeastern Iran. Iranian Journal of Public Health. 2013;42(11):1292-8. | |
| 585 | Rejected by TIAB Screening | de BD, de GM, Bosmans J, de KJ, Mokkenstorm J, Verwey B, et al. Reducing patients' suicide ideation through training mental health teams in the application of the Dutch multidisciplinary practice guideline on assessment and treatment of suicidal behavior: study protocol of a randomized controlled trial. Trials [Internet]. 2013; 14:[372 p.]. Available from: https://www.cochranelibrary.com/central/doi/10.1002/central/CN-01120522/full. | |
| 586 | Rejected by TIAB Screening | de Bruin AB, Dunlosky J, Cavalcanti RB. Monitoring and regulation of learning in medical education: The need for predictive cues. Medical Education. 2017;51(6):575-84. | |
| 587 | Rejected by TIAB Screening | de Feijter JM, de Grave WS, Dornan T, Koopmans RP, Scherpbier AJ. Students' perceptions of patient safety during the transition from undergraduate to postgraduate training: an activity theory analysis. Advances in Health Sciences Education. 2011;16(3):347-58. | |
| 588 | Rejected by TIAB Screening | de Feijter JM, de Grave WS, Koopmans RP, Scherpbier AJJA. Informal learning from error in hospitals: What do we learn, how do we learn and how can informal learning be enhanced? A narrative review. Advances in Health Sciences Education. 2013;18(4):787-805. | |
| 589 | Rejected by TIAB Screening | de Groot E, Jaarsma D, Endedijk M, Mainhard T, Lam I, Simons R-J, et al. Critically Reflective Work Behavior of Health Care Professionals. Journal of Continuing Education in the Health Professions. 2012;32(1):48-57. | |
| 590 | Rejected by TIAB Screening | de Groot E, van den Berg BAM, Endedijk MD, van Beukelen P, Simons PRJ. Critically Reflective Work Behaviour within Autonomous Professionals' Learning Communities. Vocations and Learning. 2011;4(1):41-62. | |
| 591 | Rejected by TIAB Screening | de Jesus LE. Surgions train: Always like today?. [Portuguese]. Revista do Colegio Brasileiro de Cirurgioes. 2009;36(6):529-32. | |
| 592 | Rejected by TIAB Screening | de Jong N, Konings KD, Czabanowska K. The Development of Innovative Online Problem-Based Learning: A Leadership Course for Leaders in European Public Health. Journal of University Teaching and Learning Practice. 2014;11(3). | |
| 593 | Rejected by TIAB Screening | De La Flor M, Subias E, Romero M, Lopez M, Remacha M, Miralles RM. Strategies to improve team work, assistant/resident, in the gynaecological operating theatres. [Spanish]. Ginecologia y Obstetricia Clinica. 2009;10(2):88-93. | |
| 594 | Rejected by TIAB Screening | De Leon JE. Institutional Goal Priorities in Texas: A Look at an Associate Degree Nursing Program. Reports - Descriptive Speeches/Meeting Papers. 1995. | |
| 595 | Rejected by TIAB Screening | De LR, Calabrò R, Gervasi G, De SS, Bonanno L, Corallo F, et al. Is computer-assisted training effective in improving rehabilitative outcomes after brain injury? A case-control hospital-based study. Disability and health journal [Internet]. 2014; 7(3):[356‐60 pp.]. Available from: https://www.cochranelibrary.com/central/doi/10.1002/central/CN-01115744/full. | |
| 596 | Rejected by TIAB Screening | De Simone S, Planta A, Bianco P. [The role of agentic capacities in turnover intentions among nurses]. Giornale Italiano di Medicina del Lavoro Ed Ergonomia. 2018;40(4):208-16. | |
| 597 | Rejected by TIAB Screening | de Visser M, Fluit C, Cohen-Schotanus J, Laan R. Do different curriculum aligned selection procedures admit students with different personality profiles to medical school? PLoS ONE [Electronic Resource]. 2018;13(12):e0209312. | |
| 598 | Rejected by TIAB Screening | Dealtry R. The corporate university's role in managing an epoch in learning organisation innovation. Journal of Workplace Learning. 2006;18(5):313-20. | |
| 599 | Rejected by TIAB Screening | Dean GJ, Ferro TR. Pennsylvania Adult and Continuing Education Research Conference Proceedings (Monroeville, Pennsylvania, October 8, 1994). Collected Works - Proceedings. Pennsylvania State Univ., Monroeville., Indiana Univ. of Pennsylvania.; 1994. | |
| 600 | Rejected by TIAB Screening | Dean SJ, Barratt AL, Hendry GD, Lyon PM. Preparedness for hospital practice among graduates of a problem-based, graduate-entry medical program. Medical Journal of Australia. 2003;178(4):163-6. | |
| 601 | Rejected by TIAB Screening | Dearmon VA, Riley BH, Mestas LG, Buckner EB. Bridge to shared governance: developing leadership of frontline nurses. Nursing Administration Quarterly. 2015;39(1):69-77. | |
| 602 | Rejected by TIAB Screening | Dearnley C, Matthew B. A Group of Nurses Experience Open Learning: Exploring the Impact. Open Learning. 2000;15(2):191-206. | |
| 603 | Rejected by TIAB Screening | Decoteau MA, Rivera L, Umali K, Chan AD, Soballe P, Ignacio RC. A multimodal approach improves American Board of Surgery In-Training Examination scores. American Journal of Surgery. 2018;215(2):315-21. | |
| 604 | Rejected by TIAB Screening | Degnan B, Murray L, Dunling C, Whittlestone K, Standley T, Gupta A, et al. The effect of additional teaching on medical students' drug administration skills in a simulated emergency scenario. Anaesthesia [Internet]. 2006; 61(12):[1155‐60 pp.]. Available from: https://www.cochranelibrary.com/central/doi/10.1002/central/CN-00573498/full. | |
| 605 | Rejected by TIAB Screening | Dehabadi M, Fernando B, Berlingieri P. The use of simulation in the acquisition of laparoscopic suturing skills. International Journal of Surgery. 2014;12(4):258-68. | |
| 606 | Rejected by TIAB Screening | Deiorio NM, Carney PA, Kahl LE, Bonura EM, Juve AM. Coaching: a new model for academic and career achievement. Medical Education Online. 2016;21:33480. | |
| 607 | Rejected by TIAB Screening | del Bueno DJ, et al. Implementing a Competency-based Orientation Program. Journal of Nursing Administration. 1981;11(2):24-9. | |
| 608 | Rejected by TIAB Screening | DeLeo A, Geraghty S. iMidwife: midwifery students' use of smartphone technology as a mediated educational tool in clinical environments. Contemporary Nurse. 2018;54(4-5):522-31. | |
| 609 | Rejected by TIAB Screening | Delva M, Kirby JR, Knapper CK, Birtwhistle R. Postal survey of approaches to learning among Ontario physicians: Implications for continuing medical education. BMJ: British Medical Journal. 2002;325(7374):1218-20. | |
| 610 | Rejected by TIAB Screening | Dembitzer A, Gillespie C, Hanley K, Crowe R, Zabar S, Yeboah N, et al. Clinician-educators are more burned out as clinicians than as educators: Implications for teaching (and practice). Journal of General Internal Medicine. 2012;27:S143. | |
| 611 | Outside SDL | Dembitzer A, Wang B, Grask A, Gillespie C, Hanley K, Zabar S, et al. Burnout in clinician-educators and the importance of lifelong learning: Findings from a medical education faculty development program. Journal of General Internal Medicine. 2013;28:S38. | |
| 612 | Rejected by TIAB Screening | Denny M, Higgins A. The use of computer assisted technology to enhance student psychiatric nurses learning during a practice placement. Nurse Education in Practice. 2003;3(2):80-8. | |
| 613 | Not Target Group | Denny VH. Self-Directed Workplace Literacy Distance Learning for Developmental Disabilities Workers. Final Report. Reports - Research. City Univ. of New York, NY. Center for Advanced Study in Education.; 1998. | |
| 614 | Rejected by TIAB Screening | DePaola DP. The revitalization of U.S. dental education. Journal of Dental Education. 2008;72(2 Suppl):28-42. | |
| 615 | Rejected by TIAB Screening | Deretchin LF, Hamilton RJ, Hawkins J, Contant CF. Learning behaviors in a mixed traditional and problem-based learning curriculum. Education for Health. 1999;12(2):169-79. | |
| 616 | Rejected by TIAB Screening | DeRosa RR, Amaya-Jackson L, Layne CM. From rifts to riffs: Evidence-based principles to guide critical thinking about next-generation child trauma treatments and training. Training and Education in Professional Psychology. 2013;7(3):195-204. | |
| 617 | Outside SDL | DeSilets LD. A progressive calling - nursing requires lifelong learning. Pennsylvania Nurse. 1982;37(5):3. | |
| 618 | Rejected by TIAB Screening | Desteghe L, Germeys J, Vijgen J, Koopman P, Dilling-Boer D, Schurmans J, et al. Effectiveness and usability of an online tailored education platform for atrial fibrillation patients undergoing a direct current cardioversion or pulmonary vein isolation. International journal of cardiology [Internet]. 2018; 272:[123‐9 pp.]. Available from: https://www.cochranelibrary.com/central/doi/10.1002/central/CN-01618116/full. | |
| 619 | Rejected by TIAB Screening | Deutschman M. Interventions to nurture excellence in the nursing home culture. Journal of Gerontological Nursing. 2001;27(8):37-43. | |
| 620 | Rejected by TIAB Screening | Devine OP, Harborne AC, Horsfall HL, Joseph T, Marshall-Andon T, Samuels R, et al. The Analysis of Teaching of Medical Schools (AToMS) survey: An analysis of 47,258 timetabled teaching events in 25 UK medical schools relating to timing, duration, teaching formats, teaching content, and problem-based learning. BMC Medicine. 2020;18(1). | |
| 621 | Rejected by TIAB Screening | DeWitt TG. Medical education in the United States: The role of andragogy. Hong Kong Journal of Paediatrics. 2017;22(1):34-8. | |
| 622 | Rejected by TIAB Screening | Dey V, Jones A, Spalding E. Telehealth technology: A patient centred intervention in peritoneal dialysis. Nephrology Dialysis Transplantation. 2015;30:iii326. | |
| 623 | Rejected by TIAB Screening | Dhein CR, Memon M. On-line continuing education at the College of Veterinary Medicine, Washington State University. Journal of Veterinary Medical Education. 2003;30(1):41-6. | |
| 624 | Formal Teaching | Diaz Agea JL, Megias Nicolas A, Garcia Mendez JA, Adanez Martinez MG, Leal Costa C. Improving simulation performance through Self-Learning Methodology in Simulated Environments (MAES©). Nurse Education Today. 2019;76:62-7. | |
| 625 | Rejected by TIAB Screening | Dick F, Leaven T, Dillman D, Torner R, Finken L. Core morphological concepts of disease for second-year medical students. Human Pathology. 1998;29(9):1017-20. | |
| 626 | Rejected by TIAB Screening | Dickerman J, Told T. Lifelong learning for the family physician. Osteopathic Family Physician. 2012;4(4):118-23. | |
| 627 | Rejected by TIAB Screening | Dickinson D, Tenhula W, Morris S, Brown C, Peer J, Spencer K, et al. A randomized, controlled trial of computer-assisted cognitive remediation for schizophrenia. American journal of psychiatry [Internet]. 2010; 167(2):[170‐80 pp.]. Available from: https://www.cochranelibrary.com/central/doi/10.1002/central/CN-00727920/full. | |
| 628 | Outside SDL | Dickinson G, Clark KM. Learning Orientations and Participation in Self-Education and Continuing Education. Adult Education. 1975;26(1):3-15. | |
| 629 | Rejected by TIAB Screening | Dietsch E, Mulimbalimba-Masururu L. Learning lessons from a traditional midwifery workforce in western Kenya. Midwifery. 2011;27(3):324-30. | |
| 630 | Rejected by TIAB Screening | DiLullo C, Morris HJ, Kriebel RM. Clinical competencies and the basic sciences: An online case tutorial paradigm for delivery of integrated clinical and basic science content. Anatomical Sciences Education. 2009;2(5):238-43. | |
| 631 | Rejected by TIAB Screening | DiMauro NM. Continuous professional development. Journal of Continuing Education in Nursing. 2000;31(2):59-62. | |
| 632 | Rejected by TIAB Screening | Dinkevich E, Ozuah PO. Self-directed learning activities of paediatric residents. Medical Education. 2003;37(4):388-9. | |
| 633 | Outside SDL | Dixon E. Nurse readiness and time spent in self-directed learning. Journal of Continuing Education in Nursing. 1991;22(5):215-8. | |
| 634 | Rejected by TIAB Screening | Dixon-Woods M, Regan J, Robertson N, Young B, Cordle C, Tobin M. Teaching and learning about human sexuality in undergraduate medical education. Medical Education. 2002;36(5):432-40. | |
| 635 | Rejected by TIAB Screening | Djalali A, Della Corte F, Segond F, Metzger MH, Gabilly L, Grieger F, et al. TIER competency-based training course for the first receivers of CBRN casualties: A European perspective. European Journal of Emergency Medicine. 2017;24(5):371-6. | |
| 636 | Rejected by TIAB Screening | Dochy F, Gijbels D, Segers M, Van den Bossche P. Theories of Learning for the Workplace: Building Blocks for Training and Professional Development Programs. Routledge Psychology in Education. Routledge, Taylor and Francis Group. 2011. | |
| 637 | Rejected by TIAB Screening | Dodd KW, Smith SW, Walsh B, Wang K, Rapin J, Fennell W, et al. A deep neural network learning algorithm outperforms a conventional algorithm for emergency department electrocardiogram interpretation. Academic Emergency Medicine. 2018;25 (Supplement 1):S183-S4. | |
| 638 | Rejected by TIAB Screening | Doherty-Restrepo JL, Hughes BJ, Del Rossi G, Pitney WA. Evaluation Models for Continuing Education Program Efficacy: How Does Athletic Training Continuing Education Measure up? Athletic Training Education Journal. 2009;4(3):117-24. | |
| 639 | Rejected by TIAB Screening | Dolan TC. What physician executives and health care organizations should expect from each other. Physician Executive. 1999;25(5):26-31. | |
| 640 | Rejected by TIAB Screening | Dolovich L, Sabharwal M, Agro K, Foster G, Lee A, McCarthy L, et al. The effect of pharmacist education on asthma treatment plans for simulated patients. Pharmacy World and Science. 2007;29(3):228-39. | |
| 641 | Rejected by TIAB Screening | Dombrowski T, Wrobel C, Dazert S, Volkenstein S. Flipped classroom frameworks improve efficacy in undergraduate practical courses - a quasi-randomized pilot study in otorhinolaryngology. BMC medical education [Internet]. 2018; 18(1):[294 p.]. Available from: https://www.cochranelibrary.com/central/doi/10.1002/central/CN-01777271/full. | |
| 642 | Rejected by TIAB Screening | Domenech Martinez E, Armas Ramos H, Castro Conde JR, Gonzalez Diaz JP, Mendez Perez A, Ormazabal Ramos C, et al. [Study of the introduction of the European Credit Transfer System (ECTS) in pediatrics and modification of the teaching methodology]. Anales de Pediatria. 2006;65(5):415-27. | |
| 643 | Rejected by TIAB Screening | Donald SY, Trueger NS, Liferidge A, Khaldun J, Fair M, Davis S, et al. Teaching health policy: Developing a portable E-learning tool for medical student education. Academic Emergency Medicine. 2013;20(5):S348. | |
| 644 | Formal Teaching | Donato AA, Kaliyadan AG, Wasser T. Self-directed study using MP3 players to improve auscultation proficiency of physicians: a randomized, controlled trial. The Journal of continuing education in the health professions. 2014;34(2):131-8. | |
| 645 | Formal Teaching | Donofrio PD. Practice issues in neurology. CONTINUUM: Lifelong Learning in Neurology. 2010;16(1):170-4. | |
| 646 | Rejected by TIAB Screening | Donohue C. It's a Small World after All!--Taking Your First Steps into Online Teaching and Learning. Child Care Information Exchange n147 p20. 2002;22:24-6. | |
| 647 | Rejected by TIAB Screening | Dorman T, Miller BM. Continuing medical education: The link between physician learning and health care outcomes. Academic Medicine. 2011;86(11):1339. | |
| 648 | Rejected by TIAB Screening | Dornan T, Boshuizen H, King N, Scherpbier A. Experience-based learning: a model linking the processes and outcomes of medical students' workplace learning. Medical Education. 2007;41(1):84-91. | |
| 649 | Not Target Group | Dorr L, Perels F. Improving Metacognitive Abilities as an Important Prerequisite for Self-Regulated Learning in Preschool Children. International Electronic Journal of Elementary Education. 2019;11(5):449-59. | |
| 650 | Rejected by TIAB Screening | Doucet L, Lammens R, Hendrickx S, Dewolf P. App-based learning as an alternative for instructors in teaching basic life support to school children: a randomized control trial. Acta Clinica Belgica: International Journal of Clinical and Laboratory Medicine. 2019;74(5):317-25. | |
| 651 | Rejected by TIAB Screening | Dowling C. A Comparison between Mandatory and Voluntary Continuing Education on Professional Performance. Speeches/Meeting Papers Reports - Research. 1985. | |
| 652 | Rejected by TIAB Screening | Downs M, Turner S, Bryans M, Wilcock J, Keady J, Levin E, et al. Effectiveness of educational interventions in improving detection and management of dementia in primary care: cluster randomised controlled study. BMJ (clinical research ed) [Internet]. 2006; 332(7543):[692‐6 pp.]. Available from: https://www.cochranelibrary.com/central/doi/10.1002/central/CN-00555887/full. | |
| 653 | Rejected by TIAB Screening | Drake R. Flexner! GPEP! What's next for medical education in the United States? Journal of Anatomy. 2012;221 (1):73. | |
| 654 | Rejected by TIAB Screening | Drake RL. A unique, innovative, and clinically oriented approach to anatomy education. Academic Medicine. 2007;82(5):475-8. | |
| 655 | Rejected by TIAB Screening | Dressler RA. Beyond Workbooks: The Computer as a Treatment Supplement. Speeches/Meeting Papers. 1991. | |
| 656 | Not Target Group | Drevdahl DJ, Stackman RW, Purdy JM, Louie BY. Merging Reflective Inquiry and Self-Study as a Framework for Enhancing the Scholarship of Teaching. Journal of Nursing Education. 2002;41(9):413-19. | |
| 657 | Rejected by TIAB Screening | Dröes R, van RA, Rus E, Dacier S, Meiland F. Utilization, effect, and benefit of the individualized Meeting Centers Support Program for people with dementia and caregivers. Clinical interventions in aging [Internet]. 2019; 14:[1527‐53 pp.]. Available from: https://www.cochranelibrary.com/central/doi/10.1002/central/CN-02077819/full. | |
| 658 | Rejected by TIAB Screening | Droste W. Interactive implants: Ethical, legal and social implications. Biomedizinische Technik. 2018;63 (Supplement 1):S6. | |
| 659 | Outside SDL | Drude KP, Maheu M, Hilty DM. Continuing Professional Development: Reflections on a Lifelong Learning Process. Psychiatric Clinics of North America. 2019;42(3):447-61. | |
| 660 | Formal Teaching | Drzaic M, Kummer I, Mucalo I, Bruno A, Ortner Hadziabdic M. Identifying self-assessed competencies and areas for improvement within community pharmacist-preceptors support during pre-registration training. BMC Medical Education. 2018;18(1):303. | |
| 661 | Rejected by TIAB Screening | Dua A, Rothenberg K, Lee JT. The role of industry in vascular surgery trainee education. Seminars in Vascular Surgery. 2019;32(1-2):30-2. | |
| 662 | Rejected by TIAB Screening | Dual PA, et al. Lifelong Learning and Public Health Professionals. Lifelong Learning: The Adult Years. 1980;3(8):16-9. | |
| 663 | Formal Teaching | Dubin SS. The psychology of lifelong learning: New developments in the professions. International Review of Applied Psychology. 1974;23(1):17-31. | |
| 664 | Rejected by TIAB Screening | Duffield C. A Master Class for nursing unit managers: an Australian example. Journal of Nursing Management. 2005;13(1):68-73. | |
| 665 | Outside SDL | Duffy FD, Holmboe ES. Self-assessment in lifelong learning and improving performance in practice: physician know thyself. JAMA. 2006;296(9):1137-9. | |
| 666 | Rejected by TIAB Screening | Duffy JR, Lemieux KG. A cardiac service line approach to patient-centered care. Nursing Administration Quarterly. 1995;20(1):12-23. | |
| 667 | Rejected by TIAB Screening | Duke VJA, Anstey A, Carter S, Gosse N, Hutchens KM, Marsh JA. Social media in nurse education: Utilization and E-professionalism. Nurse Education Today. 2017;57:8-13. | |
| 668 | Rejected by TIAB Screening | Dusch M, Brascher AK, Kopf A, Treede RD, Benrath J. [Medical educational evaluation of the German Pain Congress 2012. In the context of the CanMEDS physician competency framework]. Der Schmerz. 2014;28(5):520-7. | |
| 669 | Rejected by TIAB Screening | Dushyanthen S, Barrett M, Kok D, McArthur G. The development of a wholly online master's program for oncology clinicians. Journal of Global Oncology. 2018;4 (Supplement 2):9s. | |
| 670 | Rejected by TIAB Screening | Dushyanthen S, Barrett M, Mc Arthur G, Kok D. Adding colour to cancer: The art, science and education trifecta. Asia-Pacific Journal of Clinical Oncology. 2018;14 (Supplement 7):179. | |
| 671 | Not Target Group | Dworken HJ. Guided Self-Education in the Undergraduate Teaching of Gastroenterology. Journal of Medical Education. 1974;49(7):681-5. | |
| 672 | Rejected by TIAB Screening | Dwyer C, Durand H, MacNeela P, Reynolds B, Hamm R, Main C, et al. Effectiveness of a biopsychosocial e-learning intervention on the clinical judgements of medical students and GP trainees regarding future risk of disability in patients with chronic lower back pain: study protocol for a randomised controlled trial. BMJ open [Internet]. 2016; 6(5):[e010407 p.]. Available from: https://www.cochranelibrary.com/central/doi/10.1002/central/CN-01433491/full. | |
| 673 | Rejected by TIAB Screening | Dymock D. Editorial. Journal of Workplace Learning. 2007;19(4):1. | |
| 674 | Rejected by TIAB Screening | Dyson DH, Sparling SC. Delay in final publication following abstract presentation: American College of Veterinary Anesthesiologists annual meeting. Journal of Veterinary Medical Education. 2006;33(1):145-8. | |
| 675 | Rejected by TIAB Screening | Edelbring S, Dastmalchi M, Hult H, Lundberg IE, Dahlgren LO. Experiencing virtual patients in clinical learning: A phenomenological study. Advances in Health Sciences Education. 2011;16(3):331-45. | |
| 676 | Rejected by TIAB Screening | Edelsohn GA. Ethical dilemmas: What connects whole genome sequencing, management of psychogenic nonepileptic seizures, and psychiatrists' use of social media? Journal of the American Academy of Child and Adolescent Psychiatry. 2017;56 (10):S144. | |
| 677 | Rejected by TIAB Screening | Edler JR, Eberman LE. Factors Influencing Athletic Trainers&apos; Professional Development through Continuing Education. Athletic Training Education Journal. 2019;14(1):12-23. | |
| 678 | Rejected by TIAB Screening | Edrich T, Stopfkuchen-Evans M, Scheiermann P, Heim M, Chan W, Stone M, et al. A Comparison of Web-Based with Traditional Classroom-Based Training of Lung Ultrasound for the Exclusion of Pneumothorax. Anesthesia and analgesia [Internet]. 2016; 123(1):[123‐8 pp.]. Available from: https://www.cochranelibrary.com/central/doi/10.1002/central/CN-01382845/full. | |
| 679 | Rejected by TIAB Screening | Edstroem L-O. Correspondence Instruction in Ethiopia, Kenya, Tanzania, Malawi, Zambia, and Uganda--Experiences, Needs, and Interest. Dag Hammarskjold Foundation, Stockholm (Sweden). 1966. | |
| 680 | Not Target Group | Eduardo Fasce H, Cristhian Perez V, Liliana Ortiz M, Paula Parra P, Olga Matus B. Factorial structure and reliability of Fisher, King & Tague's self-directed learning readiness scale in Chilean medical students. Revista Medica de Chile. 2011;139(11):1428-34. | |
| 681 | Outside SDL | Edwards M. Lifelong learning in the NHS; Philosophy, funding and reality! Journal of Clinical Excellence. 2001;3(1):1-2. | |
| 682 | Rejected by TIAB Screening | Edwards RM, Cleland J, Bailey K, McLachlan S, McVey L-M. Pharmacist prescribers' written reflection on developing their consultation skills. Reflective Practice. 2009;10(4):437-50. | |
| 683 | Rejected by TIAB Screening | Eggermont M. Medical liability in peri-and postnatal care: The midwife and the obstetrician as partners in crime? International Journal of Gynecology and Obstetrics. 2015;131:E123. | |
| 684 | Rejected by TIAB Screening | Ehrlich E. Grass Roots Doctors. Change. 1978;10(9):16-9. | |
| 685 | Rejected by TIAB Screening | Ehrlich LM. Development of a staged orientation program in the centre for newborn care at the canberra hospital. Journal of Paediatrics and Child Health. 2010;46:66. | |
| 686 | Rejected by TIAB Screening | Eide T, Dulmen SV, Eide H. Educating for ethical leadership through web-based coaching. Nursing ethics. 2016;23(8):851-65. | |
| 687 | Not Target Group | Einspruch E, Lynch B, Aufderheide T, Nichol G, Becker L. Retention of CPR skills learned in a traditional AHA Heartsaver course versus 30-min video self-training: a controlled randomized study. Resuscitation [Internet]. 2007; 74(3):[476‐86 pp.]. Available from: https://www.cochranelibrary.com/central/doi/10.1002/central/CN-00617917/full. | |
| 688 | Rejected by TIAB Screening | Eisermann U, Haase I, Kladny B. Computer-aided multimedia training in orthopedic rehabilitation. American journal of physical medicine & rehabilitation [Internet]. 2004; 83(9):[670‐80 pp.]. Available from: https://www.cochranelibrary.com/central/doi/10.1002/central/CN-00480796/full. | |
| 689 | Rejected by TIAB Screening | Eklof M, Torner M, Pousette A. Organizational and social-psychological conditions in healthcare and their importance for patient and staff safety. A critical incident study among doctors and nurses. Safety Science. 2014;70:211-21. | |
| 690 | Not Target Group | El-Gilany AH, Abusaad Fe.l S. Self-directed learning readiness and learning styles among Saudi undergraduate nursing students. Nurse education today. 2013;33(9):1040-4. | |
| 691 | Rejected by TIAB Screening | Elbers N, Akkermans A, Cuijpers P, Bruinvels D. Effectiveness of a web-based intervention for injured claimants: a randomized controlled trial. Trials [Internet]. 2013; 14:[227 p.]. Available from: https://www.cochranelibrary.com/central/doi/10.1002/central/CN-01122454/full. | |
| 692 | Not Target Group | Elghawaby A, Siddiq K, Maarafiya M, Abdelrahman H, Hassan M, Alhammadi A. Perception and attitudes toward self-directed learning among pediatric residents and faculties in acgmei pediatric residency program-Qatar. Cogent Medicine Conference: 9th Excellence in Pediatrics Conference, EIP. 2017;4(1). | |
| 693 | Rejected by TIAB Screening | Elkjaer B, Nickelsen NCM. Intervention as Workplace Learning. Journal of Workplace Learning. 2016;28(5):266-79. | |
| 694 | Rejected by TIAB Screening | Ellaway R, Pusic M, Yavner S, Kalet A. Context matters: emergent variability in an effectiveness trial of online teaching modules. Medical education [Internet]. 2014; 48(4):[386‐96 pp.]. Available from: https://www.cochranelibrary.com/central/doi/10.1002/central/CN-01022989/full. | |
| 695 | Rejected by TIAB Screening | Ellis I, Walton G, Edwards C, Hutton A, Jones D. Living the Hylife: Addressing the information needs of physiotherapists. Physiotherapy. 2000;86(5):249-56. | |
| 696 | Rejected by TIAB Screening | Ellis LA. Capturing Nursing's Future Leaders. Journal of Professional Nursing. 1989;5(3):118. | |
| 697 | Rejected by TIAB Screening | Ellstrom E, Ellstrom P-E. Two Modes of Learning-Oriented Leadership: A Study of First-Line Managers. Journal of Workplace Learning. 2018;30(7):545-61. | |
| 698 | Rejected by TIAB Screening | ElSayed SK, Niculescu I. Vertical integration of basic science and clinical medicine during case based learning sessions for third year medical students. FASEB Journal Conference: Experimental Biology. 2017;31(1 Supplement 1). | |
| 699 | Not Target Group | Ely JWO, Jerome A. Ferguson, Kristi J. Chambliss, M. Vinson, Daniel C. Moore, Joyce L. Lifelong self-directed learning using computer database of clinical questions. The Journal of Family Practice. 1997;45(5):382-8. | |
| 700 | Outside SDL | Emblen JD, Gray GT. Comparison of nurses' self-directed learning activities. Journal of Continuing Education in Nursing. 1990;21(2):56-61. | |
| 701 | Rejected by TIAB Screening | Embo M, Helsloot K, Michels N, Valcke M. A Delphi study to validate competency-based criteria to assess undergraduate midwifery students' competencies in the maternity ward. Midwifery. 2017;53:1-8. | |
| 702 | Rejected by TIAB Screening | Embo M, Valcke M. Continuing midwifery education beyond graduation: Student midwives' awareness of continuous professional development. Nurse education in practice. 2017;24:118-22. | |
| 703 | Not Target Group | Embo MP, Driessen EW, Valcke M, Van der Vleuten CP. Assessment and feedback to facilitate self-directed learning in clinical practice of Midwifery students. Medical teacher. 2010;32(7):e263-9. | |
| 704 | Rejected by TIAB Screening | Emerson TF. Inside the VA: How Workplace Training Evaluation Impacts Employee Performance. ProQuest LLC EdD Dissertation, University of Missouri Saint Louis. 2017. | |
| 705 | Rejected by TIAB Screening | Engel-Smothers H, Heim SM. Boosting Your Baby's Brain Power. Great Potential Press, Inc. 2009. | |
| 706 | Not Target Group | Eom MR, Kim HS, Kim EK, Seong K. [Effects of teaching method using standardized patients on nursing competence in subcutaneous injection, self-directed learning readiness, and problem solving ability]. Journal of Korean Academy of Nursing. 2010;40(2):151-60. | |
| 707 | Rejected by TIAB Screening | Eom MR, Kyung Kim S, Park MH. Effectiveness of video recording and self-evaluation for skill training in undergraduate nursing education. Medico-Legal Update. 2020;20(1):2143-8. | |
| 708 | Rejected by TIAB Screening | Epes MT, et al. Developing New Models of the COMP-LAB College Basic Writing Course for Other Settings. Final Report. Reports - Evaluative. City Univ. of New York, Jamaica, NY. York Coll., City Univ. of New York Research Foundation, NY.; 1983. | |
| 709 | Rejected by TIAB Screening | Eppich WJ, Mullan PC, Brett-Fleegler M, Cheng A. "Let's Talk About It": Translating Lessons From Health Care Simulation to Clinical Event Debriefings and Coaching Conversations. Clinical Pediatric Emergency Medicine. 2016;17(3):200-11. | |
| 710 | Rejected by TIAB Screening | Eppich WJ, Rethans JJ, Dornan T, Teunissen PW. Learning how to learn using simulation: Unpacking disguised feedback using a qualitative analysis of doctors' telephone talk. Medical Teacher. 2018;40(7):661-7. | |
| 711 | Rejected by TIAB Screening | Epstein-Lubow G, Boland R, Cheong JA, Llorente MD. Training psychiatry residents and fellows in quality improvement: Geriatric psychiatry can help lead the way. American Journal of Geriatric Psychiatry. 2014;22(3):S34-S5. | |
| 712 | Rejected by TIAB Screening | Ericksen J, Milgrom J, Schembri C, Gemmill A. Towards parenthood. A public health intervention to prepare for the transition to parenthood. Archives of Women's Mental Health. 2013;16:S32. | |
| 713 | Not Target Group | Ertug N, Faydali S. Investigating the Relationship Between Self-Directed Learning Readiness and Time Management Skills in Turkish Undergraduate Nursing Students. Nursing Education Perspectives. 2018;39(2):E2-E5. | |
| 714 | Rejected by TIAB Screening | Escolar Chua RL, de Guzman AB. Do You See What I See? Understanding Filipino Elderly's Needs, Benefits, and Expectations from an Adult Continuing Education Program. Educational Gerontology. 2014;40(1):1-15. | |
| 715 | Rejected by TIAB Screening | Escolar Chua RL, de Guzman AB. Cogito Ergo Sum: A Grounded Theory of the Filipino Elderly Transition Experiences in Third Age Education Program. Educational Gerontology. 2018;44(7):433-46. | |
| 716 | Outside SDL | Escott ME. STAYING INFORMED. Help clinicians take control of the self-directed learning process. JEMS : a journal of emergency medical services. 2015;40(10):64. | |
| 717 | Rejected by TIAB Screening | Esteghamati A, Baradaran H, Monajemi A, Khankeh HR, Geranmayeh M. Core components of clinical education: a qualitative study with attending physicians and their residents. Journal of Advances in Medical Education & Professionalism. 2016;4(2):64-71. | |
| 718 | Rejected by TIAB Screening | Esterhuizen P. "The self-directed learning readiness of first year bachelor of nursing students": Comment. Journal of Research in Nursing. 2007;12(4):387-8. | |
| 719 | Rejected by TIAB Screening | Estus EL, Hume AL, Owens NJ. An active-learning course model to teach pharmacotherapy in geriatrics. American Journal of Pharmaceutical Education. 2010;74(3):38. | |
| 720 | Rejected by TIAB Screening | Ethans K, Deutscher T, Nankar M. The virtual hallway consult as an effective means of continuing professional development in physiatry. Canadian Medical Education Journal [Electronic Resource]. 2017;8(4):e60-e4. | |
| 721 | Rejected by TIAB Screening | Evans D. The patient-centered medical home as a curricular model: Medical students need an "educational home.". Academic Medicine. 2011;86(11):e2. | |
| 722 | Formal Teaching | Evelyn MN. The effect of gender, age, learning preferences, and environment on self-directed learning readiness: An exploratory case study of physician learner preferences. Dissertation Abstracts International Section A: Humanities and Social Sciences. 2012;72(8-A):2788. | |
| 723 | Accepted | Fahlman D. Examining Informal Learning Using Mobile Devices in the Healthcare Workplace. Canadian Journal of Learning and Technology. 2013;39(4). | |
| 724 | Rejected by TIAB Screening | Falcao LFDR, Ferez D, do Amaral JLG. Update on cardiopulmonary resuscitation guidelines of interest to anesthesiologists. Revista Brasileira de Anestesiologia. 2011;61(5):624-40. | |
| 725 | Rejected by TIAB Screening | Fan AP, Kosik RO, Huang L, Gjiang Y, Lien SS, Zhao X, et al. Burnout in Chinese medical students and residents: An exploratory cross-sectional study. The Lancet. 2017;390 (SPEC.ISS 1):84. | |
| 726 | Rejected by TIAB Screening | Fann JI, Sullivan ME, Skeff KM, Stratos GA, Walker JD, Grossi EA, et al. Teaching behaviors in the cardiac surgery simulation environment. Journal of Thoracic & Cardiovascular Surgery. 2013;145(1):45-53. | |
| 727 | Outside SDL | Farr A, Aliberti S, Loukides S, Massard G, Primhak R, Rohde GGU, et al. A pathway to keep all lifelong learners up to date: The ERS continuing professional development programme. European Respiratory Journal. 2020;55(2). | |
| 728 | Rejected by TIAB Screening | Farrugia A, Raul JS, Pelaccia T. How to supervise the learning of clinical reasoning among students during clinical rotations using the SNAPPS method? Description of the method and feedback from experience in an Institute of legal medicine. Revue de Medecine Legale. 2019;10(3):108-12. | |
| 729 | Not Target Group | Fasce HE, Ortega BJ, Ibanez GP, Marquez UC, Perez VC, Bustamante DC, et al. [Motivation and self-directed learning among medical students]. Revista Medica de Chile. 2016;144(5):664-70. | |
| 730 | Not Target Group | Fasce HE, Perez VC, Ortiz ML, Parra PP, Matus BO. [Factorial structure and reliability of Fisher, King & Tague's self-directed learning readiness scale in Chilean medical students]. Revista Medica de Chile. 2011;139(11):1428-34. | |
| 731 | Outside SDL | Faulkner LR. Recertification and maintenance of certification: Lifelong learning for psychiatrists and neurologists. The American Board of Psychiatry and Neurology: Looking back and moving ahead. Arlington, VA: American Psychiatric Publishing, Inc ; US; 2012. p. 283-300. | |
| 732 | Rejected by TIAB Screening | Fedele S. Permanent training in Italy, evidence based medicine's possible role: An analysis and proposal. [Italian]. Ricerca e Pratica. 2004;20(4):157-66. | |
| 733 | Rejected by TIAB Screening | Fejes A. Discourses on Employability: Constituting the Responsible Citizen. Studies in Continuing Education. 2010;32(2):89-102. | |
| 734 | Rejected by TIAB Screening | Felton N, Corse D, Stancombe C. Role-emerging physiotherapy placements in dementia care; a service improvement. Physiotherapy (United Kingdom). 2020;107 (Supplement 1):e189. | |
| 735 | Rejected by TIAB Screening | Ferguson C, DiGiacomo M, Saliba B, Green J, Moorley C, Wyllie A, et al. First year nursing students' experiences of social media during the transition to university: a focus group study. Contemporary nurse. 2016;52(5):625-35. | |
| 736 | Outside SDL | Ferguson KJ, Caplan RM. Physicians' Preferred Learning Methods and Sources of Information. Do Self-Identified Independent Learners Differ from Course Participants? Mobius. 1987;7(1):1-9. | |
| 737 | Rejected by TIAB Screening | Fernandes L, Basilio N, Figueira S, Nunes JM. [Mental Health in General Family Medicine - obstacles and expectations perceived by Family Physicians]. Ciencia & Saude Coletiva. 2017;22(3):797-805. | |
| 738 | Rejected by TIAB Screening | Ferozali F. Adult learning theory approaches among healthcare instructors. Dissertation Abstracts International Section A: Humanities and Social Sciences. 2011;72(5-A):1522. | |
| 739 | Rejected by TIAB Screening | Ferrier BM. Problem-Based Learning: Does It Make a Difference? Journal of Dental Education. 1990;54(9):550-51. | |
| 740 | Not Target Group | Filice S, Tregunno D, Edge D, Egan R. Re-imaging Clinical Education: The Interdependence of the Self-Regulated Clinical Teacher and Nursing Student. International journal of nursing education scholarship. 2020;17(1). | |
| 741 | Rejected by TIAB Screening | Fincher R, Abdulla A, Sridharan M, Houghton J, Henke J. Computer-assisted learning compared with weekly seminars for teaching fundamental electrocardiography to junior medical students. Southern medical journal [Internet]. 1988; 81(10):[1291‐4 pp.]. Available from: https://www.cochranelibrary.com/central/doi/10.1002/central/CN-00767694/full. | |
| 742 | Not Target Group | Findlater GS, Kristmundsdottir F, Parson SH, Gillingwater TH. Development of a supported self-directed learning approach for anatomy education. Anatomical sciences education. 2012;5(2):114-21. | |
| 743 | Not Target Group | Findley TW, DeLisa JA. Research in physical medicine and rehabilitation. XI. Research training: setting the stage for lifelong learning. American Journal of Physical Medicine & Rehabilitation. 1990;69(6):323-9. | |
| 744 | Rejected by TIAB Screening | Finlay K, Probyn L, Ho S. The CanMEDS resume: A useful educational portfolio tool for diagnostic radiology residents. Canadian Association of Radiologists Journal. 2012;63(4):233-6. | |
| 745 | Rejected by TIAB Screening | Finn K, Benes S, FitzPatrick K, Hardway C. Metacognition and Motivation in Anatomy and Physiology Students. International Journal of Teaching and Learning in Higher Education. 2019;31(3):476-90. | |
| 746 | Not Target Group | Fisher M, King J, Tague G. Development of a self-directed learning readiness scale for nursing education. Nurse Education Today. 2001;21(7):516-25. | |
| 747 | Not Target Group | Fitzgerald JT, Gruppen LD, White CB. The influence of task formats on the accuracy of medical students' self-assessments. Academic Medicine. 2000;75(7):737-41. | |
| 748 | Rejected by TIAB Screening | Flanagan B, Nestel D, Joseph M. Making patient safety the focus: crisis resource management in the undergraduate curriculum. Medical Education. 2004;38(1):56-66. | |
| 749 | Rejected by TIAB Screening | Flanagan M. Improving wound care teaching and learning in clinical practice. Wounds UK. 2008;4(4):96-101. | |
| 750 | Rejected by TIAB Screening | Flegar-Mestric Z, Gasljevic V, Borg C, Horvath A, Martin J, Salek T. Leonardo da vinci partnership project enhancing hospital laboratory standards for continuing professional development: A quality improvement toolkit. Clinical Chemistry and Laboratory Medicine. 2014;52:S733. | |
| 751 | Rejected by TIAB Screening | Fleischman RK, Meyer L, Watson C. Best practices in creating a culture of certification. AACN Advanced Critical Care. 2011;22(1):33-49. | |
| 752 | Rejected by TIAB Screening | Fletcher DM, Joanne. Toward a competency-based understanding of the training and development of applied sport psychologists. Sport, Exercise, and Performance Psychology. 2013;2(4):265-80. | |
| 753 | Rejected by TIAB Screening | Fletcher S. Supervision needs of nurses working in the community. International Journal of Palliative Nursing. 2008;14(4):196-200. | |
| 754 | Rejected by TIAB Screening | Flick CL. Stroke rehabilitation. 4. Stroke outcome and psychosocial consequences. Archives of Physical Medicine and Rehabilitation. 1999;80(5 SUPPL.):S21-S6. | |
| 755 | Rejected by TIAB Screening | Flojt JH, Ulrika Le Rosengren, Kristina. Need for preparedness: Nurses' experiences of competence in home health care. Home Health Care Management & Practice. 2014;26(4):223-9. | |
| 756 | Rejected by TIAB Screening | Fluit C, Bolhuis S, Grol R, Ham M, Feskens R, Laan R, et al. Evaluation and feedback for effective clinical teaching in postgraduate medical education: validation of an assessment instrument incorporating the CanMEDS roles. Medical Teacher. 2012;34(11):893-901. | |
| 757 | Rejected by TIAB Screening | Fong K, Rami-Porta R, Macbeth F, O'Rourke N, Westeel V, Pavlakis N, et al. Inaugural cochrane workshop. Journal of Thoracic Oncology. 2013;8:S125-S6. | |
| 758 | Rejected by TIAB Screening | Fontana M, Gonzalez-Cabezas C, de Peralta T, Johnsen DC. Dental Education Required for the Changing Health Care Environment. Journal of Dental Education. 2017;81(8):eS153-eS61. | |
| 759 | Rejected by TIAB Screening | Foran P. Undergraduate surgical nursing preparation and guided operating room experience: A quantitative analysis. Nurse education in practice. 2016;16(1):217-24. | |
| 760 | Not Target Group | Ford JDA, David B. Hawke, Josephine. Prevention and treatment interventions for traumatized children: Restoring children's capacities for self-regulation. Treating traumatized children: Risk, resilience and recovery. New York, NY: Routledge/Taylor & Francis Group; US; 2009. p. 195-209. | |
| 761 | Rejected by TIAB Screening | Foresi S, Putoto G. Health professionals as effective opinion makers and "global health advocates". The project "Equal opportunities for health: Action for development". Tropical Medicine and International Health. 2009;14:98. | |
| 762 | Rejected by TIAB Screening | Foronda C, Baptiste DL, Reinholdt MM, Ousman K. Cultural Humility: A Concept Analysis. Journal of transcultural nursing : official journal of the Transcultural Nursing Society. 2016;27(3):210-7. | |
| 763 | Rejected by TIAB Screening | Forrester S, Nelson M, McLeod S, Melady D. Understanding the expert approach to managing frailty in the emergency department. Canadian Journal of Emergency Medicine. 2019;21 (Supplement 1):S79-S80. | |
| 764 | Not Target Group | Forster C. Self-directed Learning in Pediatrics: Where Do We Go from Here? Pediatrics in Review. 2019;40(Suppl 1):37-8. | |
| 765 | Outside SDL | Foster CW. Institute of Medicine The Future of Nursing Report, lifelong learning, and certification. MEDSURG Nursing. 2012;21(2):115-6. | |
| 766 | Rejected by TIAB Screening | Foucault M-LV, Brigitte Thomas, Aliki Rochette, Annie Giguere, Charles-Edouard. Utilisation of an electronic portfolio to engage rehabilitation professionals in continuing professional development: Results of a provincial survey. Disability and Rehabilitation: An International, Multidisciplinary Journal. 2018;40(13):1591-9. | |
| 767 | Rejected by TIAB Screening | Fowler C, Dunston R, Lee A, Rossiter C, McKenzie J. Reciprocal Learning in Partnership Practice: An Exploratory Study of a Home Visiting Program for Mothers with Depression. Studies in Continuing Education. 2012;34(2):99-112. | |
| 768 | Rejected by TIAB Screening | Fowler P. Learning styles of radiographers. Radiography. 2002;8(1):3-11. | |
| 769 | Formal Teaching | Fox RD. New Research Agendas for CME: Organizing Principles for the Study of Self-Directed Curricula for Change. Journal of Continuing Education in the Health Professions. 1991;11(2):155-67. | |
| 770 | Rejected by TIAB Screening | Fox RDM, Paul E. Putnam, R. Wayne. Changing and learning in the lives of physicians. (1989) Changing and learning in the lives of physicians x, 194 pp New York, NY, England: Praeger Publishers; England1989. | |
| 771 | Rejected by TIAB Screening | Fox V, Blue MR. Job analysis. National Certification Board: Perioperative Nursing, Inc, document. AORN journal. 1988;47(5):1256-61, 63-69. | |
| 772 | Rejected by TIAB Screening | Fraher E, Brandt B. Toward a system where workforce planning and interprofessional practice and education are designed around patients and populations not professions. Journal of Interprofessional Care. 2019;33(4):389-97. | |
| 773 | Rejected by TIAB Screening | Fraher EP. The value of workforce data in shaping nursing workforce policy: A case study from North Carolina. Nursing Outlook. 2017;65(2):154-61. | |
| 774 | Not Target Group | Franchi T, Magudia A, Rasheed A. Appropriate use of self-directed learning at medical school prepares students for future clinical practice. Medical Education Online. 2020;25(1):1752450. | |
| 775 | Not Target Group | Frank A, Gifford K. Electronic portfolio use in pediatric residency and perceived efficacy as a tool for teaching lifelong learning. BMC Medical Education. 2017;17(1):202. | |
| 776 | Rejected by TIAB Screening | Frank JR, Choi S, Wiesenfeld L, Nussbaum C, Clark E, Weitzman B, et al. PROGRESSION of EM competence: Delphi method for derivation and validation of milestones. Canadian Journal of Emergency Medicine. 2010;12 (3):233. | |
| 777 | Rejected by TIAB Screening | Frantz J, Rowe M. The use of blogging in an evidence based practice postgraduate module. Physiotherapy (United Kingdom). 2011;97:eS364. | |
| 778 | Rejected by TIAB Screening | Fraser D, Murphy R, Worth-Butler M. An Outcome Evaluation of the Effectiveness of Pre-Registration Midwifery Programmes of Education. Reports - Research. English National Board for Nursing, Midwifery and Health Visiting, London.; 1997. | |
| 779 | Rejected by TIAB Screening | Frazier JA. 4.5 The How To's: The Use and Monitoring of Atypical Antipsychotics in Youth. Journal of the American Academy of Child and Adolescent Psychiatry. 2018;57 (10 Supplement):S126. | |
| 780 | Rejected by TIAB Screening | Freeman D, Reimer D, Khallouq BB, Hernandez C, Kauffman CA, Castiglioni A. Medical students' professional identity formation from pre-clerkship to clerkship years. Journal of General Internal Medicine. 2018;33 (2 Supplement 1):260. | |
| 781 | Rejected by TIAB Screening | Freeth D, Fry H. Nursing students' and tutors' perceptions of learning and teaching in a clinical skills centre. Nurse Education Today. 2005;25(4):272-82. | |
| 782 | Rejected by TIAB Screening | French F, Valentine M. An evaluation of the introduction of personal learning plans for general practitioners in Grampian Region (north-east Scotland). Education for General Practice. 2000;11(2):165-74. | |
| 783 | Rejected by TIAB Screening | French P, Cross D. Teaching and assessing on course for change. Nurse Education Today. 1991;11(1):19-23. | |
| 784 | Rejected by TIAB Screening | Frese M, Hefele B, Welch L, Gallo C, Grzybowski A. Technological advancements in CME increase knowledge gains and the translation of education to practice within cardiovascular care. Journal of Clinical Lipidology. 2019;13 (3):e33. | |
| 785 | Rejected by TIAB Screening | Freudenreich O, Huffman JC, Sharpe M, Beach SR, Celano CM, Chwastiak LA, et al. Updates in Psychosomatic Medicine: 2014. Psychosomatics. 2015;56(5):445-59. | |
| 786 | Rejected by TIAB Screening | Freyer-Adam J, Baumann S, Haberecht K, Tobschall S, Schnuerer I, Bruss K, et al. In-person and computer-based alcohol interventions at general hospitals: reach and retention. European journal of public health [Internet]. 2016; 26(5):[844‐9 pp.]. Available from: https://www.cochranelibrary.com/central/doi/10.1002/central/CN-01129287/full. | |
| 787 | Rejected by TIAB Screening | Fried M, Sadoughi B, Gibber M, Jacobs J, Lebowitz R, Ross D, et al. From virtual reality to the operating room: the endoscopic sinus surgery simulator experiment. Otolaryngology--head and neck surgery [Internet]. 2010; 142(2):[202‐7 pp.]. Available from: https://www.cochranelibrary.com/central/doi/10.1002/central/CN-00734928/full. | |
| 788 | Rejected by TIAB Screening | Fried MC, Hwang J, Liu S, Shapiro N, Trivedi SP. Core IM: A novel medical education podcast steeped in learning theory. Journal of General Internal Medicine. 2018;33 (2 Supplement 1):694-5. | |
| 789 | Rejected by TIAB Screening | Fried MC, Milburn S, Schlair S. Student as teacher: A student initiated, learner centered, fourth year medical school education elective. Journal of General Internal Medicine. 2015;30:S484. | |
| 790 | Rejected by TIAB Screening | Friedberg M, Mahanaimi D, Lev-Zion R, Sidi A, Glick S. Reading Habits of Third-Year Medical Students during an Integrated Endocrinology Course. Medical Teacher. 1998;20(2):133-37. | |
| 791 | Rejected by TIAB Screening | Frierson HT, Jr. Test-taking Intervention: Associated Effects on an Allied Health Certifying Examination. Journal of Allied Health. 1985;14(2):223-29. | |
| 792 | Rejected by TIAB Screening | Fry H, Davenport ES, Woodman T, Pee B. Developing Progress Files: A Case Study. Teaching in Higher Education. 2002;7(1):97-111. | |
| 793 | Rejected by TIAB Screening | Fujikura T, Nemoto T, Takayanagi K, Kashimura M, Hayasaka Y, Shimizu K. A freshman orientation program to provide an overview of the medical learning roadmap. Journal of Nippon Medical School. 2014;81(6):378-83. | |
| 794 | Not Target Group | Fujino-Oyama Y, Maeda R, Maru M, Inoue T. Validating the Japanese Self-Directed Learning Readiness Scale for Nursing Education. Journal of Nursing Education. 2016;55(2):65-71. | |
| 795 | Rejected by TIAB Screening | Fukuchi SG, Offutt LA, Sacks J, Mann BD. Teaching a multidisciplinary approach to cancer treatment during surgical clerkship via an interactive board game. American Journal of Surgery. 2000;179(4):337-40. | |
| 796 | Rejected by TIAB Screening | Fulford KWM, Handa A. Categorical and/or continuous? Learning from vascular surgery. World Psychiatry. 2018;17(3):304-5. | |
| 797 | Not Target Group | Fulkerson Z, Diab KJ, Carlos WG. Effectiveness of a self-directed critical care ultrasound course. American Journal of Respiratory and Critical Care Medicine Conference: American Thoracic Society International Conference, ATS. 2018;197(MeetingAbstracts). | |
| 798 | Outside SDL | Gaberson KB, Langston NF. Nursing as Knowledge Work: The Imperative for Lifelong Learning. AORN Journal. 2017;106(2):96-8. | |
| 799 | Rejected by TIAB Screening | Gabr A. Medical student E-library. Medical Education, Supplement. 2010;44:239. | |
| 800 | Rejected by TIAB Screening | Gabrilove JL, Ventura CD, Fattah L, Howell E, Fredericks M, Bloom L, et al. Learning to LEAD: Leadership emerging in academic departments. Journal of Clinical and Translational Science. 2018:58. | |
| 801 | Rejected by TIAB Screening | Gagnon M-PP-G, Julie Fortin, Jean-Paul Pare, Guy Cote, Jose Courcy, Francois. A learning organization in the service of knowledge management among nurses: A case study. International Journal of Information Management. 2015;35(5):636-42. | |
| 802 | Rejected by TIAB Screening | Gaidzinski RR, Peres HH, Fernandes MF. Leadership: continuous learning in the management in nursing. [Portuguese]. Revista brasileira de enfermagem. 2004;57(4):464-6. | |
| 803 | Rejected by TIAB Screening | Galve Villa M, Doody C. Physiotherapy students' and clinical tutors' perceptions of learning opportunities in relation to musculoskeletal clinical placements. Physiotherapy (United Kingdom). 2011;97:eS295-eS6. | |
| 804 | Rejected by TIAB Screening | Gamanya R, Stephen B. Integrating computer assisted instruction in traditional undergraduate dermatology promotes adult learning. British Journal of Dermatology. 2010;163:35-6. | |
| 805 | Rejected by TIAB Screening | Gamble C, Sin J, Kelly M, O'Loughlin D, Moone N. The development of a family intervention competency assessment and reflection scale (FICARS) for psychosis. Journal of Psychiatric and Mental Health Nursing. 2013;20(8):744-51. | |
| 806 | Rejected by TIAB Screening | Gamsu G, Perez E. Picture archiving and communication systems (PACS). Journal of Thoracic Imaging. 2003;18(3):165-8. | |
| 807 | Rejected by TIAB Screening | Gardner A, Schneider SM. The future of emergency medicine: Update 2011. Annals of Emergency Medicine. 2013;61(6):624-30. | |
| 808 | Rejected by TIAB Screening | Gardner AL, Clementz L, Lawrence RH, Dolansky MA, Heilman AM, Rusterholtz AR, et al. The Dyad Model for Interprofessional Academic Patient Aligned Care Teams. Federal Practitioner. 2019;36(2):88-93. | |
| 809 | Rejected by TIAB Screening | Gardner G, Sexton P, Guyer MS, Willeford KS, Levy LS, Barnum MG, et al. Clinical Instruction for Professional Practice. Athletic Training Education Journal. 2009;4(1):28-31. | |
| 810 | Rejected by TIAB Screening | Garino A. Ready, willing and able: a model to explain successful use of feedback. Advances in health sciences education : theory and practice. 2020;25(2):337-61. | |
| 811 | Rejected by TIAB Screening | Garland CW, et al. Caring for Infants and Toddlers with Disabilities: A Curriculum for Training Physicians in Early Intervention. Infants and Young Children. 1997;9(4):43-57. | |
| 812 | Rejected by TIAB Screening | Garland CW, Kniest BA. Caring for Infants and Toddlers with Disabilities: A Continuing Education Program for Nurses. Final Report. Reports - Research Tests/Questionnaires. Child Development Resources, Norge, VA.; 2002. | |
| 813 | Outside SDL | Garnet D, Sinner A, Walker C, Esmat R, Yi S. Learning in the Third Age: Drawing Wisdom from Reflective Stories in Community Art Education. International Journal of Lifelong Education. 2018;37(3):283-96. | |
| 814 | Rejected by TIAB Screening | Garten. Development of a Modularized and Mediated Course Design in Altered Physiological Systems: An Exercise in Faculty Development. Reports - Descriptive. 1979. | |
| 815 | Rejected by TIAB Screening | Garth PH. Every Voice Counts... Proceedings [of] the Annual African American and Latino/a American Adult Education Research Symposium (10th, Chicago, Illinois, April 21, 2001). Collected Works - Proceedings. 2001. | |
| 816 | Rejected by TIAB Screening | Gasparis PT, Kerridge WD, Gunderman RB. Service Learning in Radiology Education. Academic Radiology. 2017;24(4):514-5. | |
| 817 | Rejected by TIAB Screening | Gaston S, Goodwin M, Pucci J. Independent learning projects of registered nurses. Kansas Nurse. 1986;61(4):9-10. | |
| 818 | Rejected by TIAB Screening | Gaston SK, et al. Independent Learning Projects of Registered Nurses in a Mandatory Continuing Education State. Mobius. 1987;7(2):40-3. | |
| 819 | Rejected by TIAB Screening | Gates NJ, Rutjes AWS, Di Nisio M, Karim S, Chong LY, March E, et al. Computerised cognitive training for maintaining cognitive function in cognitively healthy people in midlife. Cochrane Database of Systematic Reviews. 2019(3). | |
| 820 | Rejected by TIAB Screening | Gawugah JNK, Jadva-Patel H, Jackson MT. The uptake of Continuing Professional Development (CPD) by Ghanaian radiographers. Radiography. 2011;17(4):332-44. | |
| 821 | Rejected by TIAB Screening | Gearhart S, Marohn M, Ngamruengphong S, Adrales G, Owodunni O, Duncan K, et al. Development of a train-to-proficiency curriculum for the technical skills component of the fundamentals of endoscopic surgery exam. Surgical Endoscopy. 2018;32(7):3070-5. | |
| 822 | Outside SDL | Gearon CJ. Back to school. Lifelong learning comes to health care. Hospitals & Health Networks. 2003;77(3):44-8, 2. | |
| 823 | Rejected by TIAB Screening | Geertsma RH, et al. An Independent Study Program Within a Medical Curriculum. Journal of Medical Education. 1977;52(2):123-32. | |
| 824 | Rejected by TIAB Screening | Genova J, Nahon-Serfaty I, Dansokho SC, Gagnon MP, Renaud JS, Giguere AM. The Communication AssessmenT Checklist in Health (CATCH): a tool for assessing the quality of printed educational materials for clinicians. The Journal of continuing education in the health professions. 2014;34(4):232-42. | |
| 825 | Rejected by TIAB Screening | Geoghegan SE, Clarke E, Byrne D, Power D, Moneley D, Strawbridge J, et al. Preparedness of newly qualified doctors in Ireland for prescribing in clinical practice. British Journal of Clinical Pharmacology. 2017;83(8):1826-34. | |
| 826 | Rejected by TIAB Screening | George P, Reis S, Dobson M, Nothnagle M. Using a learning coach to develop family medicine residents' goal-setting and reflection skills. Journal of Graduate Medical Education. 2013;5(2):289-93. | |
| 827 | Rejected by TIAB Screening | Gerke W, Goebel I, Bassler D. Teaching of evidence-based medicine and clinical neurology to undergraduate medical students: A pilot course. [German]. Medizinische Ausbildung. 2002;19(2):130-3. | |
| 828 | Rejected by TIAB Screening | Gershkoff AM, Cifu DX, Means KM. Geriatric rehabilitation. 1. Social, attitudinal, and economic factors. Archives of Physical Medicine & Rehabilitation. 1993;74(5-S):S402-5. | |
| 829 | Rejected by TIAB Screening | Ghaderi I, Auvergne L, Park YS, Farrell TM. Quantitative and qualitative analysis of performance during advanced laparoscopic fellowship: A curriculum based on structured assessment and feedback. American Journal of Surgery. 2015;209(1):71-8. | |
| 830 | Rejected by TIAB Screening | Ghaderi I, Park YS, Auvergne L, Farrell TM. Assessment of performance during advanced laparoscopic fellowship-a single subject design. Surgical Endoscopy and Other Interventional Techniques. 2014;28:327. | |
| 831 | Rejected by TIAB Screening | Ghahramanian A, Rezaei T, Abdullahzadeh F, Sheikhalipour Z, Dianat I. Quality of healthcare services and its relationship with patient safety culture and nurse-physician professional communication. Health Promotion Perspectives. 2017;7(3):168-74. | |
| 832 | Rejected by TIAB Screening | Gheorghe CM, Purcarea VL, Gheorghe IR, Popa-Velea O. Investigating the dimensions of learning organizations questionnaire (DLOQ) in a Romanian private ophthalmology organization. Romanian Journal of Ophthalmology. 2018;62(4):288-95. | |
| 833 | Rejected by TIAB Screening | Gheza F, Raimondi P, Solaini L, Coccolini F, Baiocchi GL, Portolani N, et al. Impact of one-to-one tutoring on fundamentals of laparoscopic surgery (FLS) passing rate in a single center experience outside the United States: a randomized controlled trial. Surgical Endoscopy. 2018;32(11):4428-35. | |
| 834 | Accepted | Ghiyasvandian S, Malekian M, Cheraghi MA. Iranian Clinical Nurses' Activities for Self-Directed Learning: A Qualitative Study. Global Journal of Health Science. 2015;8(5):48-58. | |
| 835 | Rejected by TIAB Screening | Gianakos D. Accepting limits. Archives of Internal Medicine. 1998;158(10):1059-61. | |
| 836 | Rejected by TIAB Screening | Gibbons V, Esselink T, McHugh S. Assessing practice relating to fall risk management among nurses in an acute ward setting: A best practice implementation report. JBI Database of Systematic Reviews and Implementation Reports. 2013;11(6):275-85. | |
| 837 | Rejected by TIAB Screening | Gibbs V. An investigation into the challenges facing the future provision of continuing professional development for allied health professionals in a changing healthcare environment. Radiography. 2011;17(2):152-7. | |
| 838 | Rejected by TIAB Screening | Gibson C. Educational tool for hospital-based training in family medicine. Canadian Family Physician. 2014;60(10):946-8 and e499-e501. | |
| 839 | Rejected by TIAB Screening | Gilbert GF. Mail Order CE: A Successful Experiment. Journal of Continuing Education in Nursing. 1983;14(5):16-21. | |
| 840 | Rejected by TIAB Screening | Gillespie C, Porter B, Horlick M, Hanley K, Adams J, Fox J, et al. Using osce cases to assess resident physicians' competence in inter-professional collaborative practice. Journal of General Internal Medicine. 2013;28:S235. | |
| 841 | Rejected by TIAB Screening | Gillespie H, Findlay White F, Kennedy N, Dornan T. Enhancing workplace learning at the transition into practice. Lessons from a pandemic. Medical education. 2020;16. | |
| 842 | Rejected by TIAB Screening | Gillis TA, Cheville AL, Worsowicz GM. Cardiopulmonary rehabilitation and cancer rehabilitation. 4. Oncologic rehabilitation. Archives of Physical Medicine and Rehabilitation. 2001;82(3 SUPPL. 1):S63-S8. | |
| 843 | Rejected by TIAB Screening | Ginzburg SB, Schwartz J, Gerber R, Deutsch S, Elkowitz DE, Ventura-Dipersia C, et al. Assessment of medical students' leadership traits in a problem/case-based learning program. Medical education online. 2018;23(1):1542923. | |
| 844 | Rejected by TIAB Screening | Glaser AY, Hall CB, Uribe SJ, Fried MP. Medical students' attitudes toward the use of an endoscopic sinus surgery simulator as a training tool. American Journal of Rhinology. 2006;20(2):177-9. | |
| 845 | Rejected by TIAB Screening | Glass N, Hanson G, Anger W, Laharnar N, Campbell J, Weinstein M, et al. Computer-based training (CBT) intervention reduces workplace violence and harassment for homecare workers. American journal of industrial medicine [Internet]. 2017; 60(7):[635‐43 pp.]. Available from: https://www.cochranelibrary.com/central/doi/10.1002/central/CN-01443352/full. | |
| 846 | Rejected by TIAB Screening | Glassman SJ, Rashbaum IG, Walker WC. Cardiopulmonary rehabilitation and cancer rehabilitation. 1. Cardiac rehabilitation. Archives of Physical Medicine and Rehabilitation. 2001;82(3 SUPPL. 1):S47-S51. | |
| 847 | Rejected by TIAB Screening | Glen S. Critique of the graduate nurse: An international perspective. Nurse Education Today. 2000;20(1):17-23. | |
| 848 | Formal Teaching | Glenn CA. A comparison of learner self-regulation in online and face-to-face problem-based learning courses. Dissertation Abstracts International Section A: Humanities and Social Sciences. 2015;76(6-A(E)):No Pagination Specified. | |
| 849 | Rejected by TIAB Screening | Gloster AS, Neville L, Windle J. Effects of advanced practitioners' learning in one hospital. Nursing Management (Harrow). 2015;21(10):23-30. | |
| 850 | Rejected by TIAB Screening | Godfrey EM, Kazmerski T, Thayer E, Brown G, Mentch L, Pam M, et al. Determining the needs of a patient-centered outcomes research training program for the cystic fibrosis community. Pediatric Pulmonology. 2019;54 (Supplement 2):462. | |
| 851 | Rejected by TIAB Screening | Goel A, Geraghty J, Pimblett M, Dickinson M, Shields PL. Human factors training (HFT) through simulation based scenarios in endoscopic haemostasis improves teamworking. Gut. 2015;64:A395. | |
| 852 | Not Target Group | Goeppinger J, Armstrong B, Schwartz T, Ensley D, Brady T. Self-management education for persons with arthritis: managing comorbidity and eliminating health disparities. Arthritis and rheumatism [Internet]. 2007; 57(6):[1081‐8 pp.]. Available from: https://www.cochranelibrary.com/central/doi/10.1002/central/CN-00610977/full. | |
| 853 | Rejected by TIAB Screening | Goff SL, Lagu T, Shatz AS, Pekow PS, Lindenauer PK. A qualitative analysis of hospital leaders opinions about publicly reported measures of health care quality. Journal of General Internal Medicine. 2014;29:S11. | |
| 854 | Outside SDL | Goin MK. Improving Supervisory Skills: An Exercise in Lifelong Learning. Psychotherapy supervision and consultation in clinical practice. Lanham, MD: Jason Aronson; US; 2006. p. 73-81. | |
| 855 | Rejected by TIAB Screening | Goldberg MR. Comparative effectiveness of online training assistive technology and its use for development of rehabilitation professionals' interprofessionality and reflectiveness. Dissertation Abstracts International: Section B: The Sciences and Engineering. 2014;75(6-B(E)):No Pagination Specified. | |
| 856 | Rejected by TIAB Screening | Goldman HM. Utilizing shared virtual microscopy slides to create innovative anatomical science resources in an integrated curriculum. FASEB Journal Conference: Experimental Biology. 2017;31(1 Supplement 1). | |
| 857 | Rejected by TIAB Screening | Goldman L, Miller J, Vangkonevilay P, Phrasisombath K, Vilay P, Sing M, et al. Master of family medicine distance learning program in Laos. Annals of Global Health. 2015;81 (1):28-9. | |
| 858 | Rejected by TIAB Screening | Goldman S, Barthman M, Merritt R. Reversing the script: Peer-based teaching of foundational concepts in emergency medicine using a foamed curriculum. Western Journal of Emergency Medicine. 2019;20 (4):S23. | |
| 859 | Rejected by TIAB Screening | Goldsworthy S, Lawrence N, Goodman W. The use of personal digital assistants at the point of care in an undergraduate nursing program. Computers, informatics, nursing [Internet]. 2006; 24(3):[138‐43 pp.]. Available from: https://www.cochranelibrary.com/central/doi/10.1002/central/CN-00556423/full. | |
| 860 | Formal Teaching | Goliath CL. Diffusion of an e-Portfolio to Assist in the Self-Directed Learning of Physicians: An Exploratory Study. ProQuest LLC PhD Dissertation, The University of Akron. 2009. | |
| 861 | Rejected by TIAB Screening | Gomez MP, Istrate MM, Peralta PP, Vera EE, Perales JJ, Manyalich M, et al. Organ donation innovative strategies for Southeast Asia: ODISSeA. Transplantation. 2019;103 (11 Supplement 1):S70. | |
| 862 | Rejected by TIAB Screening | Gonella S, Brugnolli A, Bosco A, Mansutti I, Canzan F, Randon G, et al. A Nationwide Italian Cross-sectional Study on Nursing Students' Perceived Workplace Safety During Clinical Practice. Nurse educator. 2020;25. | |
| 863 | Rejected by TIAB Screening | Gonella S, Brugnolli A, Terzoni S, Destrebecq A, Saiani L, Zannini L, et al. A national study of nursing homes as learning environments according to undergraduate nursing student's perspective. International Journal of Older People Nursing. 2019;14(3):e12245. | |
| 864 | Rejected by TIAB Screening | Gonzalez de la Aleja J, Martinez-Salio A, Bermejo-Pareja F. [Continuing medical education in neurology: a necessary challenge]. Neurologia. 2008;23(5):306-12. | |
| 865 | Rejected by TIAB Screening | Gonzalo JD, Wolpaw DR, Krok KL, Pfeiffer MP, McCall-Hosenfeld JS. A Developmental Approach to Internal Medicine Residency Education: Lessons Learned from the Design and Implementation of a Novel Longitudinal Coaching Program. Medical Education Online. 2019;24(1):1591256. | |
| 866 | Rejected by TIAB Screening | Goodson H, Puckeridge N, Cummins F. Developing a training programme for a de novo HEMS service in the UAE. EMA - Emergency Medicine Australasia. 2014;26:26-7. | |
| 867 | Rejected by TIAB Screening | Goodyear-Smith F. Practising alchemy: The transmutation of evidence into best health care. Family Practice. 2011;28(2):123-7. | |
| 868 | Rejected by TIAB Screening | Goos M, Brown R, Makar K. Navigating Currents and Charting Directions. Proceedings of the Annual Conference of the Mathematics Education Research Group of Australasia (31st, Brisbane, Queensland, Australia, June 28-July 1, 2008). Volumes 1 and 2. Mathematics Education Research Group of Australasia. 2008. | |
| 869 | Rejected by TIAB Screening | Gordon J, Mahabee-Gittens E, Andrews J, Christiansen S, Byron D. A randomized clinical trial of a web-based tobacco cessation education program. Pediatrics [Internet]. 2013; 131(2):[e455‐62 pp.]. Available from: https://www.cochranelibrary.com/central/doi/10.1002/central/CN-00850414/full. | |
| 870 | Rejected by TIAB Screening | Gorle K, McKechnie J. An educational initiative to help maintain competence of CCU nurses with infrequently seen procedures and situations. Canadian Journal of Cardiology. 2015;31(10):S326. | |
| 871 | Rejected by TIAB Screening | Gosling S. Physiotherapy and postgraduate study: A follow-up discussion paper. Physiotherapy. 1999;85(3):117-21. | |
| 872 | Rejected by TIAB Screening | Gostlow H, Marlow N, Babidge W, Maddern G. Systematic Review of Voluntary Participation in Simulation-Based Laparoscopic Skills Training: Motivators and Barriers for Surgical Trainee Attendance. Journal of Surgical Education. 2017;74(2):306-18. | |
| 873 | Rejected by TIAB Screening | Gould CE, Kok BC, Ma VK, Wetherell JL, Sudheimer K, Beaudreau SA. Video-Delivered Relaxation Intervention Reduces Late-Life Anxiety: A Pilot Randomized Controlled Trial. American Journal of Geriatric Psychiatry. 2019;27(5):514-25. | |
| 874 | Rejected by TIAB Screening | Goulding EA, Thomas K, Marshall T, Wheeler KI. Evaluation of the initial implementation of a multidisciplinary newborn resuscitation education program. Journal of Paediatrics and Child Health. 2014;50:12. | |
| 875 | Rejected by TIAB Screening | Graddy R, Galiatsatos P, Christmas C. The quality and importance of feedback in professional development of interns and residents. Journal of General Internal Medicine. 2014;29:S230-S1. | |
| 876 | Rejected by TIAB Screening | Graham A, Blain A, Gavin-Wear A. Educating hospital-based nurses: A different approach. Practical Diabetes International. 1998;15(4):103-4. | |
| 877 | Rejected by TIAB Screening | Graham HJ, Seabrook MA, Woodfield SJ. Structured packs for independent learning: A comparison of learning outcome and acceptability with conventional teaching. Medical Education. 1999;33(8):579-84. | |
| 878 | Rejected by TIAB Screening | Graham IS, Gleason AJ, Keogh GW, Paltridge D, Rogers IR, Walton M, et al. Australian Curriculum Framework for Junior Doctors. Medical Journal of Australia. 2007;186(S7):S14-9. | |
| 879 | Rejected by TIAB Screening | Grainger H, McGee D, Jones J, Connolly C, Howell C. Development of a UK intraoperative cell salvage education workbook. Transfusion Alternatives in Transfusion Medicine. 2009;1):29. | |
| 880 | Not Target Group | Grant VJ. Teaching Medical Students about Other Health Professionals: An Experiment in Self-directed Learning. Medical Teacher. 1987;9(3):271-74. | |
| 881 | Rejected by TIAB Screening | Grant WD, Mihai A, Scott J, Ko PY. Traditional ACLS vs. integrated simulationbased ACLS during an emergency medicine rotation for third year medical students. Academic Emergency Medicine. 2010;17:S27. | |
| 882 | Outside SDL | Gravatt ZB, Arroll B. What resources do Auckland general practitioners use for answering immediate clinical questions and for lifelong learning? Journal of Primary Health Care. 2010;2(2):100-4. | |
| 883 | Rejected by TIAB Screening | Gray A. Hospital based training. Vox Sanguinis. 2010;99:80. | |
| 884 | Rejected by TIAB Screening | Gray GE. Evaluating your performance of evidence-based medicine. How to practice evidence-based psychiatry: Basic principles and case studies. Arlington, VA: American Psychiatric Publishing, Inc ; US; 2010. p. 121-4. | |
| 885 | Rejected by TIAB Screening | Gray I. The NHS Policy Context. Continuing professional development for clinical psychologist: A practical handbook. Leicester, England: British Psychological Society; England; 2006. p. 23-46. | |
| 886 | Rejected by TIAB Screening | Gray K, Sim J. Factors in the development of clinical informatics competence in early career health sciences professionals in Australia: a qualitative study. Advances in Health Sciences Education. 2011;16(1):31-46. | |
| 887 | Rejected by TIAB Screening | Green CA, Kim EH, O'Sullivan PS, Chern H. Using Technological Advances to Improve Surgery Curriculum: Experience With a Mobile Application. Journal of Surgical Education. 2018;75(4):1087-95. | |
| 888 | Rejected by TIAB Screening | Green J, Wyllie A, Jackson D. Electronic portfolios in nursing education: a review of the literature. Nurse Education in Practice. 2014;14(1):4-8. | |
| 889 | Rejected by TIAB Screening | Green JS, et al. Continuing Education for the Health Professions. Developing, Managing, and Evaluating Programs for Maximum Impact on Patient Care1984. | |
| 890 | Rejected by TIAB Screening | Green ML, Reddy SG, Holmboe E. Teaching and evaluating point of care learning with an internet-based clinical-question portfolio. Journal of Continuing Education in the Health Professions. 2009;29(4):209-19. | |
| 891 | Rejected by TIAB Screening | Green RD, Schlairet MC. Moving toward heutagogical learning: Illuminating undergraduate nursing students' experiences in a flipped classroom. Nurse Education Today. 2017;49:122-8. | |
| 892 | Rejected by TIAB Screening | Green SM, Weaver M, Voegeli D, Fitzsimmons D, Knowles J, Harrison M, et al. The development and evaluation of the use of a virtual learning environment (Blackboard 5) to support the learning of pre-qualifying nursing students undertaking a human anatomy and physiology module. Nurse Education Today. 2006;26(5):388-95. | |
| 893 | Rejected by TIAB Screening | Green-Eide B. A Comparison of the Mandatory Continuing Education (MCE) Requirements of the Regulated Health Occupations in Minnesota 1991. | |
| 894 | Rejected by TIAB Screening | Greenberg KH, et al. University/School Partnerships in Research and Service: The Cognitive Enrichment Network National Follow through Educational Model (COGNET). Reports - Descriptive Reports - Evaluative Speeches/Meeting Papers. 1994. | |
| 895 | Rejected by TIAB Screening | Greenberg LW, Goldberg RM, Foley RP. Learning preference and personality type: their association in paediatric residents. Medical Education. 1996;30(4):307-11. | |
| 896 | Rejected by TIAB Screening | Greenberg M, Barr G, Rupp V, Patel N, Weaver K, Hamilton K, et al. Cardiopulmonary resuscitation prescription program: a pilot randomized comparator trial. Journal of emergency medicine [Internet]. 2012; 43(1):[166‐71 pp.]. Available from: https://www.cochranelibrary.com/central/doi/10.1002/central/CN-00971173/full. | |
| 897 | Rejected by TIAB Screening | Greenwood J. Critique of the graduate nurse: an international perspective. Nurse Education Today. 2000;20(1):17-23; discussion 4-9. | |
| 898 | Rejected by TIAB Screening | Gregor F. Teaching the patient with ischemic heart disease: a systemic approach to instructional design. Patient counselling and health education [Internet]. 1981; 3(2):[57‐62 pp.]. Available from: https://www.cochranelibrary.com/central/doi/10.1002/central/CN-00266914/full. | |
| 899 | Rejected by TIAB Screening | Gregor M, Shope J, Blow F, Maio R, Weber J, Nypaver M. Feasibility of using an interactive laptop program in the emergency department to prevent alcohol misuse among adolescents. Annals of emergency medicine [Internet]. 2003; 42(2):[276‐84 pp.]. Available from: https://www.cochranelibrary.com/central/doi/10.1002/central/CN-00439588/full. | |
| 900 | Rejected by TIAB Screening | Griffiths N, Spence K, Casey A. The development and implementation of an accredited neonatal nursing speciality transition program. Journal of Paediatrics and Child Health. 2013;49:114. | |
| 901 | Rejected by TIAB Screening | Grill CA, Gunnar, Jr. Wikstrom, Ewa Lindgren, Eva-Carin. Multiple balances in workplace dialogue: Experiences of an intervention in health care. Journal of Workplace Learning. 2015;27(4):267-81. | |
| 902 | Rejected by TIAB Screening | Grkovic I. Transition of the medical curriculum from classical to integrated: problem-based approach and Australian way of keeping academia in medicine. Croatian Medical Journal. 2005;46(1):16-20. | |
| 903 | Rejected by TIAB Screening | Grodin M, Johnson T, Acree J, Glaser B. Ophthalmic surgical training: a curriculum to enhance surgical simulation. Retina (philadelphia, pa) [Internet]. 2008; 28(10):[1509‐14 pp.]. Available from: https://www.cochranelibrary.com/central/doi/10.1002/central/CN-00667172/full. | |
| 904 | Not Target Group | Grover KS. The Self-Directed Learning Experience of Mothers Whose Child Has Had a Paediatric Stroke. International Journal of Lifelong Education. 2014;33(4):488-503. | |
| 905 | Rejected by TIAB Screening | Grzeskowiak LE, Thomas AE, To J, Reeve E, Phillips AJ. Enhancing continuing education activities using audience response systems: a single-blind controlled trial. The Journal of continuing education in the health professions. 2015;35(1):38-45. | |
| 906 | Rejected by TIAB Screening | Gu JA, Choi AS, Kim HG. Applying the havruta learning method to nursing education. Medico-Legal Update. 2019;19(1):653-8. | |
| 907 | Rejected by TIAB Screening | Guastello S, Jay K. Improving the patient experience through a comprehensive performance framework to evaluate excellence in person-centred care. BMJ Open Quality. 2019;8(4):e000737. | |
| 908 | Not Target Group | Guerrero AMS, Panchanathan SS, Wong EW, Dietrich LAJ, Khattab HM, Rabon L. Maintenance of education: Fostering lifelong learning. Academic Pediatrics. 2016;16 (6):e31. | |
| 909 | Rejected by TIAB Screening | Gummesson C, Nordmark E. How can we scaffold learner autonomy by innovative assessment activities? Physiotherapy (United Kingdom). 2011;97:eS1501-eS2. | |
| 910 | Outside SDL | Gumnit RJ. Practice issues in neurology: Patient care, practice income, and quality. CONTINUUM: Lifelong Learning in Neurology. 2010;16(3):246-9. | |
| 911 | Rejected by TIAB Screening | Gurman GM. Teaching the teacher-why is so important? Journal of Clinical Monitoring and Computing. 2012;26 (4):245-6. | |
| 912 | Rejected by TIAB Screening | Gurusamy KS, Vaughan J, Davidson BR. Formal education of patients about to undergo laparoscopic cholecystectomy. Cochrane Database of Systematic Reviews. 2014(2). | |
| 913 | Rejected by TIAB Screening | Gusic ME. Celebrating Education and Building the Community of Pediatric Educators. Academic Pediatrics. 2010;10(2):89-90. | |
| 914 | Rejected by TIAB Screening | Guy R, Pisani HR, Rich P, Leahy C, Mandarano G, Molyneux T. Less is more: development and evaluation of an interactive e-atlas to support anatomy learning. Anatomical sciences education. 2015;8(2):126-32. | |
| 915 | Rejected by TIAB Screening | Guy S, Kras-Dupuis A, Wolfe D. Implementation of a pressure ulcer prevention education best practice for persons with spinal cord injury. Journal of Spinal Cord Medicine. 2014;37 (5):617-8. | |
| 916 | Rejected by TIAB Screening | Gwele NS, Uys LR. Views of leading nurse educators regarding the comprehensive basic nursing programme. Curationis. 1995;18(1):5-10. | |
| 917 | Rejected by TIAB Screening | Gwynne CE, Khan A, Lawson T. Optimizing undergraduate student-patient interaction in a district general hospital. Rheumatology (United Kingdom). 2013;52:i114-i5. | |
| 918 | Rejected by TIAB Screening | Ha EH. Undergraduate nursing students' subjective attitudes to curriculum for Simulation-based objective structured clinical examination. Nurse education today. 2016;36:11-7. | |
| 919 | Rejected by TIAB Screening | Haass A. Quality control in stroke CCT/MRI procedures. [German]. Radiologe. 2005;45(5):404-11. | |
| 920 | Rejected by TIAB Screening | Haberko J, Kopaczynska Z, Talikowska E, Pawelczyk L, Jedrzejczak P. [Awareness of basic medical law standards among doctors and patients in Poland]. Przeglad Lekarski. 2008;65(5):237-40. | |
| 921 | Rejected by TIAB Screening | Hadamitzky C, Pabst R. Medical lymphology: Can Europe fulfill the need for special training in medicine? European Journal of Lymphology and Related Problems. 2009;20 (57):26. | |
| 922 | Rejected by TIAB Screening | Haden NK, Andrieu SC, Chadwick DG, Chmar JE, Cole JR, George MC, et al. The dental education environment. Journal of dental education. 2006;70(12):1265-70. | |
| 923 | Not Target Group | Hadid S. Factors Influencing Nursing Student Self-Assessment in Relation to Instructor Assessment. Journal of Nursing Education. 2017;56(2):70-6. | |
| 924 | Rejected by TIAB Screening | Hadley JK, R Zamora, J Coppus, Sf Weinbrenner, S Meyerrose, B Decsi, T Horvath, Ar Nagy, E Emparanza, Ji et al.,. Effectiveness of an e-learning course in evidence-based medicine for foundation (internship) training. Journal of the royal society of medicine [Internet]. 2010; 103(7):[288‐94 pp.]. Available from: https://www.cochranelibrary.com/central/doi/10.1002/central/CN-00749251/full. | |
| 925 | Rejected by TIAB Screening | Hagan TL, Arida JA, Hughes SH, Donovan HS. Creating Individualized Symptom Management Goals and Strategies for Cancer-Related Fatigue for Patients With Recurrent Ovarian Cancer. Cancer Nursing. 2017;40(4):305-13. | |
| 926 | Rejected by TIAB Screening | Hager P, Beckett D. Making Judgments as the Basis for Workplace Learning: Preliminary Research Findings. Working Paper. Reports - Research. Technology Univ.-Sydney, Broadway (Australia). Research Centre for Vocational Education and Training.; 1999. | |
| 927 | Rejected by TIAB Screening | Hagg-Martinell A, Hult H, Henriksson P, Kiessling A. Possibilities for interprofessional learning at a Swedish acute healthcare ward not dedicated to interprofessional education: An ethnographic study. BMJ Open. 2019;9(7). | |
| 928 | Rejected by TIAB Screening | Haggqvist B, Stenvall M, Fjellman-Wiklund A, Westerberg K, Lundin-Olsson L. "The balancing act"--licensed practical nurse experiences of falls and fall prevention: a qualitative study. BMC Geriatrics. 2012;12:62. | |
| 929 | Rejected by TIAB Screening | Haggstrom M, Bergsman AC, Mansson U, Holmstrom MR. Learning to manage vasoactive drugs-A qualitative interview study with critical care nurses. Intensive & Critical Care Nursing. 2017;39:1-8. | |
| 930 | Rejected by TIAB Screening | Hakim A, Gibson R, Johansson L. Problem-based learning as a tool in postgraduate medical education. European Respiratory Journal Conference: European Respiratory Society International Congress, ERS. 2017;50(Supplement 61). | |
| 931 | Rejected by TIAB Screening | Hales DJ, Rapaport MH. Focus Psychiatry Review (Vol 2): A workbook covering the ABPN outline of topics for recertification: Anxiety disorders; child and adolescent psychiatry; disorders of sleep, eating, and sex; ethics and professionalism; genetics and genomics; geriatric psychiatry; mood disorders; obsessive-compulsive disorder; personality and temperament; posttraumatic stress disorder and disaster psychiatry; psychopharmacology; psychosomatic medicine; psychotherapy; schizophrenia; substance abuse. Arlington, VA: American Psychiatric Association; US; 2011. | |
| 932 | Rejected by TIAB Screening | Hales DJ, Rapaport MH. FOCUS psychiatry review: DSM-5. Vol. 2, Revised edition. Arlington, VA: American Psychiatric Association; US; 2015. | |
| 933 | Rejected by TIAB Screening | Hales DJR, Mark Hyman. FOCUS psychiatry review: 400 self-assessment questions. Arlington, VA: American Psychiatric Association; US; 2007. | |
| 934 | Not Target Group | Haley HB. Does medical school instill lifelong learning. Journal of Cancer Education. 2008;23(3):197. | |
| 935 | Rejected by TIAB Screening | Hall C, Peleva E, Vithlani RH, Shah S, Bashyam M, Ramadas M, et al. FEEDBK: a novel approach for providing feedback. The clinical teacher. 2020;17(1):76-80. | |
| 936 | Not Target Group | Hall J. Staged self-directed learning model: Leaving the nest-From novice to professional. Building sustainable futures for adult learners. Charlotte, NC: IAP Information Age Publishing; US; 2015. p. 325-39. | |
| 937 | Rejected by TIAB Screening | Halling-Brown M, Patel M, Matthew W, Young K. Development of a National Electronic Interval Cancer Review for breast screening. International Journal of Computer Assisted Radiology and Surgery. 2018;13 (Supplement 1):S142-S3. | |
| 938 | Rejected by TIAB Screening | Hameed Y, Wesby R, Wagle S, Agius S, Hillam J, Tarbuck A. Improving medical student placements in psychiatry: Review of literature and a practical example. British Journal of Medical Practitioners. 2017;10(1). | |
| 939 | Not Target Group | Hamilton L, Gregor F. Self-directed learning in a critical care nursing program. Journal of Continuing Education in Nursing. 1986;17(3):94-9. | |
| 940 | Rejected by TIAB Screening | Hamilton M. Setting up an in-office independent medical examination company. Clinics in Sports Medicine. 2002;21(2):289-303. | |
| 941 | Rejected by TIAB Screening | Hammer B, Sterling M, Stromack J, Brown L. Going the extra "mILE": Continuing education for MICU nurses. Nursing Critical Care. 2019;14(3):39-43. | |
| 942 | Rejected by TIAB Screening | Hammond C. Learning To Be Healthy. The Wider Benefits of Learning Papers. Information Analyses. London Univ. (England). Centre for Research on the Wider Benefits of Learning.; 2002. | |
| 943 | Rejected by TIAB Screening | Han ER, Yeo S, Kim MJ, Lee YH, Park KH, Roh H. Medical education trends for future physicians in the era of advanced technology and artificial intelligence: an integrative review. BMC Medical Education. 2019;19(1):460. | |
| 944 | Rejected by TIAB Screening | Han JJ, Brooks AD. How can we be more "deliberate" in training surgeons? American Journal of Surgery. 2018;216(2):359-60. | |
| 945 | Rejected by TIAB Screening | Hanna DR, Flanagan J. The fundamental unity of knowledge. Research and Theory for Nursing Practice: An International Journal. 2018;32(1):6-8. | |
| 946 | Rejected by TIAB Screening | Hanson J. Surveying the experiences and perceptions of undergraduate nursing students of a flipped classroom approach to increase understanding of drug science and its application to clinical practice. Nurse Education in Practice. 2016;16(1):79-85. | |
| 947 | Rejected by TIAB Screening | Hardiman M, Dewing J. Using two models of workplace facilitation to create conditions for development of a person-centred culture: A participatory action research study. Journal of Clinical Nursing. 2019;28(15-16):2769-81. | |
| 948 | Rejected by TIAB Screening | Harding KE, Porter J, Horne-Thompson A, Donley E, Taylor NF. Not enough time or a low priority? Barriers to evidence-based practice for allied health clinicians. The Journal of continuing education in the health professions. 2014;34(4):224-31. | |
| 949 | Rejected by TIAB Screening | Harding PA, Pearce A. Advanced musculoskeletal physiotherapy in public hospitals: Utilizing a competency based training and assessment approach. Physiotherapy (United Kingdom). 2015;101:eS1184. | |
| 950 | Rejected by TIAB Screening | Hardy DL. Learning strategies and motivational patterns, as measured by the motivated strategies for learning questionnaire, among students pursuing nursing and allied health careers. Dissertation Abstracts International Section A: Humanities and Social Sciences. 2015;76(5-A(E)):No Pagination Specified. | |
| 951 | Rejected by TIAB Screening | Hardyman W, Bullock A, Brown A, Carter-Ingram S, Stacey M. Mobile technology supporting trainee doctors' workplace learning and patient care: an evaluation. BMC Medical Education. 2013;13:6. | |
| 952 | Rejected by TIAB Screening | Harmse B, Retief I. Striving for excellence: Investigating the practical aspects of dietetic practice. South African Journal of Clinical Nutrition. 2015;28(2):89-91. | |
| 953 | Rejected by TIAB Screening | Harnett N. Can Advanced Practice Radiation Therapists improve access to radiotherapy? Radiotherapy and Oncology. 2018;127 (Supplement 1):S31. | |
| 954 | Outside SDL | Harney K, Heikkinen A, Rahn S, Schemmann M. Lifelong Learning: One Focus, Different Systems. Studien zur Erwachsenenbildung, Band 192002. | |
| 955 | Rejected by TIAB Screening | Harrington SW, Bl. A comparison of computer-based and instructor-led training for long-term care staff. Journal of continuing education in nursing [Internet]. 2002; 33(1):[39‐45 pp.]. Available from: https://www.cochranelibrary.com/central/doi/10.1002/central/CN-00515472/full. | |
| 956 | Rejected by TIAB Screening | Harrington SW, Bl. Is computer-based instruction an effective way to present fire safety training to long-term care staff? Journal for nurses in staff development [Internet]. 2003; 19(3):[147‐54 pp.]. Available from: https://www.cochranelibrary.com/central/doi/10.1002/central/CN-00438194/full. | |
| 957 | Rejected by TIAB Screening | Harrington SW, Bl. The effects of computer-based training on immediate and residual learning of nursing facility staff. Journal of continuing education in nursing [Internet]. 2004; 35(4):[154‐63; quiz 86‐7 pp.]. Available from: https://www.cochranelibrary.com/central/doi/10.1002/central/CN-00481447/full. | |
| 958 | Rejected by TIAB Screening | Harrington SW, Bl. Teaching ergonomics to nursing facility managers using computer-based instruction. Journal for nurses in staff development [Internet]. 2006; 22(5):[260‐8 pp.]. Available from: https://www.cochranelibrary.com/central/doi/10.1002/central/CN-00572620/full. | |
| 959 | Rejected by TIAB Screening | Harrington SW, Bl. The effects of computer-based fire safety training on the knowledge, attitudes, and practices of caregivers. Journal of continuing education in nursing [Internet]. 2009; 40(2):[79‐86 pp.]. Available from: https://www.cochranelibrary.com/central/doi/10.1002/central/CN-00682825/full. | |
| 960 | Formal Teaching | Harrison C Mb CPF. Can we redesign the MRCGP assessment to support lifelong learning? Education for Primary Care. 2019;30(1):9-12. | |
| 961 | Rejected by TIAB Screening | Hart G. Peer consultation and review. Australian Journal of Advanced Nursing. 1990;7(2):40-6. | |
| 962 | Not Target Group | Hartman ND, Harper EN, Leppert LM, Browning BM, Askew K, Manthey DE, et al. A Multidisciplinary Self-Directed Learning Module Improves Knowledge of a Quality Improvement Instrument: The HEART Pathway. Journal for Healthcare Quality. 2018;40(1):e9-e14. | |
| 963 | Rejected by TIAB Screening | Hartmeier A. Challenges of teaching and learning clinical reasoning in undergraduate pre-clinical education. Physiotherapy (United Kingdom). 2011;97:eS456-eS7. | |
| 964 | Rejected by TIAB Screening | Hartzheim L, Hanson C, Lathrop C, Hodnefield J, Howard M, Miller M, et al. Training physician extenders in the clinical practice of diagnostic hematopathology: A novel approach to building expertise and improving quality and efficiency. Modern Pathology. 2020;33 (3):533-5. | |
| 965 | Rejected by TIAB Screening | Haruta J, Yamamoto Y. Realist approach to evaluating an interprofessional education program for medical students in clinical practice at a community hospital. Medical teacher. 2020;42(1):101-10. | |
| 966 | Rejected by TIAB Screening | Hasan M, Meara RJ, Bhowmick BK, Woodhouse KW. Continuing medical education in Wales: A survey of geriatricians. Age and Ageing. 1997;26(4):309-13. | |
| 967 | Rejected by TIAB Screening | Hashemi N, Chan WW, Bennert E. Interactive online liver modules increase knowledge of chronic liver disease among gastroenterology fellows. Gastroenterology. 2017;152 (5 Supplement 1):S231. | |
| 968 | Rejected by TIAB Screening | Hashim MJ. Principles of family medicine and general practice - defining the five core values of the specialty. Journal of Primary Health Care. 2016;8(4):283-7. | |
| 969 | Rejected by TIAB Screening | Hashmi S. A day in the life of. Archives of Disease in Childhood. 2019;104 (Supplement 4):A42. | |
| 970 | Rejected by TIAB Screening | Hasske E, Beil M, Keller K. Competency-based Education and Training of medical staff. A Programm of the Medical Academy Waldbreitbach: Concept - Implementation - Materials. GMS journal for medical education. 2017;34(4):Doc41. | |
| 971 | Rejected by TIAB Screening | Hasu M, Honkaniemi L, Saari E, Mattelmaki T, Koponen L. Learning employee-driven innovating: Towards sustained practice through multi-method evaluation. Journal of Workplace Learning. 2014;26(5):310-30. | |
| 972 | Rejected by TIAB Screening | Hathaway R, Saravanan Y, McShane M, Jain P, Bor D, Pels R. Innovations in medical education (IME) 'medical detective rounds': Adopting case-based collaborative learning in the clinicalyear of medical school to promote self directed learning and clinical reasoning. Journal of General Internal Medicine. 2018;33 (2 Supplement 1):673-4. | |
| 973 | Rejected by TIAB Screening | Hauer E, Westerberg K, Nordlund AM. Intervening with Care--Varying Outcomes of a Training and Development Programme in Elderly Care in Sweden. Journal of Vocational Education and Training. 2017;69(4):617-36. | |
| 974 | Rejected by TIAB Screening | Hautz SC, Hautz WE, Feufel MA, Spies CD. What makes a doctor a scholar: a systematic review and content analysis of outcome frameworks. BMC Medical Education. 2016;16:119. | |
| 975 | Rejected by TIAB Screening | Hawkins RE, Weiss KB. Commentary: Building the evidence base in support of the American Board of Medical Specialties maintenance of certification program. Academic Medicine. 2011;86(1):6-7. | |
| 976 | Rejected by TIAB Screening | Hayenga ES, Isaacson HB. Competence-Based Education for Adult Learners. New Directions for Community Colleges n29 p39. 1980;46. | |
| 977 | Rejected by TIAB Screening | Hayes K. Randomized trial of geragogy-based medication instruction in the emergency department. Nursing research [Internet]. 1998; 47(4):[211‐8 pp.]. Available from: https://www.cochranelibrary.com/central/doi/10.1002/central/CN-00686859/full. | |
| 978 | Rejected by TIAB Screening | Heaslip P. Creating the Thinking Practitioner: Critical Thinking in Clinical Practice. Opinion Papers. 1992. | |
| 979 | Not Target Group | Heath LA. The use of self-directed learning during fieldwork education: The students' perspective. The British Journal of Occupational Therapy. 1996;59(11):515-9. | |
| 980 | Rejected by TIAB Screening | Heflin MT. The Senior Mentor Program at Duke University School of Medicine. Gerontology and Geriatrics Education. 2006;27(2):49-58. | |
| 981 | Not Target Group | Hegge M. A model for continuing nursing education through self-directed learning. Journal of Continuing Education in Nursing. 1985;16(6):205-13. | |
| 982 | Rejected by TIAB Screening | Henderson A, Creedy D, Boorman R, Cooke M, Walker R. Development and psychometric testing of the Clinical Learning Organisational Culture Survey (CLOCS). Nurse Education Today. 2010;30(7):598-602. | |
| 983 | Not Target Group | Hendricson WD, Andrieu SC, Chadwick DG, Chmar JE, Cole JR, George MC, et al. Educational strategies associated with development of problem-solving, critical thinking, and self-directed learning. Journal of dental education. 2006;70(9):925-36. | |
| 984 | Rejected by TIAB Screening | Henry D, Vesel T, Boscardin C, van Schaik S. Motivation for feedback-seeking among pediatric residents: a mixed methods study. BMC Medical Education. 2018;18(1):145. | |
| 985 | Rejected by TIAB Screening | Herbek G. Education and training in laboratory medicine in the United States. Clinical Chemistry and Laboratory Medicine. 2014;52 (11):eA211. | |
| 986 | Rejected by TIAB Screening | Hermayer KL. The Diabetes Initiative of South Carolina Celebrates Over 20 Years of Professional Diabetes Education. The American journal of the medical sciences. 2016;351(4):325-6. | |
| 987 | Rejected by TIAB Screening | Hernández-Padilla JS, F Granero-Molina, J Fernández-Sola, C. Effects of two retraining strategies on nursing students' acquisition and retention of BLS/AED skills: a cluster randomised trial. Resuscitation [Internet]. 2015; 93:[27‐34 pp.]. Available from: https://www.cochranelibrary.com/central/doi/10.1002/central/CN-01257917/full. | |
| 988 | Rejected by TIAB Screening | Heru AM. Teaching Psychosomatic Medicine Using Problem-Based Learning and Role-Playing. Academic Psychiatry. 2011;35(4):245-8. | |
| 989 | Rejected by TIAB Screening | Heuberger R, Clark A, Logomarsino J, Shayna V, Hewlings S, Duriancik D. Synchronous classrooms: Teaching practicing nutrition clinicians through distance technologies. FASEB Journal Conference: Experimental Biology. 2016;30(Meeting Abstracts). | |
| 990 | Not Target Group | Hewitt-Taylor J. Self-directed learning: views of teachers and students. Journal of Advanced Nursing. 2001;36(4):496-504. | |
| 991 | Not Target Group | Hewitt-Taylor J. Teachers' and students' views on self-directed learning. Nursing Standard. 2002;17(1):33-8. | |
| 992 | Rejected by TIAB Screening | Heydari A, Moghaddam KB, Manzari ZS, Mahram B. The barriers of unlearning for nurses employed in hospitals: A qualitative study. Annals of Tropical Medicine and Public Health. 2018(3.3 Special Issue):SP57. | |
| 993 | Rejected by TIAB Screening | Heyns M. A strategy towards professionalism in the dissecting room. European Journal of Anatomy. 2007;11(SUPPL. 1):85-8. | |
| 994 | Rejected by TIAB Screening | Hicks RW, Patterson R. Navigating Nursing Education. AORN Journal. 2017;106(6):523-33. | |
| 995 | Rejected by TIAB Screening | Hidalgo A, Suarez S, Revuelta L, Diez B, Menendez L, Cantabrana B. Press news and pharmaceutical publicity in the teaching-learning process of pharmacology. European Journal of Clinical Pharmacology. 2010;66:S127. | |
| 996 | Rejected by TIAB Screening | Higgins M, Suddaby D, Coates J, Waterhouse M. Traditional didactic education vs web based self directed learning: Which works better to improve nurses knowledge of chest drain management? Heart. 2010;96:A18-A9. | |
| 997 | Rejected by TIAB Screening | Hilgers J, De Roos P. European core curriculum - The students' perspective, Bristol, UK, 10 July 2006. Medical Teacher. 2007;29(2-3):270-5. | |
| 998 | Accepted | Hill EK. Information seeking as self-directed learning in the lives of rural practitioners: A multi-case study. Dissertation Abstracts International Section A: Humanities and Social Sciences. 2011;71(7-A):2360. | |
| 999 | Rejected by TIAB Screening | Hill J, Rolfe IE, Pearson SA, Heathcote A. Do junior doctors feel they are prepared for hospital practice? A study of graduates from traditional and non-traditional medical schools. Medical Education. 1998;32(1):19-24. | |
| 1000 | Outside SDL | Hill, L. (2010). Self-directed learning activities that rural healthcare workers undertake through practice-based clinical decision-making for patient care. | |
| 1001 | Rejected by TIAB Screening | Hills J, Bar TD. Communications. Supervisory Management Module. Operational Management Programme. Second Edition. Guides - Classroom - Learner. Hotel and Catering Training Co., London (England). 1991. | |
| 1002 | Rejected by TIAB Screening | Hilty DM, Uno J, Chan S, Torous J, Boland RJ. Role of Technology in Faculty Development in Psychiatry. Psychiatric Clinics of North America. 2019;42(3):493-512. | |
| 1003 | Rejected by TIAB Screening | Hintz SS. Real Problems and Real Solutions: A Humanities/Foreign Language Component in Occupational/Technical Curricula. Reports - Descriptive Speeches/Meeting Papers. 1995. | |
| 1004 | Rejected by TIAB Screening | Hirai M. Development of strong cooperation between community pharmacists, hospital pharmacists and pharmacy school faculty members which promotes pharmacists' life long learning and clinical clerkship for pharmacy students. [Japanese]. Yakugaku Zasshi. 2007;127(2):285-90. | |
| 1005 | Rejected by TIAB Screening | Hirsch KA. The Impact of Differentiated Instructional Techniques on Non-Traditional, Adult Student Engagement in a Baccalaureate Nursing Completion Program for Registered Nurses (RN-BSN) Course. ProQuest LLC EdD Dissertation, Ball State University. 2013. | |
| 1006 | Rejected by TIAB Screening | Hlongwa EN, Sibiya MN. Challenges affecting the implementation of the Policy on Integration of Mental Health Care into primary healthcare in KwaZulu-Natal province. Curationis. 2019;42(1):e1-e9. | |
| 1007 | Rejected by TIAB Screening | Hlushak C, Acharya H, Ashworth N. Current practice management knowledge in a physical medicine and rehabilitation residency program. PM and R. 2011;3(10):S319-S20. | |
| 1008 | Rejected by TIAB Screening | Hmelo CE, et al. The Cognitive Effects of Problem-Based Learning: A Preliminary Study. Reports - Research Speeches/Meeting Papers. 1994. | |
| 1009 | Rejected by TIAB Screening | Hoad-Reddick G, Theaker E. Providing support for problem-based learning in dentistry: the Manchester experience. European Journal of Dental Education. 2003;7(1):3-12. | |
| 1010 | Rejected by TIAB Screening | Hobma SO, Ram PM, van Merode F, van der Vleuten CPM, Grol RPTM. Feasibility, appreciation and costs of a tailored continuing professional development approach for general practitioners. Quality in Primary Care. 2004;12(4):271-8. | |
| 1011 | Rejected by TIAB Screening | Hoffmann HF. ESI's scientific schools: A privileged place for knowledge transfer. Radiotherapy and Oncology. 2014;110:S43. | |
| 1012 | Rejected by TIAB Screening | Hoffmann S, Dreher-Hummel T, Dollinger C, Frei IA. Patient complaints as a means to improve quality of hospital care. Results of a qualitative content analysis. Pflege. 2018;31(2):101-9. | |
| 1013 | Rejected by TIAB Screening | Hogg S, Roe Y, Mills R. Implementing evidence-based continuous quality improvement strategies in an urban Aboriginal Community Controlled Health Service in South East Queensland: a best practice implementation pilot. JBI Database Of Systematic Reviews And Implementation Reports. 2017;15(1):178-87. | |
| 1014 | Rejected by TIAB Screening | Holder B. An Investigation of Hope, Academics, Environment, and Motivation as Predictors of Persistence in Higher Education Online Programs. Internet and Higher Education. 2007;10(4):245-60. | |
| 1015 | Rejected by TIAB Screening | Holder BA. An investigation of hope, academics, environment, and motivation predictors of persistence in higher education online programs. Dissertation Abstracts International Section A: Humanities and Social Sciences. 2007;68(1-A):49. | |
| 1016 | Rejected by TIAB Screening | Holdsworth CS, Eh Delany, Cm. Using simulation pedagogy to teach clinical education skills: a randomized trial. Physiotherapy theory and practice [Internet]. 2016; 32(4):[284‐95 pp.]. Available from: https://www.cochranelibrary.com/central/doi/10.1002/central/CN-01342759/full. | |
| 1017 | Rejected by TIAB Screening | Holland CA. Preparing for the future: A case study of role changing and reengineering. Clinical Laboratory Management Review. 1995;9(5):363-9. | |
| 1018 | Rejected by TIAB Screening | Holley D, Santos P, Cook J, Kerr M. "Cascades, Torrents & Drowning" in Information: Seeking Help in the Contemporary General Practitioner Practice in the UK. Interactive Learning Environments. 2016;24(5):954-67. | |
| 1019 | Rejected by TIAB Screening | Holmboe ES, Lynn L, Duffy FD. Improving the quality of care via maintenance of certification and the Web: an early status report. Perspectives in Biology & Medicine. 2008;51(1):71-83. | |
| 1020 | Rejected by TIAB Screening | Holmgren D, Aspegren K, Wekell P. [On call education for paediatricians may improve patient safety. Continuing professional development project in western Sweden evaluated]. Lakartidningen. 2016;113(03):08. | |
| 1021 | Rejected by TIAB Screening | Homer CS, O Alpert, Hr Owusu, Ms Schneider, L Rappaport, La Rubin, Dh. An evaluation of an innovative multimedia educational software program for asthma management: report of a randomized, controlled trial. Pediatrics [Internet]. 2000; 106(1 Pt 2):[210‐5 pp.]. Available from: https://www.cochranelibrary.com/central/doi/10.1002/central/CN-00298159/full. | |
| 1022 | Not Target Group | Hong H, Zhou L, Maitta RW, Downes KA. Development of resident self-tracking tools for measurement of milestones in transfusion medicine quality assurance education. Transfusion. 2013;53:247A-8A. | |
| 1023 | Rejected by TIAB Screening | Hook KM, Pfeiffer CA. Impact of a new curriculum on medical students' interpersonal and interviewing skills. Medical Education. 2007;41(2):154-9. | |
| 1024 | Rejected by TIAB Screening | Hooyman N. Challenges to Adopting and Sustaining Family-Focused Competencies in Social Work Education. Journal of Social Work Education. 2008;44:137-41. | |
| 1025 | Rejected by TIAB Screening | Horlick M, Rockfeld J, Fishman M, Cocks PM, Porter B. A novel ambulatory care curriculum: Teaching the skills of information managementand life-long learning. Journal of General Internal Medicine. 2014;29:S502. | |
| 1026 | Formal Teaching | Horsley T, O'Neill J, McGowan JL, Perrier L, Kane G, Campbell C. Interventions to improve question formulation in professional practice and self-directed learning: Systematic review. Medical Education, Supplement. 2010;44:35. | |
| 1027 | Rejected by TIAB Screening | Hortin JA. Successful Examples of Instructional Technology in Higher Education. Reports - Descriptive. 1981. | |
| 1028 | Rejected by TIAB Screening | Hoskins B. A Day in the Sandbox. Journal of Continuing Higher Education. 2009;57(1):48-50. | |
| 1029 | Rejected by TIAB Screening | Hosmer-Gallo C, Welch LK, Hefele B. The use of advanced learning formats and partnerships to enhance cme and diabetes care. Diabetes Conference: 79th Scientific Sessions of the American Diabetes Association, ADA. 2019;68(Supplement 1). | |
| 1030 | Rejected by TIAB Screening | Hosoi E. [Continuous learning process in nursing: a comment by a nursing supervisor]. Kangogaku Zasshi - Japanese Journal of Nursing. 1984;48(1):88. | |
| 1031 | Rejected by TIAB Screening | Hoverman JR, Mann BB, Hayes J, Wilfong LS, Neubauer MA. A schematic case episode format to aid in "Choosing Wisely". Journal of Clinical Oncology Conference: ASCO's Quality Care Symposium. 2016;34(7 SUPPL. 1). | |
| 1032 | Rejected by TIAB Screening | Howard J. The emotional diary - A framework for reflective practice. Education for General Practice. 1997;8(4):288-91. | |
| 1033 | Rejected by TIAB Screening | Hsiao CT, Mm Chen, Cc. Using interactive multimedia e-Books for learning blood cell morphology in pediatric hematology. BMC medical education [Internet]. 2016; 16(1):[290 p.]. Available from: https://www.cochranelibrary.com/central/doi/10.1002/central/CN-01914260/full. | |
| 1034 | Rejected by TIAB Screening | Hsu LL, Hsieh SI. Development and psychometric evaluation of the competency inventory for nursing students: a learning outcome perspective. Nurse Education Today. 2013;33(5):492-7. | |
| 1035 | Rejected by TIAB Screening | Hu J. Vocational quality training of clinical pharmacists. [Chinese]. Pharmaceutical Care and Research. 2013;13(3):161-5. | |
| 1036 | Rejected by TIAB Screening | Huang JT, L Tompane, T Dillon, L Pian, M Gottschalk, M Norman, Gj Bartholomew, Lk. Preparing adolescents with chronic disease for transition to adult care: a technology program. Pediatrics [Internet]. 2014; 133(6):[e1639‐46 pp.]. Available from: https://www.cochranelibrary.com/central/doi/10.1002/central/CN-01053774/full. | |
| 1037 | Rejected by TIAB Screening | Hudson R, Maslin-Prothero S, Oates L. Flexible Learning in Action: Case Studies in Higher Education. Staff and Educational Development Series1997. | |
| 1038 | Rejected by TIAB Screening | Hudziak JJ. Environments, Epigenes, Brains, and Behavior. Journal of the American Academy of Child and Adolescent Psychiatry. 2018;57 (10 Supplement):S126. | |
| 1039 | Formal Teaching | Huggins K. Lifelong learning - The key to competence in the intensive care unit? Intensive and Critical Care Nursing. 2004;20(1):38-44. | |
| 1040 | Outside SDL | Hull C. Modernizing lifelong learning. Professional Nurse. 2000;15(5):294-5. | |
| 1041 | Rejected by TIAB Screening | Hummel LJ. An Approach to a Study of Physician Learning Patterns. Mobius. 1984;4(4):48-50. | |
| 1042 | Outside SDL | Hummel LJ. An investigation of physician self-directed learning activities. Research in medical education:proceedings of the annual Conference. 1985;24:213-8. | |
| 1043 | Outside SDL | Hunt J. Greatest Hits for Making It Stick: Enhancing Durable Knowledge in Lifelong Learning. Journal of the American Academy of Child and Adolescent Psychiatry. 2019;58 (10 Supplement):S127-S8. | |
| 1044 | Outside SDL | Hunt J, Brannan E, Sexson S. Lifelong Learning for Professional Development in Psychiatry: Pedagogy, Innovations, and Maintenance of Certification. Psychiatric Clinics of North America. 2019;42(3):425-37. | |
| 1045 | Outside SDL | Hunt JI, Sexson SB, Williams ER, Birmaher B, Walkup JT, Wilens T. Helping You Know What You Don't Know: A Self-Assessment Review of Psychopharmacology. Journal of the American Academy of Child and Adolescent Psychiatry. 2018;57 (10 Supplement):S336. | |
| 1046 | Rejected by TIAB Screening | Hunter DJ, Lapp I, Frenk J. Education in public health: Expanding the frontiers. American Journal of Preventive Medicine. 2014;Part S3. 47(5):S286-S7. | |
| 1047 | Rejected by TIAB Screening | Hunter JP, Stinson J, Campbell F, Stevens B, Wagner SJ, Simmons B, et al. A novel pain interprofessional education strategy for trainees: Assessing impact on interprofessional competencies and pediatric pain knowledge. Pain Research and Management. 2015;20(1):e12-e20. | |
| 1048 | Rejected by TIAB Screening | Hussein IH. Integrated self-study medical histology modules: Innovative approach. FASEB Journal Conference: Experimental Biology. 2018;32(1 Supplement 1). | |
| 1049 | Rejected by TIAB Screening | Huxley C, Sturt J, Dale J, Walker R, Caramlau I, O'Hare JP, et al. Is it possible to predict improved diabetes outcomes following diabetes self-management education: a mixed-methods longitudinal design. BMJ Open. 2015;5(11):e008781. | |
| 1050 | Rejected by TIAB Screening | Huyghe EM, P Sui, D Schover, Lr. Banking on Fatherhood: pilot studies of a computerized educational tool on sperm banking before cancer treatment. Psycho-oncology [Internet]. 2009; 18(9):[1011‐4 pp.]. Available from: https://www.cochranelibrary.com/central/doi/10.1002/central/CN-00729849/full. | |
| 1051 | Rejected by TIAB Screening | Ifanti AA, Argyriou AA, Kalofonos HP. Promises and hurdles of undergraduate medical development in Greece. Advances in Medical Education & Practice. 2011;2:201-8. | |
| 1052 | Not Target Group | Ihm J, Choi H, Roh S. Flipped-learning course design and evaluation through student self-assessment in a predental science class. Korean Journal of Medical Education. 2017;29(2):93-100. | |
| 1053 | Rejected by TIAB Screening | Ihm JJ, Lee G, Kim KK, Jang KT, Jin BH. Who succeeds at dental school? Factors predicting students' academic performance in a dental school in republic of Korea. Journal of Dental Education. 2013;77(12):1616-23. | |
| 1054 | Rejected by TIAB Screening | Imafuku R, Kataoka R, Ogura H, Suzuki H, Enokida M, Osakabe K. What did first-year students experience during their interprofessional education? A qualitative analysis of e-portfolios. Journal of Interprofessional Care. 2018;32(3):358-66. | |
| 1055 | Rejected by TIAB Screening | Imbert E, Mills L, Chou CL, Chang A, Saba G, Azzam A, et al. Inter professional curriculum for early health professions student son communication to promote patient adherence. Journal of General Internal Medicine. 2018;33 (2 Supplement 1):721. | |
| 1056 | Rejected by TIAB Screening | Immonen K, Oikarainen A, Tomietto M, Kaariainen M, Tuomikoski AM, Kaucic BM, et al. Assessment of nursing students' competence in clinical practice: A systematic review of reviews. International Journal of Nursing Studies. 2019;100:103414. | |
| 1057 | Rejected by TIAB Screening | Infantino M. Gardening: a strategy for health promotion in older women. Journal of the New York State Nurses Association. 2004;35(2):10-7. | |
| 1058 | Rejected by TIAB Screening | Inui TS, Williams Jr WT, Goode L, Anderson RJ, Bhak KN, Forsyth JD, et al. Sustaining the development of primary care in academic medicine. Academic Medicine. 1998;73(3):245-57. | |
| 1059 | Rejected by TIAB Screening | Inuwa I, Taranikanti V, RoyChoudhry S. An observation of the teaching role of clinicians and basic scientists during early year of a revised undergraduate medical curriculum - The Sultan Qaboos University experience. Journal of Anatomy. 2012;221 (1):81-2. | |
| 1060 | Rejected by TIAB Screening | Irabor OC, Berger A, Mbarika V, Hammad N, Odedina F, Ngwa W. Assessing trainee's need and readiness for ecancer education and training in Africa. Journal of Clinical Oncology Conference. 2018;36(15 Supplement 1). | |
| 1061 | Rejected by TIAB Screening | Irani JL, Greenberg JA, Blanco MA, Greenberg CC, Ashley S, Lipsitz SR, et al. Educational value of the operating room experience during a core surgical clerkship. American Journal of Surgery. 2010;200(1):167-72. | |
| 1062 | Rejected by TIAB Screening | Irby DM. Teaching and learning in ambulatory care settings: a thematic review of the literature. Academic Medicine. 1995;70(10):898-931. | |
| 1063 | Rejected by TIAB Screening | Irvine AB, Mb Gates, Dm Fitzwater, El Seeley, Jr Bourgeois, M. Internet training to respond to aggressive resident behaviors. Gerontologist [Internet]. 2012; 52(1):[13‐23 pp.]. Available from: https://www.cochranelibrary.com/central/doi/10.1002/central/CN-00840660/full. | |
| 1064 | Rejected by TIAB Screening | Irvine BB, Mb Gates, Dm Fitzwater, El Seeley, Jr Bourgeois, M. An internet training to reduce assaults in long-term care. Geriatric nursing (new york, NY) [Internet]. 2012; 33(1):[28‐40 pp.]. Available from: https://www.cochranelibrary.com/central/doi/10.1002/central/CN-00814762/full. | |
| 1065 | Not Target Group | Irvine S, Williams B, Ozmen M, McKenna L. Exploration of self-regulatory behaviours of undergraduate nursing students learning to teach: A social cognitive perspective. Nurse Education in Practice. 2019;41:102633. | |
| 1066 | Rejected by TIAB Screening | Irwin M, Malague M. Nursing Transition: An Individualized Course To Promote Mobility from the LVN to RN Role. Registered Nurse Shortage Project. Final Report. Reports - Descriptive. North Harris Montgomery Community Coll. District, Houston, TX.; 1992. | |
| 1067 | Rejected by TIAB Screening | Isenberg S. Merging Education and Business Models to Create and Sustain Transformational Change. International Journal of Adult Vocational Education and Technology. 2010;1(4):31-47. | |
| 1068 | Rejected by TIAB Screening | Ismeurt J, Ismeurt R, Miller BK. Field-dependence/independence: considerations in staff development. Journal of Continuing Education in Nursing. 1992;23(1):38-41. | |
| 1069 | Not Target Group | Iwata J, Clayton J, Saravani S-J. Using Self-Reflection and Badges in Moodle-Based Medical English Review Courses for Enhancing Learners' Autonomy. International Association for Development of the Information Society Paper presented at the International Conference on Educational Technologies. 2013. | |
| 1070 | Rejected by TIAB Screening | Izzard RS, Pounder DJ. Human rights and medical education. Romanian Journal of Legal Medicine. 1997;5(1):87-91. | |
| 1071 | Not Target Group | Jabade MV, Joshi SG, Chavan R. A study to assess the effectiveness of self-instructional module on knowledge and practices of needle stick injury among the nurses working in selected hospitals of Pune City. Indian Journal of Public Health Research and Development. 2019;10(7):1562-5. | |
| 1072 | Not Target Group | Jabbour J, Bakeman A, Robey T, Jabbour N. Self-directed Learning in Otolaryngology Residents' Preparation for Surgical Cases. Annals of Otology, Rhinology & Laryngology. 2017;126(4):296-303. | |
| 1073 | Rejected by TIAB Screening | Jackson D, McDonald G, Wilkes L. Thriving in the workplace: Learning from innovative practices. The resilient nurse: Empowering your practice. New York, NY: Springer Publishing Company; US; 2011. p. 105-14. | |
| 1074 | Rejected by TIAB Screening | Jackson D, Watson R. Workplace learning: a continuing concern in nurse education. Contemporary Nurse. 2011;38(1-2):3-5. | |
| 1075 | Outside SDL | Jackson L, Jowsey T, Honey MLL. In-Service Education: Evolving Internationally to Meet Nurses' Lifelong Learning Needs. Journal of Continuing Education in Nursing. 2019;50(7):313-8. | |
| 1076 | Rejected by TIAB Screening | Jackson N, Fellows C, Leng J. Adding value to the education of nurses, midwives and operating department practitioners through a 'life-wide' curriculum. Nurse Education Today. 2010;30(3):271-5. | |
| 1077 | Outside SDL | Jackson S. Learning to Live: The Relationship between Lifelong Learning and Lifelong Illness. International Journal of Lifelong Education. 2006;25(1):51-73. | |
| 1078 | Rejected by TIAB Screening | Jackson T. Building the 'continuous learning' healthcare system. The HIM journal. 2014;43(1):4-5. | |
| 1079 | Rejected by TIAB Screening | Jaffar AA. YouTube: An emerging tool in anatomy education. Anatomical sciences education. 2012;5(3):158-64. | |
| 1080 | Rejected by TIAB Screening | Jain YS, Garg A, Jhamb DK, Jain P, Karar A. Preparing India to leverage power of mobile technology: Development of a bilingual mobile health tool for heart patients. Cardiovascular and Hematological Agents in Medicinal Chemistry. 2019;17(2):125-34. | |
| 1081 | Rejected by TIAB Screening | Jakobsen F, Musaeus P, Kirkeby L, Hansen TB, Morcke AM. Emotions and clinical learning in an interprofessional outpatient clinic: a focused ethnographic study. Journal of interprofessional care. 2019;33(1):57-65. | |
| 1082 | Rejected by TIAB Screening | Jamatia B. Bridging the Gap between Community and Cardiologists. Journal of Learning for Development. 2015;2(1). | |
| 1083 | Rejected by TIAB Screening | James C, James N, Davies D, Harvey P, Tweddle S. Preferences for different sources of information about cancer. Patient Education & Counseling. 1999;37(3):273-82. | |
| 1084 | Rejected by TIAB Screening | Jansson AB, Engstrom A, Parding K. What about learning? A study of temporary agency staffing and learning conditions in Swedish health care. Journal of Workplace Learning. 2020;32(1):63-75. | |
| 1085 | Rejected by TIAB Screening | Jantzen D. Reframing professional development for first-line nurses: Feature. Nursing Inquiry. 2008;15(1):21-9. | |
| 1086 | Rejected by TIAB Screening | Jantzen D. Refining nursing practice through workplace learning: A grounded theory. Journal of Clinical Nursing. 2019;28(13-14):2565-76. | |
| 1087 | Rejected by TIAB Screening | Jarvis P. Paradoxes of learning: On becoming an individual in society. San Francisco, CA: Jossey-Bass; US; 1992. | |
| 1088 | Outside SDL | Jarvis P. Lifelong education and its relevance to nursing. Nurse Education Today. 2005;25(8):655-60. | |
| 1089 | Rejected by TIAB Screening | Jarvis P, Watts M. The Routledge International Handbook of Learning. Routledge International Handbooks of Education. Routledge, Taylor and Francis Group. 2011. | |
| 1090 | Rejected by TIAB Screening | Javaudin F, Montassier E, Goffinet N, Quilliot F, Potel G, Batard E. Interactive E-learning in the Emergency Department: Participation in an Antibiotic stewardship Program. Annales Francaises de Medecine d'Urgence. 2014;4(3):167-72. | |
| 1091 | Rejected by TIAB Screening | Jeffries PR, Beach M, Decker SI, Dlugasch L, Groom J, Settles J, et al. Multi-center development and testing of a simulation-based cardiovascular assessment curriculum for advanced practice nurses. Nursing Education Perspectives. 2011;32(5):316-22. | |
| 1092 | Not Target Group | Jelovsek JE, Diwadkar G, Frick AC, Taylor C. Self-directed learning and supervised practice sessions with verbal feedback improves basic and advanced surgical performance in first year gynecology residents. Journal of Pelvic Medicine and Surgery. 2010;16(2):S14-S5. | |
| 1093 | Rejected by TIAB Screening | Jenkins K. Joint ranzcp/jspn symposium: Innovations in psychiatric continuing professional development. Australian and New Zealand Journal of Psychiatry. 2019;53 (Supplement 1):26. | |
| 1094 | Rejected by TIAB Screening | Jenkins SG, R Morrell, Ds. Computer-assisted instruction versus traditional lecture for medical student teaching of dermatology morphology: a randomized control trial. Journal of the american academy of dermatology [Internet]. 2008; 59(2):[255‐9 pp.]. Available from: https://www.cochranelibrary.com/central/doi/10.1002/central/CN-00648641/full. | |
| 1095 | Not Target Group | Jenkins TB, Carlson JH, Herrick CA. Developing self-directed learning modules. Journal of Nursing Staff Development. 1998;14(1):17-22. | |
| 1096 | Outside SDL | Jennett PA, Swanson RW. Lifelong, Self-Directed Learning: Why Physicians and Educators Should Be Interested. [and] Traditional and New Approaches to CME: Perceptions of a Variety of CME Activities. Journal of Continuing Education in the Health Professions. 1994;14(2):69-82. | |
| 1097 | Rejected by TIAB Screening | Jensen K. The Desire to Learn: An Analysis of Knowledge-Seeking Practices among Professionals. Oxford Review of Education. 2007;33(4):489-502. | |
| 1098 | Rejected by TIAB Screening | Jenson C, Reid F, Rowlands G. Locum and salaried general practitioners: An exploratory study of recruitment, morale, professional development and clinical governance. Education for Primary Care. 2008;19(3):285-302. | |
| 1099 | Rejected by TIAB Screening | Jesus LE. [Surgeons training: today as always?]. Revista do Colegio Brasileiro de Cirurgioes. 2009;36(6):529-32. | |
| 1100 | Not Target Group | Jha V, Duffy S, Murdoch-Eaton D. Development of transferable skills during short special study modules: students' self-appraisal. Medical Teacher. 2002;24(2):202-4. | |
| 1101 | Rejected by TIAB Screening | Jiang T, Jiang J, Wang RP, Zhou Y, Wu Y, Xu L. Study in clinical teaching for ophthalmology professional degree graduate students. [Chinese]. International Eye Science. 2016;16(11):2109-11. | |
| 1102 | Rejected by TIAB Screening | Jibaja-Weiss MV, Rj Granchi, Ts Neff, Ne Robinson, Ek Spann, Sj Aoki, N Friedman, Lc Beck, Jr. Entertainment education for breast cancer surgery decisions: a randomized trial among patients with low health literacy. Patient education and counseling [Internet]. 2011; 84(1):[41‐8 pp.]. Available from: https://www.cochranelibrary.com/central/doi/10.1002/central/CN-00813311/full. | |
| 1103 | Rejected by TIAB Screening | Johnson B, Webb M, Bhalakia A, Beckworth K, Wesson DE, Naik-Mathuria B. Physician-Driven Firearm Storage Education: Does it Work? Journal of the American College of Surgeons. 2019;229 (4 Supplement 1):S212. | |
| 1104 | Rejected by TIAB Screening | Johnson CE, Keating JL, Molloy EK. Psychological safety in feedback: What does it look like and how can educators work with learners to foster it? Medical education. 2020;54(6):559-70. | |
| 1105 | Not Target Group | Johnson CE, Yates K, Sullivan ME. Building a Framework for Self-Regulated Learning in Surgical Education: A Delphi Consensus Among Experts in Surgical Education. Journal of Surgical Education. 2019;76(6):e56-e65. | |
| 1106 | Rejected by TIAB Screening | Johnson LG. Assessing the Needs of Adult Learners: Methods and Models. Marketing Studies Series, Vol. I. Guides - Non-Classroom Reports - Evaluative. Ohio Board of Regents, Columbus.; 1980. | |
| 1107 | Rejected by TIAB Screening | Johnson P. Staff Retention. Personnel Management Module. Operational Management Programme. Second Edition. Guides - Classroom - Learner. Hotel and Catering Training Co., London (England). 1991. | |
| 1108 | Rejected by TIAB Screening | Johnson P. Leadership and Motivation. Supervisory Management Module. Operational Management Programme. Third Edition. Guides - Classroom - Learner. Hotel and Catering Training Co., London (England). 1991. | |
| 1109 | Rejected by TIAB Screening | Johnson PN, Gildon BL, Condren M, Miller JL, Hagemann TM, Lewis TV, et al. A survey of pediatric degree option program graduates in a doctor of pharmacy curriculum: Confidence and initial employment position. Currents in Pharmacy Teaching and Learning. 2019;11(12):1296-302. | |
| 1110 | Rejected by TIAB Screening | Johnson PT. Concept and Theory in Continuing Education for Nurses. Mobius. 1987;7(3):32-8. | |
| 1111 | Rejected by TIAB Screening | Jonas D, Burns B. The transition to blended e-learning. Changing the focus of educational delivery in children's pain management. Nurse Education in Practice. 2010;10(1):1-7. | |
| 1112 | Rejected by TIAB Screening | Jones D, Taylor T, Powell S, Scott J, Scott L. Parkinson's Disease (PD) through pictures and poetry: Neurological narratives in pre-registration physiotherapy education. Physiotherapy (United Kingdom). 2011;97:eS1523. | |
| 1113 | Not Target Group | Jones DB, Stefanidis D, Korndorffer JR, Jr., Dimick JB, Jacob BP, Schultz L, et al. SAGES University MASTERS Program: a structured curriculum for deliberate, lifelong learning. Surgical Endoscopy. 2017;31(8):3061-71. | |
| 1114 | Not Target Group | Jones JL, Kirkland M. From continuing education to continuing professional education: The shift to lifelong learning in occupational therapy. American Journal of Occupational Therapy. 1984;38(8):503-4. | |
| 1115 | Rejected by TIAB Screening | Jones ML, Filip SJ, Smith H, Remsburg-Bell E. Strategy management system in perinatal services: the role of a patient resource manager. Lippincott's Case Management. 2002;7(1):27-42. | |
| 1116 | Rejected by TIAB Screening | Jones VS, Holland AJA, Oldmeadow W. Inductive teaching method - An alternate method for small group learning. Medical Teacher. 2008;30(8):e246-e9. | |
| 1117 | Not Target Group | Jones WJ. Self-directed learning and student selected goals in nurse education. Journal of Advanced Nursing. 1981;6(1):59-69. | |
| 1118 | Not Target Group | Jones WP, Crank JN, Loe SA. Extending Specialist Training in Counseling: The Efficacy of Self-Instruction. College Student Journal. 2006;40(4):885-900. | |
| 1119 | Not Target Group | Jones-Schenk J. Embrace lifelong learning. Imprint. 2000;47(2):26. | |
| 1120 | Not Target Group | Jons-Cox LD. The self-directed osteopathic medical student: Bringing adult learning into the osteopathic manipulative technique lab. International Journal of Osteopathic Medicine. 2014;17(1):61-5. | |
| 1121 | Rejected by TIAB Screening | Jooma R. Proposals for a scheme for continuing professional development in Pakistan. Journal of the Pakistan Medical Association. 2011;61(12):1231-3. | |
| 1122 | Rejected by TIAB Screening | Josephsen J. Electronic Portfolios for Distance Learning: A Case from a Nursing Clinical Course. International Journal of ePortfolio. 2012;2(1):15-27. | |
| 1123 | Rejected by TIAB Screening | Josephsen JM. A Qualitative Analysis of Metacognition in Simulation. The Journal of nursing education. 2017;56(11):675-8. | |
| 1124 | Rejected by TIAB Screening | Jost GF, Cunningham M, Schaeren S. Aospine needs assessment at the spine surgery department, university hospital basel. Global Spine Journal. 2017;7 (2 Supplement 1):36S-7S. | |
| 1125 | Rejected by TIAB Screening | Joy AK, Sahm L, Mathers J, O'Flynn S, Kerins D. Interprofessional ward-based teamwork to enable safe prescribing practice in healthcare undergraduates. Medical Education, Supplement. 2011;45:35-6. | |
| 1126 | Rejected by TIAB Screening | Juba KM, Ricca BP. Design of a problem-based learning pain and palliative care elective course. Currents in Pharmacy Teaching and Learning. 2014;6(3):421-8. | |
| 1127 | Rejected by TIAB Screening | Jubin P. [Contract learning: effects of professionalization on the student nurse]. Recherche en Soins Infirmiers. 2013(112):107-24. | |
| 1128 | Rejected by TIAB Screening | Jung J, Shilkofski N. Pediatric Resuscitation Education in Low-Middle-Income Countries: Effective Strategies for Successful Program Development. Journal of Pediatric Intensive Care. 2017;6(1):12-8. | |
| 1129 | Rejected by TIAB Screening | Junod Perron N, Hudelson P. How do junior doctors working in a multicultural context make sense of somatisation? Swiss Medical Weekly. 2005;135(31-32):475-9. | |
| 1130 | Rejected by TIAB Screening | Jurjus RA, Krum J, Goldman EF. Design for learning: adapting the microscopic anatomy laboratory to adult learners. Anatomical sciences education. 2013;6(3):177-81. | |
| 1131 | Rejected by TIAB Screening | Kadmon M, Busemann A, Euteneier A, Gawad K, Grone J, Berberat P. Modular postgraduate training in surgery - A national concept with future. [German]. Zentralblatt fur Chirurgie - Zeitschrift fur Allgemeine, Viszeral- und Gefasschirurgie. 2012;137(2):138-43. | |
| 1132 | Not Target Group | Kammer JE, Hautz WE, Marz M. Self-monitoring accuracy does not increase throughout undergraduate medical education. Medical Education. 2020;54(4):320-7. | |
| 1133 | Not Target Group | Kao YH, Yu CW, Kuo SY, Kuang IH. [Self-directed learning in nursing students with different background factors]. [Chinese]. Hu li za zhi The journal of nursing. 2013;60(4):53-64. | |
| 1134 | Rejected by TIAB Screening | Kapoor A, Kalraiya A, Longia S. Use of SNAPPS Model for Pediatric Outpatient Education. Indian pediatrics. 2017;54(4):288-90. | |
| 1135 | Rejected by TIAB Screening | Karaduman AA, Aksu Yildirim S, Inal Ince D, Mutlu A, Bayrakci Tunay V, Ulger O, et al. Hacettepe University experience in an international multicenter project: Development of an interdisciplinary clinical master program in rehabilitation sciences. Turkish Journal of Physiotherapy and Rehabilitation. 2019;30 (2):S57. | |
| 1136 | Rejected by TIAB Screening | Karamichalis JM, Barach PR, Nathan M, Henaine R, del Nido PJ, Bacha EA. Assessment of technical competency in pediatric cardiac surgery. Progress in Pediatric Cardiology. | |
| 1137 | Rejected by TIAB Screening | Karani R, Fromme HB, Cayea D, Muller D, Schwartz A, Harris IB. How medical students learn from residents in the workplace: a qualitative study. Academic Medicine. 2014;89(3):490-6. | |
| 1138 | Not Target Group | Karimi FZ, Alesheikh A, Pakravan S, Abdollahi M, Damough M, Anbaran ZK, et al. Surveying the factor structure and reliability of the Persian version of the Jefferson Scale of Physician Lifelong Learning (JeffSPLL) in staff of medical sciences. Electronic Physician [Electronic Resource]. 2017;9(10):5611-6. | |
| 1139 | Rejected by TIAB Screening | Karkada S, Radhakrishnan J, Natarajan J, Matua GA, Kaddoura M. Knowledge and competency of novice nursing students in nasogastric tube feeding: Is simulation better than case scenario? Oman Medical Journal. 2019;34(6):528-33. | |
| 1140 | Rejected by TIAB Screening | Kashora FK, Charles DA. Online-learning: exploring practices among Foundation doctors. Journal of Advances in Medical Education & Professionalism. 2019;7(1):14-9. | |
| 1141 | Not Target Group | Kassab SE, Al-Shafei AI, Salem AH, Otoom S. Relationships between the quality of blended learning experience, self-regulated learning, and academic achievement of medical students: a path analysis. Advances in Medical Education & Practice. 2015;6:27-34. | |
| 1142 | Rejected by TIAB Screening | Kassam A, Sharma N, Harvie M, O'Beirne M, Topps M. Patient safety principles in family medicine residency accreditation standards and curriculum objectives: Implications for primary care. Canadian Family Physician. 2016;62(12):e731-e9. | |
| 1143 | Rejected by TIAB Screening | Kasschau RA. Experiential Learning In Introductory Psychology: Student's Perspective. Reports - Descriptive Speeches/Meeting Papers. 1978. | |
| 1144 | Not Target Group | Kastenmeier AS, Redlich PN, Fihn C, Treat R, Chou R, Homel A, et al. Individual learning plans foster self-directed learning skills and contribute to improved educational outcomes in the surgery clerkship. American Journal of Surgery. 2018;216(1):160-6. | |
| 1145 | Rejected by TIAB Screening | Kastner M, Lillie E, Ashoor H, Perrier L, Cardoso R, Straus S, et al. Quality improvement strategies to optimise transition of patients with heart failure to independent living: Protocol for a scoping review. BMJ Open. 2015;4(11). | |
| 1146 | Not Target Group | Kato H, Burger A, Emoto K, Sakama R, Uehara Y, Segon A, et al. Differences in goals during residency training between the united states and Japan: Time to address gaps between competencies and trainee self-identified goals. Journal of General Internal Medicine. 2017;32 (2 Supplement 1):S163. | |
| 1147 | Rejected by TIAB Screening | Katz LG, et al. Current Topics in Early Childhood Education. Volume IV. Reports - Research Collected Works - General ERIC Publications. ERIC Clearinghouse on Elementary and Early Childhood Education, Urbana, IL.; 1982. | |
| 1148 | Rejected by TIAB Screening | Katzman JG, Comerci G, Boyle JF, Duhigg D, Shelley B, Olivas C, et al. Innovative telementoring for pain management: project ECHO pain. The Journal of continuing education in the health professions. 2014;34(1):68-75. | |
| 1149 | Rejected by TIAB Screening | Kauffmann E, Harrison MB, Burke SO, Wong C. Stress-point intervention for parents of children hospitalized with chronic conditions. Pediatric nursing. 1998;24(4):362-6. | |
| 1150 | Rejected by TIAB Screening | Kaufman JA. Arguments for and against recertification. CardioVascular and Interventional Radiology. 2013;36:S65. | |
| 1151 | Rejected by TIAB Screening | Kay J. Review of Professionalism in psychiatry. The American Journal of Psychiatry. 2012;169(5):543-4. | |
| 1152 | Rejected by TIAB Screening | Kaye AD, Okanlawon OJ, Urman RD. Clinical performance feedback and quality improvement opportunities for perioperative physicians. Advances in Medical Education & Practice. 2014;5:115-23. | |
| 1153 | Rejected by TIAB Screening | Keegan RH, T Brown, G. Use of the virtual ventilator, a screen-based computer simulation, to teach the principles of mechanical ventilation. Journal of veterinary medical education [Internet]. 2009; 36(4):[436‐43 pp.]. Available from: https://www.cochranelibrary.com/central/doi/10.1002/central/CN-00751975/full. | |
| 1154 | Rejected by TIAB Screening | Keeping LM, English LM. Informal and incidental learning with patients who use continuous ambulatory peritoneal dialysis. Nephrology Nursing Journal: Journal of the American Nephrology Nurses' Association. 2001;28(3):313-4, 9-22; discussion 23. | |
| 1155 | Rejected by TIAB Screening | Keeve PL, Gerhards U, Arnold WA, Zimmer S, Zollner A. Job requirements compared to dental school education: impact of a case-based learning curriculum. GMS Zeitschrift Fur Medizinische Ausbildung. 2012;29(4):Doc54. | |
| 1156 | Rejected by TIAB Screening | Keim-janssen SA, Vandermeulen SP. Competence and confidence increases with clinical skills testing in the gross anatomy lab. Clinical Anatomy. 2011;24 (8):1028. | |
| 1157 | Rejected by TIAB Screening | Kekale T, Cervai S. Editorial. Journal of Workplace Learning. 2008;20(3):No Pagination Specified. | |
| 1158 | Rejected by TIAB Screening | Keller JM, Claar D, Chu DC, Ferreira JC, Hossain T, Carlos WG, et al. Mechanical ventilation training during graduate medical education. American Journal of Respiratory and Critical Care Medicine Conference. 2019;199(9). | |
| 1159 | Rejected by TIAB Screening | Kelley FJ, Kopac CA. Advanced health assessment in nurse practitioner programs. Journal of Professional Nursing. 2001;17(5):218-25. | |
| 1160 | Rejected by TIAB Screening | Kelly AM, Richardson D. Training for the role of triage in Australasia. Emergency Medicine. 2001;13(2):230-2. | |
| 1161 | Rejected by TIAB Screening | Kelly B, Squires JE, Feingold B, Hooper DK, Mazariegos GV. Quality initiatives in pediatric transplantation. Current Opinion in Organ Transplantation. 2019;24(1):64-72. | |
| 1162 | Rejected by TIAB Screening | Kelly DR, MacKay L. CELT: a computerised evaluative learning tool for continuing professional development. Medical Education. 2003;37(4):358-67. | |
| 1163 | Not Target Group | Kennedy G, Rea JNM, Rea IM. Prompting medical students to self-assess their learning needs during the ageing and health module: a mixed methods study. Medical Education Online. 2019;24(1):1579558. | |
| 1164 | Rejected by TIAB Screening | Kennedy MB, Williams SE, Haq I, Okorie M. UK medical students' perspectives on practical prescribing teaching and learning provisions: a cross-sectional survey. European Journal of Clinical Pharmacology. 2019;75(10):1451-8. | |
| 1165 | Not Target Group | Kennedy MR, Coelho C. Self-regulation after traumatic brain injury: a framework for intervention of memory and problem solving. Seminars in Speech & Language. 2005;26(4):242-55. | |
| 1166 | Rejected by TIAB Screening | Kenny AJ, Kendall S. Serving two masters: quality teaching and learning versus economic rationalism. Nurse Education Today. 2001;21(8):648-55. | |
| 1167 | Rejected by TIAB Screening | Kent F, Hayes J, Glass S, Rees CE. Pre-registration interprofessional clinical education in the workplace: a realist review. Medical Education. 2017;51(9):903-17. | |
| 1168 | Rejected by TIAB Screening | Kent F, Nankervis K, Johnson C, Hodgkinson M, Baulch J, Haines T. 'More effort and more time.' Considerations in the establishment of interprofessional education programs in the workplace. Journal of interprofessional care. 2018;32(1):89-94. | |
| 1169 | Rejected by TIAB Screening | Kerka S. Health and Adult Literacy. Practice Application Brief No. 7. ERIC Publications. ERIC Clearinghouse on Adult, Career, and Vocational Education, Columbus, OH.; 2000. | |
| 1170 | Not Target Group | Kerr D, Ratcliff J, Tabb L, Walter R. Undergraduate nursing student perceptions of directed self-guidance in a learning laboratory: An educational strategy to enhance confidence and workplace readiness. Nurse Education in Practice. 2020;42:102669. | |
| 1171 | Rejected by TIAB Screening | Kerr E. I'm finding it hard to talk-Airway compromise following tPA. International Journal of Stroke. 2013;8:19. | |
| 1172 | Not Target Group | Khabaz Mafinejad M, Aghili R, Emami Z, Malek M, Baradaran H, Taghavinia M, et al. Study guides: effective tools to improve self-directed learning skills of medical students. Acta Medica Iranica. 2014;52(10):781-5. | |
| 1173 | Rejected by TIAB Screening | Khalfallah M, Dougaz W, Jerraya H, Samaali I, Mazigh S, Loueslati MH, et al. Self-directed learning digital tool versus tutorials under the guidance of an educator: Randomized trial. [French]. Tunisie Medicale. 2019;97(2):296-303. | |
| 1174 | Rejected by TIAB Screening | Khalfallah M, Dougaz W, Jerraya H, Samaali I, Mazigh S, Loueslati MH, et al. Self-directed learning digital tool versus tutorials under the guidance of an educator: Randomized trial. Tunisie Medicale. 2019;97(2):296-303. | |
| 1175 | Rejected by TIAB Screening | Khalil MK, Paas F, Johnson TE, Su YK, Payer AF. Effects of instructional strategies using cross sections on the recognition of anatomical structures in correlated CT and MR images. Anatomical Sciences Education. 2008;1(2):75-83. | |
| 1176 | Rejected by TIAB Screening | Khan MM, Kosciuk P, Cole A. Educational quality improvement for the adult learner: Implementation of a structured, self-directed, fellow driven outpatient pulmonary curriculum. American Journal of Respiratory and Critical Care Medicine Conference. 2019;199(9). | |
| 1177 | Rejected by TIAB Screening | Khan R, Plahouras J, Johnston BC, Scaffidi MA, Grover SC, Walsh CM. Virtual reality simulation training for health professions trainees in gastrointestinal endoscopy. Cochrane Database of Systematic Reviews. 2018;2018(8). | |
| 1178 | Rejected by TIAB Screening | Khazanova D, Safdieh JE. Continuing Medical Education in Neurology. Seminars in Neurology. 2018;38(4):479-85. | |
| 1179 | Rejected by TIAB Screening | Kho MC, Ks Azhar, Mn Hamzah, Ml Chuah, Km Bustam, A Chan, Hc. Implementing blended learning in emergency airway management training: a randomized controlled trial. BMC emergency medicine [Internet]. 2018; 18(1):[1 p.]. Available from: https://www.cochranelibrary.com/central/doi/10.1002/central/CN-01449486/full. | |
| 1180 | Rejected by TIAB Screening | Kido M, Yoshiyama Y, Yago K, Itoh T, Currie J, Sorofman B. Japanese pharmacy education change facilitated by faculty development in the United States. Journal of the American Pharmacists Association. 2010;50 (2):282. | |
| 1181 | Rejected by TIAB Screening | Kiely BM, Ig. Stress self-help packages in primary care: a controlled trial evaluation. Journal of the Royal College of General Practitioners [Internet]. 1986; 36(288):[307‐9 pp.]. Available from: https://www.cochranelibrary.com/central/doi/10.1002/central/CN-00043924/full. | |
| 1182 | Rejected by TIAB Screening | Kiener ME. The incorporation of learning among British and American nurses: Two case studies. Dissertation Abstracts International Section A: Humanities and Social Sciences. 1984;45(5-A):1277. | |
| 1183 | Rejected by TIAB Screening | Kim H, Yoo Y, Shim H. Effects of an Internet-based intervention on plasma glucose levels in patients with type 2 diabetes. Journal of nursing care quality [Internet]. 2005; 20(4):[335‐40 pp.]. Available from: https://www.cochranelibrary.com/central/doi/10.1002/central/CN-00530456/full. | |
| 1184 | Rejected by TIAB Screening | Kim J, Lee S, Kim J. Effects of a web-based stroke education program on recurrence prevention behaviors among stroke patients: a pilot study. Health education research [Internet]. 2013; 28(3):[488‐501 pp.]. Available from: https://www.cochranelibrary.com/central/doi/10.1002/central/CN-00886115/full. | |
| 1185 | Rejected by TIAB Screening | Kim JS, Koh MS, Sun KS. Development of e-learning multimedia contents for PBL. Studies in health technology and informatics. 2006;122:813-5. | |
| 1186 | Rejected by TIAB Screening | Kim Y. The learning characteristics of primary care physicians. [Korean]. Korean journal of medical education. 2015;27(3):213-9. | |
| 1187 | Rejected by TIAB Screening | King A, Adams D, Barrie M. Utilization of educational blogs to supplement self-directed learning and small group based didactic sessions. Western Journal of Emergency Medicine. 2016;17 (Supplement 1):S78. | |
| 1188 | Rejected by TIAB Screening | King A, Holder Jr MG, Ahmed RA. Errors as allies: Error management training in health professions education. BMJ Quality and Safety. 2013;22(6):516-9. | |
| 1189 | Rejected by TIAB Screening | King JA, Fitchett EJ, Winyard P, Fertleman C. An intercalated BSC in paediatrics and child health: Improving the profile of paediatric training in medical school undergraduates. Archives of Disease in Childhood. 2019;104 (Supplement 2):S239-A40. | |
| 1190 | Rejected by TIAB Screening | King T, Smith L. Patient's knowledge of heart failure medications: Are they understanding? Heart and Lung. 2015;44 (6):557. | |
| 1191 | Rejected by TIAB Screening | King TS, Sharma R, Jackson J, Fiebelkorn KR. Clinical Case-Based Image Portfolios in Medical Histopathology. Anatomical sciences education. 2019;12(2):200-9. | |
| 1192 | Rejected by TIAB Screening | Kinsella D, Fry M, Zecchin A. Motivational factors influencing nurses to undertake postgraduate hospital-based education. Nurse Education in Practice. 2018;31:54-60. | |
| 1193 | Rejected by TIAB Screening | Kirby JR, Delva M, Knapper C, Birtwhistle R. Development of the Approaches to Work and Workplace Climate Questionnaires for Physicians. Evaluation & the Health Professions. 2003;26(1):104-21. | |
| 1194 | Rejected by TIAB Screening | Kirch DG. The role of academic psychiatry in the transformation of health care. Academic Psychiatry. 2011;35(2):73-5. | |
| 1195 | Rejected by TIAB Screening | Kirkpatrick MK. NINE: newspapers in nursing education. Nurse educator. 1994;19(6):21-3. | |
| 1196 | Rejected by TIAB Screening | Kirkpatrick MK, Brown ST. Leadership development in geriatric care through the Intergeneration Make a Difference Project. Nursing Education Perspectives. 2006;27(2):89-92. | |
| 1197 | Rejected by TIAB Screening | Kirkpatrick MK, Esterhuizen P, Jesse E, Brown ST. Improving self-directed learning/intercultural competencies: breaking the silence. Nurse educator. 2015;40(1):46-50. | |
| 1198 | Rejected by TIAB Screening | Kirrane C. Using action learning in reflective practice. Professional nurse (London, England). 2001;16(5):1102-5. | |
| 1199 | Rejected by TIAB Screening | Kirsivali-Farmer K. Staff development sessions. A strategy to facilitate nursing staff education with limited teaching resources. Journal of nursing staff development : JNSD. 1994;10(4):214-8. | |
| 1200 | Rejected by TIAB Screening | Kissin EY, Nishio J, Yang M, Backhaus M, Balint PV, Bruyn GA, et al. Self-directed learning of basic musculoskeletal ultrasound among rheumatologists in the United States. Arthritis care & research. 2010;62(2):155-60. | |
| 1201 | Rejected by TIAB Screening | Klaus R, Lee L. Efficacy of undergraduate medical education in anatomical sciences for surgical residency preparation. FASEB Journal Conference: Experimental Biology. 2017;31(1 Supplement 1). | |
| 1202 | Rejected by TIAB Screening | Kleiber P, Tisdell L. Annual Adult Education Research Conference (AERC) Proceedings (31st, Athens, Georgia, May 18-20, 1990). Collected Works - Proceedings. Georgia Univ., Athens. Center for Continuing Education.; 1990. | |
| 1203 | Rejected by TIAB Screening | Klein AV, Hardy S, Lim R, Marshall DA. Regulatory Decision Making in Canada-Exploring New Frontiers in Patient Involvement. Value in Health. 2016;19(6):730-3. | |
| 1204 | Outside SDL | Klein C. Continuum Lifelong Learning in Neurology: Introduction. CONTINUUM Lifelong Learning in Neurology. 2008;14(2):13-4. | |
| 1205 | Rejected by TIAB Screening | Klessig JM, Wolfsthal SD, Levine MA, Stickley W, Bing-You RG, Lansdale TF, et al. A pilot survey study to define quality in residency education. Academic Medicine. 2000;75(1):71-3. | |
| 1206 | Rejected by TIAB Screening | Knauss LK. Supervisory issues related to treating children with serious emotional disturbance. Handbook of serious emotional disturbance in children and adolescents. Hoboken, NJ: John Wiley & Sons Inc; US; 2002. p. 112-27. | |
| 1207 | Rejected by TIAB Screening | Kneeland P, Pierce R, Ranji S, Dohan D, Vidyarthi A. A potent partnership: Empowering residents to lead a failure-modes-and-effects analysis for an academic medical center. Journal of Hospital Medicine. 2010;5:94. | |
| 1208 | Rejected by TIAB Screening | Knight L, Schroeder A, Wintch S, Arnolde V, Nichols A. Saving a life after discharge: CPR discharge training to parents of high-risk children. Critical Care Medicine. 2009;37 (12 SUPPL.):A261. | |
| 1209 | Rejected by TIAB Screening | Knoefel J, Herman C. Dementia care training for primary care providers: Project ECHOTM. Neurology Conference: 67th American Academy of Neurology Annual Meeting, AAN. 2015;84(SUPPL. 14). | |
| 1210 | Rejected by TIAB Screening | Knoefel J, Herman C. Project echoTM: Innovation in professional dementia care education and improved access to expertise, knowledge and skills. Journal of the American Geriatrics Society. 2015;63:S113. | |
| 1211 | Rejected by TIAB Screening | Knollmann-Ritschel BEC, Suarez E, Gilliland W, Conran R, Pock A. Pathology Course Director Perspectives of a Recent LCME Experience: Preparation in an Integrated Curriculum With the Revised Standards. Academic Pathology. 2017;4:2374289516687070. | |
| 1212 | Rejected by TIAB Screening | Knowles JK. Domestic violence: Florida licensed mental health professionals' perceived level of competence. Dissertation Abstracts International: Section B: The Sciences and Engineering. 2012;72(10-B):5877. | |
| 1213 | Rejected by TIAB Screening | Knowles MS. Applications in Continuing Education for the Health Professions. Chapter Five of "Andragogy in Action.". Mobius. 1985;5(2):80-100. | |
| 1214 | Rejected by TIAB Screening | Knox A, Merani S, Galleghar R, Pawa J, Logie N, Lewis M. Peer-assisted learning in undergraduate medical education: Promoting self-directed professional development and experience in medical education. Medical Education, Supplement. 2010;44:12. | |
| 1215 | Rejected by TIAB Screening | Knox AB. Life Long Self Directed Education. 1973. | |
| 1216 | Rejected by TIAB Screening | Knudsen JS, Aglen GS, Danbolt I, Engesnes N. Musical Pathfinders of the Kindergarten. Contemporary Issues in Early Childhood. 2019;20(2):163-76. | |
| 1217 | Rejected by TIAB Screening | Kocaman G, Dicle A, Ugur A. A longitudinal analysis of the self-directed learning readiness level of nursing students enrolled in a problem-based curriculum. Journal of Nursing Education. 2009;48(5):286-90. | |
| 1218 | Rejected by TIAB Screening | Koch J, Kubiak NT, Mitchell CK, Smith C, Bishop L, Burk M. An internal medicine didactic curriculum under goes a 21st century redesign. Journal of General Internal Medicine. 2016;31(2):S805. | |
| 1219 | Not Target Group | Koh J, Dubrowski A. Merging Problem-Based Learning with Simulation-Based Learning in the Medical Undergraduate Curriculum: The PAIRED Framework for Enhancing Lifelong Learning. Cureus. 2016;8(6):e647. | |
| 1220 | Rejected by TIAB Screening | Kohl-Hackert N, Krautter M, Andreesen S, Hoffmann K, Herzog W, Junger J, et al. Workplace learning: an analysis of students' expectations of learning on the ward in the Department of Internal Medicine. GMS Zeitschrift Fur Medizinische Ausbildung. 2014;31(4):Doc43. | |
| 1221 | Rejected by TIAB Screening | Kohler MJ, Slocum C, Siddiqui I, O'Connor K, Bolster MB. Implementation of a collaborative rheumatology and physiatry musculoskeletal ultrasound training program. Arthritis and Rheumatology. 2014;66:S879. | |
| 1222 | Rejected by TIAB Screening | Koivisto JM, Hannula L, Boje RB, Prescott S, Bland A, Rekola L, et al. Design-based research in designing the model for educating simulation facilitators. Nurse Education in Practice. 2018;29:206-11. | |
| 1223 | Rejected by TIAB Screening | Kokol P, Blazun Vosner H, Zeleznik D, Vosner J, Saranto K. Bibliometric Patterns of Research Literature Production on Nursing Informatics Competence. Journal of Nursing Education. 2015;54(10):565-71. | |
| 1224 | Rejected by TIAB Screening | Kong W, Wang Y, Yue J, Chen J, Peng Y, Zhang S, et al. [The establishment of paired-standardized patients and its application in the PBL teaching of otorhinolaryngology]. Lin Chuang Er Bi Yan Hou Tou Jing Wai Ke Za Zhi = Journal Of Clinical Otorhinolaryngology, Head, & Neck Surgery. 2008;22(16):764-5, 8. | |
| 1225 | Rejected by TIAB Screening | Konishi E, Saiki T, Kamiyama H, Nishiya K, Tsunekawa K, Imafuku R, et al. Improved cognitive apprenticeship clinical teaching after a faculty development program. Pediatrics International. 2020;62(5):542-8. | |
| 1226 | Rejected by TIAB Screening | Konrad M, Hornell A, Gomes AM, Olsen S, De Looy A. DIETS2: European dietetic advanced competences-EDAC 2012. Annals of Nutrition and Metabolism. 2013;1):1736-7. | |
| 1227 | Rejected by TIAB Screening | Kontio R, Hätönen H, Joffe G, Pitkänen A, Lahti M, Välimäki M. Impact of eLearning course on nurses' professional competence in seclusion and restraint practices: 9-month follow-up results of a randomized controlled study (ISRCTN32869544). Journal of psychiatric and mental health nursing [Internet]. 2013; 20(5):[411‐8 pp.]. Available from: https://www.cochranelibrary.com/central/doi/10.1002/central/CN-01124729/full. | |
| 1228 | Rejected by TIAB Screening | Kontio R, Pitkänen A, Joffe G, Katajisto J, Välimäki M. eLearning course may shorten the duration of mechanical restraint among psychiatric inpatients: a cluster-randomized trial. Nordic journal of psychiatry [Internet]. 2014; 68(7):[443‐9 pp.]. Available from: https://www.cochranelibrary.com/central/doi/10.1002/central/CN-01014458/full. | |
| 1229 | Rejected by TIAB Screening | Kooloos JG, de Waal Malefijt MC, Ruiter DJ, Vorstenbosch MA. Loosely-guided, self-directed learning versus strictly-guided, station-based learning in gross anatomy laboratory sessions. Anatomical sciences education. 2012;5(6):340-6. | |
| 1230 | Rejected by TIAB Screening | Koota E, Kaariainen M, Melender HL. Educational interventions promoting evidence-based practice among emergency nurses: A systematic review. International emergency nursing. 2018;41:51-8. | |
| 1231 | Rejected by TIAB Screening | Kopp S, Smith H. Developing effective web-based regional anesthesia education: a randomized study evaluating case-based versus non-case-based module design. Regional anesthesia and pain medicine [Internet]. 2011; 36(4):[336‐42 pp.]. Available from: https://www.cochranelibrary.com/central/doi/10.1002/central/CN-00811395/full. | |
| 1232 | Rejected by TIAB Screening | Korhonen T, Lammintakanen J. Web-based learning in professional development: Experiences of Finnish nurse managers. Journal of Nursing Management. 2005;13(6):500-7. | |
| 1233 | Rejected by TIAB Screening | Kossioni AE, Kavadella A, Tzoutzas I, Bakas A, Tsiklakis K, Bailey S, et al. The development of an exemplar e-module for the continuing professional development of European dentists. European Journal of Dental Education. 2013;17 Suppl 1:38-44. | |
| 1234 | Rejected by TIAB Screening | Kotnis GR, Wilkerson TA, Yantsides CP, Raab SS. Simulation based medical education (SBME) of intraoperative frozen section interpretation. Laboratory Investigation. 2011;91:434A. | |
| 1235 | Rejected by TIAB Screening | Kovacich J. Interdisciplinary team training on the information superhighway. Journal of Interprofessional Care. 1996;10(2):111-9. | |
| 1236 | Rejected by TIAB Screening | Kowlowitz V, Davenport CS, Palmer MH. Development and dissemination of Web-based clinical simulations for continuing geriatric nursing education. Journal of Gerontological Nursing. 2009;35(4):37-43. | |
| 1237 | Rejected by TIAB Screening | Koziol-Dube K, Burke G, Dimario FJ. Self-directed online learning of child neurology for medical students. Annals of Neurology. 2010;14):S134. | |
| 1238 | Rejected by TIAB Screening | Kranz C, Love A, Roche C. How to Write a Good Test Question: Nine Tips for Novice Nurse Educators. Journal of Continuing Education in Nursing. 2019;50(1):12-4. | |
| 1239 | Rejected by TIAB Screening | Kraus M, Mauch F, Ammann B, Cunningham M, Gebhard F. [Use of magnetic resonance imaging in orthopaedic trauma surgery: global needs analysis]. Unfallchirurg. 2014;117(3):190, 2-6. | |
| 1240 | Rejected by TIAB Screening | Krishna S, Francisco B, Balas E, König P, Graff G, Madsen R. Internet-enabled interactive multimedia asthma education program: a randomized trial. Pediatrics [Internet]. 2003; 111(3):[503‐10 pp.]. Available from: https://www.cochranelibrary.com/central/doi/10.1002/central/CN-00431030/full. | |
| 1241 | Rejected by TIAB Screening | Krishnamurthy S, Borges N, Koles P. Interactive case vignette and slide-based pathology modules improve student understanding of disease processes. Laboratory Investigation. 2014;94:148A. | |
| 1242 | Not Target Group | Kubiak C, Rogers AM, Turner A. The Learning Experiences of Health and Social Care Paraprofessionals on a Foundation Degree. International Journal of Lifelong Education. 2010;29(3):373-86. | |
| 1243 | Rejected by TIAB Screening | Kuiper R. The effect of prompted self-regulated learning strategies in a clinical nursing preceptorship. (critical thinking). Dissertation Abstracts International: Section B: The Sciences and Engineering. 1999;60(4-B):1532. | |
| 1244 | Rejected by TIAB Screening | Kuiper R. Enhancing metacognition through the reflective use of self-regulated learning strategies. Journal of Continuing Education in Nursing. 2002;33(2):78-87. | |
| 1245 | Rejected by TIAB Screening | Kuiper RA. Nursing reflections from journaling during a perioperative internship. AORN Journal. 2004;79(1):195-8. | |
| 1246 | Rejected by TIAB Screening | Kulstad C, Lovell E. An innovative individualized interactive instruction curriculum. Annals of Emergency Medicine. 2012;60 (5):S170. | |
| 1247 | Rejected by TIAB Screening | Kumar JK, Gupta R, Basavaraj P, Singla A, Prasad M, Pandita V, et al. An insight into health care setup in national capital region of India using dimensions of learning organizations questionnaire (DLOQ)- A cross-sectional study. Journal of Clinical and Diagnostic Research. 2016;10(6):ZC01-ZC5. | |
| 1248 | Rejected by TIAB Screening | Kumaria S, Bhola P, Orlinsky DE. Influences that count: professional development of psychotherapists and counsellors in India. Asia Pacific Journal of Counselling and Psychotherapy. 2018;9(1):86-106. | |
| 1249 | Rejected by TIAB Screening | Kummerfeldt VD. Developing the next generation of clinical laboratory scientist supervisors: A study of supervisory competencies in clinical laboratory. Dissertation Abstracts International Section A: Humanities and Social Sciences. 2020;81(3-A):No Pagination Specified. | |
| 1250 | Rejected by TIAB Screening | Kuperstock JE, Horny M, Platt MP. Mobile app technology is associated with improved otolaryngology resident in-service performance. Laryngoscope. 2019;129(1):E15-E20. | |
| 1251 | Rejected by TIAB Screening | Kuramoto AM, Wyman JF. Design and Implementation of Effective Delivery Approaches for Continuing Nursing Education. Mobius. 1986;6(1):6-10. | |
| 1252 | Rejected by TIAB Screening | Kurtz M, Seltzer J, Shagan D, Thime W, Wexler B. Computer-assisted cognitive remediation in schizophrenia: what is the active ingredient? Schizophrenia research [Internet]. 2007; 89(1‐3):[251‐60 pp.]. Available from: https://www.cochranelibrary.com/central/doi/10.1002/central/CN-00576766/full. | |
| 1253 | Rejected by TIAB Screening | Kwok J, Liao W, Baxter S. Evaluation of an online peer fundus photograph matching program in teaching direct ophthalmoscopy to medical students. Canadian Journal of Ophthalmology. 2017;52(5):441-6. | |
| 1254 | Rejected by TIAB Screening | Kwok YLA, Callard M, McLaws ML. An automated hand hygiene training system improves hand hygiene technique but not compliance. American Journal of Infection Control. 2015;43(8):821-5. | |
| 1255 | Rejected by TIAB Screening | Kwon H, Cho J, Kim H, Song B, Ko S, Lee J, et al. Establishment of blood glucose monitoring system using the internet. Diabetes care [Internet]. 2004; 27(2):[478‐83 pp.]. Available from: https://www.cochranelibrary.com/central/doi/10.1002/central/CN-00481499/full. | |
| 1256 | Rejected by TIAB Screening | Kydd A, Fulford H. Access to Learning Opportunities for Residents in Care Homes: Reviewing the challenges and possibilities. Maturitas. 2020;140:14-23. | |
| 1257 | Accepted | Kyndt E, Vermeire E, Cabus S. Informal Workplace Learning among Nurses: Organisational Learning Conditions and Personal Characteristics That Predict Learning Outcomes. Journal of Workplace Learning. 2016;28(7):435-50. | |
| 1258 | Rejected by TIAB Screening | L'Abate L. Sourcebook of interactive practice exercises in mental health. New York, NY: Springer Science + Business Media; US; 2011. | |
| 1259 | Rejected by TIAB Screening | La Greca J, Forsyth K, McDonald W, Owen J. Medical education - Workshop: PREP Basic Training Portal - A virtual learning environment for the 21st century. Internal Medicine Journal. 2010;40:29. | |
| 1260 | Rejected by TIAB Screening | La Sota E, Di Corcia M, Daggy J. Obstetrician/gynecologists' attitudes, knowledge and caring of lesbian, bisexual, and transgender patients. Obstetrics and Gynecology. 2017;129 (Supplement 1):111S. | |
| 1261 | Rejected by TIAB Screening | Lajoie SP, Gube M. Adaptive expertise in medical education: Accelerating learning trajectories by fostering self-regulated learning. Medical Teacher. 2018;40(8):809-12. | |
| 1262 | Rejected by TIAB Screening | Lakhani A, Jan R, Baig M, Mubeen K, Ali SA, Shahid S, et al. Experiences of the graduates of the first baccalaureate midwifery programme in Pakistan: A descriptive exploratory study. Midwifery. 2018;59:94-9. | |
| 1263 | Rejected by TIAB Screening | Lakiotaki E, Vrasidas C, Armenski G, Brcic L, Brcik I, Smeets A, et al. Acquiring diagnostic experience in renal neoplastic pathology through HIPON web course. Virchows Archiv. 2014;465(1):S286. | |
| 1264 | Rejected by TIAB Screening | Lal MM. Keep the Momentum Going. Journal of Nursing Administration. 2020;50(6):305-6. | |
| 1265 | Rejected by TIAB Screening | Lambert S, Girgis A, Turner J, Kayser K, McComb V, Chambers S, et al. Use of the coping-together workbook by couples facing cancer: Insights into preferences for and barriers to self-directed learning. Asia-Pacific Journal of Clinical Oncology. 2011;7:134. | |
| 1266 | Rejected by TIAB Screening | Lambert S, Girgis A, Turner J, Kayser K, McComb V, Chambers S, et al. Preliminary findings of a qualitative evaluation of a coping skills intervention for couples facing cancer: Insights into preferences for self-directed learning. Psycho-Oncology. 2011;20:22-3. | |
| 1267 | Rejected by TIAB Screening | Lammintakanen J, Kivinen T. Continuing Professional Development in Nursing: Does Age Matter? Journal of Workplace Learning. 2012;24(1):34-47. | |
| 1268 | Rejected by TIAB Screening | Lana-Peixoto MA. The education of neurologists for the XXI century. [Portuguese]. Arquivos de neuro-psiquiatria. 1993;51(3):409-15. | |
| 1269 | Rejected by TIAB Screening | Landers K. Enchanging at the Crossroads...Research and Practice. Program and Proceedings for the Annual Adult Education Research Conference (27th, Syracuse, New York, May 23-25, 1986). Collected Works - Proceedings Reports - Research. Syracuse Univ., NY.; 1986. | |
| 1270 | Rejected by TIAB Screening | Landry AY, Hearld LR. Did we learn everything we need to know in school?: An evaluation of executive workplace learning in healthcare organizations. Leadership & Organization Development Journal. 2013;34(2):164-81. | |
| 1271 | Rejected by TIAB Screening | Lange L, Haak S, Lincoln M, Thompson C, Turner C, Weir C, et al. Use of Iliad to improve diagnostic performance of nurse practitioner students. Journal of nursing education [Internet]. 1997; 36(1):[36‐45 pp.]. Available from: https://www.cochranelibrary.com/central/doi/10.1002/central/CN-00135510/full. | |
| 1272 | Rejected by TIAB Screening | Langenbach M. Curriculum Models in Adult Education1993. | |
| 1273 | Rejected by TIAB Screening | Langenthal SF. Infant Developmental Assessment: A Self-Study Guide for School Psychologists 1984. | |
| 1274 | Outside SDL | Lanier J. Lifelong learning: how different journeys can lead to a single goal. Ohio Nurses Review. 2010;85(4):10-1. | |
| 1275 | Rejected by TIAB Screening | Lanzilotti SS, et al. The Practice Integrated Learning Sequence: Linking Education with the Practice of Medicine. Adult Education Quarterly. 1986;37(1):38-47. | |
| 1276 | Rejected by TIAB Screening | Laota Z. Counseling refugees: Examining mental health professionals' learning experiences and recommendations for effective training. Dissertation Abstracts International Section A: Humanities and Social Sciences. 2020;81(7-A):No Pagination Specified. | |
| 1277 | Rejected by TIAB Screening | LaPaglia D. Training healers from the heart. PsycCRITIQUES [Internet]. 2014; 59(17):[No Pagination Specified p.]. Available from: http://ovidsp.ovid.com/ovidweb.cgi?T=JS&CSC=Y&NEWS=N&PAGE=fulltext&D=psyc11&AN=2014-11027-001. | |
| 1278 | Rejected by TIAB Screening | Larisey MM. Student Self-Assessment: A Tool for Learning. Adult Learning. 1994;5(6):9-10. | |
| 1279 | Rejected by TIAB Screening | Larkin E, O'Connor K, McNally P. Improving e-learning environments for tomorrow's paediatricians. Archives of Disease in Childhood. 2019;104 (Supplement 3):A257. | |
| 1280 | Rejected by TIAB Screening | LaRocco DJ, Bruns DA. It&apos;s Not the "What," It&apos;s the "How": Four Key Behaviors for Authentic Leadership in Early Intervention. Young Exceptional Children. 2013;16(2):33-44. | |
| 1281 | Rejected by TIAB Screening | Larsen D. Student perceptions of a self-regulated learning program in a neurology clerkship. Neurology Conference: 67th American Academy of Neurology Annual Meeting, AAN. 2015;84(SUPPL. 14). | |
| 1282 | Rejected by TIAB Screening | Larsen D, Wesevich A, Lichtenfeld J, Varpio L. Student self-regulated learning: How collaboration and the clinical environment impact success. Neurology Conference: 68th American Academy of Neurology Annual Meeting, AAN. 2016;86(16 SUPPL. 1). | |
| 1283 | Rejected by TIAB Screening | Larsen DP, Naismith RT, Margolis M. High-Frequency Learning Goals: Using Self-Regulated Learning to Influence Day-to-Day Practice in Clinical Education. Teaching & Learning in Medicine. 2017;29(1):93-100. | |
| 1284 | Rejected by TIAB Screening | Larsen DP, Wesevich A, Lichtenfeld J, Artino AR, Jr., Brydges R, Varpio L. Tying knots: an activity theory analysis of student learning goals in clinical education. Medical Education. 2017;51(7):687-98. | |
| 1285 | Rejected by TIAB Screening | Larson DB. Tackling the problem of error in diagnostic radiology. Pediatric Radiology. 2015;45(6):790-2. | |
| 1286 | Rejected by TIAB Screening | Larson PR, Chege P, Dahlman B, Gibson C, Evensen A, Colon-Gonzalez MC, et al. Future of Family Medicine Faculty Development in Sub-Saharan Africa. Family Medicine. 2017;49(3):203-10. | |
| 1287 | Rejected by TIAB Screening | Latham CE. President's message. Continuous learning: key to thriving in turbulent times. Anna Journal. 1998;25(4):376-7. | |
| 1288 | Formal Teaching | Leach DC, Fletcher SW. Perspectives on continuing education in the health professions: improving health care through lifelong learning. Chest. 2008;134(6):1299-303. | |
| 1289 | Rejected by TIAB Screening | Lear DW. Spanish for Working Medical Professionals: Linguistic Needs. Foreign Language Annals. 2005;38(2):223-35. | |
| 1290 | Rejected by TIAB Screening | Lee C, Asher SR, Chutinan S, Gallucci GO, Ohyama H. The Relationship Between Dental Students' Assessment Ability and Preclinical and Academic Performance in Operative Dentistry. Journal of Dental Education. 2017;81(3):310-7. | |
| 1291 | Accepted | Lee DY. The influence of individual and organizational variables on informal learning among nurses in Korean hospitals. Dissertation Abstracts International Section A: Humanities and Social Sciences. 2018;79(9-A(E)):No Pagination Specified. | |
| 1292 | Rejected by TIAB Screening | Lee EE, Chin CM, Mani A. A triple threat: Developing a clinical informatics rotation to improve resident informatics knowledge, incorporate systems based practice, and expose residents to a rapidly growing field. Journal of General Internal Medicine. 2018;33 (2 Supplement 1):681-2. | |
| 1293 | Rejected by TIAB Screening | Lee L, Chao Y, Huang C, Fang J, Wang S, Chuang C, et al. Cognitive Style and Mobile E-Learning in Emergent Otorhinolaryngology-Head and Neck Surgery Disorders for Millennial Undergraduate Medical Students: randomized Controlled Trial. Journal of medical Internet research [Internet]. 2018; 20(2):[e56 p.]. Available from: https://www.cochranelibrary.com/central/doi/10.1002/central/CN-01935254/full. | |
| 1294 | Rejected by TIAB Screening | Lee M, Brown L, Bender J, Machan J, Overly F. A medical simulation-based educational intervention for emergency medicine residents in neonatal resuscitation. Academic emergency medicine [Internet]. 2012; 19(5):[577‐85 pp.]. Available from: https://www.cochranelibrary.com/central/doi/10.1002/central/CN-00839871/full. | |
| 1295 | Rejected by TIAB Screening | Lee MK. Effects of mobile phone-based app learning compared to computer-based web learning on nursing students: pilot randomized controlled trial. Healthcare Informatics Research. 2015;21(2):125-33. | |
| 1296 | Rejected by TIAB Screening | Lee MM, et al. Development and Testing of Self-Instruction Programs in Nutrition for Dental Students. Journal of Dental Education. 1981;45(6):344-48. | |
| 1297 | Rejected by TIAB Screening | Lee S. The development of a novel CME program to enhance emergency medicine care in rural areas: The SEME (supplemental emergency medicine experience) program. Academic Emergency Medicine. 2015;22(5):S122-S3. | |
| 1298 | Rejected by TIAB Screening | Lee S, Kim DH, Chae SM. Self-directed learning and professional values of nursing students. Nurse Education in Practice. 2020;42:102647. | |
| 1299 | Rejected by TIAB Screening | Lee SH, Neutze JA, Mosher TJ, Beatty-Chadha J. Improving feedback sharing culture in radiology. Diagnosis. 2018;5 (4):eA124. | |
| 1300 | Rejected by TIAB Screening | Lee TW, Lee SH, Kim HH, Kang SJ. Effective intervention strategies to improve health outcomes for cardiovascular disease patients with low health literacy skills: A systematic review. Asian Nursing Research. 2012;6(4):128-36. | |
| 1301 | Rejected by TIAB Screening | Lee-Kim YN, Whittle S, Porea T, Fruge E, Gramatges M. Strategies for implementing an effective Morbidity, Mortality, and Improvement (MMI) conference. Pediatric Blood and Cancer. 2018;65 (Supplement 1):S153-S4. | |
| 1302 | Rejected by TIAB Screening | Lefebvre GG, Shore EM. Avoiding complications by a hands-on mentor programme. Best Practice and Research: Clinical Obstetrics and Gynaecology. 2016;35:3-12. | |
| 1303 | Rejected by TIAB Screening | Lehmann LS, Sulmasy LS, Desai S, Acp Ethics P, Human Rights C. Hidden Curricula, Ethics, and Professionalism: Optimizing Clinical Learning Environments in Becoming and Being a Physician: A Position Paper of the American College of Physicians. Annals of Internal Medicine. 2018;168(7):506-8. | |
| 1304 | Rejected by TIAB Screening | Lehmann R, Seitz A, Meyburg J, Hoppe B, Hoffmann GF, Tonshoff B, et al. Pediatric in-hospital emergencies: real life experiences, previous training and the need for training among physicians and nurses. BMC Research Notes. 2019;12(1):19. | |
| 1305 | Rejected by TIAB Screening | Leibenluft E. Irritability and disruptive mood dysregulation disorder. Journal of the American Academy of Child and Adolescent Psychiatry. 2017;56 (10):S143-S4. | |
| 1306 | Rejected by TIAB Screening | Leicher V, Mulder RH. Individual and contextual factors influencing engagement in learning activities after errors at work: A replication study in a German Retail Bank. Journal of Workplace Learning. 2016;28(2):66-80. | |
| 1307 | Rejected by TIAB Screening | Lekalakala-Mokgele E. Facilitation in problem-based learning: Experiencing the locus of control. Nurse Education Today. 2010;30(7):638-42. | |
| 1308 | Rejected by TIAB Screening | Lemaire JB, Miller EN, Polachek AJ, Wong H. Stakeholder Groups' Unique Perspectives About the Attending Physician Preceptor Role: A Qualitative Study. Journal of General Internal Medicine. 2019;34(7):1158-66. | |
| 1309 | Rejected by TIAB Screening | Lemkin AB, Noska A, Graham K, Trappey B, McQuilkin P, Kempf J, et al. Creating collaborative connections across a continent: Seven children's hospitals striving for a sustainable global health partnership. Annals of Global Health. 2014;80 (3):168. | |
| 1310 | Rejected by TIAB Screening | Lemma F, McLaren S, Boudioni M, Woods LP. Training experiences, educational priorities and career plans of primary care staff. Education for Primary Care. 2007;18(1):67-75. | |
| 1311 | Rejected by TIAB Screening | Lenburg CB. Open learning and Career Mobility in Nursing. CVMosby Co,StLouis,Mo. 1975:397 p. $US 11.05/-. | |
| 1312 | Rejected by TIAB Screening | Lenburg CB. Do External Degree Programs Really Work? Nursing Outlook. 1990;38(5):234-38. | |
| 1313 | Rejected by TIAB Screening | Lerchenfeldt S, Ferrari T, Nyland R, Patino G. Autonomic Nervous System Team-Based Learning Module. Mededportal Publications. 2016;12:10507. | |
| 1314 | Rejected by TIAB Screening | Lerner C, Dombro AL, Powers S. Learning & Growing Together: Understanding and Supporting Your Child's Development = Aprender y crecer juntos: Como comprender y fomentar el desarrollo de sus hijos [with] Tip Sheets: Ideas for Professionals in Programs That Serve Young Children and Their Families. Guides - Non-Classroom. Zero to Three: National Center for Infants, Toddlers and Families, Washington, DC.; 2001. | |
| 1315 | Rejected by TIAB Screening | Lesher DC, Bomberger AS. The Roving Inservice--An Innovative Approach to Learning. Journal of Continuing Education in Nursing. 1983;14(3):19-22. | |
| 1316 | Rejected by TIAB Screening | Lesyk JJ. A School Psychologist's Self-Study Guide to Sport Psychology. Journal of Applied School Psychology. 2005;21(2):169-85. | |
| 1317 | Rejected by TIAB Screening | Leung E, Dix D, Ford J, Barnard D, McBride E. The pediatric hematology/oncology educational laboratory in-training examination (PHOELIX): A formative evaluation of laboratory skills for Canadian pediatric hematology/oncology trainees. Pediatric Blood and Cancer. 2015;62(11):1952-5. | |
| 1318 | Rejected by TIAB Screening | Levchuk JW. Self-directed learning of hospital pharmacy residents in western Canada. American Journal of Hospital Pharmacy. 1983;40(1):78-83. | |
| 1319 | Rejected by TIAB Screening | Levett-Jones TL. Self-directed learning: Implications and limitations for undergraduate nursing education. Nurse Education Today. 2005;25(5):363-8. | |
| 1320 | Rejected by TIAB Screening | Levine BH, Albucher RC. Patient management exercises in psychiatry. Arlington, VA: American Psychiatric Association; US; 2011. | |
| 1321 | Rejected by TIAB Screening | Lew KY, Ang SK, Chiang J, Chan SY, Chew SF, Lim ST, et al. Oncology clinical pharmacists at a cancer centre in Singapore. Proceedings of Singapore Healthcare. 2011;20:96. | |
| 1322 | Rejected by TIAB Screening | Lewis S, Fitzgerald E, Collins N, Flinn K, O'Gorman C, Murphy AM. Student and teacher experience of case-based E-learning in paediatrics. Archives of Disease in Childhood. 2019;104 (Supplement 3):A257-A8. | |
| 1323 | Rejected by TIAB Screening | Leyva KA, Burke-Doe A. The use of a 3 dimensional anatomy application to enhance engagement and satisfaction of graduate students enrolled in anatomy courses in a physical and occupational therapy program. FASEB Journal Conference: Experimental Biology. 2018;32(1 Supplement 1). | |
| 1324 | Not Target Group | Li H, Wang Z, Jiang N, Liu Y, Wen D. Lifelong learning of Chinese rural physicians: preliminary psychometrics and influencing factors. BMC Medical Education. 2015;15:192. | |
| 1325 | Rejected by TIAB Screening | Li QL, Li J, Chen ML, Xie HF, Li YP, Chen X. Comparison of three problem-based learning conditions (real patients, digital and paper) with lecture-based learning in a dermatology course: a prospective randomized study from China. Medical teacher. 2013;35(2):e963-70. | |
| 1326 | Not Target Group | Li S-TT, Paterniti DA, Co JPT, West DC. Successful self-directed lifelong learning in medicine: A conceptual model derived from qualitative analysis of a national survey of pediatric residents. Academic Medicine. 2010;85(7):1229-36. | |
| 1327 | Rejected by TIAB Screening | Li S-TT, Paterniti DA, Tancredi DJ, Co JPT, West DC. Is residents' progress on individualized learning plans related to the type of learning goal set? Academic Medicine. 2011;86(10):1293-9. | |
| 1328 | Rejected by TIAB Screening | Li ST, Favreau MA, West DC. Pediatric resident and faculty attitudes toward self-assessment and self-directed learning: a cross-sectional study. BMC Medical Education. 2009;9:16. | |
| 1329 | Not Target Group | Li ST, Paterniti DA, Co JP, West DC. Successful self-directed lifelong learning in medicine: a conceptual model derived from qualitative analysis of a national survey of pediatric residents. Academic Medicine. 2010;85(7):1229-36. | |
| 1330 | Rejected by TIAB Screening | Li ST, Paterniti DA, Tancredi DJ, Burke AE, Trimm RF, Guillot A, et al. Resident Self-Assessment and Learning Goal Development: Evaluation of Resident-Reported Competence and Future Goals. Academic pediatrics. 2015;15(4):367-73. | |
| 1331 | Rejected by TIAB Screening | Li ST, Tancredi DJ, Burke AE, Guillot A, Guralnick S, Trimm RF, et al. Self-assessment on the competencies and reported improvement priorities for pediatrics residents. Journal of Graduate Medical Education. 2012;4(4):445-53. | |
| 1332 | Rejected by TIAB Screening | Li STT, Tancredi DJ, Co JPT, West DC. Factors Associated with Successful Self-Directed Learning Using Individualized Learning Plans During Pediatric Residency. Academic Pediatrics. 2010;10(2):124-30. | |
| 1333 | Rejected by TIAB Screening | Liang G. Layered system evaluation in improving nurses' practice of outonomous learning enthusiasm. Cancer Nursing. 2017;40 (6 Supplement 1):E40. | |
| 1334 | Rejected by TIAB Screening | Liang HF, Wu KM, Weng CH, Hsieh HW. Nurses' Views on the Potential Use of Robots in the Pediatric Unit. Journal of Pediatric Nursing. 2019;47:e58-e64. | |
| 1335 | Rejected by TIAB Screening | Liaw S, Wong L, Ang S, Ho J, Siau C, Ang E. Strengthening the afferent limb of rapid response systems: an educational intervention using web-based learning for early recognition and responding to deteriorating patients. BMJ quality & safety [Internet]. 2016; 25(6):[448‐56 pp.]. Available from: https://www.cochranelibrary.com/central/doi/10.1002/central/CN-01158691/full. | |
| 1336 | Rejected by TIAB Screening | Liaw SY, Chan SWc, Scherpbier A, Rethans JJ, Pua GG. Recognizing, responding to and reporting patient deterioration: Transferring simulation learning to patient care settings. Resuscitation. | |
| 1337 | Rejected by TIAB Screening | Liebert CA, Mazer L, Bereknyei Merrell S, Lin DT, Lau JN. Student perceptions of a simulation-based flipped classroom for the surgery clerkship: A mixed-methods study. Surgery. 2016;160(3):591-8. | |
| 1338 | Rejected by TIAB Screening | Liljedahl M. On learning in the clinical environment. Perspectives on Medical Education. 2018;7(4):272-5. | |
| 1339 | Rejected by TIAB Screening | Liljedahl M, Bjorck E, Kalen S, Ponzer S, Bolander Laksov K. To belong or not to belong: nursing students' interactions with clinical learning environments - an observational study. BMC medical education. 2016;16:197. | |
| 1340 | Rejected by TIAB Screening | Lilley SH, Clay M, Greer A, Harris J, Cummings HD. Interdisciplinary Rural Health Training for Health Professional Students: Strategies for Curriculum Design. Journal of Allied Health. 1998;27(4):208-12. | |
| 1341 | Rejected by TIAB Screening | Lillis S, Van Dyk V. Workplace-based assessment for vocational registration of international medical graduates. The Journal of continuing education in the health professions. 2014;34(4):260-4. | |
| 1342 | Rejected by TIAB Screening | Lim D, Schoo A, Lawn S, Litt J. Embedding and sustaining motivational interviewing in clinical environments: a concurrent iterative mixed methods study. BMC Medical Education. 2019;19(1):164. | |
| 1343 | Rejected by TIAB Screening | Lim FA, Nadeau CA. Student-Led Interest Groups: An Adjunct to Learner-Centered Nursing Education. Nursing Education Perspectives. 2016;37(4):232-5. | |
| 1344 | Rejected by TIAB Screening | Lim JK, Golub RM. Graduate Medical Education Research in the 21 st Century and JAMA On Call. JAMA: Journal of the American Medical Association. 2004;292(23):2913-5. | |
| 1345 | Rejected by TIAB Screening | Lim LM, Chiu LH, Dohrmann J, Tan KL. Registered nurses' medication management of the elderly in aged care facilities. International Nursing Review. 2010;57(1):98-106. | |
| 1346 | Rejected by TIAB Screening | Lin C-C, Han C-Y, Pan I, Chen L-C. The teaching-learning approach and critical thinking development: A qualitative exploration of Taiwanese nursing students. Journal of Professional Nursing. 2015;31(2):149-57. | |
| 1347 | Rejected by TIAB Screening | Lin EM, Aikin JL, Bailey W, Fitzgerald B, Mings D, Mitchell S, et al. Improving ambulatory oncology nursing practice. An innovative educational approach. Cancer Nursing. 1993;16(1):53-62. | |
| 1348 | Rejected by TIAB Screening | Lin YH, Chen CH. Reflections on nursing education in Taiwan and its prospects from the perspective of adult education. [Chinese]. Hu li za zhi The journal of nursing. 2007;54(1):11-6. | |
| 1349 | Rejected by TIAB Screening | Linares AZ. Learning style preference, self-directed learning readiness and locus of control of baccalaureate nursing students. Dissertation Abstracts International. 1988;49(3-B):683-4. | |
| 1350 | Rejected by TIAB Screening | Lindblad M, Flink M, Ekstedt M. Safe medication management in specialized home healthcare - an observational study. BMC Health Services Research. 2017;17(1):598. | |
| 1351 | Rejected by TIAB Screening | Lindeman BM, Law JK, Lipsett PA, Arbella T, Stem M, Lidor AO. A blended online curriculum in the basic surgery clerkship: A pilot study. American Journal of Surgery. 2015;209(1):145-51. | |
| 1352 | Rejected by TIAB Screening | Linden K, Renaud J, Zohr R, Gaudet M, Haddad A, Pantarotto J, et al. Clinical Specialist Radiation Therapist in Palliative Radiation Therapy: Report of an Orientation, Training, and Support Program. Journal of Medical Imaging and Radiation Sciences. 2019;50(4):543-50. | |
| 1353 | Rejected by TIAB Screening | Lindgren S, Lancaster RJ. Encouraging Specialty Certification: How Multilevel Support Can Help. Journal of Continuing Education in Nursing. 2016;47(2):49-51. | |
| 1354 | Rejected by TIAB Screening | Lingeman B, Mazza L. Self-directed learning for staff development. Journal of Continuing Education in Nursing. 1986;17(3):100. | |
| 1355 | Rejected by TIAB Screening | Links M. Learning to be an advanced trainer. Asia-Pacific Journal of Clinical Oncology. 2012;8:54. | |
| 1356 | Rejected by TIAB Screening | Linn BS, Zeppa R. Effect of the surgical clerkship on student perception of essential skills and self-assessed abilities. Annals of Surgery. 1982;195(1):50-4. | |
| 1357 | Rejected by TIAB Screening | Linné A, Liedholm H. Effects of an interactive CD-program on 6 months readmission rate in patients with heart failure - a randomised, controlled trial. BMC cardiovascular disorders [Internet]. 2006; 6:[30 p.]. Available from: https://www.cochranelibrary.com/central/doi/10.1002/central/CN-00566060/full. | |
| 1358 | Rejected by TIAB Screening | Liou S-R, Cheng C-Y, Tsai H-M, Chang C-H. Innovative strategies for teaching nursing research in Taiwan. Nursing Research. 2013;62(5):335-43. | |
| 1359 | Rejected by TIAB Screening | Liu CH, Tang WR, Weng WH, Lin YH, Chen CY. The process of coping with stress by Taiwanese medical interns: a qualitative study. BMC Medical Education. 2016;16:10. | |
| 1360 | Rejected by TIAB Screening | Liu HY, Hilty DM. The central role of professional development and psychiatry. Psychiatric Clinics of North America. 2019;42(3):xiii-xv. | |
| 1361 | Rejected by TIAB Screening | Liu HY, Tsai HM, Wang IT, Chen NH. Predictors of self-perceived levels of creative teaching behaviors among nursing school faculty in Taiwan: A preliminary study. Journal of Professional Nursing. 2020;36(3):171-6. | |
| 1362 | Rejected by TIAB Screening | Liu KJ, Tkachenko E, Waldman A, Boskovski MT, Hartman RI, Levin AA, et al. A video-based, flipped classroom, simulation curriculum for dermatologic surgery: A prospective, multi-institution study. Journal of the American Academy of Dermatology. 2019;81(6):1271-6. | |
| 1363 | Rejected by TIAB Screening | Liu KP, Chan CC, Hui-Chan CW. Clinical reasoning and the occupational therapy curriculum. Occupational Therapy International. 2000;7(3):173-83. | |
| 1364 | Rejected by TIAB Screening | Liu T, Luo J, He H, Zheng J, Zhao J, Li K. History-taking instruction for baccalaureate nursing students by virtual patient training: A retrospective study. Nurse Education Today. 2018;71:97-104. | |
| 1365 | Rejected by TIAB Screening | Lloyd B, Pfeiffer D, Dominish J, Heading G, Schmidt D, McCluskey A. The New South Wales Allied Health Workplace Learning Study: barriers and enablers to learning in the workplace. BMC Health Services Research. 2014;14:134. | |
| 1366 | Rejected by TIAB Screening | Lloyd G, Skarratts D, Robinson N, Reid C. Communication skills training for emergency department senior house officers - A qualitative study. Journal of Accident and Emergency Medicine. 2000;17(4):246-50. | |
| 1367 | Rejected by TIAB Screening | Lo WL, Lin YG, Pan YJ, Wu YJ, Hsieh MC. Faculty development program for general medicine in Taiwan: Past, present, and future. Tzu Chi Medical Journal. 2014;26(2):64-7. | |
| 1368 | Rejected by TIAB Screening | Lobchuk MM. [Humanistic care--a vital dialog with myself as a nurse]. Pflege. 1996;9(2):120-6. | |
| 1369 | Rejected by TIAB Screening | Lock LK, Schnell Z, Pratt-Mullen J. A Mixed Model Design Study of RN to BS Distance Learning:Survey of Graduates' Perceptions of Strengths and Challenges. Online Journal of Distance Learning Administration. 2011;14(3). | |
| 1370 | Rejected by TIAB Screening | Lockspeiser TM, Li ST, Burke AE, Rosenberg AA, Dunbar AE, 3rd, Gifford KA, et al. In Pursuit of Meaningful Use of Learning Goals in Residency: A Qualitative Study of Pediatric Residents. Academic Medicine. 2016;91(6):839-46. | |
| 1371 | Rejected by TIAB Screening | Lockspeiser TM, Schmitter PA, Lane JL, Hanson JL, Rosenberg AA, Park YS. Assessing residents' written learning goals and goal writing skill: validity evidence for the learning goal scoring rubric. Academic Medicine. 2013;88(10):1558-63. | |
| 1372 | Rejected by TIAB Screening | Lockyer J, Armson H. Learning theories in medical education: How do theories inform curriculum design and assessment along the continuum of medical education? Medical Education, Supplement. 2010;44:61-2. | |
| 1373 | Rejected by TIAB Screening | Loka SR, Doshi D, Kulkarni S, Baldava P, Adepu S. Effect of reflective thinking on academic performance among undergraduate dental students. Journal of Education & Health Promotion. 2019;8:184. | |
| 1374 | Rejected by TIAB Screening | Long HB, et al. Current Developments in Self-Directed Learning. Collected Works - General Reports - Research Opinion Papers. 1996. | |
| 1375 | Rejected by TIAB Screening | Long S, Dawes C, Harley E. Incorporating AAP breastfeeding curriculum into medical student elective. Breastfeeding Medicine. 2014;9:S-21. | |
| 1376 | Rejected by TIAB Screening | Lonnemann M. Expanding the physical therapy classroom through the use of an international wiki. Physiotherapy (United Kingdom). 2011;97:eS1542. | |
| 1377 | Rejected by TIAB Screening | Lopez-Bover M, Mackrell M, Luck J, Leyva A. Growing your teaching methodology in bone marrow transplant (BMT) staff education: Going beyond PowerPoint. Biology of Blood and Marrow Transplantation. 2015;21(2):S125. | |
| 1378 | Rejected by TIAB Screening | Loughlin KR, Granatir T, Jordan GH. Continuing Medical Education and Maintenance of Certification. Urology Practice. 2016;3(6):481-5. | |
| 1379 | Rejected by TIAB Screening | Loughlin-Presnal JE, Bierman KL. Promoting parent academic expectations predicts improved school outcomes for low-income children entering kindergarten. Journal of school psychology. 2017;62:67-80. | |
| 1380 | Rejected by TIAB Screening | Lovin BK. Professional Learning through Workplace Partnerships. New Directions for Adult and Continuing Education n55 p61. 1992;69. | |
| 1381 | Rejected by TIAB Screening | Lowdermilk D, Fishel A. Computer simulations as a measure of nursing students' decision-making skills. Journal of nursing education [Internet]. 1991; 30(1):[34‐9 pp.]. Available from: https://www.cochranelibrary.com/central/doi/10.1002/central/CN-00073283/full. | |
| 1382 | Rejected by TIAB Screening | Lowe MM, Aparicio A, Galbraith R, Dorman T, Dellert E, American College of Chest Physicians H, et al. The future of continuing medical education: effectiveness of continuing medical education: American College of Chest Physicians Evidence-Based Educational Guidelines. Chest. 2009;135(3 Suppl):69S-75S. | |
| 1383 | Rejected by TIAB Screening | Lu CF, Wu SM, Shu YM, Yeh MY. [Applying Game-Based Learning in Nursing Education: Empathy Board Game Learning]. Hu Li Tsa Chih - Journal of Nursing. 2018;65(1):96-103. | |
| 1384 | Rejected by TIAB Screening | Lubarda J, Braun R, Hanauer SB. Impact of online continuing medical education on physician knowledge of pathogenesis and biologic therapies in inflammatory bowel disease. American Journal of Gastroenterology. 2015;110:S795. | |
| 1385 | Rejected by TIAB Screening | Lubarda J, Chatterjee P, Muino J, Chey WD. Improving diagnosis and management of IBS-D through continuing medical education. American Journal of Gastroenterology. 2017;112 (Supplement 1):S1468. | |
| 1386 | Rejected by TIAB Screening | Lubarda J, Dermer S, Chatterjee P, Hanifin J, Lio P. Making the connection: Physician knowledge on PDE4 inhibitors in atopic dermatitis. Journal of the American Academy of Dermatology. 2017;76 (6 Supplement 1):AB164. | |
| 1387 | Rejected by TIAB Screening | Lubarda J, Smith S, Johnson K. The Dash to Educate on Nash: Does Medical Education Improve Physician Knowledge? Gastroenterology. 2019;156 (6 Supplement 1):S-1236. | |
| 1388 | Rejected by TIAB Screening | Lubbe W, Botha CS. The dimensions of reflective practice: A teacher educator's and nurse educator's perspective. Reflective Practice. 2020:No Pagination Specified. | |
| 1389 | Rejected by TIAB Screening | Lucas G, Dressler DD. Preparing medical students for the future, rather than the present. Journal of General Internal Medicine. 2015;30:S505. | |
| 1390 | Rejected by TIAB Screening | Lucieer SM, van der Geest JN, Eloi-Santos SM, de Faria RMD, Jonker L, Visscher C, et al. The Development of Self-Regulated Learning during the Pre-Clinical Stage of Medical School: A Comparison between a Lecture-Based and a Problem-Based Curriculum. Advances in Health Sciences Education. 2016;21(1):93-104. | |
| 1391 | Rejected by TIAB Screening | Luconi F. Exploring rural family physicians' learning from a web-based continuing medical education program on Alzheimer's disease: A pilot study. Dissertation Abstracts International Section A: Humanities and Social Sciences. 2010;70(12-A):4574. | |
| 1392 | Rejected by TIAB Screening | Luk Suet Ching W. The Influence of a Distance-Learning Environment on Students' Field Dependence/Independence. Journal of Experimental Education. 1998;66(2):149-60. | |
| 1393 | Rejected by TIAB Screening | Lumley-Leger K, McNeil RC, Fisher A, Miller T, Draper S. Supporting the implementation of stroke care clinical practice guidelines through online self-directed learning. Canadian Journal of Neurological Sciences. 2010;37(3):S104. | |
| 1394 | Rejected by TIAB Screening | Lundberg N. Integrating learning contexts in a medical informatics program - Preparing for the introduction of PACS. International Journal of Medical Informatics. 1998;50 (1-3):87-93. | |
| 1395 | Rejected by TIAB Screening | Lunyk-Child OI, Crooks D, Ellis PJ, Ofosu C, O'Mara L, Rideout E. Self-directed learning: faculty and student perceptions. Journal of Nursing Education. 2001;40(3):116-23. | |
| 1396 | Rejected by TIAB Screening | Lutz G, Scheffer C, Edelhaeuser F, Tauschel D, Neumann M. A reflective practice intervention for professional development, reduced stress and improved patient care - A qualitative developmental evaluation. Patient Education and Counseling. 2013;92(3):337-45. | |
| 1397 | Rejected by TIAB Screening | Lwin AT, Lwin T, Naing P, Oo Y, Kidd D, Cerullo M, et al. Self-Directed Interactive Video-Based Instruction Versus Instructor-Led Teaching for Myanmar House Surgeons: A Randomized, Noninferiority Trial. Journal of Surgical Education. 2018;75(1):238-46. | |
| 1398 | Rejected by TIAB Screening | Lyons V. Competencies 101: The knowledge, skills, behaviors and attitudes of a top notch anatomy educator. FASEB Journal Conference: Experimental Biology. 2015;29(1 Meeting Abstracts). | |
| 1399 | Rejected by TIAB Screening | Lyons-Warren AM, Kirby JP, Larsen DP. Student views on the role of self-regulated learning in a surgery clerkship. Journal of Surgical Research. 2016;206(2):273-9. | |
| 1400 | Rejected by TIAB Screening | Lysack C, Dama M, Neufeld S, Andreassi E. A compliance and satisfaction with home exercise: a comparison of computer-assisted video instruction and routine rehabilitation practice. Journal of allied health [Internet]. 2005; 34(2):[76‐82 pp.]. Available from: https://www.cochranelibrary.com/central/doi/10.1002/central/CN-00523341/full. | |
| 1401 | Rejected by TIAB Screening | Lyte VJ, Thompson IG. The diary as a formative teaching and learning aid incorporating means of evaluation and renegotiation of clinical learning objectives. Nurse Education Today. 1990;10(3):228-32. | |
| 1402 | Rejected by TIAB Screening | Ma X, Yang Y, Wang X, Zang Y. An integrative review: Developing and measuring creativity in nursing. Nurse Education Today. 2018;62:1-8. | |
| 1403 | Rejected by TIAB Screening | Mac Giolla Phadraig C, Griffiths C, McCallion P, McCarron M, Wride MA, Nunn JH. How dentists learn behaviour support skills for adults with intellectual developmental disorders: A qualitative analysis. European Journal of Dental Education. 2020;22:22. | |
| 1404 | Rejected by TIAB Screening | Macallan DC, Kent A, Holmes SC, Farmer EA, McCrorie P. A model of clinical problem-based learning for clinical attachments in medicine. Medical Education. 2009;43(8):799-807. | |
| 1405 | Rejected by TIAB Screening | MacDougall C, Schwartz BS, Kim L, Nanamori M, Shekarchian S, Chin-Hong PV. An interprofessional curriculum on antimicrobial stewardship improves knowledge and attitudes toward appropriate antimicrobial use and collaboration. Open Forum Infectious Diseases. 2017;4(1). | |
| 1406 | Rejected by TIAB Screening | Mackay FD, Atkinson PR, Lewis D. Medicus doces te ipsum: A study in self-directed ultrasound learning. Canadian Journal of Emergency Medicine. 2015;17 (Supplement 2):S44. | |
| 1407 | Rejected by TIAB Screening | Mackay FD, Zhou F, Lewis D, Fraser J, Atkinson PR. Can You Teach Yourself Point-of-care Ultrasound to a Level of Clinical Competency? Evaluation of a Self-directed Simulation-based Training Program. Cureus. 2018;10(9):e3320. | |
| 1408 | Rejected by TIAB Screening | MacKenzie C. The Politics of Representation: A Personal Reflection on the Problematic Positioning of the Midwifery Educator. Studies in Continuing Education. 2004;26(1):117-28. | |
| 1409 | Rejected by TIAB Screening | MacKenzie JJ, Stockley D, Hastings-Truelove A, Nowlan Suart T, Katsoulas E, Kawaja M, et al. Student Reflections on the Queen's Accelerated Route to Medical School Programme. Journal of Medical Education & Curricular Development. 2019;6:2382120519836789. | |
| 1410 | Rejected by TIAB Screening | Mackin J. Self-directed learning: video programs. Nursing Staff Development Insider. 1992;1(3):2-3. | |
| 1411 | Rejected by TIAB Screening | Mackin J. Self-directed learning. To be a great facilitator, don't follow these steps. Nursing Staff Development Insider. 1993;2(4):3, 7. | |
| 1412 | Rejected by TIAB Screening | Mackin J. Self-directed learning: teaching by testing. Nursing Staff Development Insider. 1993;2(5):1, 6-7. | |
| 1413 | Rejected by TIAB Screening | Mackin J. Four lessons on self-directed learning. Nursing Staff Development Insider. 1994;3(2):5, 8. | |
| 1414 | Rejected by TIAB Screening | Mackin J. Self-directed learning. Info-letters: the cure for the "out of touch" syndrome. Nursing Staff Development Insider. 1994;3(3):4-5, 7. | |
| 1415 | Rejected by TIAB Screening | MacKinnon KR, Ross LE, Rojas Gualdron D, Ng SL. Teaching health professionals how to tailor gender-affirming medicine protocols: A design thinking project. Perspectives on Medical Education. 2020;16:16. | |
| 1416 | Rejected by TIAB Screening | Macleod S. How to invest in the future GP: How the HPE scheme delivered this. Education for Primary Care. 2006;17(4):385-90. | |
| 1417 | Rejected by TIAB Screening | Madaiah S, Purva M. Impact of a leadership programme on front line quality improvement. Archives of Disease in Childhood. 2017;102 (Supplement 1):A84. | |
| 1418 | Rejected by TIAB Screening | Maddox TM, Albert NM, Borden WB, Curtis LH, Ferguson TB, Kao DP, et al. The learning healthcare system and cardiovascular care: A scientific statement from the American Heart Association. Circulation. 2017;135(14):e826-e57. | |
| 1419 | Rejected by TIAB Screening | Maertens H, Aggarwal R, Moreels N, Vermassen F, Van HI. A Proficiency Based Stepwise Endovascular Curricular Training (PROSPECT) Program Enhances Operative Performance in Real Life: a Randomised Controlled Trial. European journal of vascular and endovascular surgery [Internet]. 2017; 54(3):[387‐96 pp.]. Available from: https://www.cochranelibrary.com/central/doi/10.1002/central/CN-01446874/full. | |
| 1420 | Rejected by TIAB Screening | Maffettone A, Rinaldi M, Schettino M, Gatti A, Gargiulo A, Ussano L. Continuing educational learning: A useful tool for Clinical Governance in the treatment of diabetic inpatients. Project AMD Campania region (group management protocols in hospital). Italian Journal of Medicine. 2014;8:75. | |
| 1421 | Rejected by TIAB Screening | Mafinejad MK, Aghili R, Emami Z, Malek M, Baradaran H, Taghavinia M, et al. Study guides: Effective tools to improve self-directed learning skills of medical students. Acta Medica Iranica. 2014;52(10):781-5. | |
| 1422 | Rejected by TIAB Screening | Magalhaes P, Mourao R, Pereira R, Azevedo R, Pereira A, Lopes M, et al. Experiences During a Psychoeducational Intervention Program Run in a Pediatric Ward: A Qualitative Study. Frontiers in Pediatrics. 2018;6:124. | |
| 1423 | Rejected by TIAB Screening | Magola E, Willis S, Schafheutle E. Evaluation of a group coaching programme to support the transition of newly-qualified community pharmacists. International Journal of Pharmacy Practice. 2017;25 (Supplement 2):40-1. | |
| 1424 | Rejected by TIAB Screening | Magorian KG. Faculty Perceptions of Effective Practices for Utilizing a Framework to Develop a Concept-Based Curriculum in Nursing Education. ProQuest LLC EdD Dissertation, University of South Dakota. 2013. | |
| 1425 | Rejected by TIAB Screening | Mahmoud NN. Maintenance of certification: How to stay board certified by the American board of colon and rectal surgery. Seminars in Colon and Rectal Surgery. 2020;31(1). | |
| 1426 | Rejected by TIAB Screening | Mahmoudi G, Rahimi V, Fani F, Jahani MA, Mahmoudjanloo S. A comparison of the levels of organizational learning in hospitals, based on ownership types: A case study in Iran. Gazi Medical Journal. 2018;29(4):303-7. | |
| 1427 | Rejected by TIAB Screening | Main K, Schaefer C. Learning Projects of the Active Aging Eighty-Five and Over Population in the United States. Information Analyses Speeches/Meeting Papers. 1998. | |
| 1428 | Rejected by TIAB Screening | Major R, Tetley J. Recognising, managing and supporting dyslexia beyond registration. The lived experiences of qualified nurses and nurse academics. Nurse Education in Practice. 2019;37:146-52. | |
| 1429 | Rejected by TIAB Screening | Makai GE, Schaeffer KD, Sloan NL. Independent learning of electrosurgery in gynecology: A randomized controlled trial. Journal of Gynecologic Surgery. 2017;33(2):51-6. | |
| 1430 | Rejected by TIAB Screening | Makeeva V, Isbell JA, Caruthers K, Brooks W. The impact of team-based learning (TBL) on physician assistant student's academic performance in gross anatomy. FASEB Journal Conference: Experimental Biology. 2016;30(Meeting Abstracts). | |
| 1431 | Rejected by TIAB Screening | Makwabe EF, Pereira-Kamath N, Vincent L, Mngumi J, Kelly H, Pichan F, et al. Sat-489 Audit of a Whatsapp Based Continuing Education Platform to Facilitate on the Job Formal Learning to Improve Quality of Care Delivered among Haemodialysis Nurses. Kidney International Reports. 2020;5 (3 Supplement):S204. | |
| 1432 | Rejected by TIAB Screening | Malau-Aduli BS, Alele FO, Heggarty P, Teague PA, Sen Gupta T, Hays R. Perceived clinical relevance and retention of basic sciences across the medical education continuum. Advances in physiology education. 2019;43(3):293-9. | |
| 1433 | Accepted | Malekian M, Ghiyasvandian S, Cheraghi MA, Hassanzadeh A. Iranian Clinical Nurses' Readiness for Self-Directed Learning. Global Journal of Health Science. 2015;8(1):157-64. | |
| 1434 | Rejected by TIAB Screening | Malekzadeh S, Smith RV. 11th annual academic bowl. Otolaryngology - Head and Neck Surgery (United States). 2017;157 (1 Supplement 1):P14-P5. | |
| 1435 | Rejected by TIAB Screening | Maley MAL, Harvey JR, de Boer BWB, Scott NW, Arena GE. Addressing current problems in teaching pathology to medical students: Blended learning. Medical Teacher. 2008;30(1):e1-e9. | |
| 1436 | Rejected by TIAB Screening | Mallinson AM, McGrath AJ, Marwick J. The use of an online learning module (LearnPro NHSTM) to educate and assess staff working in cystic fibrosis care. Journal of Cystic Fibrosis. 2018;17 (Supplement 3):S126. | |
| 1437 | Rejected by TIAB Screening | Malloch K. Living excellence: life after Magnet designation. Nursing Administration Quarterly. 2009;33(2):93-8. | |
| 1438 | Rejected by TIAB Screening | Mallory R, Jackson J. Factor analysis of highly rated attendings. Journal of General Internal Medicine. 2010;25:S275. | |
| 1439 | Rejected by TIAB Screening | Malone C, Boggs E, Menninger I. Self-directed laparoscopic box training: Training the trainees. BJOG: An International Journal of Obstetrics and Gynaecology. 2013;120:592-3. | |
| 1440 | Not Target Group | Malta S, Dimeo SB, Carey PD. Self-direction in learning: does it change over time? Journal of Allied Health. 2010;39(2):e37-41. | |
| 1441 | Outside SDL | Mamary, Charles P. Promoting Self-Directed Learning for Continuing Medical Education. Medical Teacher. 2003;25(2):188-90. | |
| 1442 | Rejected by TIAB Screening | Mansour M, Schlair S, Leung S, Arad D, LeFrancois D. Defining quality of resident work rounds: Impact of team size and PGY-level. Journal of General Internal Medicine. 2015;30:S133. | |
| 1443 | Rejected by TIAB Screening | Manterola Alvarez D. [Socio-economic and psycho-affective factors and their influence on academic performance of residents in Obstetrics and Gynecology]. Ginecologia y Obstetricia de Mexico. 2015;83(3):139-47. | |
| 1444 | Rejected by TIAB Screening | Manyalich M, Guasch X, Paez G, Valero R, Del Rio M. Professional training on organ donation program in the european training program for organ donation (ETPOD) project to raise donation consciousness and to encourage a positive attitude towards it. Transplantation. 2010;90:552. | |
| 1445 | Rejected by TIAB Screening | Marc M, Bartosiewicz A, Burzynska J, Chmiel Z, Januszewicz P. A nursing shortage - a prospect of global and local policies. International Nursing Review. 2019;66(1):9-16. | |
| 1446 | Not Target Group | Marelli L, Berlingieri P, Epstein O. Virtual reality curriculum in endoscopy: Modular self-directed training on simulator (simbionix gi mentor) using expert benchmark. Gastroenterology. 2011;140(5):S718. | |
| 1447 | Rejected by TIAB Screening | Markaki A, Wingo N, Watts P, Steadman L, Coles K, Rae T, et al. Building Capacity for Nursing and Midwifery Education Through an Asynchronous Online Course. The Journal of nursing education. 2020;59(1):38-41. | |
| 1448 | Rejected by TIAB Screening | Markert RJ. Medical Education at Wright State University: An Evaluation during the First Year of Residency by the Class of 1980 and Their Residency Supervisors. Program Evaluation Studies, Report Number 3. Reports - Evaluative. Wright State Univ., Dayton, OH.; 1981. | |
| 1449 | Rejected by TIAB Screening | Marques HMC. Postgraduate degree in advanced pharmaceutical technology. Pharmacy Education. 2010;10 (1):14-5. | |
| 1450 | Rejected by TIAB Screening | Marquez N, Acuna Parker V. The road to excellence in clinical education: Intersection between academia and clinical practice. Physiotherapy (United Kingdom). 2015;101:eS32-eS3. | |
| 1451 | Not Target Group | Marquez UC, Fasce HE, Perez VC, Ortega BJ, Parra PP, Ortiz ML, et al. [Relationship between self-directed learning with learning styles and strategies in medical students]. Revista Medica de Chile. 2014;142(11):1422-30. | |
| 1452 | Rejected by TIAB Screening | Marshall B, Sabbagh L. Effects of perceived educational support on usage of an internet nursing reference center. Journal of continuing education in nursing [Internet]. 2015; 46(4):[161‐8; quiz 9‐70 pp.]. Available from: https://www.cochranelibrary.com/central/doi/10.1002/central/CN-01109848/full. | |
| 1453 | Rejected by TIAB Screening | Marshall E, York J, Magruder K, Yeager D, Knapp R, De SM, et al. Implementation of online suicide-specific training for VA providers. Academic psychiatry [Internet]. 2014; 38(5):[566‐74 pp.]. Available from: https://www.cochranelibrary.com/central/doi/10.1002/central/CN-01118258/full. | |
| 1454 | Rejected by TIAB Screening | Marshall J, Stewart M, Ostbye T. Small-group CME using e-mail discussions. Can it work? Canadian family physician medecin de famille canadien [Internet]. 2001; 47:[557‐63 pp.]. Available from: https://www.cochranelibrary.com/central/doi/10.1002/central/CN-00327615/full. | |
| 1455 | Rejected by TIAB Screening | Marsick VJ. Human service organizations as communities of learning. Education through community organizations. San Francisco, CA: Jossey-Bass; US; 1990. p. 45-54. | |
| 1456 | Rejected by TIAB Screening | Martin J. The challenge of introducing continuous professional development for paramedics. Journal of Emergency Primary Health Care. 2006;4(2). | |
| 1457 | Rejected by TIAB Screening | Martin JC, Avant RF, Bowman MA, Bucholtz JR, Dickinson JR, Evans KL, et al. The Future of Family Medicine: a collaborative project of the family medicine community. Annals of Family Medicine. 2004;2 Suppl 1:S3-32. | |
| 1458 | Rejected by TIAB Screening | Martin LC, Arenas-Montoya NM, Barnett TO. Impact of Nurse Certification Rates on Patient Satisfaction and Outcomes: A Literature Review. Journal of Continuing Education in Nursing. 2015;46(12):549-54; quiz 55-6. | |
| 1459 | Rejected by TIAB Screening | Martin P, Duffy T, Johnston B, Banks P, Harkess-Murphy E, Martin CR. Family health nursing: a response to the global health challenges. Journal of family nursing. 2013;19(1):99-118. | |
| 1460 | Rejected by TIAB Screening | Masatoshi N, et al., editors. The State of Continuing Education in Japan1994. | |
| 1461 | Rejected by TIAB Screening | Mason J, Loader K. Using a serious game to train violence risk assessment and management skills. Simulation & Gaming. 2019;50(2):124-35. | |
| 1462 | Rejected by TIAB Screening | Mather CA, Cummings EA, Gale F. Advancing mobile learning in Australian healthcare environments: nursing profession organisation perspectives and leadership challenges. BMC Nursing. 2018;17:44. | |
| 1463 | Rejected by TIAB Screening | Mathew R, Gucciardi E, De Melo M, Barata P. Self-management experiences among men and women with type 2 diabetes mellitus: a qualitative analysis. BMC Family Practice. 2012;13:122. | |
| 1464 | Rejected by TIAB Screening | Matsuo K, Rosenheck RE, Tierney KE, Tsao-Wei DD, Groshen SL, Pham HQ, et al. Paper or bandwidth: How do physicians continue learning? Gynecologic Oncology. 2011;123 (2):440. | |
| 1465 | Rejected by TIAB Screening | Matsuo O, Takahashi Y, Abe C, Tanaka K, Nakashima A, Morita H. Trial of integrated laboratory practice. Advances in Physiology Education. 2011;35(2):237-40. | |
| 1466 | Not Target Group | Matsuyama Y, Nakaya M, Okazaki H, Leppink J, van der Vleuten C. Contextual attributes promote or hinder self-regulated learning: A qualitative study contrasting rural physicians with undergraduate learners in Japan. Medical Teacher. 2018;40(3):285-95. | |
| 1467 | Rejected by TIAB Screening | Mattheos N. The Internet and the oral healthcare professionals: potential and challenges of a new era. International Journal of Dental Hygiene. 2007;5(3):151-7. | |
| 1468 | Rejected by TIAB Screening | Matthews DT, Maguire J, Carson SS. Lower attending physician-to-patient ratio improves resident perception of intensive care unit education. American Journal of Respiratory and Critical Care Medicine Conference: American Thoracic Society International Conference, ATS. 2013;187(MeetingAbstracts). | |
| 1469 | Rejected by TIAB Screening | Matthews K, Wright C, Osborne C. Blending work-integrated learning with distance education in an Australian radiation therapy advanced practice curriculum. Radiography. 2014;20(3):277-82. | |
| 1470 | Rejected by TIAB Screening | Mauksch L, Farber S, Greer H. Design, dissemination, and evaluation of an advanced communication elective at seven U.S. medical schools. Academic Medicine. 2013;88(6):843-51. | |
| 1471 | Rejected by TIAB Screening | Maxwell RS, Pounder DJ. The Medicine and Human Rights special study module: A Physicians for Human Rights (UK) initiative. Medical Teacher. 1999;21(3):294-8. | |
| 1472 | Rejected by TIAB Screening | Maxwell SM. WebQuests: A viable solution to meeting the continuing professional education needs of home health care nurses. Home Health Care Management & Practice. 2009;21(3):171-6. | |
| 1473 | Rejected by TIAB Screening | May M, Fletcher M. Eat what you love with diabetes: How to use the mindful eating cycle for self-management. Diabetes. 2013;62:A624. | |
| 1474 | Rejected by TIAB Screening | Mazerolle SM, Walker SE, Kirby JL. Support Received During the Transition to Practice for the Secondary School Graduate-Assistant Athletic Trainer. Journal of Athletic Training. 2016;51(10):780-8. | |
| 1475 | Rejected by TIAB Screening | McAllister A, Aanstoot J, Hammarstrom IL, Samuelsson C, Johannesson E, Sandstrom K, et al. Learning in the tutorial group: a balance between individual freedom and institutional control. Clinical Linguistics & Phonetics. 2014;28(1-2):47-59. | |
| 1476 | Rejected by TIAB Screening | McAllister M. Learning contracts: an Australian experience. Nurse education today. 1996;16(3):199-205. | |
| 1477 | Rejected by TIAB Screening | McAllister M, Troyer M, Kearns L. Assessment and management of social determinants of health in the primary care setting, an internal medicine resident curriculum. Journal of General Internal Medicine. 2019;34 (2 Supplement):S790. | |
| 1478 | Rejected by TIAB Screening | McAloon C, Leach H, Gill S, Aluwalia A, Trevelyan J. Improving ECG Competence in Medical Trainees in a UK District General Hospital. Cardiology Research. 2014;5(2):51-7. | |
| 1479 | Rejected by TIAB Screening | McArdle P, Grainger J. Why study healthcare ethics? [References]. Foundations of healthcare ethics: Theory to practice. New York, NY: Cambridge University Press; US; 2015. p. 1-13. | |
| 1480 | Rejected by TIAB Screening | McBrearty M, Wilson S, Finch A. Competency assessment for nurse led blood and platelet ordering - A competency too far? Transfusion Medicine. 2010;20:41. | |
| 1481 | Rejected by TIAB Screening | McCabe MJ. Analysis of Learning Styles of Nursing Students at University of Central Florida. Curriculum and Program Planning 1983. | |
| 1482 | Rejected by TIAB Screening | McCarthy C. Learner engagement in anaesthesia. Irish Journal of Medical Science. 2017;186 (3 Supplement 1):S155-S6. | |
| 1483 | Rejected by TIAB Screening | McClurg C, Powelson S, Lang E, Aghajafari F, Edworthy S. Evaluating effectiveness of small group information literacy instruction for Undergraduate Medical Education students using a pre- and post-survey study design. Health information and libraries journal. 2015;32(2):120-30. | |
| 1484 | Rejected by TIAB Screening | McCluskey A, Cusick A. Strategies for introducing evidence-based practice and changing clinician behaviour: A manager's toolbox. Australian Occupational Therapy Journal. 2002;49(2):63-70. | |
| 1485 | Rejected by TIAB Screening | McConaghy JR. Evolving medical knowledge: moving toward efficiently answering questions and keeping current. Primary Care; Clinics in Office Practice. 2006;33(4):831-7, v. | |
| 1486 | Rejected by TIAB Screening | McConnaughey S, Freeman R, Kim S, Sheehan F. Integrating Scaffolding and Deliberate Practice Into Focused Cardiac Ultrasound Training: A Simulator Curriculum. Mededportal Publications. 2018;14:10671. | |
| 1487 | Rejected by TIAB Screening | McConville B, Skibowski E. Trainee led ultrasound teaching in regional anaesthesia in a district general hospital. Anaesthesia. 2014;69:96. | |
| 1488 | Rejected by TIAB Screening | McCrea H. Motivation for continuing education in midwifery. Midwifery. 1989;5(3):134-45. | |
| 1489 | Rejected by TIAB Screening | McDonald L. A comparison of teaching methods based on Kolb learning styles in a nurse anesthesia program. Dissertation Abstracts International Section A: Humanities and Social Sciences. 2018;78(12-A(E)):No Pagination Specified. | |
| 1490 | Rejected by TIAB Screening | McDonald WJ. Council of Medical Specialty Societies: Committed to Continuing Medical Education Reform. Journal of Continuing Education in the Health Professions. 2005;25(3):144-50. | |
| 1491 | Rejected by TIAB Screening | McDonough M, Marks I. Teaching medical students exposure therapy for phobia/panic - randomized, controlled comparison of face-to-face tutorial in small groups vs. solo computer instruction. Medical education [Internet]. 2002; 36(5):[412‐7 pp.]. Available from: https://www.cochranelibrary.com/central/doi/10.1002/central/CN-00380251/full. | |
| 1492 | Rejected by TIAB Screening | McGervey MA, Scott SC, Mahar JH, Mohmand MG, Isada C, Spencer A. Modified problem-based learning: An innovative approach to improve residency education, critical reasoning, and collaboration. Journal of General Internal Medicine. 2019;34 (2 Supplement):S832. | |
| 1493 | Rejected by TIAB Screening | McGinnis P, Guenther LA, Romen M, Wainwright S. Factors that influence development and integration of professional core values into physical therapy practice. Physiotherapy (United Kingdom). 2011;97:eS784-eS5. | |
| 1494 | Rejected by TIAB Screening | McGowan BS, Wasko M, Vartabedian BS, Miller RS, Freiherr DD, Abdolrasulnia M. Understanding the factors that influence the adoption and meaningful use of social media by physicians to share medical information. Journal of Medical Internet Research. 2012;14(5):e117. | |
| 1495 | Rejected by TIAB Screening | McGrae McDermott M, Curry RH, Stille FC, Martin GJ. Use of learning contracts in an office-based primary care clerkship. Medical Education. 1999;33(5):374-81. | |
| 1496 | Rejected by TIAB Screening | McGrath PJ. Informal learning experiences--less than continuing education courses, more than scanning the literature. Hospital Libraries. 1977;2(10):6-8. | |
| 1497 | Rejected by TIAB Screening | McHenry MS, Fischer LJ, Chun Y, Vreeman RC. A systematic review of portable electronic technology for health education in resource-limited settings. Global Health Promotion. 2019;26(2):70-81. | |
| 1498 | Rejected by TIAB Screening | McIntosh M, Konzelmann J, Smith J, Kalynych C, Wears R, Schneider H, et al. Stabilization and treatment of dental avulsions and fractures by emergency physicians using just-in-time training. Annals of emergency medicine [Internet]. 2009; 54(4):[585‐92 pp.]. Available from: https://www.cochranelibrary.com/central/doi/10.1002/central/CN-00718681/full. | |
| 1499 | Rejected by TIAB Screening | McKauge L, Coombes J. Quality use of medicine experiential placements for fourth year pharmacy students. Pharmacy Education. 2005;5(2):137-42. | |
| 1500 | Rejected by TIAB Screening | McKendry S, Boyd V. Defining the "Independent Learner" in UK Higher Education: Staff and Students' Understanding of the Concept. International Journal of Teaching and Learning in Higher Education. 2012;24(2):209-20. | |
| 1501 | Rejected by TIAB Screening | McKeown SI. Design and implementation of an innovative 'C4 program': Competency assessment, communication, continuous learning and career planning for respiratory therapists at Vancouver general hospital. Canadian Journal of Respiratory Therapy. 2010;46(3):33-5. | |
| 1502 | Rejected by TIAB Screening | McKinley DW. Capsule commentary on Gallagher et al., internists' attitudes about assessing and maintaining clinical competence. Journal of General Internal Medicine. 2014;29(4):649. | |
| 1503 | Rejected by TIAB Screening | McKinney K, Lineberry M, Conigliaro J. Weighing the influences: Advice seeking during residency training. Journal of General Internal Medicine. 2010;25:S429. | |
| 1504 | Rejected by TIAB Screening | McLaughlin C, Barin E, Ford H, Upperman J, Cassidy L, Burke RV. Formative research experiences in pediatric surgeons: a mixed methods study of Pediatric Trauma Society members. Pediatric Surgery International. 2019;35(4):495-9. | |
| 1505 | Rejected by TIAB Screening | McLellan JL, Taylor WH. Canadian Association for the Study of Adult Education Proceedings of the Annual Conference (7th, Calgary, Alberta, Canada, May 1988). Collected Works - Proceedings Reports - Research. Calgary Univ., Alberta. Faculty of Continuing Education.; 1988. | |
| 1506 | Rejected by TIAB Screening | McLeod GA, Vaughan B, Carey I, Shannon T, Winn E. Pre-professional reflective practice: Strategies, perspectives and experiences. International Journal of Osteopathic Medicine. 2020;35:50-6. | |
| 1507 | Rejected by TIAB Screening | McMahon G, Fonda S, Gomes H, Alexis G, Conlin P. A randomized comparison of online- and telephone-based care management with internet training alone in adult patients with poorly controlled type 2 diabetes. Diabetes technology & therapeutics [Internet]. 2012; 14(11):[1060‐7 pp.]. Available from: https://www.cochranelibrary.com/central/doi/10.1002/central/CN-00853780/full. | |
| 1508 | Rejected by TIAB Screening | McMahon G, Gomes H, Hickson HS, Hu T, Levine B, Conlin P. Web-based care management in patients with poorly controlled diabetes. Diabetes care [Internet]. 2005; 28(7):[1624‐9 pp.]. Available from: https://www.cochranelibrary.com/central/doi/10.1002/central/CN-00522822/full. | |
| 1509 | Rejected by TIAB Screening | McMahon MA, Christopher KA. Case study method and problem-based learning: utilizing the pedagogical model of progressive complexity in nursing education. International Journal of Nursing Education Scholarship. 2011;8:Article 22. | |
| 1510 | Formal Teaching | McMaster R, Lopez V, Cleary M. Lifelong learning and professional practice. Nursing & Health Sciences. 2018;20(1):1-3. | |
| 1511 | Rejected by TIAB Screening | McMillan DE, Wenger GR. Effects of curriculum and format changes in a medical pharmacology course, 1983 to 1987. Journal of Medical Education. 1987;62(10):836-41. | |
| 1512 | Rejected by TIAB Screening | McMillan WJ, Barrie RB. Recruiting and retaining rural students: evidence from a faculty of dentistry in South Africa. Rural and remote health. 2012;12:1855. | |
| 1513 | Rejected by TIAB Screening | McMullan M, Jones R, Lea S. The effect of an interactive e-drug calculations package on nursing students' drug calculation ability and self-efficacy. International Journal of Medical Informatics. 2011;80(6):421-30. | |
| 1514 | Rejected by TIAB Screening | McNamee LS, O'Brien FY, Botha JH. Student perceptions of medico-legal autopsy demonstrations in a student-centred curriculum. Medical Education. 2009;43(1):66-73. | |
| 1515 | Rejected by TIAB Screening | McNeil K, Mitchell R, Parker V. The paradoxical effects of workforce shortages on rural interprofessional practice. Scandinavian Journal of Caring Sciences. 2015;29(1):73-82. | |
| 1516 | Rejected by TIAB Screening | McRae KE, Davies GAL, Easteal RA, Smith GN. Creation of plastinated placentas as a novel teaching resource for medical education in obstetrics and gynaecology. Placenta. 2015;36(9):1045-51. | |
| 1517 | Rejected by TIAB Screening | McSherry R, Kell J, Pearce P. Clinical supervision and clinical governance. Nursing Times. 2002;98(23):30-2. | |
| 1518 | Rejected by TIAB Screening | McSwain C, et al. The Use of a Criterion Performance Checklist to Improve Efficiency and Effectiveness in a CPR Self-Teaching Program. Journal of Medical Education. 1979;54(9):736-38. | |
| 1519 | Not Target Group | Mead MS. The Effect of Self-Directed Learning Readiness and Online Course Quality Ratings on Student Satisfaction and Academic Performance in Undergraduate eLearning. ProQuest LLC PhD Dissertation, University of Missouri Kansas City. 2011. | |
| 1520 | Rejected by TIAB Screening | Meaney P, Tsima B, Sutton R, Steenhoff A, Kestler A, Irving S, et al. Novel training improves acquisition and retention of life support skills in resource limited settings. Critical Care Medicine. 2011;39:132. | |
| 1521 | Rejected by TIAB Screening | Meaney PA, Boulet JR, Sutton RM, Kestler AM, Tsima B, Shilkof-Ski NA, et al. Cognitive and skills acquisition of novice learners after infant cpr training of healthcare providers at secondary and tertiary hospitals in botswana. Pediatric Critical Care Medicine. 2011;12(3):A169. | |
| 1522 | Rejected by TIAB Screening | Meaney PA, Sutton RM, Tsima B, Steenhoff AP, Shilkofski N, Boulet JR, et al. Training hospital providers in basic CPR skills in Botswana: Acquisition, retention and impact of novel training techniques. Resuscitation. 2012;83(12):1484-90. | |
| 1523 | Rejected by TIAB Screening | Medalia A, Dorn H, Watras-Gans S. Treating problem-solving deficits on an acute care psychiatric inpatient unit. Psychiatry research [Internet]. 2000; 97(1):[79‐88 pp.]. Available from: https://www.cochranelibrary.com/central/doi/10.1002/central/CN-00330019/full. | |
| 1524 | Formal Teaching | Mehta N, Geissel K, Rhodes E, Salinas G. Comparative effectiveness in CME: evaluation of personalized and self-directed learning models. Journal of Continuing Education in the Health Professions. 2015;35 Suppl 1:S24-6. | |
| 1525 | Rejected by TIAB Screening | Meijer LJ, de Groot E, Blaauw-Westerlaken M, Damoiseaux RA. Intraprofessional collaboration and learning between specialists and general practitioners during postgraduate training: a qualitative study. BMC Health Services Research. 2016;16(a):376. | |
| 1526 | Rejected by TIAB Screening | Melady D. An innovative online curriculum for accredited continuing medical education in geriatric emergency medicine: www.geri-EM.com. Canadian Journal of Emergency Medicine. 2014;16:S37. | |
| 1527 | Rejected by TIAB Screening | Melburg V, Hanner MB. Student Outcomes Using a Cross-Sectional Design for Nursing External Degree Programs. Speeches/Meeting Papers Reports - Research. 1994. | |
| 1528 | Rejected by TIAB Screening | Melo Prado H, Hannois Falbo G, Rodrigues Falbo A, Natal Figueiroa J. Active learning on the ward: outcomes from a comparative trial with traditional methods. Medical Education. 2011;45(3):273-9. | |
| 1529 | Rejected by TIAB Screening | Melo S, Beck M. Intra and interorganizational learning networks and the implementation of quality improvement initiatives: The case of a Portuguese teaching hospital. Human Resource Development Quarterly. 2015;26(2):155-83. | |
| 1530 | Rejected by TIAB Screening | Melton RF. Planning and Developing Open and Distance Learning : A Quality Assurance Approach. Radiological Studies in Distance Education2002. | |
| 1531 | Rejected by TIAB Screening | Menahem S. The contribution of the paediatrician and psychiatrist to the management of the child, adolescent and his family--a paediatrician's viewpoint. Australian Paediatric Journal. 1987;23(4):235-9. | |
| 1532 | Rejected by TIAB Screening | Menahem S, Paget N. Role play for the clinical tutor: towards problem-based learning. Medical Teacher. 1990;12(1):57-61. | |
| 1533 | Rejected by TIAB Screening | Mendiratta P, Chaffee K, Goodin JB, Podrazik P. Novelty interprofessional geriatric skills bootcamp: 'booster session' and "flipped classroom". Journal of the American Geriatrics Society. 2017;65 (Supplement 1):S68. | |
| 1534 | Rejected by TIAB Screening | Mentkowski M, Doherty A. Careering After College: Establishing the Validity of Abilities Learned in College for Later Careering and Professional Performance. Final Report to the National Institute of Education. Overview and Summary. [Revised.]. Reports - Research. Alverno Coll., Milwaukee, WI.; 1984. | |
| 1535 | Not Target Group | Mercer P, Bailey H, Cook P. Perceptions, attitudes and opinions of general dental practitioners and dental nurses to the provision of lifelong learning for the dental team. British Dental Journal. 2007;202(12):747-53. | |
| 1536 | Rejected by TIAB Screening | Merchant R, Clark M, Langan T, Mayer K, Seage G, DeGruttola V. Can computer-based feedback improve emergency department patient uptake of rapid HIV screening? Annals of emergency medicine [Internet]. 2011; 58(1 Suppl 1):[S114‐9.e1‐2 pp.]. Available from: https://www.cochranelibrary.com/central/doi/10.1002/central/CN-00791224/full. | |
| 1537 | Not Target Group | Merchant VA. Lifelong learning: a requirement for life. Journal of Michigan Dental Association. 2014;96(6):14. | |
| 1538 | Rejected by TIAB Screening | Merino JL, Arribas F, Botto GL, Huikuri H, Kraemer LI, Linde C, et al. Core curriculum for the heart rhythm specialist: executive summary. Europace. 2009;11(10):1381-6. | |
| 1539 | Rejected by TIAB Screening | Mertens F, de Groot E, Meijer L, Wens J, Gemma Cherry M, Deveugele M, et al. Workplace learning through collaboration in primary healthcare: A BEME realist review of what works, for whom and in what circumstances: BEME Guide No. 46. Medical Teacher. 2018;40(2):117-34. | |
| 1540 | Rejected by TIAB Screening | Mesh LJ. Collaborative Language Learning for Professional Adults. Electronic Journal of e Learning. 2010;8(2):161-72. | |
| 1541 | Rejected by TIAB Screening | Mestdagh E, Van Rompaey B, Peremans L, Meier K, Timmermans O. Proactive behavior in midwifery: A qualitative overview from midwifery student's perspective. Nurse Education in Practice. 2018;31:1-6. | |
| 1542 | Rejected by TIAB Screening | Metcalfe K, Alg GS, Wright L, Merriman C, Thompson S. Is inter-professional education an effective way to teach geriatric medicine to medical and nursing students? European Geriatric Medicine. 2015;6:S95. | |
| 1543 | Rejected by TIAB Screening | Metz MJ, Durski MT, O'Malley DeGaris M, Daugherty TC, Vaught RL, Cornelius CJ, et al. Student Self-Assessment of Operative Dentistry Experiences: A Time-Dependent Exercise in Self-Directed Learning. Journal of Dental Education. 2017;81(5):571-81. | |
| 1544 | Rejected by TIAB Screening | Meyer A, Krishnamurthy P, Sur M, Payne V, Meeks D, Rao R, et al. Calibration of diagnostic accuracy and confidence in physicians working in academic and non-academic settings. Diagnosis. 2015;2 (1):eA10. | |
| 1545 | Rejected by TIAB Screening | Meyer K. Evidence-based medicine: Benefits, limitations and implementation strategies. International Pediatrics. 2006;21(4):239-43. | |
| 1546 | Rejected by TIAB Screening | Mickan S, Golenko X, Buys N. Exploring future health workforce educational needs: A qualitative investigation. Journal of Multidisciplinary Healthcare. 2019;12:527-32. | |
| 1547 | Rejected by TIAB Screening | Middleton R, Alvand A, Garfjeld RP, Hargrove C, Kirby G, Rees J. Simulation-Based Training Platforms for Arthroscopy: a Randomized Comparison of Virtual Reality Learning to Benchtop Learning. Arthroscopy [Internet]. 2017; 33(5):[996‐1003 pp.]. Available from: https://www.cochranelibrary.com/central/doi/10.1002/central/CN-01443837/full. | |
| 1548 | Rejected by TIAB Screening | Mihailidou A. Blood pressure measurement skills: Is there a need to standardize? Journal of Hypertension. 2015;33:e283. | |
| 1549 | Rejected by TIAB Screening | Mikulaninec C. Effects of mailed preoperative instructions on learning and anxiety. Patient education and counseling [Internet]. 1987; 10(3):[253‐65 pp.]. Available from: https://www.cochranelibrary.com/central/doi/10.1002/central/CN-00267007/full. | |
| 1550 | Rejected by TIAB Screening | Millde-Luthander C, Högberg U, Nyström M, Pettersson H, Wiklund I, Grunewald C. The impact of a computer assisted learning programme on the ability to interpret cardiotochography. A before and after study. Sexual & reproductive healthcare [Internet]. 2012; 3(1):[37‐41 pp.]. Available from: https://www.cochranelibrary.com/central/doi/10.1002/central/CN-00970585/full. | |
| 1551 | Not Target Group | Miller AE. Preface. Lifelong learning in neurology. CONTINUUM: Lifelong Learning in Neurology. 2012;18(3):513-4. | |
| 1552 | Rejected by TIAB Screening | Miller J, Vivona B, Roth G. Work Role Transitions--Expert Nurses to Novice Preceptors. European Journal of Training and Development. 2017;41(6):559-74. | |
| 1553 | Not Target Group | Miller Juve AK. Reflective Practice and Readiness for Self-Directed Learning in Anesthesiology Residents Training in the United States. ProQuest LLC EdD Dissertation, Portland State University. 2012. | |
| 1554 | Rejected by TIAB Screening | Miller P. Workplace learning by action learning: A practical example. Journal of Workplace Learning. 2003;15(1):14-23. | |
| 1555 | Rejected by TIAB Screening | Miller PM. Osteoporosis advocacy: Does it have a positive role in the college classroom? Osteoporosis International. 2011;22:S427-S8. | |
| 1556 | Rejected by TIAB Screening | Miller SH, Thompson JN, Mazmanian PE, Aparicio A, Davis DA, Spivey BE, et al. Continuing medical education, professional development, and requirements for medical licensure: A white paper of the conjoint committee on continuing medical education. Journal of Continuing Education in the Health Professions. 2008;28(2):95-8. | |
| 1557 | Rejected by TIAB Screening | Mills S, Krouse AM, Rossi-Schwartz R, Klein JM. Curriculum Revision: Student Stress and Lessons Learned. Journal of Nursing Education. 2017;56(6):337-42. | |
| 1558 | Outside SDL | Milne J. Nursing is lifelong learning. British Journal of Nursing. 2016;25(12):S3. | |
| 1559 | Rejected by TIAB Screening | Mistraletti G, Mantovani E, Cadringher P, Cerri B, Corbella D, Umbrello M, et al. Enteral vs. intravenous ICU sedation management: study protocol for a randomized controlled trial. Trials [Internet]. 2013; 14:[92 p.]. Available from: https://www.cochranelibrary.com/central/doi/10.1002/central/CN-00875129/full. | |
| 1560 | Rejected by TIAB Screening | Mistraletti G, Umbrello M, Anania S, Andrighi E, Di CA, Martinetti F, et al. Neurological assessment with validated tools in general ICU: multicenter, randomized, before and after, pragmatic study to evaluate the effectiveness of an e-learning platform for continuous medical education. Minerva anestesiologica [Internet]. 2017; 83(2):[145‐54 pp.]. Available from: https://www.cochranelibrary.com/central/doi/10.1002/central/CN-01955736/full. | |
| 1561 | Rejected by TIAB Screening | Mitchell H, Lucas C, Charlton K, McMahon A. Models of nutrition-focused continuing education programs for nurses: a systematic review of the evidence. Australian Journal of Primary Health. 2018;24(2):101-8. | |
| 1562 | Rejected by TIAB Screening | Mitchell R, Liu PL. A study of resident learning behavior. Teaching and Learning in Medicine. 1995;7(4):233-40. | |
| 1563 | Rejected by TIAB Screening | Mlott SR, Marcotte DB, Lira FT. The efficacy of programmed instruction in the training of paraprofessionals. Journal of Clinical Psychology. 1976;32(2):419-24. | |
| 1564 | Not Target Group | Mofrad MN, Karami M. The effects of portfolio method on self-directed learning (SDL) of nursing students. Biosciences Biotechnology Research Asia. 2013;10(2):817-21. | |
| 1565 | Rejected by TIAB Screening | Mogali SR, Vallabhajosyula R, Ng CH, Lim D, Ang ET, Abrahams P. Scan and Learn: Quick Response Code Enabled Museum for Mobile Learning of Anatomy and Pathology. Anatomical Sciences Education. 2019;12(6):664-72. | |
| 1566 | Rejected by TIAB Screening | Mohan S, Follansbee C, Nwankwo U, Hofkosh D, Sherman F, Hamilton M. Embedding patient simulation in a pediatric cardiology rotation: a unique opportunity for improving resident education. Congenital heart disease [Internet]. 2015; 10(1):[88‐94 pp.]. Available from: https://www.cochranelibrary.com/central/doi/10.1002/central/CN-01705155/full. | |
| 1567 | Rejected by TIAB Screening | Mohanaruban A, Flanders L, Rees H. Case-based discussion: perceptions of feedback. The clinical teacher. 2018;15(2):126-31. | |
| 1568 | Not Target Group | Moja L, Kwag KH. Point of care information services: a platform for self-directed continuing medical education for front line decision makers. Postgraduate Medical Journal. 2015;91(1072):83-91. | |
| 1569 | Rejected by TIAB Screening | Monroe KS. The Assessment of Self-Directed Learning Readiness in Medical Education. ProQuest LLC PhD Dissertation, Mercer University. 2014. | |
| 1570 | Formal Teaching | Monroe KS. The relationship between assessment methods and self-directed learning readiness in medical education. International Journal of Medical Education. 2016;7:75-80. | |
| 1571 | Rejected by TIAB Screening | Monroe KS, Evans MA, Mukkamala SG, Williamson JL, Jabaley CS, Mariano ER, et al. Moving anesthesiology educational resources to the point of care: Experience with a pediatric anesthesia mobile app. Korean Journal of Anesthesiology. 2018;71(3):192-200. | |
| 1572 | Rejected by TIAB Screening | Monrouxe LV, Bullock A, Gormley G, Kaufhold K, Kelly N, Roberts CE, et al. New graduate doctors' preparedness for practice: a multistakeholder, multicentre narrative study. BMJ Open. 2018;8(8):e023146. | |
| 1573 | Rejected by TIAB Screening | Monrouxe LV, Bullock A, Tseng HM, Wells SE. Association of professional identity, gender, team understanding, anxiety and workplace learning alignment with burnout in junior doctors: a longitudinal cohort study. BMJ Open. 2017;7(12):e017942. | |
| 1574 | Rejected by TIAB Screening | Monrouxe LV, Rees CE. "It's just a clash of cultures": emotional talk within medical students' narratives of professionalism dilemmas. Advances in Health Sciences Education. 2012;17(5):671-701. | |
| 1575 | Rejected by TIAB Screening | Monrouxe LV, Rees CE, Poole R, Madav U, John D, Oliver R. Healthcare students' narratives of professionalism dilemmas: A multi-centre study of workplace learning. Medical Education, Supplement. 2011;45:34. | |
| 1576 | Rejected by TIAB Screening | Monrouxe LV, Rees CE, Wells S, Linford H. UK medical students' professionalism dilemmas: Examining dilemma types and their correlates. Medical Education, Supplement. 2011;45:50. | |
| 1577 | Rejected by TIAB Screening | Monsieurs KG, De Regge M, Schelfout S, D'Hondt F, Mpotos N, Valcke M, et al. Efficacy of a self-learning station for basic life support refresher training in a hospital: A randomized controlled trial. European Journal of Emergency Medicine. 2012;19(4):214-9. | |
| 1578 | Rejected by TIAB Screening | Montenery S. Problem-Based Learning for Didactic Presentation to Baccalaureate Nursing Students. Creative Nursing. 2017;23(2):102-11. | |
| 1579 | Rejected by TIAB Screening | Monterrosa A. The subject "Women's Medicine" structured along the lines of "autonomous learning". [Spanish]. Clinica e Investigacion en Ginecologia y Obstetricia. 2003;30(5):157-62. | |
| 1580 | Not Target Group | Montin L, Koivisto JM. Effectiveness of self-directed learning methods compared with other learning methods in nursing education related to nursing students' or registered nurses' learning outcomes: A systematic review protocol. JBI Database of Systematic Reviews and Implementation Reports. 2014;12(2):1-8. | |
| 1581 | Rejected by TIAB Screening | Moore A, Fertleman C. Outcomes & perspectives of a paediatric iBSc course. Archives of Disease in Childhood. 2019;104 (Supplement 2):A256-A7. | |
| 1582 | Rejected by TIAB Screening | Moore DE, Jr., Green JS, Gallis HA. Achieving desired results and improved outcomes: integrating planning and assessment throughout learning activities. Journal of Continuing Education in the Health Professions. 2009;29(1):1-15. | |
| 1583 | Rejected by TIAB Screening | Moore DE, Jr., Pennington FC. Practice-based learning and improvement. Journal of Continuing Education in the Health Professions. 2003;23 Suppl 1:S73-80. | |
| 1584 | Rejected by TIAB Screening | Moore K, Vaughan B. Assessment of Australian osteopathic learners' clinical competence during workplace learning. International Journal of Osteopathic Medicine. 2016;19:50-60. | |
| 1585 | Rejected by TIAB Screening | Moore K, Vaughan B, Cox A. A pilot study to develop a tool for the assessment of students' clinical record keeping. International Journal of Osteopathic Medicine. 2017;24:37-41. | |
| 1586 | Rejected by TIAB Screening | Moore LJ. Ethical and Organisational Tensions for Work-Based Learners. Journal of Workplace Learning. 2007;19(3):161-72. | |
| 1587 | Outside SDL | Moore M, Van Schaik J, Montgomery CL. Physician retraining, lifelong learning, and the library. Bulletin of the Medical Library Association. 1992;80(4):374-6. | |
| 1588 | Rejected by TIAB Screening | Moradi Y, Ahmadi F, Sadeghi A, Oshvandi K. Conceptualizing and determining core clinical competencies in nursing students: a qualitative study. International Nursing Review. 2019;66(4):530-40. | |
| 1589 | Rejected by TIAB Screening | Moran V. Study of comparison of independent learning activities vs. attendance at staff development by staff nurses. Journal of Continuing Education in Nursing. 1977;8(3):14-21. | |
| 1590 | Rejected by TIAB Screening | Moran-Barrios J, De Gauna-Bahillo PR. Reinventing specialty training of physicians? Principles and challenges. [Spanish]. Nefrologia. 2010;30(6):604-12. | |
| 1591 | Rejected by TIAB Screening | Moreno Morales N, Casuso Holgado MJ, Labajos Manzanares MT, Rodriguez Martinez MDC, Baron Lopez FJ, Iglesias Parra MR, et al. Assessment of implementation of the EHEA in the degrees of the health sciences universitary college of malaga. Physiotherapy (United Kingdom). 2011;97:eS1493. | |
| 1592 | Rejected by TIAB Screening | Morgan HR. A Problem-Oriented Independent Studies Programme in Basic Medical Sciences. Medical Education. 1977;11(6):394-8. | |
| 1593 | Rejected by TIAB Screening | Mori B, Batty HP, Brooks D. The feasibility of an electronic reflective practice exercise among physiotherapy students. Medical Teacher. 2008;30(8):e232-e8. | |
| 1594 | Rejected by TIAB Screening | Moriya R. The Future of Pharmaceutical Education Based on Our Experience of Medical Education. Yakugaku Zasshi - Journal of the Pharmaceutical Society of Japan. 2017;137(4):413-9. | |
| 1595 | Rejected by TIAB Screening | Morony S, Weir K, Duncan G, Biggs J, Nutbeam D, McCaffery KJ. Enhancing communication skills for telehealth: development and implementation of a Teach-Back intervention for a national maternal and child health helpline in Australia. BMC health services research. 2018;18(1):162. | |
| 1596 | Rejected by TIAB Screening | Moroz A, Edgley SR, Lew HL, Chae J, Lombard LA, Reddy CC, et al. Rehabilitation Interventions in Parkinson Disease. PM and R. 2009;1(3 SUPPL.):S42-S8. | |
| 1597 | Rejected by TIAB Screening | Morris J. Evaluation of Open Learning Material Designed for Part of the Diploma Level Research Module for Pre- and Post-Registration Nurses. Nurse Education Today. 1999;19(8):601-09. | |
| 1598 | Rejected by TIAB Screening | Morrison EE. Complementary therapies: what are the ethics challenges for Kansas nurses? The Kansas nurse. 2007;82(5):3-5. | |
| 1599 | Outside SDL | Morrow G, Rothwell C, Wright P. Self-directed learning groups: a vital model for education, support and appraisal amongst sessional GPs. Education for Primary Care. 2012;23(4):270-6. | |
| 1600 | Rejected by TIAB Screening | Mosalanejad L, Razeghi B, Abdollahifard S. Educational game: A fun and team based learning in psychiatric course and its effects on learning indicators. Bangladesh Journal of Medical Science. 2018;17(4):631-7. | |
| 1601 | Rejected by TIAB Screening | Mossey P. The changing face of dental education. British Dental Journal. 2004(SUPPL.):3-4. | |
| 1602 | Rejected by TIAB Screening | Mott B, Horgan K, Flesch L, Hayward M, Demmel K, Morrison C. Moving a bone marrow transplant unit towards a high reliability unit. Biology of Blood and Marrow Transplantation. 2014;20(2):S305. | |
| 1603 | Rejected by TIAB Screening | Mousavi-Nasab N, Kraft A. Evolving roles: A look at the paths travelled by seven cytotechnologists at one academic institution. Journal of the American Society of Cytopathology. 2017;6 (5):S7-S8. | |
| 1604 | Rejected by TIAB Screening | Mulder ER, Bacon CEW, Edler JR, Hankemeier DA, Games KE, Van Lunen BL, et al. Motivators, Anticipated Challenges, and Supportive Factors for Athletic Trainers Pursuing the Doctor of Athletic Training Degree. Athletic Training Education Journal. 2018;13(2):148-57. | |
| 1605 | Rejected by TIAB Screening | Mullan Y, Miller A, Bertoni K, Jones H, Otto R, Zimmermann E. Lessons learned from our CSII working group. Canadian Journal of Diabetes. 2012;36(5):S28. | |
| 1606 | Rejected by TIAB Screening | Muller D. Reforming premedical education-Out with the old, in with the new. The New England Journal of Medicine. 2013;368(17):1567-9. | |
| 1607 | Outside SDL | Muller KE. Emotional intelligence and self-directed learning. Dissertation Abstracts International Section A: Humanities and Social Sciences. 2007;68(5-A):1782. | |
| 1608 | Rejected by TIAB Screening | Munasinghe SR, Senaviratne PAUK, Liyanapatabandi D, Olupeliyawa OM. Designing and implementation of a problem based learning (PBL) programme with real life case scenarios for the management of transfusion related adverse reactions. Vox Sanguinis. 2010;99:41. | |
| 1609 | Rejected by TIAB Screening | Murdoch-Eaton D, Jolly B. Undergraduate projects - Do they have to be within the conventional medical environment? Medical Education. 2000;34(2):95-100. | |
| 1610 | Rejected by TIAB Screening | Murphy A, Conley R, Ng V. Describing the study habits of emergency medicine residents, a preliminary analysis. Western Journal of Emergency Medicine. 2017;18 (Supplement 1):S10. | |
| 1611 | Rejected by TIAB Screening | Murphy J, Griffin C, Higgs B. Research-Teaching Linkages: Practice and Policy. Proceedings of the Third Annual Conference of the National Academy for the Integration of Research, Teaching and Learning (3rd, Dublin, Ireland, November 11-12, 2009). National Academy for Integration of Research, Teaching and Learning. 2010. | |
| 1612 | Rejected by TIAB Screening | Murphy N, Trovato M, Kim H, Kim CT, Moberg-Wolff E. Pediatric Rehabilitation: 2. Environmental Factors Affecting Participation. PM and R. 2010;2(3):S12-S8. | |
| 1613 | Rejected by TIAB Screening | Murray R, McKay E, Thompson S, Donald M. Practising reflection: A medical humanities approach to occupational therapist education. Medical Teacher. 2000;22(3):276-81. | |
| 1614 | Rejected by TIAB Screening | Murray SB, Levy M, Lord J, McLaren K. Peer-facilitated reflection: A tool for continuing professional development for faculty. Academic Psychiatry. 2013;37(2):125-8. | |
| 1615 | Rejected by TIAB Screening | Murrell DF. Contributions of the independent learning programme at University of New South Wales to dermatology registrar training 2006-2016. Australasian Journal of Dermatology. 2017;58 (Supplement 1):28. | |
| 1616 | Rejected by TIAB Screening | Murthy VK, Dhaliwal G, O'Brien B. An inquiry into the early careers of master clinicians. Diagnosis. 2018;5 (4):eA66. | |
| 1617 | Rejected by TIAB Screening | Muscarella M, Kranenburg-Van Koppen L, Grijpink-Van Den Biggelaar K, Drop SLS. Global application of disorders of sex development-related electronic resources: E-learning, e-consultation and e-information sharing. Endocrine Development. 2014;27:268-83. | |
| 1618 | Rejected by TIAB Screening | Mutabdzic D, Mylopoulos M, Murnaghan ML, Patel P, Zilbert N, Seemann N, et al. Coaching surgeons: Is culture limiting our ability to improve? Annals of Surgery. 2015;262(2):213-6. | |
| 1619 | Rejected by TIAB Screening | Muthukrishnan SP, Chandran DS, Afreen N, Bir M, Dastidar SG, Jayappa H, et al. Planning, Implementation, and Evaluation of Multicomponent, Case-Based Learning for First-Year Indian Medical Undergraduates. Advances in Physiology Education. 2019;43(4):504-11. | |
| 1620 | Rejected by TIAB Screening | Myers MH, Jr. Comparison of Print and Nonprint Media in a Nursing Learning Module [and] Development of Independent Learning Modules for Use in Nursing Continuing Education: Lessons Learned from the First Project of This Type. Reports - Descriptive Reports - Evaluative Speeches/Meeting Papers. 1981. | |
| 1621 | Rejected by TIAB Screening | Myers NA. A study of the learning strategies of metacognition, metamotivation, metamemory, critical thinking, and resource management of nursing students on a regional campus of a large midwestern university. Dissertation Abstracts International: Section B: The Sciences and Engineering. 1999;60(3-B):1029. | |
| 1622 | Rejected by TIAB Screening | Mylopoulos M, Woods NN. Having our cake and eating it too: Seeking the best of both worlds in expertise research. Medical Education. 2009;43(5):406-13. | |
| 1623 | Rejected by TIAB Screening | Nabaho L. Developing Generic Competences in Life Sciences: The Untold Story of the Makerere University College of Health Sciences in Uganda. Tuning Journal for Higher Education. 2017;4(2):389-406. | |
| 1624 | Rejected by TIAB Screening | Naeger DM, Straus CM, Phelps A, Courtier J, Webb EM. Student-created independent learning modules: An easy high-value addition to radiology clerkships. Academic Radiology. 2014;21(7):879-87. | |
| 1625 | Rejected by TIAB Screening | Nagendran M, Gurusamy KS, Aggarwal R, Loizidou M, Davidson BR. Virtual reality training for surgical trainees in laparoscopic surgery. Cochrane Database of Systematic Reviews. 2013(8). | |
| 1626 | Rejected by TIAB Screening | Nahata MC. Experience with an Independent Study Program in Pathophysiology for Doctor of Pharmacy Students. American Journal of Pharmaceutical Education. 1986;50(3):278-80. | |
| 1627 | Rejected by TIAB Screening | Nandiwada DR, Zimmer SM, McNamara M, McNeil M, Spagnoletti C, Kohli A, et al. Evidence based medicine on the fly: Interdisciplinary communication on the consult services. Journal of General Internal Medicine. 2016;31(2):S819. | |
| 1628 | Formal Teaching | Narang A, Velagapudi P, Rajagopalan B, LeBude B, Kithcart AP, Snipelisky D, et al. A New Educational Framework to Improve Lifelong Learning for Cardiologists. Journal of the American College of Cardiology. 2018;71(4):454-62. | |
| 1629 | Rejected by TIAB Screening | Narayanan RP, Kirk P, Lewis S. Uptake and perceptions of an e-learning package on blood transfusion by trainees in Wales. Journal of the Royal College of Physicians of Edinburgh. 2008;38(4):298-301. | |
| 1630 | Rejected by TIAB Screening | Nash S, Scammel J. Skills to ensure success in mentoring and other workplace learning approaches. Nursing Times. 2010;106(2):17-20. | |
| 1631 | Rejected by TIAB Screening | Nason GJ, Burke M, Akram M, Giri S, Flood HD. The use of smartphone applications by urology trainees. Irish Journal of Medical Science. 2014;183(1):S52. | |
| 1632 | Not Target Group | Nathaniel TI, Gainey JC, Williams JA, Stewart BL, Hood MC, Brechtel LE, et al. Impact and educational outcomes of a small group self-directed teaching strategy in a clinical neuroscience curriculum. Anatomical Sciences Education. 2018;11(5):478-87. | |
| 1633 | Rejected by TIAB Screening | Naur TMH, Nilsson PM, Pietersen PI, Clementsen PF, Konge L. Simulation-Based Training in Flexible Bronchoscopy and Endobronchial Ultrasound-Guided Transbronchial Needle Aspiration (EBUS-TBNA): A Systematic Review. Respiration. 2017;93(5):355-62. | |
| 1634 | Rejected by TIAB Screening | Nazim SM, Riaz Q, Ather MH. Effect of a two-day extensive continuing medical education course on participants' knowledge of clinical and operative urology. Turkish Journal of Urology. 2018;44(6):484-9. | |
| 1635 | Rejected by TIAB Screening | Nazira R, Venkatesha B, Bashirb AS, Sedkyc K, Lippmannb S. What residents should know before starting inpatient psychiatry? Eastern Journal of Medicine. 2012;17(3):105-10. | |
| 1636 | Rejected by TIAB Screening | Neher M, Stahl C, Ellstrom P-E, Nilsen P. Knowledge sources for evidence-based practice in rheumatology nursing. Clinical Nursing Research. 2015;24(6):661-79. | |
| 1637 | Rejected by TIAB Screening | Neher MS, Stahl C, Nilsen P. Learning Opportunities in Rheumatology Practice: A Qualitative Study. Journal of Workplace Learning. 2015;27(4):282-97. | |
| 1638 | Rejected by TIAB Screening | Neilan BA, et al. Clinical Oncology Assistantship Program for Medical Students. Journal of Medical Education. 1985;60(6):473-77. | |
| 1639 | Rejected by TIAB Screening | Neilan BA, Westbrook KC, Riggs C, Berry DH, Lang NP. Clinical oncology assistantship program for medical students. Journal of Medical Education. 1985;60(6):473-7. | |
| 1640 | Rejected by TIAB Screening | Nelson-Marten P, Skiba D, Howell S, Krebs LU. An innovative curriculum plan for advanced practice in oncology nursing. Journal of Cancer Education. 1997;12(2):89-94. | |
| 1641 | Rejected by TIAB Screening | Nemani VM, Park C, Nawabi DH. What makes a "great resident": the resident perspective. Current reviews in musculoskeletal medicine. 2014;7(2):164-7. | |
| 1642 | Rejected by TIAB Screening | Nembhard IM, Tucker AL. Deliberate learning to improve performance in dynamic service settings: Evidence from hospital intensive care units. Organization Science. 2011;22(4):907-22. | |
| 1643 | Rejected by TIAB Screening | Nemcek Jr AA. Vascular and interventional radiology training: What should it be? Seminars in Interventional Radiology. 1995;12(3):228-35. | |
| 1644 | Rejected by TIAB Screening | Nesbitt J, Barton G. Nursing journal clubs: A strategy for improving knowledge translation and evidenced-informed clinical practice invited manuscript for the journal of radiology nursing. Journal of Radiology Nursing. 2014;33(1):3-8. | |
| 1645 | Rejected by TIAB Screening | Nevala J, Coady A, Courtemanche J, Gallant V, Wong T. Online tuberculosis training for canadian physicians and nurses: A success story. Canadian Journal of Infectious Diseases and Medical Microbiology. 2013;24:19B. | |
| 1646 | Rejected by TIAB Screening | Nevalainen M, Lunkka N, Suhonen M. Work-based learning in health care organisations experienced by nursing staff: A systematic review of qualitative studies. Nurse Education in Practice. 2018;29:21-9. | |
| 1647 | Rejected by TIAB Screening | Newble DI, Hejka EJ. Approaches to learning of medical students and practising physicians: Some empirical evidence and its implications for medical education. Educational Psychology. 1991;11(3-4):333-42. | |
| 1648 | Rejected by TIAB Screening | Newton JM, Billett S, Ockerby CM. Journeying through clinical placements - An examination of six student cases. Nurse Education Today. 2009;29(6):630-4. | |
| 1649 | Rejected by TIAB Screening | Newton JM, White K, Cross WM, Ockerby C, Billett S. Outcomes of a clinical partnership model for undergraduate nursing students. Contemporary Nurse. 2011;39(1):119-27. | |
| 1650 | Rejected by TIAB Screening | Newton N, Vogl L, Teesson M, Andrews G. CLIMATE Schools: alcohol module: cross-validation of a school-based prevention programme for alcohol misuse. Australian and New Zealand journal of psychiatry [Internet]. 2009; 43(3):[201‐7 pp.]. Available from: https://www.cochranelibrary.com/central/doi/10.1002/central/CN-00688324/full. | |
| 1651 | Rejected by TIAB Screening | Ng CF. Training in percutaneous nephrolithotomy: The learning curve and options. Arab Journal of Urology. 2014;12(1):54-7. | |
| 1652 | Rejected by TIAB Screening | Ng SL, Bartlett D, Lucy SD. The education and socialization of audiology students and novices. Seminars in Hearing. 2012;33(2):177-95. | |
| 1653 | Not Target Group | Nguyen D, Dooley-Hash S, Rooney D. A novel mixed modality approach in self-directed procedural simulation. Academic Emergency Medicine. 2017;24 (Supplement 1):S104. | |
| 1654 | Rejected by TIAB Screening | Nguyen K, Devor M. Raging over nonadherence: Learning disability in an elderly patient. Journal of General Internal Medicine. 2010;25:S542-S3. | |
| 1655 | Not Target Group | Nguyen XV, Adams SJ, Hobbs SK, Ganeshan D, Wasnik AP. Radiologist as Lifelong Learner: Strategies for Ongoing Education. Academic Radiology. 2019;26(8):1120-6. | |
| 1656 | Rejected by TIAB Screening | Ngwa W, Irabor O, Swanson W, Bhagwat M. A new platform for collaborative global radiation oncology education. Medical Physics. 2018;45 (6):e423-e4. | |
| 1657 | Rejected by TIAB Screening | Nichols LO, DeFriese AM, Malone CC. Team process. Team performance in health care: Assessment and development. New York, NY: Kluwer Academic/Plenum Publishers; US; 2002. p. 71-88. | |
| 1658 | Rejected by TIAB Screening | Nicholson S, Osonnaya C, Y HC, Savage W, Hennessy E, Collinson S. Designing a community-based fourth-year obstetrics and gynaecology module: An example of innovative curriculum development. Medical Education. 2001;35(4):398-403. | |
| 1659 | Rejected by TIAB Screening | Nickel F, Jede F, Minassian A, Gondan M, Hendrie J, Gehrig T, et al. One or two trainees per workplace in a structured multimodality training curriculum for laparoscopic surgery? Study protocol for a randomized controlled trial - DRKS00004675. Trials [Internet]. 2014; 15:[137 p.]. Available from: https://www.cochranelibrary.com/central/doi/10.1002/central/CN-01117028/full. | |
| 1660 | Outside SDL | Niemi-Murola L. [Empathy is lifelong learning]. Duodecim. 2015;131(1):62-7. | |
| 1661 | Rejected by TIAB Screening | Nilsen LL. Workplace Learning among General Practitioners and Specialists: The Use of Videoconferencing as a Tool. Journal of Workplace Learning. 2011;23(8):501-17. | |
| 1662 | Rejected by TIAB Screening | Nixon H, Stariha J, Farrer J, Wong C, Maisels M, Toledo P. Resident Competency and Proficiency in Combined Spinal-Epidural Catheter Placement Is Improved Using a Computer-Enhanced Visual Learning Program: a Randomized Controlled Trial. Anesthesia and analgesia [Internet]. 2019; 128(5):[999‐1004 pp.]. Available from: https://www.cochranelibrary.com/central/doi/10.1002/central/CN-01940950/full. | |
| 1663 | Rejected by TIAB Screening | Nixon NA, Ko YJ, Lim HJ, Elser C, Tam VC. Oncology education for internal medicine residents: The value of participating in a medical oncology rotation. Journal of Clinical Oncology Conference. 2016;34(Supplement 15). | |
| 1664 | Rejected by TIAB Screening | Nixon NA, Lim H, Elser C, Ko YJ, Lee-Ying R, Tam VC. Oncology education for Canadian internal medicine residents: the value of participating in a medical oncology elective rotation. Current Oncology. 2018;25(3):213-8. | |
| 1665 | Not Target Group | No authorship i. Lifelong learning for professional oncology nurses. Oncology Nursing Forum. 2012;39(2):127. | |
| 1666 | Rejected by TIAB Screening | Noble C, Billett S. Learning to prescribe through co-working: junior doctors, pharmacists and consultants. Medical Education. 2017;51(4):442-51. | |
| 1667 | Rejected by TIAB Screening | Noble C, Billett S, Armit L, Collier L, Hilder J, Sly C, et al. "It&apos;s Yours to Take": Generating Learner Feedback Literacy in the Workplace. Advances in Health Sciences Education. 2020;25(1):55-74. | |
| 1668 | Rejected by TIAB Screening | Noble C, Brazil V, Teasdale T, Forbes M, Billett S. Developing junior doctors' prescribing practices through collaborative practice: Sustaining and transforming the practice of communities. Journal of Interprofessional Care. 2017;31(2):263-72. | |
| 1669 | Rejected by TIAB Screening | Noble C, Hassell K. Informal learning in the workplace: What are the environmental barriers for junior hospital pharmacists? International Journal of Pharmacy Practice. 2008;16(4):257-63. | |
| 1670 | Not Target Group | Noh GO, Kim DH. Effectiveness of a self-directed learning program using blended coaching among nursing students in clinical practice: a quasi-experimental research design. BMC Medical Education. 2019;19(1):225. | |
| 1671 | Not Target Group | Nordberg A, Carreiro S, Chai PR, Carey J, Bird S. Bringing emergency wellness and encouraging lifelong learning (be well)-an innovative wellness elective model. Academic Emergency Medicine. 2017;24 (Supplement 1):S285. | |
| 1672 | Rejected by TIAB Screening | Norouzi H, Tohidi S, Moonaghi HK, Vashini HB. The comporison of the effects of the lecture and self-learning module on nurses' teaching-competency to cardiac patient education cardiac patient education. [Persian]. Journal of Mazandaran University of Medical Sciences. 2014;24(SUPPL. 1):196-202. | |
| 1673 | Rejected by TIAB Screening | Nothnagle M, Anandarajah G, Goldman RE, Reis S. Struggling to be self-directed: residents' paradoxical beliefs about learning. Academic Medicine. 2011;86(12):1539-44. | |
| 1674 | Not Target Group | Novak MK, Palladino C, Ange B, Richardson D. Measuring health professions students' orientation toward lifelong learning. Journal of Allied Health. 2014;43(3):146-9. | |
| 1675 | Rejected by TIAB Screening | Nshaho J. Innovative strategies in teaching of biomedical sciences to health professionals. Nigerian Journal of Physiological Sciences. 2005;20(1-2):8-10. | |
| 1676 | Rejected by TIAB Screening | Numminen OH, Leino-Kilpi H, Arend Avd, Katajisto J. Nurse educators' teaching of codes of ethics. Nurse Education Today. 2010;30(2):124-31. | |
| 1677 | Rejected by TIAB Screening | Nussbaum GM, Ault SP. Evergreen staff: building a tree of knowledge for continuous learning. Journal of Healthcare Information Management. 2000;14(3):83-96. | |
| 1678 | Rejected by TIAB Screening | Nussbaum MS. Invited lecture: American Board of Surgery Maintenance of Certification explained. American Journal of Surgery. 2008;195(3):284-7. | |
| 1679 | Rejected by TIAB Screening | O. Brien B Keene TS, R. L. Team huddles as workplace learning opportunities: An observational study. Journal of General Internal Medicine. 2014;29:S218-S9. | |
| 1680 | Rejected by TIAB Screening | O'Brien B, Teherani A. Using workplace learning to improve patient care. Academic Medicine. 2011;86(11):e12. | |
| 1681 | Rejected by TIAB Screening | O'Brien K, Moore A, Hartley P, Dawson D. Lessons about work readiness from final year paramedic students in an Australian university. Australasian Journal of Paramedicine. 2013;10(4). | |
| 1682 | Rejected by TIAB Screening | O'Brien R, Woodbridge S, Hammond A, Adkin J, Culley J. The Development and Evaluation of a Vocational Rehabilitation Training Programme for Rheumatology Occupational Therapists. Musculoskeletal Care. 2013;11(2):99-105. | |
| 1683 | Rejected by TIAB Screening | O'Connor E, Moore M, Cullen W, Cantillon P. A qualitative study of undergraduate clerkships in the intensive care unit: It's a brand new world. Perspectives on medical education. 2017;6(3):173-81. | |
| 1684 | Rejected by TIAB Screening | O'Connor M. Beyond the classroom: nurse leader preparation and practices. Nursing Administration Quarterly. 2011;35(4):333-7. | |
| 1685 | Rejected by TIAB Screening | O'Connor N, Kotze B. 'Learning organizations': A clinician's primer. Australasian Psychiatry. 2008;16(3):173-8. | |
| 1686 | Outside SDL | O'Dowd A, Norris R. Paying the price for lifelong learning. Nursing Times. 2003;99(15):10-1. | |
| 1687 | Rejected by TIAB Screening | O'Keefe M, Roberton DM. Medical student education in paediatrics and child health: Where are we going? Journal of Paediatrics and Child Health. 1998;34(3):211-2. | |
| 1688 | Not Target Group | O'Kell SP. A study of the relationships between learning style, readiness for self-directed learning and teaching preference of learner nurses in one health district. Nurse Education Today. 1988;8(4):197-204. | |
| 1689 | Rejected by TIAB Screening | O'Neill B, et al. World Perspective Case Descriptions on Educational Programs for Adults: Australia. Reports - Descriptive. 1989. | |
| 1690 | Rejected by TIAB Screening | O'Rourke KS, Bolster MB, Criscione-Schreiber LG, Jonas BL. An instrument to provide documentation (iPROD) of practice-based learning and improvement (PBLI): A pilot trial conducted by the carolinas fellows collaborative. Arthritis and Rheumatism. 2009;10):1224. | |
| 1691 | Outside SDL | O'Shea E. Self-directed learning in nurse education: a review of the literature. Journal of Advanced Nursing. 2003;43(1):62-70. | |
| 1692 | Rejected by TIAB Screening | Oaklief CR, Oaklief MM. Participation in Kansas Noncredit Adult Education. A Survey of Seven Participant Groups. Final Report. Reports - Research. Kansas State Univ., Manhattan. Dept. of Adult and Occupational Education.; 1983. | |
| 1693 | Rejected by TIAB Screening | Obura T, Brant WE, Miller F, Parboosingh IJ. Participating in a Community of Learners enhances resident perceptions of learning in an e-mentoring program: proof of concept. BMC Medical Education. 2011;11:3. | |
| 1694 | Rejected by TIAB Screening | Ockerby CM, Newton JM, Cross WM, Jolly BC. A Learning Partnership: Exploring Preceptorship through Interviews with Registered and Novice Nurses. Mentoring and Tutoring: Partnership in Learning. 2009;17(4):369-85. | |
| 1695 | Not Target Group | Oddi LF. Comparison of self-directed learning scores among graduate students in nursing, adult education, and law. Journal of Continuing Education in Nursing. 1988;19(4):178-81. | |
| 1696 | Rejected by TIAB Screening | Oddi LF, et al. Construct Validation of the Oddi Continuing Learning Inventory. Adult Education Quarterly. 1990;40(3):139-45. | |
| 1697 | Rejected by TIAB Screening | Oh JW, Huh B, Kim MR. Effect of learning contracts in clinical pediatric nursing education on students' outcomes: A research article. Nurse Education Today. 2019;83:104191. | |
| 1698 | Not Target Group | Ohliger J. Is lifelong adult education a guarantee of permanent inadequacy? [References]. Challenging the professionalization of adult education: John Ohliger and contradictions in modern practice. San Francisco, CA: Jossey-Bass; US; 2009. p. 47-63. | |
| 1699 | Rejected by TIAB Screening | Ohno R. The undergraduate medical education in neurology and the active learning in small groups. [Japanese]. Clinical Neurology. 1996;36(12):1341-2. | |
| 1700 | Rejected by TIAB Screening | Ohta R, Son D. What do medical residents learn on a rural Japanese island? Journal of Rural Medicine. 2018;13(1):11-7. | |
| 1701 | Rejected by TIAB Screening | Okisaka S. Changing aspects in continuing medical education. [Japanese]. Nippon Ganka Gakkai zasshi. 2007;111(2):83-8. | |
| 1702 | Rejected by TIAB Screening | Okuda Y, Bryson EO, DeMaria Jr S, Jacobson L, Quinones J, Shen B, et al. The utility of simulation in medical education: What is the evidence? Mount Sinai Journal of Medicine. 2009;76(4):330-43. | |
| 1703 | Not Target Group | Oliveira AL, Silva JT, Lima MP. Aging and health: Self-efficacy for Self-direction in Health Scale. Revista de Saude Publica. 2016;50(0):04. | |
| 1704 | Rejected by TIAB Screening | Olmos-Vega FM, Dolmans D, Guzman-Quintero C, Echeverri-Rodriguez C, Teunnissen PW, Stalmeijer RE. Disentangling residents' engagement with communities of clinical practice in the workplace. Advances in Health Sciences Education. 2019;24(3):459-75. | |
| 1705 | Rejected by TIAB Screening | Olmos-Vega FM, Dolmans D, Vargas-Castro N, Stalmeijer RE. Dealing with the tension: how residents seek autonomy and participation in the workplace. Medical Education. 2017;51(7):699-707. | |
| 1706 | Rejected by TIAB Screening | Olmos-Vega FM, Dolmans DH, Guzman-Quintero C, Stalmeijer RE, Teunissen PW. Unravelling residents' and supervisors' workplace interactions: an intersubjectivity study. Medical Education. 2018;52(7):725-35. | |
| 1707 | Rejected by TIAB Screening | Olsen TH, Glad T, Filstad C. Learning to Learn Differently. Journal of Workplace Learning. 2018;30(1):18-31. | |
| 1708 | Rejected by TIAB Screening | Olson TH. Education for Educators in Medical Technology 1977. | |
| 1709 | Rejected by TIAB Screening | Omer TY, Suliman WA, Thomas L, Joseph J. Perception of nursing students to two models of preceptorship in clinical training. Nurse Education in Practice. 2013;13(3):155-60. | |
| 1710 | Rejected by TIAB Screening | Omrani S, Fardanesh H, Hemmati N, Hemmati N. Exploring an Appropriate Instructional Design Model for Continuing Medical Education. Turkish Online Journal of Distance Education. 2012;13(3):347-61. | |
| 1711 | Formal Teaching | Oncology Nursing S. Lifelong learning for professional oncology nurses. Oncology Nursing Forum. 2012;39(2):127. | |
| 1712 | Rejected by TIAB Screening | Onishi M, Sasaki M, Nagata A, Kanda K. Development of nurses with specialties: the nurse administrators' perspective. Journal of Nursing Management. 2008;16(7):795-803. | |
| 1713 | Rejected by TIAB Screening | Onrubia X, Traore H. Improving performance of rural birth attendants by means of a mobile simulation training unit. International Journal of Gynecology and Obstetrics. 2018;143 (Supplement 3):483-4. | |
| 1714 | Rejected by TIAB Screening | Ooi SY, Karamatic R, Kendall BJ, Hewett DG. Enhancing gastroenterology training in Queensland: A statewide gastroenterology education program. Journal of Gastroenterology and Hepatology. 2010;25:A43. | |
| 1715 | Not Target Group | Opipari VP, Daniels SR, Wilmott RW, Jacobs RF. Association of Medical School Pediatric Department Chairs Principles of Lifelong Learning in Pediatric Medicine. JAMA Pediatrics. 2016;170(11):1087-92. | |
[truncated: 301,631 more chars]
