# Supplementary material for: Self-directed learning in health professions: A mixed-methods systematic review of the literature
Source: PLoS One. 2025 May 2;20(5):e0320530. doi: 10.1371/journal.pone.0320530 (PMC12047769; doi:10.1371/journal.pone.0320530)
Supplement: S2 File — (DOCX) [file pone.0320530.s009.docx]

SX Appendix: Risk of Bias: ROBINS-1

|  | Author/Year/ Country | Bias due to confounding | Bias in selection of participants into the study | Bias in classification of interventions | Bias due to deviations from intended interventions | Bias due to missing data | Bias in measurement of outcomes | Bias in selection of the reported result | Overall risk of bias |
| --- | --- | --- | --- | --- | --- | --- | --- | --- | --- |
| 1 | Acar, 2023[^65^](#_ENREF_65), Turkey | Serious | Moderate | N/A | Low | Low | Moderate | Low | Serious |
| 2 | Allen, 2024[^66^](#_ENREF_66), USA | Serious | Moderate | N/A | N/A | Low | Moderate | Moderate | Serious |
| 3 | Andersen, 2022[^51^](#_ENREF_51), Denmark | Serious | Moderate | N/A | N/A | Low | Moderate | Low | Serious |
| 4 | Antofie, 2023^[67](#_ENREF_67)^, Romania | Serious | Moderate | N/A | N/A | Low | Moderate | Moderate | Serious |
| 5 | Berg Jansson, 2022[^68^](#_ENREF_68), Sweden | Serious | Moderate | N/A | N/A | Low | Moderate | Moderate | Serious |
| 6 | Bing-Jonsson, 2023[^69^](#_ENREF_69), Norway | Serious | Moderate | N/A | N/A | Moderate | Moderate | Moderate | Serious |
| 7 | Bolton, 2022^[70](#_ENREF_70)^, Paraguay | Serious | Moderate | N/A | N/A | Moderate | Moderate | Low | Serious |
| 8 | Brydges 2012[^28^](#_ENREF_28), Canada | Serious | Moderate | N/A | N/A | Low | Moderate | Moderate | Serious |
| 9 | Chakkaravarthy, 2020[^52^](#_ENREF_52), Brunei | Serious | Moderate | N/A | N/A | Moderate | Moderate | Low | Serious |
| 10 | Claponea, 2023[^71^](#_ENREF_71), Romania | Serious | Moderate | N/A | N/A | Moderate | Moderate | Moderate | Serious |
| 11 | Claret, 2020[^53^](#_ENREF_53), Spain | Serious | Moderate | N/A | N/A | Moderate | Moderate | Moderate | Serious |
| 12 | Clouder, 2022[^72^](#_ENREF_72), UK | Serious | Moderate | N/A | N/A | Moderate | Moderate | Low | Serious |
| 13 | Cuyvers, 2024[^73^](#_ENREF_73), the Netherlands | Serious | Moderate | N/A | N/A | Moderate | Moderate | Moderate | Serious |
| 14 | Fahlman, 2013[^54^](#_ENREF_54), Canada | Serious | Moderate | N/A | N/A | Moderate | Moderate | Moderate | Serious |
| 15 | Gathu, 2022[^74^](#_ENREF_74), Kenya | Serious | Moderate | N/A | N/A | Moderate | Moderate | Moderate | Serious |
| 16 | Ghiyasvandian 2015 [^55^](#_ENREF_55), Iran | Serious | Moderate | N/A | N/A | Moderate | Moderate | Low | Serious |
| 17 | Hill, 2010[^56^](#_ENREF_56), USA | Serious | Moderate | N/A | N/A | Moderate | Moderate | Low | Serious |
| 18 | Kim, 2024[^75^](#_ENREF_75), South Korea | Serious | Moderate | N/A | N/A | Moderate | Moderate | Low | Serious |
| 19 | Kyndt, 2016[^57^](#_ENREF_57), Belgium | Serious | Moderate | N/A | N/A | Moderate | Moderate | Low | Serious |
| 20 | Lee, 2017[^58^](#_ENREF_58), South Korea (Dissertation) | Serious | Moderate | N/A | N/A | Moderate | Moderate | Moderate | Serious |
| 21 | Lim, 2021[^59^](#_ENREF_59), USA | Serious | Moderate | N/A | N/A | Moderate | Moderate | Low | Serious |
| 22 | Lin, 2023[^76^](#_ENREF_76), Taiwan | Serious | Moderate | N/A | N/A | Moderate | Moderate | Moderate | Serious |
| 23 | Liu, 2024[^77^](#_ENREF_77), China | Serious | Moderate | N/A | N/A | Moderate | Moderate | Moderate | Serious |
| 24 | Malekian, 2015[^60^](#_ENREF_60), Iran | Moderate | Low | Low | Low | Moderate | Moderate | Moderate | Moderate |
| 25 | Mamiya, 2023[^78^](#_ENREF_78), Japan | Moderate | Moderate | Low | Low | Moderate | Moderate | Moderate | Moderate |
| 26 | Papanagnou, 2022[^79^](#_ENREF_79), USA | N/A | N/A | N/A | N/A | N/A | N/A | N/A | N/A |
| 27 | Sockalingam, 2022[^80^](#_ENREF_80), Canada | Moderate | Moderate | Low | Low | Moderate | Moderate | Moderate | Moderate |
| 28 | Strachan, 2015[^61^](#_ENREF_61), UK (Editorial) | N/A | N/A | N/A | N/A | N/A | N/A | N/A | N/A |
| 29 | Sturesson-Sabel, 2021[^62^](#_ENREF_62), Sweden | N/A | N/A | N/A | N/A | N/A | N/A | N/A | N/A |
| 30 | Taylor, 2015[^63^](#_ENREF_63), USA | Moderate | Moderate | Low | Low | Moderate | Moderate | Moderate | Moderate |
| 31 | Yang, 2023[^81^](#_ENREF_81), China | Moderate | Moderate | Low | Low | Moderate | Moderate | Moderate | Moderate |
| 32 | Yao, 2023[^82^](#_ENREF_82), China | Serious | Moderate | N/A | Low | Low | Moderate | Low | Serious |
| 33 | Yun, 2019[^64^](#_ENREF_64), South Korea | Moderate | Moderate | Low | Low | Moderate | Moderate | Moderate | Moderate |
| 34 | Wang, 2024[^83^](#_ENREF_83), China | Serious | Moderate | N/A | N/A | Low | Moderate | Moderate | Serious |
