# Supplementary material for: Self-directed learning in health professions: A mixed-methods systematic review of the literature
Source: PLoS One. 2025 May 2;20(5):e0320530. doi: 10.1371/journal.pone.0320530 (PMC12047769; doi:10.1371/journal.pone.0320530)
Supplement: Attachment — Submitted filename: SDL_Reply to Reviewers Comments.docx [file pone.0320530.s011.docx]

Reply to Reviewers’ Comments

Editor:

**Comment 1:** Please ensure that your manuscript meets PLOS ONE's style requirements, including those for file naming. The PLOS ONE style templates can be found at

<https://journals.plos.org/plosone/s/file?id=wjVg/PLOSOne_formatting_sample_main_body.pdf> and

<https://journals.plos.org/plosone/s/file?id=ba62/PLOSOne_formatting_sample_title_authors_affiliations.pdf>.

**Reply:** Thank you for the reminder regarding the manuscript formatting. We have reviewed the PLOS ONE style requirements and ensured that our manuscript now adheres to them, including the file naming conventions and formatting guidelines outlined in the provided templates.

Specifically, we have:

- Applied the correct file naming as per PLOS ONE requirements.
- Updated the manuscript structure, including title page, author affiliations, and headings, to match the formatting samples.
- Ensured the figures, tables, and supplementary files follow PLOS ONE guidelines for clarity and consistency.

We appreciate your guidance and hope the updated manuscript now complies with all formatting requirements.

**Comment 2.** Thank you for stating the following in the Competing Interests section:

[I have read the journal's policy and the authors of this manuscript have the following competing interests: JBE is an associate editor for BMC Medical Education and has received travel expenses from Medtronic for the Save the Brain Initiative training. ].

Please confirm that this does not alter your adherence to all PLOS ONE policies on sharing data and materials, by including the following statement: ""This does not alter our adherence to  PLOS ONE policies on sharing data and materials.” (as detailed online in our guide for authors <http://journals.plos.org/plosone/s/competing-interests>).  If there are restrictions on sharing of data and/or materials, please state these. Please note that we cannot proceed with consideration of your article until this information has been declared.

Please include your updated Competing Interests statement in your cover letter; we will change the online submission form on your behalf.

**Reply:** Thank you for your feedback regarding the Competing Interests section. We confirm that the competing interests mentioned in our initial statement do not alter our adherence to all PLOS ONE policies on sharing data and materials.

Here is the updated Competing Interests statement:

“JBE is an associate editor for BMC Medical Education and has received travel expenses from Medtronic for the Save the Brain Initiative training. This does not alter our adherence to  PLOS ONE policies on sharing data and materials. (…)”

We hope this satisfies the journal's requirements, and we appreciate your attention to this matter.

**Comment 3:** Please include your tables as part of your main manuscript and remove the individual files. Please note that supplementary tables (should remain/ be uploaded) as separate ""supporting information"" files".

**Reply:** Thank you. We have done the necessary changes accordingly.

**Comment 4:** Please review your reference list to ensure that it is complete and correct. If you have cited papers that have been retracted, please include the rationale for doing so in the manuscript text, or remove these references and replace them with relevant current references. Any changes to the reference list should be mentioned in the rebuttal letter that accompanies your revised manuscript. If you need to cite a retracted article, indicate the article’s retracted status in the References list and also include a citation and full reference for the retraction notice.

**Reply:** Thank you for your thoughtful feedback. We have carefully reviewed our reference list to ensure it is complete and correct, and we confirm that, based on our verification through databases of **Retraction Watch** and **PubMed**, none of the cited papers in the current version of the manuscript have been retracted.

However, we acknowledge that retraction databases are continuously updated. If you are aware of any paper we have cited that is on a retraction list, please kindly inform us, and we will promptly address the issue.

**Comment 5:** The manuscript titled "Self-Directed Learning in Health Professions: A Mixed-Methods Systematic Review of the Literature" presents a well-organized and clearly written study that addresses an important topic in health education.

**Reply:** Thank you for your positive feedback on our manuscript titled "Self-Directed Learning in Health Professions: A Mixed-Methods Systematic Review of the Literature." We are delighted that you found the study well-organized and clearly written, and that it addresses an important topic in health education. We appreciate your support and look forward to furthering this valuable conversation in the field.

**Comment 6:** However, both reviewers have identified some areas that require further refinement to strengthen the overall quality of the paper.Based on the reviewers' comments, the following areas should be addressed:
Meta-Analysis Methodology: Provide more details on the meta-analysis methodology used, including the inclusion and exclusion criteria, data extraction process, and statistical analysis techniques.

**Reply:** Thank you for your constructive feedback. We appreciate the opportunity to further enhance our manuscript by addressing the areas you've highlighted. We agree that additional details will help clarify our approach regarding the meta-analysis methodology. We revised the manuscript to include more comprehensive information on the inclusion and exclusion criteria, the data extraction process, and the statistical analysis techniques employed. Specifically, we elaborated on how studies were selected, how data were extracted, and the specific statistical models attempted in the meta-analysis.

One can now read: “We planned a meta-analysis to examine specific outcomes from the quantitative studies involving quantitative SDL outcomes. The meta-analysis was planned with RevMan software v.5.4.20, applying a random-effects model to account for the expected heterogeneity across the included studies. The data selected included means and standard deviation (SD), with the effect size estimated by the mean difference (MD) and reported with 95% confidence intervals (CI). Studies that did not report group means or MD were excluded from the meta-analysis. Where meta-analyses could not be conducted, we summarized and described the data in the text.”

**Comment 7:** Meta-Analysis Results: Clearly present the results of the meta-analysis, including the overall effect size, heterogeneity, and potential moderators.

**Reply**: We appreciate your valuable input on the meta-analysis reporting. We acknowledge the importance of using meta-analysis appropriately, and while it is a powerful tool for deriving meaningful conclusions, it can be problematic when high variability exists among studies. In our case, we found that a meta-analysis was not feasible due to the high variability of scales used across the included studies. A common criticism of meta-analyses is the risk of ‘combining apples with oranges,’ especially when studies are clinically diverse. In our review, the diversity in outcome measures and assessment tools used for self-directed learning in health professions rendered a meta-analysis potentially misleading. Genuine differences in effects might have been obscured, making it nonsensical to combine all studies in a single analysis.

Additionally, some studies included varying comparisons of SDL interventions, making it challenging to combine the outcomes in a meaningful way. As a result, we chose to summarize and describe the findings narratively rather than risk compounding errors through a meta-analysis that could produce a misleading or biased result. We clarified these points in the revised manuscript and provided a detailed explanation of the rationale for not conducting a meta-analysis in this instance.

We added to the Results Section: While we initially planned to conduct a meta-analysis to synthesize the quantitative outcomes of SDL interventions, due to the high variability in the scales and outcome measures used across the included studies, a meta-analysis was deemed inappropriate. Combining studies with such diverse methodologies would risk producing misleading results and obscure meaningful differences in effects.

**Comment 8:** Abstract Clarity: Move the statement about the significance of individual motivation, learning environment, technological resources, social interactions, and healthcare professionals' readiness for SDL from the conclusion to the methodology section, merging it with the third sentence.

**Reply:** Thank you. We have changed the sentence accordingly.

**Comment 9:** Section Headings: Add "Methodology" as a main heading above the "study design" sub-heading to improve the paper's structure.

**Reply:** Thank you. We have made the amendments accordingly.

**Comment 10:** Limitations: Include a dedicated section for limitations, discussing any potential biases, methodological shortcomings, or generalizability issues.

Reply: Thank you for your suggestion to include a dedicated limitations section. We agree that addressing potential biases, methodological shortcomings, and generalizability issues is crucial for providing a transparent and balanced interpretation of our findings.

In the revised manuscript, we expanded the limitations section to further discuss the aforementioned aspects.

The updated version reads: “This systematic review has several important limitations. First, while we aimed to explore the application and effectiveness of various SDL models within healthcare organizations, this was only partially achieved. Few studies adopted a work organizational perspective, limiting our ability to fully analyze how organizational psychology impacts SDL strategies for healthcare professionals. The vagueness of the SDL concept also posed challenges throughout the review. This ambiguity was reflected in the low agreement between raters during the initial article screening phase, which led both reviewers to screen all articles to ensure consistency. Our comprehensive search strategy, which included related terms such as “self-regulated learning” and “informal learning,” sometimes made it difficult to determine from titles and abstracts whether SDL was being used as intended. We aimed to separate formal learning from informal SDL, but articles often lacked clarity on this distinction. Despite our efforts, relevant studies may have been missed, and some studies using SDL models or frameworks may not have been explicitly identified, suggesting that the actual count of studies using such models could be higher than reported. In addition, many of the included studies showed variability in design quality, which prevented a meta-analysis, and some were at risk of bias due to inadequate reporting or the absence of control groups. This could have impacted the reliability of the findings, and this potential bias must be considered when interpreting the results. Also, the wide range of outcome measures and assessment tools used across the studies limited our ability to conduct a meaningful meta-analysis. Variations in study designs, SDL formats, and implementation strategies introduced inconsistencies that affected the comparability of results, further complicating data synthesis. Furthermore, given the specific focus on SDL in health professions education, the findings may not be fully generalizable to other educational fields or professional settings. Finally, the studies included in this review were conducted in diverse geographical regions, which may limit the applicability of the findings to different healthcare systems or educational environments.”

----------------------------------------------------------------------------------------------------------------
Reviewers' comments:

**Comments to the Author**

**Comment 1:** Is the manuscript technically sound, and do the data support the conclusions?

Reviewer #1: Yes

Reviewer #2: Yes

**Reply:** We sincerely thank both reviewers for their positive feedback and confirmation that the manuscript is technically sound and that the data support the conclusions. We appreciate your time and effort in reviewing our work and are glad that the technical aspects and conclusions align with your expectations.

**Comment 2:** Has the statistical analysis been performed appropriately and rigorously?

Reviewer #1: Yes

Reviewer #2: Yes

**Reply:** We appreciate both reviewers' acknowledgment that the statistical analysis has been performed appropriately and rigorously. Thank you for your careful evaluation of our methodology and analysis. We are pleased that our approach meets the necessary standards, and we remain committed to maintaining rigor in the presentation and interpretation of our findings.

**Comment 3:** Have the authors made all data underlying the findings in their manuscript fully available?

Reviewer #1: Yes

Reviewer #2: Yes

**Reply:** Thank you for your comment regarding data availability. We confirm that all data underlying the findings in our manuscript have been made fully available. The data supporting our analysis are provided in the supplementary materials and are accessible as per the journal’s data-sharing policies.

**Comment 4:** Is the manuscript presented in an intelligible fashion and written in standard English?

Reviewer #1: Yes

Reviewer #2: Yes

**Reply:** We are grateful to both reviewers for their positive feedback regarding the clarity and language of the manuscript. We are pleased that the presentation is intelligible and meets the standards of written English.

**Comment 5:** Reviewer #1: Wverything is good but need improve for several aspects such as methodology and discussion. Detailing aspect of theory anf empirical data should have point intersection for elaborating the phebomena and devine the purposes of the study

**Reply:** Thank you for your constructive feedback. We appreciate your recognition of the strengths in our manuscript and the suggestion to further improve specific areas.

We added the following sentence to the methodology: “The JBI methodology for systematic reviews, which integrates both qualitative and quantitative evidence. Grounded in Knowles’ adult learning theory[6] and Bandura’s self-efficacy theory[18], our empirical approach aligned with these frameworks by selecting outcome measures such as learner engagement and self-efficacy. The JBI methodology allowed us to rigorously synthesize diverse evidence and account for variations in SDL interventions, ensuring robust and theory-driven conclusions.”

We also expanded the discussion to address the reviewer’s comments**: “**The results demonstrated a significant improvement in self-efficacy scores, supporting Knowles’ theory of adult learning, which posits that autonomy leads to enhanced learner confidence. Interestingly, engagement scores showed only marginal improvement, suggesting that other factors, perhaps not covered by the theory, may play a role in engagement during SDL interventions. These findings highlight the need for further refinement of the theoretical model to account for additional motivational factors.”

**Comment 6:** Reviewer #2: The manuscript is well written with the attention to detail. The themes were well itemized and clearly presented.
However, these minor issues need to be addressed.

More details should be provided on the meta-analysis of the data (if conducted) in the methodology and the outputs in the results section. i it was not done please indicate that and state how you handled the quantitative data.

**Reply:** Thank you. We added a section on statistics, that includes the description of the attempt of a meta-analysis and in the results the reasons why it was not feasible. For more details please refer to Comments 6 and 7 from the Editor.

**Comment 7:** In the abstract, the following statement - "The findings emphasize the significance of individual motivation, the learning environment, technological resources, social interactions, and healthcare professionals' readiness for SDL." should be moved from conclusion and be merged with the third sentence in the methodology (in the abstract).

**Reply:** Thank you. We have changed the sentence accordingly.

**Comment 8:** Add "Methodology" as the main heading above "study design" sub-heading (lines 99 to 211 are all under methodology).

**Reply:** Thank you. We have made the amendments accordingly.

**Comment 9:** Limitation should be a section with a heading (between lines 573 and 574.

**Reply:** Thank you. We have made the amendments accordingly.
