# Supplementary material for: Self-directed learning in health professions: A mixed-methods systematic review of the literature
Source: PLoS One. 2025 May 2;20(5):e0320530. doi: 10.1371/journal.pone.0320530 (PMC12047769; doi:10.1371/journal.pone.0320530)
Supplement: Attachment — Submitted filename: SDL_Reply to reviewers_R2.docx [file pone.0320530.s012.docx]

Reply to Reviewers’ Comments

Self-Directed Learning in Health Professions: a Mixed-methods Systematic Review of the Literature

**# Journal Requirements:**

**Comment 1:** Please review your reference list to ensure that it is complete and correct. If you have cited papers that have been retracted, please include the rationale for doing so in the manuscript text, or remove these references and replace them with relevant current references. Any changes to the reference list should be mentioned in the rebuttal letter that accompanies your revised manuscript. If you need to cite a retracted article, indicate the article’s retracted status in the References list and also include a citation and full reference for the retraction notice.

**Reply:** Thank you for your comment. We have carefully reviewed the reference list in detail and did not identify any papers that have so far been retracted. However, if the publisher has access to additional information indicating that any of the cited references have been retracted, we would appreciate your notification, and we will promptly take appropriate action to either replace or justify the inclusion of such references.

—-------------------------------------------------------------------------------------------------------------

**# Additional Editor Comments:**

**Comment 1:** The authors have made substantial improvements to the manuscript, addressing most of the reviewers' comments with detailed revisions. The paper now adheres more closely to PLOS ONE's formatting and content standards. While there are minor areas that could be further improved, the overall quality of the revision suggests that the manuscript is nearing readiness for acceptance with minor revisions. These revisions could focus on enhancing the clarity of certain sections and expanding the discussion to ensure broader applicability

**Reply:** Thank you for your positive feedback and for acknowledging the improvements made to the manuscript. We appreciate your suggestion to further enhance the clarity of certain sections and expand the discussion to ensure broader applicability. Please see comments below for further changes.

**Comment 2:** First, while the authors provided a clear rationale for not conducting a meta-analysis due to high variability, the decision not to combine the data might limit the quantitative strength of the review. A more robust discussion around this decision could be helpful in emphasizing the potential impact on the overall findings.

**Reply:** Thank you for this insightful comment. We agree that the decision not to conduct a meta-analysis may influence the perceived quantitative strength of the review. However, after careful consideration, we concluded that the significant heterogeneity across the included studies, in terms of study design, outcome measures, SDL models, and intervention types, would have made it challenging to produce meaningful or valid conclusions through meta-analysis.

To further emphasize this point, we have expanded the discussion in the 'Results” section (Pages 11-12) to better explain the potential impact of this decision. We address how the variability in the studies reflects the diversity of self-directed learning (SDL) models and their application across different healthcare contexts, and how attempting to statistically combine these heterogeneous data could obscure meaningful differences.

One can now read (Pages 11-12): “In this review, we opted for a narrative synthesis rather than a meta-analysis due to the significant heterogeneity observed across the included studies. The variability in study design, population characteristics, intervention types, and outcome measures made it inadequate to aggregate the data, as this would risk the introduction of biases. Studies ranged from qualitative explorations to randomized controlled trials, each employing different methodologies and measuring diverse outcomes related to SDL in healthcare settings. Additionally, the heterogeneity in sample sizes, demographic profiles, and contexts presented a challenge for statistical synthesis. To preserve the integrity of the individual findings and to avoid obscuring the complexity and nuances within the data, we synthesized the results narratively. This approach allowed us to explore trends and gaps in the literature, highlight key themes, and provide a more detailed understanding of SDL models and factors in various healthcare environments, ensuring that the conclusions were both contextually relevant and reflective of the diverse research landscape.(...)

Additionally, we highlight that our decision to perform a narrative synthesis allowed for a more nuanced analysis of qualitative and contextual factors influencing SDL, which would have been challenging to capture through meta-analysis. While this approach may limit the review’s quantitative impact, it strengthens the depth and relevance of the qualitative insights, offering a more comprehensive understanding of SDL in healthcare settings.

We added this to the Limitations section (Pages 35): “The reasons for skipping the meta-analysis were presented above. Although it was not possible, it reflects the broader complexity of SDL in healthcare, where different professional contexts, educational environments, and learner characteristics shape the implementation and effectiveness of SDL models. By opting for a qualitative approach, we could preserve each study's unique insights and offer a more nuanced interpretation of the findings. This allowed us to identify not only common themes and facilitators of SDL but also highlight gaps in the current research, particularly concerning the integration of SDL in different healthcare contexts. While a meta-analysis could have provided stronger quantitative conclusions, our approach ensures that the review captures the richness and diversity of the field, offering a comprehensive and contextually informed perspective on SDL in healthcare.

**Comment 3:** Second, although the discussion addresses most aspects of the review, a deeper exploration of the global applicability of self-directed learning (SDL) across different healthcare systems would add value. Expanding the discussion beyond the current scope, which primarily focuses on existing studies, would be beneficial.

**Reply:** "Thank you for this insightful suggestion. We agree that a broader exploration of the global applicability of self-directed learning (SDL) across various healthcare systems would enhance the value of the discussion. To address this, we have expanded the discussion to include an examination of how SDL can be adapted to different healthcare environments, accounting for variations in healthcare systems, cultural norms, and resource availability. This expanded discussion highlights the versatility of SDL and its potential to support lifelong learning and professional development in both high-income and resource-limited settings. The revised section now emphasizes how differing healthcare structures and policies can influence the implementation and success of SDL interventions.

The text in the revised manuscript now reads (Discussion, Pages 32-33): SDL holds significant potential for application across diverse healthcare systems globally, offering a flexible and adaptable framework for continuous professional development. In high-income countries with well-resourced healthcare systems, SDL aligns demand to infrastructure that supports ongoing education, access to digital tools, and a culture of autonomy in learning[88, 89]. Healthcare professionals in these settings often benefit from institutional support, advanced technology, and structured opportunities for SDL, making integrating SDL into their professional development pathways easier [90]. However, the applicability of SDL extends beyond these contexts, as it can be a critical educational strategy in low- and middle-income countries where resources for formal training may be limited. In such environments, SDL can be a cost-effective and scalable model, allowing healthcare professionals to engage in learning activities tailored to their needs, even without extensive institutional support or formal educational programs[91, 92]. This adaptability highlights SDL’s relevance in fostering lifelong learning and addressing the professional development needs of healthcare workers in diverse global contexts[90].

However, the global applicability of SDL is not without challenges, as it must be supported to suit the unique cultural, structural, and resource constraints of different healthcare systems. In countries with more hierarchical healthcare systems, implementing SDL may require a shift in organizational culture to promote greater autonomy and self-regulation among healthcare professionals[91]. Moreover, cultural norms regarding education and authority can influence how SDL is perceived and adopted. For example, in cultures that strongly emphasise deference to senior healthcare professionals, encouraging junior staff to take ownership of their learning may require specific interventions, such as mentoring programs or peer support networks[93]. Additionally, in resource-constrained settings, the availability of digital tools and reliable internet access may limit the effectiveness of SDL strategies that rely on online learning platforms. In these contexts, the success of SDL may depend on innovative approaches, such as using mobile learning or community-based learning networks[94]. Therefore, while SDL offers a universally applicable model for continuous professional development, its implementation must be carefully tailored to each healthcare system’s socio-cultural and economic realities to ensure its effectiveness and sustainability.

**Comment 4:** Third, some thematic findings related to social interactions and the learning environment could be further clarified. While these factors are discussed, providing more concrete examples of their practical impact on SDL could improve the narrative.

**Reply:** Thank you for this valuable comment. We agree that providing more concrete examples of how social interactions and the learning environment influence self-directed learning (SDL) would strengthen the narrative. In response, we have expanded the discussion to include specific examples that illustrate how peer collaboration, mentoring, and supportive learning environments can facilitate SDL in healthcare settings. For example, peer-to-peer learning in clinical rounds where healthcare professionals share insights on patient cases fosters continuous, self-directed knowledge acquisition. Additionally, mentorship programs that pair junior clinicians with experienced practitioners can guide goal-setting and resource navigation, creating a structured yet self-directed learning pathway. We also reference hospitals that provide e-learning platforms, which integrate SDL into daily practice, allowing staff to access relevant learning modules during work hours. These examples clarify the practical implications of these factors and highlight their importance in creating an optimal environment for SDL.

One can now read (Page 34): Social interactions and the learning environment also play pivotal roles in fostering SDL within healthcare settings. Peer collaboration during clinical rounds, for instance, allows healthcare professionals to exchange knowledge and discuss patient cases in real-time, fostering informal learning outside of formal training programs[89]. Similarly, mentorship programs, where junior staff are paired with experienced clinicians, provide structured support for setting learning goals, accessing resources, and receiving feedback, all of which encourage self-directed learning[95]. Additionally, institutions that offer digital learning platforms and online courses further promote SDL by allowing healthcare professionals to engage in learning at their own pace, during work hours, or in between clinical duties[55]. Hospitals that prioritize open communication, regular feedback, and protected learning time create environments conducive to SDL, making it easier for professionals to pursue their educational goals while maintaining their clinical responsibilities[56]. These practical examples demonstrate how both social interactions and supportive environments can significantly enhance the SDL process in healthcare settings.

**Comment 5:** Fourth, while the authors have embedded tables within the manuscript, ensuring that all supplementary materials are correctly formatted and accessible is crucial for maintaining clarity and alignment with PLOS ONE’s submission guidelines.

**Reply:** Thank you for this important comment. We have carefully reviewed all supplementary materials and tables to ensure they are correctly formatted and aligned with PLOS ONE’s submission guidelines. We have checked that all tables are clearly labeled and properly referenced within the manuscript, and we have verified that supplementary materials are accessible and appropriately formatted to maintain clarity. If there are any additional formatting requirements or issues with accessibility that we may have overlooked, we would appreciate further guidance and will promptly address them.
