# Supplementary material for: Self-directed learning in health professions: A mixed-methods systematic review of the literature
Source: PLoS One. 2025 May 2;20(5):e0320530. doi: 10.1371/journal.pone.0320530 (PMC12047769; doi:10.1371/journal.pone.0320530)
Supplement: Attachment — Submitted filename: Reply to Comments_SDL_R3.docx [file pone.0320530.s013.docx]

Reply to Comments:

Journal Requirements:

**Comment 1:** Please review your reference list to ensure that it is complete and correct. If you have cited papers that have been retracted, please include the rationale for doing so in the manuscript text, or remove these references and replace them with relevant current references. Any changes to the reference list should be mentioned in the rebuttal letter that accompanies your revised manuscript. If you need to cite a retracted article, indicate the article’s retracted status in the References list and also include a citation and full reference for the retraction notice.

**Reply:** Thank you for your feedback. We have carefully reviewed the reference list to ensure it is complete, accurate, and current. After a thorough review, we confirm that no retracted articles are cited in our manuscript. However, if the editorial team is aware of any retractions we may have overlooked, we kindly request that you inform us so we can promptly address them. Additionally, and to maintain the highest standards of accuracy, we cross-checked all references for completeness, correct formatting. We appreciate the opportunity to ensure the quality of our references and remain open to further guidance.

**Additional Editor Comments:**

**Comment 1:** The manuscript is well-structured, adheres to the journal’s guidelines, and presents valuable insights into SDL in healthcare. Acceptance with minor revisions is recommended, focusing on minor language refinement and ensuring overall coherence in the final submission.

**Reply:** We sincerely thank the reviewer for recognizing the manuscript's structure and valuable insights. As part of our commitment to addressing the suggested minor language refinements, we have utilized Grammarly, a professional proofreading tool, to evaluate and improve the text.

The Grammarly report indicated an impressive overall score of 96%, placing the manuscript among the top 4% of all texts analyzed by the tool. It highlighted areas for improvement, such as word choice, sentence clarity, and minor grammatical inconsistencies. Based on this feedback, we carefully reviewed the identified issues and implemented corrections to ensure linguistic precision and coherence throughout the manuscript.

These adjustments have strengthened the manuscript's presentation while maintaining the integrity of its content. We believe the updated version now meets the highest standards of readability and coherence and appreciate your constructive feedback in guiding this process.

**Comment 2:** While revising your submission, please upload your figure files to the Preflight Analysis and Conversion Engine (PACE) digital diagnostic tool, [https://pacev2.apexcovantage.com/](https://track.editorialmanager.com/CL0/https:%2F%2Fpacev2.apexcovantage.com%2F/1/010f0192e2e46e72-7aa55cad-52eb-4bd4-a49b-073b0d869b3d-000000/hnBDlQ3C5JaGDkv0f0r_OW8hP7wKUmIqq7Qbj_lL0xI=183). PACE helps ensure that figures meet PLOS requirements. To use PACE, you must first register as a user. Registration is free. Then, login and navigate to the UPLOAD tab, where you will find detailed instructions on how to use the tool. If you encounter any issues or have any questions when using PACE, please email PLOS at [figures@plos.org](mailto:figures@plos.org). Please note that Supporting Information files do not need this step.

**Reply:** Thank you for your instructions regarding the use of the PACE digital diagnostic tool to ensure our figures meet PLOS requirements. We have successfully registered on the platform, uploaded our figure files, and reviewed the results provided by PACE.

Based on the diagnostic feedback, we made the necessary adjustments to ensure that all figures comply with PLOS guidelines for resolution, format, and other specifications. The updated figures have been incorporated into our revised manuscript.

Please let us know if there are any additional steps required. Thank you for your guidance throughout this process.
