# Supplementary material for: Self-directed learning in health professions: A mixed-methods systematic review of the literature
Source: PLoS One. 2025 May 2;20(5):e0320530. doi: 10.1371/journal.pone.0320530 (PMC12047769; doi:10.1371/journal.pone.0320530)
Supplement: Attachment — Submitted filename: Reply to reviewers comments_R4.docx [file pone.0320530.s014.docx]

PONE-D-24-23600R3

Self-Directed Learning in Health Professions: a Mixed-methods Systematic Review of the Literature

\# Journal Requirements:
**Comment 1:** Please review your reference list to ensure that it is complete and correct. If you have cited papers that have been retracted, please include the rationale for doing so in the manuscript text, or remove these references and replace them with relevant current references. Any changes to the reference list should be mentioned in the rebuttal letter that accompanies your revised manuscript. If you need to cite a retracted article, indicate the article’s retracted status in the References list and also include a citation and full reference for the retraction notice.

**Reply:** Thank you for this important reminder. We have carefully reviewed all references cited in our manuscript to ensure their accuracy, relevance, and current status. Additionally, we have verified that none of the cited articles are included in the retraction list by cross-checking with authoritative databases (e.g., PubMed, Retraction Watch, and CrossMark). To further ensure completeness, we contacted the journal's editorial staff, who confirmed that this message was part of their standard procedure and that none of the cited articles in our reference list have been flagged as retracted. As such, no changes were necessary to the references in our manuscript. We appreciate the opportunity to confirm this and ensure the robustness of our work.

\# Additional Editor Comments:

**Comment 1:** In summary, the manuscript is close to being accepted but still requires minor adjustments to fully meet the journal’s standards. It is recommended that the authors complete these revisions before resubmission.

**Reply:** Thank you for your feedback and for noting that the manuscript is close to acceptance. We are also grateful for the additional reply following our personal contact with the editor, which provided further clarity on the required minor adjustments.

We have carefully reviewed the manuscript and made the necessary revisions to ensure it fully meets the journal’s standards. We trust that these updates address all outstanding points, and we appreciate the opportunity to submit the revised version for your consideration.

**Comment 2:** Inclusion of more specific cases related to key conclusions and additional detailed connections between theoretical models and practical applications

**Reply:** Thank you for this insightful suggestion. We have revised the manuscript to include more specific cases to support key conclusions and enhance the connection between theoretical models and practical applications. These revisions include, in the discussion part, page 32, line 580 ff:

***Specific Cases Related to Key Conclusions:***

- During the COVID-19 pandemic, mobile devices and online platforms became essential tools for informal learning. Healthcare professionals widely utilized mobile applications to access real-time clinical guidelines, collaborate with peers, and navigate the rapidly changing healthcare landscape. This aligns with the findings of Fahlman (2013), which emphasize the role of mobile devices in promoting self-directed learning (SDL).
- Mentorship programs were successfully implemented in hospitals, pairing junior staff with experienced clinicians. These programs facilitated goal-setting, access to resources, and constructive feedback, particularly in high-pressure situations. Papanagnou et al. highlighted how mentorship strengthens SDL competencies and fosters professional development in challenging clinical contexts.

***Detailed Connections Between Theoretical Models and Practical Applications:***

- Knowles’ Adult Learning Theory: Practical applications of this theory are evident in the adoption of digital tools, such as e-learning platforms, that foster autonomy and allow healthcare professionals to learn at their own pace. These tools empower learners by providing flexibility and control over their educational journeys, consistent with Knowles’ principles of self-direction.
- Bandura’s Self-Efficacy Theory: Mentorship programs serve as practical extensions of this theory by enhancing self-efficacy. Structured feedback and goal-setting provided through these programs enable junior clinicians to build confidence in their clinical abilities and their capacity to direct their learning effectively.
- Theory of Self-Directed Learning Processes (SDLPs): The dimensions of personal, process, and contextual factors highlighted in the SDLP framework are demonstrated in interventions such as peer collaboration, mentorship programs, and organizational support. These initiatives exemplify how tailored interventions address the interplay of these dimensions to enhance SDL in diverse healthcare environments.

These additions have been incorporated into the revised Discussion section, where we have expanded on the theoretical implications of our findings and their practical applications within healthcare organizations. We believe these revisions provide concrete examples and further clarify the translation of theory into practice, strengthening the manuscript overall.

**Comment 3:** Conduct a final, comprehensive proofread of the manuscript to ensure all grammatical, typographical, and formatting issues are addressed.

**Reply:** Thank you for emphasizing the importance of ensuring grammatical, typographical, and formatting accuracy. To address this, we have sent the manuscript for professional proofreading to ensure that all grammatical, typographical, and formatting issues are addressed comprehensively. The revised version reflects these corrections, and we are confident that the manuscript now meets the highest standards of clarity and consistency. Thank you for your guidance and support in improving the quality of our submission.

**Comment 4:** While revising your submission, please upload your figure files to the Preflight Analysis and Conversion Engine (PACE) digital diagnostic tool, <https://pacev2.apexcovantage.com/>. PACE helps ensure that figures meet PLOS requirements. To use PACE, you must first register as a user. Registration is free. Then, login and navigate to the UPLOAD tab, where you will find detailed instructions on how to use the tool. If you encounter any issues or have any questions when using PACE, please email PLOS at [figures@plos.org](mailto:figures@plos.org). Please note that Supporting Information files do not need this step.

**Reply:** Thank you for providing the information regarding the PACE digital diagnostic tool. We have followed the instructions and uploaded our figure files to PACE to ensure they meet the PLOS requirements. The tool confirmed that all figures comply with the necessary standards. If there are any additional requirements or adjustments needed, we will be happy to address them promptly. Thank you for your guidance in this process.
